# A Proof of Concept, Phase II Randomized European Trial, on the Efficacy of ALF-5755, a Novel Extracellular Matrix-Targeted Antioxidant in Patients with Acute Liver Diseases

**DOI:** 10.1371/journal.pone.0150733

**Published:** 2016-03-16

**Authors:** Bertrand Nalpas, Philippe Ichaï, Laure Jamot, Nicolas Carbonell, Marika Rudler, Philippe Mathurin, François Durand, Guido Gerken, Michael Manns, Christian Trautwein, Dominique Larrey, Sylvie Radenne, Christophe Duvoux, Vincent Leroy, Jacques Bernuau, Jamila Faivre, Nicolas Moniaux, Christian Bréchot, Gilles Amouyal, Paul Amouyal, Didier Samuel

**Affiliations:** 1 Inserm, Département de l’Information Scientifique et de la Communication, Paris, France; 2 Centre Hépatobiliaire Paul Brousse and Inserm U 1193, Villejuif, France; 3 Hôpital Universitaire Paul Brousse, Villejuif, France; 4 Alfact Innovation, Paris, France; 5 Service Hépato-gastro-entérologie, Hôpital Saint Antoine, Paris, France; 6 Service Hépatologie et de Gastroentérologie, Hôpital La Pitié Salpétrière, Paris, France; 7 Service des maladies de l'appareil digestif, Hôpital Claude Huriez, Lille, France; 8 Service Hépatologie, Hôpital Beaujon, Clichy, France; 9 Gastroenterology and Hepatology Unit, University of Essen, Essen, Germany; 10 Gastroenterology and Hepatology Unit, University of Hanover, Hanover, Germany; 11 Medizinische Klinik III, University Clinic, RWTH-Aachen, Aachen, Germany; 12 Service Hépato-Gastro-Entérologie, Hôpital Saint-Eloi, Montpellier, France; 13 Service Hépatologie et Gastro-Entérologie, Hôpital Croix-Rousse, Lyon, France; 14 Service d'Hépato-Gastro-Entérologie, Hôpital Henri MondorCréteil, France; 15 Département d’Hépato-Gastroentérologie, Hôpital de Grenoble, Grenoble, France; 16 Institut Pasteur, Paris, France; Hvidovre Hospital, DENMARK

## Abstract

**Objective:**

No efficient medical treatment is available for severe acute hepatitis (SAH) except N-acetylcysteine for acetaminophen-induced acute liver failure. The human C-type lectin Reg3α, referred to as ALF-5755, improved survival in an animal model of acute liver failure and was well tolerated in a phase 1 trial in humans. We performed a phase 2a trial of ALF5755 in non-acetaminophen induced SAH.

**Design:**

double-blind, randomized, placebo-controlled study. The primary end-point was the improvement in the coagulation protein synthesis assessed by the change of Prothrombin (PR) during the 72 hours following treatment initiation calculated as PRH0 minus PRH72 divided by 72 (PR slope H0H72). Intention to treat (ITT) and per-protocol (PP) analysis of the entire group and the Hepatitis B virus (HBV)/AIH (auto-immune hepatitis) sub-group were done separately.

**Results:**

57 patients were included. Twenty-eight received ALF-5755, 29 the placebo. Etiologies were: Hepatitis A (n = 10), HBV (n = 13), AIH (n = 9), drug-induced (n = 8), other (n = 17). On the whole group, nor the PR slope H0H72 (0.18±0.31 vs 0.25±0.32), nor the transplant-free survival rate at day 21 (75 vs 86%) differed between groups. Conversely, in the HBV-AIH subgroup, in which ALF was more severe, PR slope H0-H72 was higher in the ALF-5755 arm, the difference being significant in PP analysis (0.048±0.066 vs -0.040±0.099, p = 0.04); the median length of hospitalization was lower in the ALF-5755 group (8 vs 14 days, p = 0.02).

**Conclusion:**

ALF-5755 was not efficient in a ITT analysis performed on the whole sample; however it led to a significant, although moderate, clinical benefit in a PP analysis of the sub-group of patients with HBV or AIH related SAH. As HBV is the major cause of SAH in Asia and Africa and AIH a growing cause, this study emphasizes the need to pursuit the evaluation of this novel medical treatment of SAH.

**Trial Registration:**

ClinicalTrials.gov NCT01318525

## Introduction

Acute liver failure (ALF) is a rare, orphan disease, characterized by a massive cell death and the suppression of the regenerative capacity of the liver, often leading to multi-organ failure and death [1]; this liver destruction is the outcome of a progressive hepatocyte necrosis named severe acute hepatitis (SAH), the frontier between SAH and ALF being the occurrence of a liver encephalopathy which can progress from the mild stage 1 to the highly severe stage 4. The major etiologies of ALF are hepatitis viral infection (HBV, HAV, HEV), auto-immune hepatitis, drug-induced including suicides by acetaminophen overdose. Minor etiologies include neoplastic infiltration, acute Budd—Chiari syndrome, heatstroke, mushroom ingestion, metabolic diseases such as Wilson’s disease and pregnancy; however in about 15% the ALF cause remains unknown [2]. ALF can occur on a healthy liver or be superimposed on a chronic liver disease, i.e a liver already injured. This latter situation often occurs in HBV-related chronic hepatitis during a reactivation phase seen in 15–30% of HBeAg negative [3]. Due to the high HBV prevalence in Africa and Asia, viral hepatitis is the leading cause of ALF in these areas [2]. Another model of acute on chronic liver disease is auto-immune chronic hepatitis (AIH), which was the third cause of ALF in the USA after exclusion of acetaminophen overdose [2] and accounted for 26% of the cases in a recent Argentina series [4].

The exact mechanism of ALF is still not well-known and might vary according the etiology and the underlying liver status. Nevertheless, ALF is characterized by a strong oxidative stress which leads to a massive tissue injury and suppresses liver cell proliferation. Liver oxidative stress results from a strong increase in the production of reactive oxygen and nitrogen species, generated by the causal agents. Several pathways are activated in excess including tumor necrosis factor, TNF-related apoptosis-inducing ligand, CD95 (APO-1/Fas) [5] and finally the complement system which leads to an over production of the proinflammatory anaphylatoxin C5a [6].

Owing to the progressive nature of the liver failure, the main preoccupation of clinicians is to avoid the progression from SAH to ALF and the worsening of encephalopathy when it is already present at time of admission. The general idea is to save time in order to allow the liver to regenerate. In this regard, several treatments have been proposed.

N acetyl cysteine (NAC) administration is an efficient therapy in acetaminophen-induced ALF [7], subjected it is administered early after ALF development. NAC has been reported to be efficient in non-acetaminophen ALF with low grade encephalopathy [8] but there is no complete agreement between authors in this regard [9, 10]. Specific medical treatment according to the ALF etiology have also been tested, such as nucleoside analog therapy in HBV related ALF but the majority of studies did not show any significant improvement in survival [3], or such as corticotherapy in severe AIH related ALF but this did not improve the prognostic [11]. Extracorporeal liver-assist devices and bioartificial devices are also potential ALF therapy; some of them are in evaluation [1] but even they are shown to be efficient they will require a specific and sophisticated environment. Finally, liver transplantation is the ultimate option of ALF treatment when no process has been able to control or to stop the liver failure, ALF now accounting for around 8% of liver transplant activity in Europe and the US [12]. Together, a convenient and efficient treatment exists for acetaminophen induced ALF but there is an unmet therapeutic need for all the ALF non-related to acetaminophen intoxication.

The human secreted C-type lectin Reg3α (also called hepatocarcinoma- intestine-pancreas/pancreatitis-associated protein [HIP/PAP]) is a good candidate in this regard. Indeed *in vitro*, HIP/PAP conferred strong resistance against TNFα-induced apoptosis to pancreas and liver cells and protected against acetaminophen- and Fas-induced ALFs [5, 13]. These exciting properties led to consider the pharmacological use of HIP/PAP through the development of a recombinant (rc) full-length human HIP/PAP protein (rcHIP/PAP). This molecule, referred to as ALF-5755, was tested in an animal model of ALF, the Fas-intoxicated mice, and it improved significantly the survival in a dose-dependent and time-dependent manner [13]; moreover a phase 1 trial demonstrated a good tolerability and pharmacokinetic profile of rcHIP/PAP in healthy subjects [13].

We therefore designed a proof of concept (POC), phase IIa study to evaluate the efficacy of intravenous ALF-5755 to improve liver function as compared with placebo in patients with non-acetaminophen SAH or early stage of ALF.

## Patients and Methods

### Patients

Patients eligible for the study were male and female adult patients with nonacetaminophen severe acute hepatitis and early stage acute liver failure. A full description of the protocol is provided in S1 Text. Patient had to meet all the following inclusion criteria prior to randomization: 1. Early stage acute liver failure or severe acute hepatitis defined as: 15% ≤ Prothrombin rate < 50%, no more than grade I or II encephalopathy, presumed acute illness onset of less than 26 weeks, no evidence of underlying cirrhosis, no known history of chronic hepatitis; 2. Ability to receive first treatment dose within the first 12 hours after biological baseline assessment; 3. Age ≥ 18 and ≤ 75 years; 4. Contraception (only for females of childbearing potential) to be taken throughout the study until D21; 5. A signed written informed consent from patient or from patient's next of kin; 6. Affiliation to social security insurance system.

Exclusion criteria were the following: 1. Acetaminophen-induced hepatitis defined as acetaminophen intake > 4 g/day, at least once in the 7 days prior to baseline; 2. Shock liver (ischemic hepatopathy) or HELLP syndrome or Budd-Chiari syndrome or intrahepatic malignancy; 3. Serum creatinine ≥ 180 μmol/L; 4. Body Mass Index ≥ 35; 5. Sepsis defined as systemic response to proven or suspected infection manifested by two or more of the Systemic Inflammatory Response Syndrome criteria. 6. Uncontrolled active bleeding; 7. Patients who received fresh frozen plasma, PPSB (Prothrombine-Proconvertine-Stuart-B), or vitamin K infusion over the last 48 hours; 8. Patient receiving liver support device treatment, including but not exclusively bioartificial liver, Extracorporeal Liver Assist Device, transgenic pig perfusion; 9. Patient receiving hemodialysis, hemofiltration or hemodiafiltration treatment; 10. Intractable arterial hypotension (arterial systolic blood pressure equal to or below 70 mmHg) present or require inotropic drugs at baseline; 11. Human Immunodeficiency Virus (HIV) positive patient; 12. Active cancer; 13. Pregnancy or breast-feeding; 14. Surgery within 4 weeks prior to baseline, or unsolved surgical disease outside liver transplantation; 15: Patient included in another clinical trial within 4 weeks prior to baseline; 16. Patient with organ or bone-marrow allograft; 17. Absolute contra-indication to liver transplantation.

### Methods

**Fig 1:**
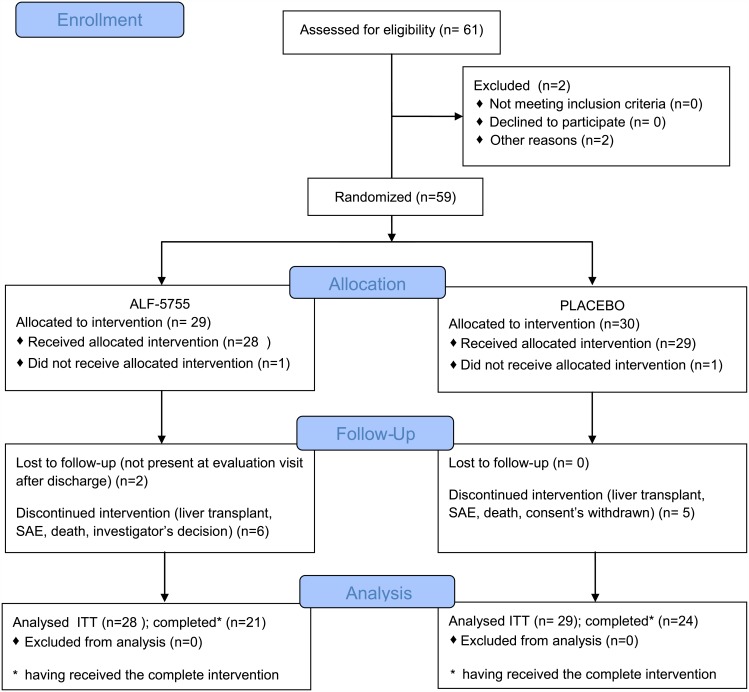
Flow Diagram.

This was a prospective, multicentre, double-blind, placebo-controlled phase IIa study (EudraCT number 2010-020657-14; Clinical trial.gov: NCT01318525). The protocol was approved by the official agencies (S2 and S3 Text), by the “Comité de Protection des Personnes (CPP)” of the Bicêtre hospital (France) on July 2010 (S4 Text) and by the Essen Ethic Committee (Germany) in April 2011(S5 Text). Patients were recruited in 12 Liver units with transplant facilities located in France and in Germany. Written informed consent was obtained from all participants or their next of kin when they were unable to sign. In addition to ALF-5755 or placebo, patients received the best standard treatment on assessment of investigators. The study was opened on October 2010 in France and July 2011 in Germany and closed on April 2013. The flow diagram is presented in Fig 1. Patients were followed-up for adverse events and survival every 12 hours during the infusion period, 12 hours after the last infusion, at day 8 and 21.

### Drug administration

**Fig 2:**
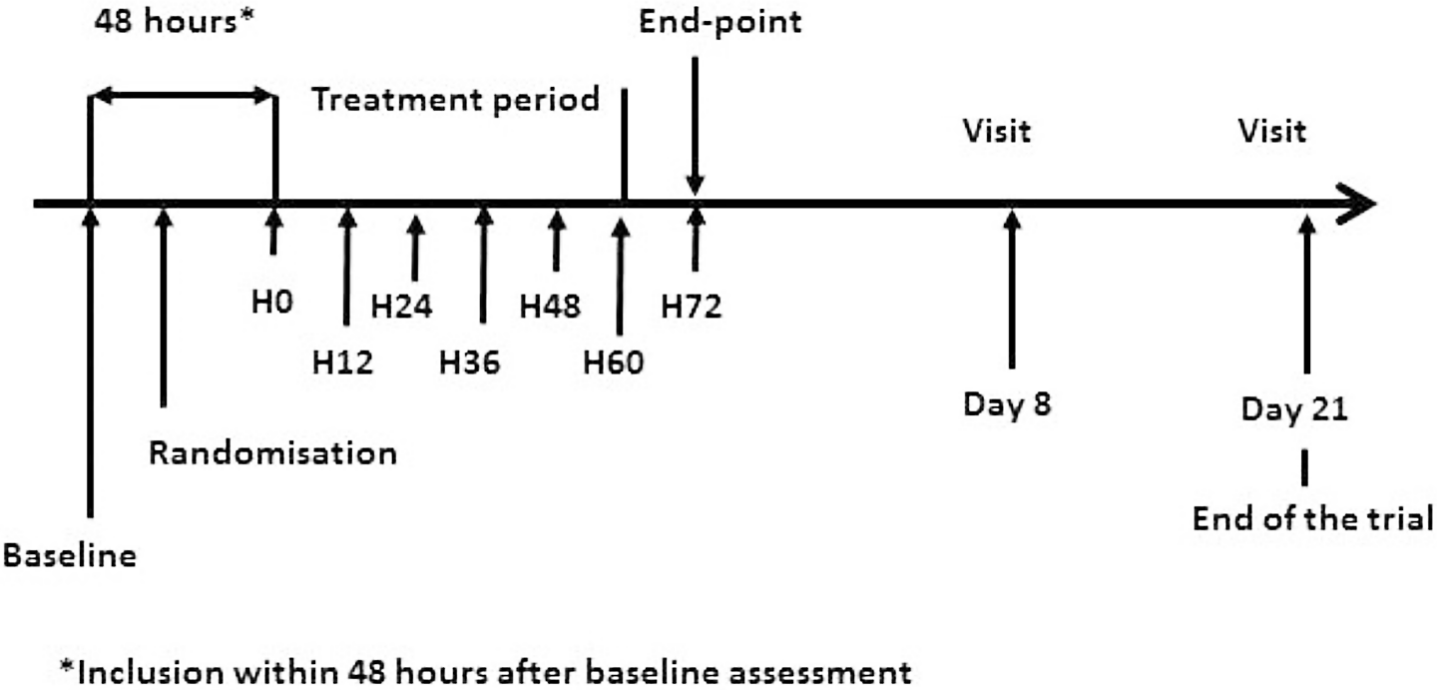
Protocol of drug administration.

The protocol of drug administration (Fig 2) was scheduled according to data obtained in experimental studies on animal and in humans during the phase I clinical trial of ALF-5755 [13]. Patients received slow intravenous infusions of 10 mg (25 ml) of ALF-5755 or placebo (physiological saline solution: 0.9% NaCl) over 10 minutes using automatic syringes every 12 hours over 3 days. Randomisation was in the ratio 1:1 and stratified by site.

### Study endpoints

ALF is a rare disease and the recruitment of patients is slow; for example, 8 years (1998 to 2006) were necessary for Lee et al [8] to recruit 170 patients with non-acetaminophen related ALF, a length not compatible with a POC study. Moreover, as patients with ALF are treated as early as possible, according to the AASLD recommendations published in 2005 [14], using the usual criteria of efficacy in ALF treatment trials, that is the survival rate, would have requested to include a very high number of patients to reach a confident statistical power.

Therefore we look for a surrogate, biological, marker of efficacy. The King’s College [15] and the Clichy [16] criteria are currently used by physicians to decide the transfer to a liver unit with transplantation facilities; they are prognostic markers but they have not been evaluated sequentially as a marker of efficacy of a given treatment. Considering the biological ALF-5755 capacity to confer a resistance to the cellular necrosis as described above, we hypothesized that ALF-5755 might improve the coagulation protein synthesis, a widely used hallmark of liver function. Indeed, in patients with ALD factor VII level at admission and changes in factor V during hospitalization have been reported to be reliable prognostic factors [17] as well as serial prothrombin time in paracetamol induced fulminant hepatic failure [18]. Therefore we decided to assess the ALF-5755 efficacy using the change in prothrombin according to time, a prognostic marker that we evaluated recently in patients with severe ALD [19]; then the primary endpoint was the change in Prothrombin (PR) during 72 hours, referred to as PR slope H0H72, following treatment initiation calculated as the difference between PR at H72 and PR at H0 pre-dose divided by the time elapsed between the two dosages, i.e 72h (*mathematical formula*: *(PRH0-PRH72)/72*). PR was measured every twelve hours. The secondary end-points were the transplant-free survival rate, defined as being alive and not transplanted, at Day 21, a variable proposed early by Lee et al [8], the length of hospitalization, the safety and the rate of change in MELD [20] during 72 hours following treatment initiation. MELD is a scoring system for assessing the severity of chronic liver disease based on the patient's values for serum bilirubin, serum creatinine, and the international normalized ratio for prothrombin time (INR). It usually ranges from less than 10 to more than 40; higher is the value, poorer is the survival prognosis. After the unblinding of the study data the time to reach a prothrombin value equal to 50%, a cut-off above which liver transplant is no more considered, was added as a secondary efficacy analyze. Patients were followed-up until day 21 after inclusion, even those who early discontinued.

### Subgroup analysis

When we analyzed our initial cohort of patients with ALF [19], we noticed that patients with a HBV or a AIH-related ALF behaved differently from the others in terms of PR slope H0H72 (-0.02±0.07 vs 0.30±0.26, p<0.001) and mortality (76.9 vs 36.0%, p<0.001) (Bertrand Nalpas, personal communication), a result not reported in our publication [19]; this result fitted well with reports showing that HBV ALF mortality was higher than that with hepatitis A or E infections [21, 22] and other showing that as AIH related ALF respond poorly to corticosteroids so patients should be systematically placed on the list for transplantation [14]. Hypothesizing that it could be the same in the present prospective study we scheduled to perform a secondary analysis of ALF-5755 efficacy on the specific subgroup of patients with HBV-AIH related ALF, if possible since we could not anticipated the number of patients satisfying this condition who will be included.

### Sample size

The sample size was determined from a series of patients reported elsewhere [19]. The mean and standard deviation of the PR slope H0H72 (0.22±0.27) from this series were assumed for the placebo group. We hypothesized that the PR slope H0H72 in the ALF-5755 would be twice that of the placebo. Based on these data, it was calculated that the required number of patients was 30 per group using a 2-sided 2-sample t-test with an alpha and beta level equal to 5% and 80% respectively.

### Statistical analysis

Quantitative variables were described as mean±SD or median and qualitative values as frequency. PR slope between placebo and active groups on the whole sample and the HBV/HAI subgroup was compared by a non-parametric test (Wilcoxon). Mortality and liver transplantation frequencies were compared by a Chi^2^ test and their time-to-event by a log-rank test. In addition, the comparison was adjusted for sub-groups based on initial PR and etiology (combination of HBV and AIH etiologies, see the study endpoint section) using the Cochran-Mantel-Haenszel test. A complete description of the statistical analysis plan is provided in S6 Text. Adjustment on hepatic encephalopathy could not be done owing to the small number of patients displaying this complication (see the results section). In a post-hoc analysis we compare the time to event (Kaplan plot and log rank) for achieving a Prothrombin value of 50%. A p-value below 0.05 was considered as significant. Statistical analyses were performed using SAS^®^, Version 9.2 (SAS Institute, Cary, Northern Carolina, USA).

## Results

Fifty-nine patients were randomized, of which 57 patients were treated (2 patients were randomized by mistake and did not receive treatment) and constituted the ITT population; 28 received the study drug and 29 the placebo. There were 33 men and 24 women aged of 41.7±15.8 y. Their main characteristics are presented in Table 1; the incidence of hepatic encephalopathy was rather low (around 10%) and the MELD score was around 25, thus the series was made of patients with moderately severe ALF.

**Table 1:** Main characteristics of the studied patients at time of inclusion.

	ALF-5755	Placebo	p
N	28	29	
M/W	9/21	14/15	NS
Age (Mean±SD)	43.5±14.8	40.0±15.3	NS
BMI (Mean±SD)	24.5±5.0	23.3±4.4	NS
Etiology (N patients)			NS
HAV	4	6	
HBV	9	4	
AIH	4	5	
Drug induced	4	4	
Unknown^1^	3	5	
Others	4	5	
Concomitant NAC^2^ (N, %)	23 (82.1)	24 (82.7)	NS
Hepatic encephalopathy (N, %)	3 (10.3)	3 (11.1)	NS
Bilirubin	303 (17–612)	212 (18–511)	NS
ASAT	2157 (98–27033)	1440 (123–8643)	NS
ALAT	2296 (190–7215)	3191 (415–7951)	NS
Prothrombin rate (Median, range)	34 (16–49)	35 (17–49)	NS
INR (Median, range)	2.2 (1.5–4.5)	2.2 (1.2–4.4)	NS
MELD (Median, range)	26(14–40)	25 (14–40)	NS

^1^. despite complete investigation;

^2^. N acetyl cysteine.

Twenty-two patients had a HBV or a AIH related ALF, among 13 were treated and 9 received the placebo (difference NS). They were no significant differences between the two groups in terms of the disease severity nor between demographic parameters except for the proportion of caucasian patient which was higher in the ALF-5755 group. All, except two (1 in each arm), patients with HBV related ALF received an antiviral treatment and all those with AIH received corticosteroids; NAC was administered to 23 (82.1%) and 24 (82.7%) of the ALF-5755 and the placebo groups, respectively. None of the patients received treatments that could affect the PR.

The Intention to treat (ITT) population was made of all patients having received at least one infusion of the study drug or the placebo, that is the 57 patients included. Among these, 51 received the six infusions and were analyzed apart as per protocol (PP); the reasons why 6 patients did not completed the treatment were liver transplant in 4 and severe SAE in 2.

### Impact of ALF-5755 on the total population

The analysis of PR slope H0-H72 showed no significant changes between the ALF-5755 and the placebo group for the ITT population (0.18±0.31 vs 0.25±0.32, NS) (Table 2).

**Table 2:** Comparative efficacy between ALF-5755 and placebo on the PR slope from H0 to H72 and the survival without liver transplantation.

Condition	ALF-5755	Placebo	p
ITT*	28	29	
PR slope H0-H72	0.18±0.31	0.25±0.32	NS
LT free (N, %)	21 (75)	25 (86.2)	NS
Days in hospital (median, range)	7[1.5–83]	10 [2.5–85]	NS
PP**	25	26	
PR slope H0-H72	0.21±0.26	0.27±0.32	NS
LT free (N, %)	21 (84)	23(88.5%)	NS
Days in hospital (median, range)	7 [5–83]	10 [5–85]	NS

* intention to treat;

** per protocol: administration of the 6 infusions.

Eleven patients (19.6%) were liver transplanted or died during the 21 days of follow-up and the transplant-free survival rate at day 21 did not significantly differ between the active drug (75%) and the placebo (86.2%) and so it was for the time to event using the log-rank test (p = 0.14). The median number of days in the hospital for the ITT population alive with no transplant was slightly lower the ALF-5755 group (10 days) compared to the placebo group (14 days) but the difference was not significant.

Among the 6 patients with HE at baseline, 3 (2 receiving ALF-5755 and 1 the placebo) were liver transplanted within 5 days following admission while HE had disappeared at H72 in the remaining 3; in both the active and the placebo group, no HE occurred in those patients without HE at admission.

### Pharmacokinetics

Pharmacokinetic studies of ALF-5755 were performed in a subset of 8 patients randomly selected. While C_max_ was stable from the first to the sixth infusion (2144.00 ng/mL versus 2189.33 ng/mL), T_1/2_ increased from 5.32 hours at the first infusion to 17.58 hours at the sixth infusion. AUC_0-12_ increased from 3085.83 ng/mL at the first infusion to 3563.59 ng/mL at the sixth infusion.

### Prognostic value of PR slope H0H72

The cut-off predictive value of PR slope H0H72 for survival was reported to be 0.1 [19]. Among the 57 patients studied, 29 (50.8%) had a PR slope H0H72 equal to or higher than 0.1, and all of them survived without liver transplantation. Conversely, 28 (49.2%) had a value <0.1 and 11 either died or were liver-transplanted. Therefore the positive predictive value of a PR slope H0H72 was equal to 1 in this series (the maximum value expected) while among those who had a negative screening test, the probability of being disease-free (i.e the negative predictive value) was 0.39 (maximum value = 1).

### Impact of ALF-5755 on the HBV-AIH sub-group

This sub-group was made of 22 patients (13 ALF-5755 and 9 placebo) of which 18 (10 ALF-5755 and 8 placebo) received the 6 infusions. As hypothesized, the HBV-AIH related SAH subgroup had a different behavior than the SAH related to other known etiologies. Patients from the HBV-AIH group were older (47.7±12.4 vs 38.0±15.6, p = 0.02) and had a more severe SAH than their counterparts on admission as stated by a higher bilirubin level (394±133 vs 182±165 μmol/l, p<0.001) and a higher MELD score (28.5±6.0 vs 23.2±5.9, p = 0.002). These two groups also differed on the transplant-free survival rate which was significantly lower in the HBV-HAI group (63.6% vs 91.4%, p = 0.013); moreover in the HBV-AIH subgroup the mean PR slope H0-H72 was flat or even negative while it was positive, about + 0.3 in the other etiologies (Table 3).

**Table 3:** Values of PR slope from H0 to H72 according to the etiology of the acute liver failure.

Etiology	N	PR slope H0-H72	Survival rate(%)
HAV	10	0.36±0.16	100
Drug	8	0.30±0.36	87.5
Other	9	0.31±0.25	87.5
Unknown	8	0.47±0.41	88.9
*All combined*	*35*	*0*.*36±0*.*29*	*91*.*4*
HBV	13	-0.02±0.13	76.9
HAI	9	-0.0±0.05	44.4
*All combined*	*22*	*-0*.*01±0*.*10*	*63*.*6*

In the HBV-AIH group the mean PR slope H0-H72 was two times higher in the ALF-5755 arm (n = 13) as compared to placebo (n = 9) (Table 4) and the difference was significant (0.048±0.066 vs -0.040±0.099, p = 0.04; mean difference = -0.088, CI 95% [-0.171–0.004], effect size = 1.09) when analysis was done in the 18 patients having received the complete treatment (PP).

**Table 4:** ALF-5755 efficacy in HBV or AIH related severe acute hepatitis.

Condition	ALF-5755	Placebo	p
**ITT** *(n = 22)	13	9	
PR slope H0-H72	0.001±0.11	-0.032±0.09	NS
LT free (N, %)	8 (61.5)	6 (66.7)	NS
Days in hospital (median, range)	7 [1.5–18]	14 [2.5–85]	0.02
PP** **(n = 18)**	10	8	
PR slope H0-H72	0.048±0.066	-0.040±0.099	0.043
LT free (N, %)	8 (80)	6(75%)	NS
Days in hospital (median, range)	8 [5–18]	16 [8–85]	0.03

* intention to treat;

** per protocol: administration of the 6 infusions.

Although the transplant-free survival rate did not differed between ALF-5755 and placebo (80 vs 75%, NS), the median number of days in the hospital in non-transplanted patients was significantly (p = 0.02) lower in the ALF-5755 group (8 days) compared to the placebo group (14 days).

**Fig 3:**
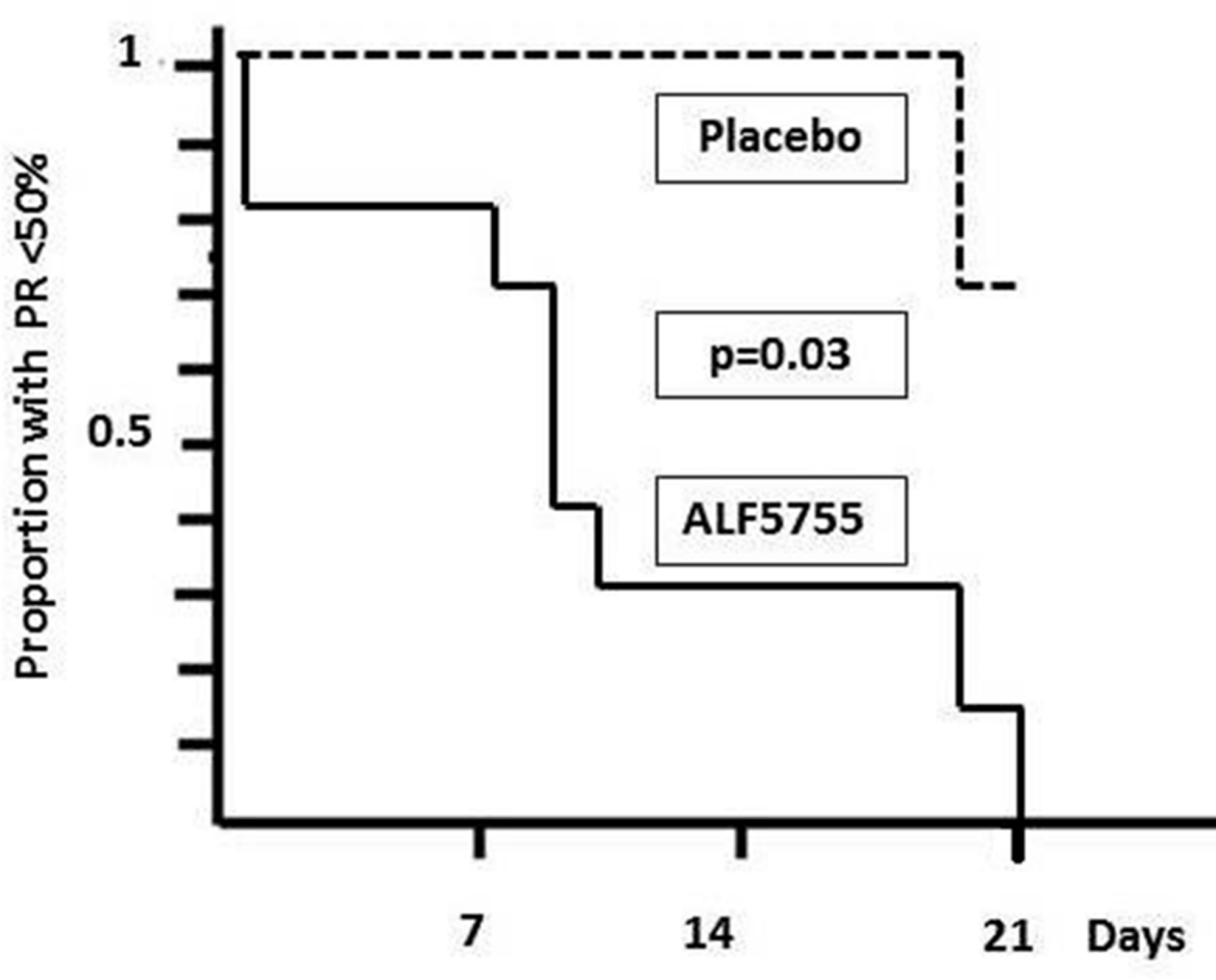
Median time for patients to reach a PR rate > = 50% in the HBV-HAI patients receiving ALF-5755 (full line) or placebo (dotted line). Analysis was performed on the ITT group (n = 22). Comparison by log-rank test was significant at the 0.03 level.

Finally the median time for patients treated with ALF-5755 to reach a PR rate > = 50%, usual cut-off value of severe hepatic failure [25], was 8 days, a length significantly lower (p = 0.03) than that of the placebo group of which median time was greater than the cut off time of 21 days (Fig 3).

### Safety

A total of 262 treatment-emergent adverse events (TEAE), among which the majority was mild or moderate severity, were reported in 55 of the 57 patients (Table 5).

**Table 5:** Treatment Emergent Adverse Events.

	ALF-5755 (N = 28)	Placebo (N = 29)	Total (N = 57)
	n N (%)*	n N (%)	n N (%)
Treatment Emergent Adverse Events	133 28 (100.0%)	129 27 (93.1%)	262 55 (96.5%)
Severe Treatment Emergent Adverse Events	27 10 (35.7%)	17 12 (41.4%)	44 22 (38.6%)
Serious Treatment Emergent Adverse Events	8 5 (17.9%)	11 7 (24.1%)	19 12 (21.1%)
Drug-Related Treatment Emergent Adverse Events	18 7 (25.0%)	9 7 (24.1%)	27 14 (24.6%)
Serious Drug-Related Treatment Emergent Adverse Events	2 2 (7.1%)	6 4 (13.8%)	8 6 (10.5%)
Treatment Emergent Adverse Events leading to withdrawal	0 0	4 2 (6.9%)	4 2 (3.5%)
Severity			
CTCAE** grade 1 or Mild	86 25 (89.3%)	67 23 (79.3%)	153 48 (84.2%)
CTCAE grade 2 or Moderate	20 10 (35.7%)	45 18 (62.1%)	65 28 (49.1%)
CTCAE grade 3 or Severe	18 10 (35.7%)	13 11 (37.9%)	31 21 (36.8%)
CTCAE grade 4 or Life Threatening	8 3 (10.7%)	3 3 (10.3%)	11 6 (10.5%)
CTCAE grade 5 or Death	1 1 (3.6%)	1 1 (3.4%)	2 2 (3.5%)

* n = number of events,

N = number of subjects,

% = percentage of subjects;

**CTCAE = Common Terminology Criteria for Adverse Events.

The majority of TEAEs were reported for metabolism and nutrition disorders (57.9%), gastrointestinal disorders (45.6%), general disorders and administration site conditions (26.3%), nervous system disorders (21.1%), and blood and lymphatic system disorders (17.5%) (S1 Table). Ten (35.7%) patients were reported with twenty-seven severe adverse events (SAEs) in the ALF-5755 group, and twelve (41.4%) patients in the placebo group were reported with 17 SAEs. Two patients in the placebo group, and none in the ALF-5755 group, were reported with four adverse events that led to withdrawal of treatment. Complete results of the trial are provided in the S7 Text.

## Discussion

ALF-5755 offers a novel and original approach to liver failure treatment based on the reduction of cellular necrosis and stimulation of liver cell proliferation. Although we found no significant differences between the ALF-5755 group and the placebo group with respect to the primary end point when the analysis was done on the whole sample, ALF-5755 might be of potential interest in a specific subgroup of patients with HBV-AIH related severe SAH.

The lack of global clinical efficacy that we observed could be due to a number of factors. Firstly, our series was characterized by a high rate of survival, 80.7%, while it was 52% in another of patients resembling to ours, i.e. with non-acetaminophen induced low grade ALF [8]; therefore such a striking natural improvement hampered any demonstration of the potential efficacy of the treatment in evaluation. Such hypothesis is reinforced by the results obtained in the most severe sub-group of our series, that is the patients with HBV or AIH related SAH, although we agree that these results should be taken with caution due to the small size of this sub-group. Indeed, this sub-group was characterized by lower survival rate as compared to the remaining counterpart, in accordance with previous data [22], and by a flat PR slope H0H72. The reasons explaining these differences in outcome according to the etiology are not well understood however one could suspect that in the HBV and AIH sub-group, the acute liver failure might well occur on a liver previously and chronically injured; since both HBV and AIH are known to lead chronic hepatitis, those patients could well have been diagnosed as having a non cirrhotic Acute on Chronic Liver Failure (ACLF), a syndrome recently revisited [23]. In our protocol, a history of chronic hepatitis was indeed an exclusion criteria but histological evaluation of the 6 liver biopsies from HBV or AIH patients available at the time of transplantation showed features of chronic hepatitis and thus did confirm the diagnosis of ACLF. Therefore, in the specific sub-group of HBV-AIH related ACLF, the active drug ALF-5755 significantly improved the PR slope as compared to the placebo, although in the PP analysis only; this could well be due to the specific ALF-5755 pharmacological properties since the transition from a chronic liver disease to ACLF is associated with an increase in proinflammatory cytokine [24] leading to necrosis of liver cells, as well as fibrosis and cholestasis. As a consequence, the time period to reach a prothrombin rate higher than 50%, a value above which the inscription on a waiting list for liver transplantation is not considered [25], was significantly shortened. Finally the duration of hospital stay was decreased but there was no significant benefit on survival. Although obtained on a short series these results suggest that ALF-5755 could improve the ACLF outcome through boosting the liver regeneration process when it is slowed down or even stopped.

The second reason of the global lack of efficacy might come from a non-adapted therapeutic administration schedule. The chosen dose, 10mg per infusion, was based on data obtained in experimental models after verification it was safely tolerated in humans. The timing of infusion was based on pharmacokinetic data obtained in the phase I study [13]; given a terminal plasma half-life around 4.5 hours, the administration was scheduled every 12 hours. However, pharmacokinetics studies performed in the present phase II study showed a residual accumulation all along the 6 infusions and a sharp increase in the T1/2 which was three times higher at the sixth infusion as compared to the first one. ALF 5755 is acting as both a survival factor and a mitogenic molecule. Also, this is a C-type lectin which binds the fibrin network of the extracellular matrix and targets to the matrix its strong and intrinsic antioxidant action. Thus, interestingly, binding to the extracellular matrix specifically occurs upon liver inflammation and has been shown to last for at least 24 hours upon a single infusion [26]. These modes of actions and the pharmacological pattern may indeed offer the possibility to optimize the administration schedule.

Although not devoted to evaluate the performance of biomarkers of survival, our prospective study confirms the performance of the PRslope from the admission to the third day of hospitalization as a prognostic marker in SAH [19]. Indeed, in that series, the positive predictive value of a PR slope > = 0.1 was equal to 1, i.e the maximum probability, while the predictive negative value was 0.39. It is not however an “early” prognostic marker since it can be calculated only three days following admittance and therefore could not replace the scores assessing the severity on admission such as the Meld [20] score. But from the third day, subjected the patients is still alive, using the PR slope appears to be convenient owing to its simplicity.

## Conclusions

In conclusion, although ALF-5755 was not efficient on the whole sample our results identify a sub-group of ALF patients, i.e. those in whom the liver regeneration process is under arrest, specifically with HBV or AIH related ACLF in our series, for whom ALF-5755 administration led to a significant, although moderate, clinical benefit. These results obtained on small sample need to be confirmed in a larger series but as ALF-5755 is an original medical treatment with respect to its modes of action and pharmacological pattern and as HBV is the major cause of ACLF in Asia and Africa and AIH a growing cause, this study emphasizes the interest and the need to pursuit the evaluation of ALF-5755 in ACLF treatment.

## Supporting Information

S1 CONSORT Checklist (DOCX)

S1 Table Treatment-Emergent Adverse Events. (DOC)

S1 Text Study Protocol. (PDF)

S2 Text Administrative approval France. (PDF)

S3 Text Administrative approval Germany. (PDF)

S4 Text Ethical Approval France. (PDF)

S5 Text Ethical Approval Germany. (PDF)

S6 Text Statistical analysis plan. (PDF)

S7 Text Clinical study report. (PDF)
