# Supplementary material for: A Proof of Concept, Phase II Randomized European Trial, on the Efficacy of ALF-5755, a Novel Extracellular Matrix-Targeted Antioxidant in Patients with Acute Liver Diseases
Source: PLoS One. 2016 Mar 16;11(3):e0150733. doi: 10.1371/journal.pone.0150733 (PMC4794150; doi:10.1371/journal.pone.0150733)
Supplement: S1 Table — (DOC) [file pone.0150733.s002.doc]

**Supplementary file S1: Treatment-Emergent Adverse Events in ALF-5755 and placebo groups**

|  | **ALF-5755 (N=28)** | **Placebo (N=29)** | **Total (N=57)** |
| --- | --- | --- | --- |
|  | **n N (%)** | **n N (%)** | **n N (%)** |
| Any Adverse Event | 133 28 (100.0%) | 129 27 (93.1%) | 262 55 (96.5%) |
| Metabolism and nutrition disorders | 34 16 (57.1%) | 37 17 (58.6%) | 71 33 (57.9%) |
| Gastrointestinal disorders | 28 16 (57.1%) | 21 10 (34.5%) | 49 26 (45.6%) |
| Blood and lymphatic system disorders | 8 4 (14.3%) | 16 6 (20.7%) | 24 10 (17.5%) |
| Nervous system disorders | 9 5 (17.9%) | 8 7 (24.1%) | 17 12 (21.1%) |
| General disorders and administration site conditions | 7 6 (21.4%) | 9 9 (31.0%) | 16 15 (26.3%) |
| Investigations | 10 4 (14.3%) | 5 4 (13.8%) | 15 8 (14.0%) |
| Infections and infestations | 11 5 (17.9%) | 3 3 (10.3%) | 14 8 (14.0%) |
| Skin and subcutaneous tissue disorders | 4 3 (10.7%) | 6 5 (17.2%) | 10 8 (14.0%) |
| Psychiatric disorders | 3 3 (10.7%) | 6 6 (20.7%) | 9 9 (15.8%) |
| Renal and urinary disorders | 4 4 (14.3%) | 4 3 (10.3%) | 8 7 (12.3%) |
| Cardiac disorders | 4 4 (14.3%) | 3 3 (10.3%) | 7 7 (12.3%) |
| Vascular disorders | 6 5 (17.9%) | 1 1 (3.4%) | 7 6 (10.5%) |
| Respiratory thoracic and mediastinal disorders | 2 2 (7.1%) | 3 3 (10.3%) | 5 5 (8.8%) |
| Musculoskeletal and connective tissue disorders | 1 1 (3.6%) | 3 3 (10.3%) | 4 4 (7.0%) |

n=number of events, N=number of subjects, %=percentage of subjects
