# Supplementary material for: A Proof of Concept, Phase II Randomized European Trial, on the Efficacy of ALF-5755, a Novel Extracellular Matrix-Targeted Antioxidant in Patients with Acute Liver Diseases
Source: PLoS One. 2016 Mar 16;11(3):e0150733. doi: 10.1371/journal.pone.0150733 (PMC4794150; doi:10.1371/journal.pone.0150733)
Supplement: S1 Text — (PDF) [file pone.0150733.s003.pdf]

CLINICAL STUDY PROTOCOL - CONFIDENTIAL

**Protocol Number:**	**ALF-5755_P2_ALF**
**EudraCT Number:**	2010-020657-14
**Investigational Product:**	ALF-5755
**Title:**	A multicentre, double-blind, randomized, placebo-controlled study to evaluate the efficacy and the safety of ALF-5755 in patients with nonacetaminophen severe acute hepatitis and early stage acute liver failure
**Version and Date:**	Final Version 01; 07 May 2010
**Sponsor:**	Alfact Innovation
**Sponsor's Medical Contacts:**	Dr Gilles Amouyal, Chief Executive Officer<br>Dr Paul Amouyal, President<br>Alfact Innovation, 320 rue Saint Honoré,<br>75001 Paris<br>Phone: +33 1 53 40 87 97

ORION
Clinical Services Ltd.
7 Bath Road
Slough
Berkshire
SL1 3UA
UK

2. SIGNATURE PAGE

Title: A multicentre, double-blind, randomized, placebo-controlled study to evaluate the efficacy and the safety of ALF-5755 in patients with nonacetaminophen severe acute hepatitis and early stage acute liver failure

Sponsor

Paul Amouyal, MD
President
Alfact Innovation
Tel: +33 1 45 59 35 66
Fax: + 33 1 53 40 60 52
e-mail: amouyal.paul@wanadoo.fr

____
Signature

____
Date

Principal Investigator

Didier Samuel, MD PhD
Centre Hépatobiliaire
Hôpital Paul Brousse, Villejuif
Tel: +33 1 45 59 34 03
Fax: + 33 1 45 59 38 57
e-mail: didier.samuel@pbr.aphp.fr

____
Signature

____
Date

Project Manager

Nathalie Ernault Roseau
Head of Clinical Operations
ORION Santé
Tel: +33 (0) 1 40 83 40 83
Fax: +33 (0) 1 40 83 40 84
e-mail: nathalie.ernault-roseau@orioncro.com

____
Signature

____
Date

Statistician

Dr Bertrand Nalpas
Director of Research
Inserm
Tel: +33 (0) 6 80 90 59 11
e-mail: bertrand.nalpas@gmail.com

____

Signature

____

Date

3. SYNOPSIS

Company / Sponsor:	Alfact Innovation<br>320 rue Saint Honoré, 75001 Paris, France
Name of finished product:	ALF-5755
Name of active ingredient:	rhHIP/PAP
Study title:	A multicentre, double-blind, randomized, placebo-controlled study to evaluate the efficacy and the safety of ALF-5755 in patients with nonacetaminophen severe acute hepatitis and early stage acute liver failure.
Protocol number:	ALF-5755_P2_ALF
Clinical phase:	Phase II
Principal investigator:	Dr Didier Samuel, Centre Hépatobiliaire, Hôpital Paul Brousse, Villejuif, France
Centres:	10 to 19 centres in France
Rationale:	Acute liver failure is a rare but dramatic disease of multiple etiologies, often affecting young subjects, marked by the sudden loss of hepatic function in a person without preexisting liver disease (Lee, Squires et al. 2008), and leading to a rapid deterioration of the liver functions with presence of coagulopathy and hepatic encephalopathy.<br>Acute liver failure is a life-threatening disease with a high rate of mortality in absence of liver transplantation, except for acetaminophen-induced acute liver failure for which N-acetyl cysteine is a registered antidote.<br>ALF-5755, rhHIP/PAP, a C-type lectin, has been shown to promote cell survival after apoptotic or oxidative stress, and hepatocytes' regeneration in primary cultures and *in vivo*. ALF-5755 significantly increased survival in the Fas mediated acute liver failure model in mice, and had very good tolerance up to the maximal tested dose of

	20 mg in a phase I healthy volunteer study.<br><br>ALF-5755 may become, in this dramatic disease with high unmet medical need, a future therapy for the treatment of patients suffering from severe acute hepatitis (SAH) and acute liver failure (ALF) not due to acetaminophen overdose, where liver transplantation is the sole treatment in the absence of spontaneous recovery.
Objectives:	Primary:<br>- to evaluate the efficacy of ALF-5755 versus placebo, measured by rate of change of prothrombin ratio (PR) during the 72 hours following treatment initiation, in patients with nonacetaminophen SAH and early stage ALF.<br><br>Secondary:<br>- to evaluate the safety and tolerability of ALF-5755 versus placebo in patients with nonacetaminophen SAH and early stage ALF.<br>- to determine the pharmacokinetic (PK) parameters of ALF-5755 versus placebo in patients with nonacetaminophen SAH and early stage ALF.
Dose and duration of treatment:	10 mg (25 ml) every 12 hours over 3 days
Route and mode of administration:	Slow intravenous infusion over 10 minutes using automatic syringes
Form:	Lyophilized (freeze-dried) powder, to be reconstituted with water for injection
Study design:	Prospective, multicentre, double-blind, placebo-controlled study of ALF-5755 in patients with nonacetaminophen SAH and early stage ALF, randomized in the ratio 1:1.<br><br>A minimum of 60 patients will be recruited into the study in the following two treatment groups:<br>- Group A: approximately 30 patients will receive ALF-5755<br>- Group B: approximately 30 patients will receive placebo (physiological saline solution: 0.9% NaCl)<br><br>Patients will receive 10 mg (25 ml) of ALF5755 or placebo every 12 hours over 3 days in slow intravenous infusions over 10 minutes using automatic syringes.<br><br>Recruitment will continue until 60 patients are evaluable, i.e. have no major deviation impacting the evaluability of the primary endpoint

	and have PR assessments allowing rate of change of PR calculation.
Diagnosis and main criteria for inclusion / exclusion:	Patients included in this study should have nonacetaminophen SAH and early stage ALF as confirmed by medical history, physical and biological examinations prior to inclusion. Patients should be 18 to 65 years old and willing or patient's next of kin willing to give written informed consent.<br><br>**Inclusion criteria:**<br><br>Patient must meet **all inclusion criteria** prior to randomization:<br><br>1. A signed written informed consent from patient or from patient's next of kin<br>2. Early stage acute liver failure OR severe acute hepatitis defined as:<br>- $15\% \leq PR < 50\%$<br>- No hepatic encephalopathy, OR grade I or II encephalopathy (Appendix E)<br>- Presumed acute illness onset of less than 26 weeks<br>- No evidence of underlying chronic liver disease<br>3. Patient who can receive first treatment dose within the first 12 hours after biological baseline assessment<br>4. Age ≥ 18 and ≤ 65 years<br>5. Contraception (only for females of childbearing potential) to be taken throughout the study until D21. Sole mechanic contraceptives, such as condoms, are advised. Note: Oral contraceptives may have contraindications in case of severe acute hepatitis and acute liver failure<br>6. Patient affiliated to social security insurance system.<br><br>**Exclusion criteria:**<br><br>Patients who meet **any of the following exclusion criteria** are not eligible for randomization:<br><br>1. Acetaminophen-induced hepatitis defined as acetaminophen intake > 4 g/day, at least once in the 7 days prior to baseline

	2. Shock liver (ischemic hepatopathy) OR HELLP syndrome OR Budd-Chiari syndrome OR intrahepatic malignancy<br>3. Serum creatinine ≥ 180 µmol/L<br>4. Body Mass Index (BMI) ≥ 35<br>5. Sepsis defined as systemic response to proven or suspected infection manifested by two or more of the Systemic Inflammatory Response Syndrome (SIRS) criteria (i.e. Temperature > 38°C or < 36°C; Heart rate > 90 beats/min; Respiratory rate > 20 breaths/min or $PaCO_2$ < 32 mgHg; white blood cell (WBC) count > 12.000 cells/mm^3, < 4.000 cells/mm^3, or > 10% immature (band) forms) at baseline<br>6. Uncontrolled active bleeding<br>7. Patients who received fresh frozen plasma, PPSB (Prothrombine-Proconvertine-Stuart-B), or vitamin K infusion over the last 48 hours<br>8. Patient receiving liver support device treatment, including but not exclusively bioartificial liver (BAL), Extracorporeal Liver Assist Device (ELAD), transgenic pig perfusion<br>9. Patient receiving hemodialysis, hemofiltration or hemodiafiltration treatment<br>10. Intractable arterial hypotension (arterial systolic blood pressure equal to or below 70 mmHg) present or require inotropic drugs at baseline<br>11. Human Immunodeficiency Virus (HIV) positive patient<br>12. Active cancer<br>13. Pregnancy or breast-feeding<br>14. Surgery within 4 weeks prior to baseline, or unsolved surgical disease outside liver transplantation.<br>15. Patient included in another clinical trial within 4 weeks prior to baseline<br>16. Patient with organ or bone-marrow allograft<br>17. Absolute contra-indication to liver transplantation.

Sample size:	A minimum of 60 patients (approximately 30 patients per group)
Study population:	Patients with nonacetaminophen SAH and early stage ALF will be randomized to either Group A or Group B.<br>The randomization ratio will be 1:1.
Dosing regimen:	Patients will receive study treatment every 12 hours over 3 days, either placebo or ALF-5755, according to the following dosage schedule.<br>The time between biological baseline assessment and first injection of ALF-5755 or placebo should be as short as possible, but should not exceed 12 hours.

Group A	Group B
ALF-5755:<br>10 mg (25 ml) given in slow intravenous infusion over 10 minutes with an automatic syringe<br>at H0, H12, H24, H36, H48 and H60	Placebo: physiological saline solution (0.9% NaCl)<br>25 ml given in slow intravenous infusion over 10 minutes with an automatic syringe<br>at H0, H12, H24, H36, H48 and H60

	Patients should continue to receive (in addition to ALF-5755 or placebo) the best standard treatment on assessment of investigators.
Number of planned visits:	11 visits including baseline assessment
Study duration:	A baseline assessment (prior to randomization); followed by a treatment period and a mandatory hospital inpatient follow-up from H0 to H84 (after first infusion) and a visit on D8 (after H0). The End of Study visit will be on D21 (after H0).<br>Patients will remain in the clinical hospital unit under permanent medical and nursing supervision for 4 days, i.e. up to 24 hours after the last infusion, and will undergo an inpatient or outpatient hospital visit at both D8 and D21.

Efficacy endpoints and parameters:	Primary endpoint:<br>- Rate of change of PR during 72 hours following treatment initiation<br>Secondary endpoints:<br>- Rate of change of Factor V (FV) plasma level during 72 hours following treatment initiation<br>- Rate of change of international normalized ratio (INR) during 72 hours following treatment initiation<br>- Rate of change of alanine transaminases (ALT) plasma level during 72 hours following treatment initiation<br>- Rate of change of aspartate transaminases (AST) plasma level during 72 hours following treatment initiation<br>- Change of Hepatic Encephalopathy Grade (HE grade) during 72 hours following treatment initiation<br>- Overall survival rate at D21<br>- Transplant-free survival rate at D21<br>- Liver transplant rate at D21<br>- Length of hospitalisation (days)<br>Exploratory endpoint:<br>- Total and cleaved cytokeratin 18 plasma levels (CK18)
Safety endpoints and parameters:	To determine the safety and tolerability of ALF-5755, evaluations will include as described in the Study Flow Chart (Appendix C):<br>A. Comparison of the frequency of:<br>1. local and systemic adverse events (AEs/serious AEs [SAEs]) (AEs should be recorded from the timepoint that the patient informed consent document [PICD] has been signed),<br>B. Assessment of:<br>1. laboratory findings (including haematology, biochemistry and urinalysis),<br>2. clinical examination, clinically significant vital signs (blood pressure [BP], heart rate [HR], respiratory rate [RR]); body temperature,<br>3. HE grades (Appendix E) and Glasgow Coma Scores (GCSs) (Appendix F),<br>4. electrocardiogram (ECG) abnormalities,

	5. anti-ALF-5755 antibody plasma levels,<br>6. Beta human chorionic gonadotropin (hCG) blood pregnancy test in women of childbearing potential,<br>7. Arterial blood gas in case of grade I or II hepatic encephalopathy or GCS < 15.<br><br>N.B. Haematology will include: haemoglobin, hematocrit, complete WBC count, red blood cell (RBC) count and platelet count. Biochemistry will include: PR, FV, INR, fibrinogen, ALT, AST, alkaline phosphatase, Gamma glutamyltransferase (GGT), total and conjugated bilirubin, ammonia, albumin, creatinine, urea, sodium, potassium, chloride, bicarbonates, lactates, calcium, magnesium, phosphorus, glucose, total proteins, amylase and lipase, and urinalysis: glucose, proteins, nitrites, ketones, leucocytes, blood, density and pH.<br><br>C. Other baseline parameters performed at baseline only include:<br>1. weight, height, BMI,<br>2. acetaminophen plasma level,<br>3. alcohol plasma level,<br>4. screening of acute and chronic viral infections (serology and/or polymerase chain reaction [PCR]): Hepatitis A Virus (HAV), Hepatitis B Virus (HBV), Hepatitis C Virus (HCV), Hepatitis D Virus (HDV), Hepatitis E Virus (HEV), Herpes Simplex Virus (HSV), VZV, HIV<br>5. autoimmune markers (antinuclear antibody [ANA], anti-smooth muscle antibody [ASMA], antibodies to liver and kidney microsomes [anti-LKM], immunoglobulin [Ig] levels),<br>6. cupremia and ceruloplasmin plasma levels, and cupruria.
Pharmacokinetic endpoints and parameters:	Plasma blood levels of ALF-5755 will be determined at several times during infusions in the first 16 patients included at Paul Brousse Hospital and Beaujon Hospital (8 patients per centre) (refer to the Pharmacokinetics Schedule [Appendix D]). This full PK evaluation in patients after the first and the last infusion will allow PK information to be obtained in patients in order to study possible modifications of ALF-5755 PK due to the patient’s condition and to quantify a potential accumulation after repeated administration.<br><br>Baseline plasma concentrations measured at H0, reflecting endogenous hepatocarcinoma-intestine-pancreas/pancreatitis-associated protein (HIP/PAP) levels will be subtracted from each ALF-5755 plasma concentration.

	PK parameters (C_{max}, t_{max}, AUC_{0-12}, AUC_{0-t} and $t_{1/2}$) of ALF-5755 will be determined from plasma concentrations measured at H0 after the first infusion and at H60 after the 6th infusion.
Statistical methods:	Rate of change of PR, FV, INR, ALT and AST during 72 hours following treatment initiation in ALF-5755 and placebo arms will be compared by a non parametric test (Wilcoxon).<br>Mortality and liver transplantation frequencies will be compared by a Chi² test and their time-to-event by a log-rank test.<br>The achievement of steady-state will be assessed by comparison of ALF-5755 concentrations on H48, H60 and H72. Accumulation will be assessed by comparison of AUC_{0-12} between H0 and H12 and AUC_{0-12} between H60 and H72.

4. TABLE OF CONTENTS

1. TITLE PAGE 1

2. SIGNATURE PAGE 2

3. SYNOPSIS 4

4. TABLE OF CONTENTS 12

5. LIST OF ABBREVIATIONS AND DEFINITIONS OF TERMS 16

6. INTRODUCTION 18
6.1. GENERAL INFORMATION 18
6.2. MECHANISM OF ACTION 18
6.3. PHARMACEUTICS 18
6.4. PRE-CLINICAL STUDIES 19
6.5. CLINICAL DATA 22
6.6. DISEASE REVIEW AND RATIONALE FOR THE STUDY 24
6.7. RISK-BENEFIT ASSESSMENT 26

7. OBJECTIVES 28
7.1. PRIMARY OBJECTIVE 28
7.2. SECONDARY OBJECTIVES 28

8. INVESTIGATIONAL PLAN 29
8.1. SUMMARY OF STUDY DESIGN 29
8.2. DISCUSSION OF TRIAL DESIGN 32
8.3. TRIAL TIMETABLE (STUDY PERIODS) 32

9. SELECTION AND WITHDRAWAL OF SUBJECTS 33
9.1. INFORMED CONSENT 33
9.2. INCLUSION CRITERIA 33
9.3. EXCLUSION CRITERIA 34
9.4. ASSIGNMENT OF PATIENT NUMBER 35
9.5. PREMATURE WITHDRAWAL OF PATIENTS FROM STUDY AND REPLACEMENT POLICY 35
9.5.1. Discontinuation criteria 35

9.5.2. Replacement policy 36

10. STUDY MEDICATION 37

10.1. PRESENTATION, STORAGE, PACKAGING AND LABELLING 37

10.1.1. Presentation, Storage, Packaging and Labelling of Investigational Product 37

10.1.2. Presentation, Storage, Packaging and Labelling of Placebo 40

10.2. BLINDING 40

10.3. EMERGENCY UNBLINDING 40

10.4. DOSE, ROUTE AND SCHEDULE OF INVESTIGATIONAL PRODUCT ADMINISTRATION 41

10.5. DOSE JUSTIFICATION 41

10.6. CRITERIA FOR SCHEDULE ADJUSTMENT/DOSE-MODIFICATION OF INVESTIGATIONAL PRODUCT 41

10.7. INVESTIGATIONAL PRODUCT ACCOUNTABILITY 41

10.8. TREATMENT COMPLIANCE 42

11. CONCOMITANT THERAPY/MEDICAL MANAGEMENT OF ADVERSE EVENTS 43

11.1. MEDICATIONS THAT MAY NOT BE ADMINISTERED 43

11.2. MANDATORY CONCOMITANT THERAPY 43

12. SCHEDULE OF VISITS 44

12.1. PRE-STUDY EVALUATION 44

12.1.1. Study Baseline assessment (prior to randomization) 44

12.2. TREATMENT PERIOD 45

12.2.1. Schedule of visits for on-treatment period 45

12.2.2. Treatment administration/general procedures 45

12.2.3. Assessments to be performed during the treatment period 45

12.3. DAY 8 VISIT AND FINAL VISIT ON DAY 21 46

12.4. PREMATURE DISCONTINUATION 47

13. ASSESSMENTS OF EFFICACY 49

13.1. SPECIFICATION OF EFFICACY PARAMETERS 49

13.2. METHODS AND TIMING FOR ASSESSING AND RECORDING EFFICACY PARAMETERS 49

14. ASSESSMENTS OF SAFETY 51

14.1. SPECIFICATION OF SAFETY PARAMETERS 51

14.1.1. Clinical examination, body temperature and vital signs 51

14.1.2. Safety laboratory assessments 52

14.1.3. Electrocardiogram 52

14.1.4. Hepatic encephalopathy grade (HE grade) and Glasgow Coma Score (GCS) 53

14.1.5. Anti-ALF-5755 antibody plasma levels 53
14.2. ADVERSE EVENT REPORTING 54
14.2.1. Definition of an Adverse Event 54
14.2.2. Serious Adverse Events 55
14.2.3. Recording and Reporting of Adverse Events 55
14.2.4. Reporting Serious Adverse Events to EC 58
14.2.5. Adverse Event Reporting Period 59
14.3. PHARMACOKINETIC EVALUATION 59
14.4. DATA SAFETY MONITORING BOARD (DSMB) 60

15. EVALUATION OF RESULTS 61
15.1. SAMPLE SIZE AND STUDY POWER 61
15.2. STATISTICAL METHODS 62
15.2.1. General considerations 62
15.2.2. Populations for analysis 63
15.3. BASELINE CHARACTERISTICS 63
15.4. EVALUATION OF EFFICACY 63
15.4.1. Evaluation of primary efficacy endpoint 63
15.4.2. Evaluation of secondary efficacy endpoints 64
15.4.1 Evaluation of exploratory endpoint(s) 66
15.4.2 Sub-group evaluation of efficacy and exploratory endpoint*(s)* 66
15.5. OTHER EVALUATIONS 66
15.5.1. Pharmacokinetic evaluation 66
15.6. INTERIM ANALYSIS 66

16. OBLIGATIONS OF THE PRINCIPAL INVESTIGATOR 67
16.1. INDEPENDENT ETHICS COMMITTEE (IEC) 67
16.2. REGULATORY BODY APPROVAL 68
16.3. INFORMED CONSENT AND SCREENING DATA 68
16.4. CASE REPORT FORMS AND SOURCE DOCUMENT VERIFICATION 68
16.5. CONFIDENTIALITY 69
16.6. STAFF INFORMATION & RESPONSIBILITIES 69
16.7. DOCUMENTATION REQUIRED PRIOR TO INITIATION 69
16.8. DOCUMENTATION REQUIRED DURING THE STUDY 70
16.9. ESSENTIAL DOCUMENT RETENTION 70

17. STUDY MANAGEMENT 71
17.1. MONITORING 71
17.2. QUALITY ASSURANCE AND QUALITY CONTROL 71
17.2.1. Quality Control 71
17.2.2. Quality Assurance 71
17.2.3. Audit 71

17.3. DATA QUERY PROCESS 72
17.4. PROTOCOL DEVIATIONS/AMENDMENTS 72
17.5. DISCONTINUATION OF THE STUDY 72
17.6. PUBLICATIONS 72

18. STUDY TIMETABLE 73

19. REFERENCES 74

20. APPENDICES 76

LIST OF TABLES

Table 8-1: Dosage Schedule 29
Table 10-1: Composition of formulation 38
Table 14-1: Relationship of the Adverse Event to the Investigational Product 57
Table 14-2: Definition of Severity of Adverse Events 57
Table 15-1: PR evolution 61
Table 15-2: Results 62

LIST OF FIGURES

Figure 8-1: Study Design 30
Figure 10-1: Amino acid sequence of ALF-5755. 37

LIST OF APPENDIX

Appendix A: World Medical Association Declaration of Helsinki (2008 version) 77
Appendix B: ICH-GCP – Investigator’s responsibilities 80
Appendix C: Study Flow Chart 86
Appendix D: Pharmacokinetics Schedule 87
Appendix E: Grades of hepatic encephalopathy 88
Appendix F: Glasgow Coma Scale 89

5. LIST OF ABBREVIATIONS AND DEFINITIONS OF TERMS

ActD	Actinomycin D
AE	Adverse Event
AFSSAPS	Agence française de sécurité sanitaire des produits de santé
ALF	Acute Liver Failure
ALT	Alanine Transaminases
ANA	Antinuclear antibody
Anti-LKM	Antibodies to liver and kidney microsomes
ASMA	Anti-smooth muscle antibody
AST	Aspartate Transaminases
AUC	Area under the curve (exposure to drug)
BAL	Bioartificial liver
BP	Blood Pressure
BMI	Body Mass Index
C_{max}	Maximum observed plasma concentration
cGMP	Current Good Manufacturing Practice
CI	Confidence Interval
CK18	Cytokeratin 18
CR	Complete Response
CRA	Clinical Research Associate
CRF	Case Report Form
CS	Clinically Significant
CTC	Common Toxicity Criteria
CV	Curriculum Vitae
CVC	Central Venous Catheter
DLT	Dose Limiting Toxicity
DSMB	Data Safety Monitoring Board
EC	Ethics Committee
EC50	Median effective concentration
ECG	Electrocardiogram
ELAD	Extracorporeal Liver Assist Device
EU	European Union
FPI	First-Patient-In
FV	Factor V
GCP	Good Clinical Practice
GCS	Glasgow Coma Scores
GMP	Good Manufacturing Practice
GGT	Gamma glutamyltransferase
hCG	Human chorionic gonadotropin
HAV	Hepatitis A Virus
HBV	Hepatitis B Virus
HCV	Hepatitis C Virus
HDV	Hepatitis D Virus
HE	Hepatic Encephalopathy
HEV	Hepatitis E Virus
HIP/PAP	Hepatocarcinoma-intestine-pancreas/pancreatitis-associated protein
HIV	Human Immunodeficiency Virus
HR	Heart Rate
HSV	Herpes Simplex Virus
IC50	half maximal inhibitory concentration
ICH	International Conference on Harmonisation
IDM	Independent Drug Monitor

IEC	Independent Ethics Committee
IFN	Interferon
Ig	Immunoglobulin
IL	Interleukin
IMPD	Investigational Medicinal Product Dossier
INR	International Normalized Ratio
IP	Investigational Product
ITT	Intent-to-Treat
LPI	Last-Patient-In
LPLV	Last-Patient-Last-Visit
LPS	ipopolysaccharide
MAIO	monoamine oxidase inhibitors
NAC	N-acetyl cysteine
NCR	No carbon repeat
NOAEL	No observed adverse effect level
NOEL	No observed effect level
NSAID	Nonsteroidal anti-inflammatory drugs
PCR	Polymerase chain reaction
PD	Pharmacodynamic
PK	Pharmacokinetics
PKA	Protein Kinase A
PICD	Patient Informed Consent Document
PMA	Phorbol Myristate Acetate
PP	Per-Protocol
PR	Prothrombin Rate
PT	Prothrombin Time
QA	Quality Assurance
QC	Quality Control
RBC	Red Blood Cell
RR	Respiratory Rate
SAE	Serious Adverse Event
SAH	Severe Acute Hepatitis
SAP	Statistical Analysis Plan
SD	Standard Deviation
SIRS	Systemic Inflammatory Response Syndrome
SOC	System organ class
SOP	Standard Operating Procedure
$T_{1/2}$	Apparent elimination half-life
TEAE	Treatment-emergent adverse event
TNF	Tumour necrosis factor
t_{max}	Time to achieve maximum concentration
UK	United Kingdom
VZV	Varicella Zoster Virus
WBC	White Blood Cell
WHO	World Health Organization

6. INTRODUCTION

6.1. GENERAL INFORMATION

ALF-5755, rhHIP/PAP, has been developed to become a future therapeutic specialty for the treatment of patients suffering from severe acute hepatitis (SAH) and acute liver failure (ALF). ALF-5755 promotes both cell survival after apoptotic or oxidative stress and regeneration of hepatocytes in primary cultures and *in vivo*. ALF-5755 significantly increased survival in a Fas mediated acute liver failure model in mice.

PX7-F8-ALF-5755 current Good Manufacturing Processes (cGMP) batch was and will be used for phase I and this current phase II clinical trials.

6.2. MECHANISM OF ACTION

ALF-5755 is a 150 amino-acid recombinant protein derived from the human protein HIP/PAP (Hepatocarcinoma-Intestine-Pancreas / Pancreatitis Associated Protein). HIP/PAP belongs to the family of C-type lectins and acts as an adhesion molecule for hepatocytes.

ALF-5755 has been shown to share the same biological activities as the human protein HIP/PAP. HIP/PAP is a secreted protein developing its biological activities in a paracrine fashion. To date, no membrane-bound or cytoplasmic receptor for human HIP/PAP has been described. The specific mechanism of action of HIP/PAP remains unclear. It is only understood that at least a part of its mitogenic and anti-apoptotic properties functions through the Protein Kinase A (PKA) signaling pathway (Simon, Pauloin et al. 2003)

This protein is constitutively expressed by some specialized epithelial cell subsets in the digestive tract and the pancreas. It is over-expressed in hepatocellular carcinoma, acute pancreatitis, cystic fibrosis, and Crohn's disease. The plasma level of HIP/PAP is about 30 µg/L in healthy volunteers.

HIP/PAP is also a liver paracrine growth factor which favours liver regeneration after partial hepatectomy, and liver protection against acetaminophen- (paracetamol) and J0-2- (antibody recognizing the Fas/CD95 receptor) induced fulminant hepatitis in mice. HIP/PAP is able to activate early stages of hepatocyte regeneration, which was confirmed using a knockout mice model, as invalidation of the HIP/PAP gene sensitized mice to Fas intoxication and delayed liver regeneration.

6.3. PHARMACEUTICS

ALF-5755 is a recombinant protein produced by fermentation of an *E. coli* production strain preserved as a Master Cell Bank. It has the amino acid sequence of the human HIP/PAP mature protein. For the sake of expression in *E. coli* an additional methionine was added in first position.

ALF-5755 is presented as a sterile lyophilised powder in 20 mL vials. It should be reconstituted with 6.7 mL of water for injection to yield an injectable solution at 0.40 mg/mL.

Three successive GMP compliant batches were produced and an extensive analytical package was developed. ALF-5755 achieves a very high level of purity and displays the same biological activity as HIP/PAP.

6.4. PRE-CLINICAL STUDIES

Pre-clinical Pharmacology

ALF-5755 effects have been investigated in various *in vitro* and *in vivo* models to assess its anti-apoptotic, mitogenic and anti-oxidative properties.

Pharmacology

In vitro studies demonstrated the anti-apoptotic effects of ALF-5755 in primary rat hepatocytes pre-incubated with tumour necrosis factor-α/ actinomycin D (TNF_{α}/ActD) or H_2O_2. The minimal active concentration was 0.25 µg/mL and the half maximal inhibitory concentration (IC_{50}) 0.5 µg/mL. This effect was also observed in hepatocytes from dogs, monkeys and humans.
ALF-5755 was shown to be mitogenic in rat hepatocytes with an estimated median effective concentration (EC_{50}) between 0.5 and 1 µg/mL. This activity is thought to be of prime importance for liver regeneration in patients suffering from ALF. It was observed in hepatocytes from dog, monkey and human.

In vivo, ALF-5755 has been extensively studied in a mouse model of ALF and in the rat after partial hepatectomy.
The Fas agonist monoclonal antibody (J0-2), injected to C57BL6/J mice, a recognised model, induced a massive liver failure leading to cell death and ultimately to animal death. ALF-5755, when given intravenously at the dose of 750 µg/kg 6 or 9 hrs post intoxication, increased survival. Treatment of mice with ALF-5755 at the dose of 750 µg/kg at different time points resulted in an increase in liver cell proliferation. Treatment at 6 hrs with various doses of ALF-5755 (from 50 to 2500 µg/kg) showed a statistically significant effect from 250 µg/kg. Treatment with ALF-5755 (2500 µg/kg) decreased the level of active caspase 3 reflecting apoptosis inhibition.
The proliferative properties of ALF-5755 was confirmed in BDIX rats injected with rat colorectal carcinoma-derived cell lines to induce multiple hepatic metastases and subjected to a 70% hepatectomy to favour hepatocyte proliferation. A significant increase in non cancerous liver cell proliferation was observed 24 hrs after intravenous injection of ALF-5755 at 500 µg/kg. It is important to note that in this model, the size of hepatic metastatic nodules relative to the total liver area was not increased by the ALF-5755 treatment.

Secondary pharmacology

The bactericidal activity of HIP/PAP protein against Gram positive bacteria was confirmed with ALF-5755. The IC_{50} was estimated at 16.7 µg/mL.

Safety pharmacology

Safety pharmacology studies with ALF-5755 were performed by intravenous route in rats and dogs.

ALF-5755 did not modify neurobehavioral parameters and body temperature up to 3.4 mg/kg in the rat. The no observed effect level (NOEL) of ALF-5755 was equal to or greater than 3.4 mg/kg.
Hemodynamic parameters were assessed in telemetered conscious dogs up to 1.7 mg/kg. ALF-5755 did not induce any statistically significant change in arterial blood pressure, heart rate or conduction times. Very few animals displayed a transient decrease in arterial blood pressure (BP) during the infusion period with ALF-5755 at 0.34 mg/kg (2 animals) and 1.7 mg/kg (1 animal), associated in one dog with a transient decrease in heart rate. This hypotensive effect was not dose dependent and was not considered to be a direct pharmacological effect of ALF-5755. The NOEL of ALF-5755 was equal to or greater than 1.7 mg/kg.
ALF-5755 did not induce any statistically significant changes in respiratory parameters in conscious rats during a 4 hr period of measurement after dosing at 0.3, 1 and 3.4 mg/kg. The NOEL of ALF-5755 was equal to or greater than 3.4 mg/kg.

ADME and Pharmacokinetics in animals

The pharmacokinetics (PK) of ALF-5755 was evaluated in the mouse, rat and monkey following acute and repeated intravenous administrations by using a commercially available kit for human PAP.

In the mouse receiving an intravenous bolus of [3H]-ALF-5755, the PK profile of radioactivity was characterized by a rapid decrease in the plasma level (distribution half life: 10 minutes) and a prolonged terminal phase indicating a slow elimination (125 minutes). These preliminary data suggest a rapid tissue distribution that is preponderant during the first 30 minutes following the injection. Then, the excretion becomes the major clearance mechanism.

Dose linearity was shown for the area under the curve (AUC) in rats and monkeys within the repeated dose toxicity studies. No significant changes in the AUC/dose ratio were observed over 2 weeks in both animal species. There were no gender differences for rats and monkeys.
The accumulation factors in rats and monkeys (AUC day 14/AUC day 1) indicate that ALF-5755 had no tendency to accumulate in the body after successive intravenous administrations over 14 days with a 24 hr interval between doses.

The metabolism is expected to be similar to proteolytic degradation resulting in peptide and amino acid products.

Pre-Clinical Toxicology

A single dose toxicity study and a 7 day preliminary repeated dose study were conducted in Sprague Dawley rats to define the doses to be used in the repeated dose study. No death or abnormal clinical signs were observed at 3.4 mg/kg for 7 consecutive days by the intravenous route. Only a slight increase in body temperature was reported in female rats. No lesions were noticed.

The repeated dose toxicity studies performed in Sprague Dawley rats and Cynomolgus monkeys (14 days intravenous administration, 7 days follow-up) showed that ALF-5755 was well tolerated. The highest tested dose was the maximal administrable dose in both species. No target tissues were identified.

In rats receiving 0.3, 1 or 3.4 mg/kg/day, no drug-related mortality was seen. No relevant clinical signs, no effects on clinical chemistry and haematology were seen, whatever the dose administered. Blood was found in the urine of most of the animals in both vehicle and ALF-5755 treated groups. This effect was likely due to the high osmolarity of the vehicle. In the clinical batch the osmolarity level is lowered to that of physiological saline solution.There were no effects on organ weights and no histopathological findings in any tissue attributed to treatment with ALF-5755. Therefore, the no observed adverse effect level (NOAEL) was considered to be equal to or higher than 3.4 mg/kg.

ALF-5755 was well tolerated in monkeys receiving intravenous 0.2, 0.5, or 1.25 mg/kg/day. There were no death, no abnormal clinical signs, and no effects on body weight or changes on food consumption indicative of any adverse effect. Higher mean inorganic phosphorus levels were observed in male animals treated at 1.25 mg/kg. However, it is noticeable that some animals in other groups also displayed elevated concentrations before treatment (pre-test).
Greater spleen weights were seen in males receiving the highest dose. As no microscopic impact was observed, these findings were considered to be of minor toxicological significance. The NOAEL of ALF-5755 was considered to be equal to or higher than 1.25 mg/kg/day.

In both studies, the PK analysis showed that the ratio C_{max}/dose and $AUC_{(0-t)}$/dose remained nearly constant when the dose increased, suggesting that the exposure was dose-linear. As expected for a human protein administered to animals, antibodies against ALF-5755 were detected in some rats and monkeys by using a bridging immuno-assay method. The formation of antibodies in both species was not dose related. In the vast majority of the animals, the antibodies were not neutralizing. Moreover, they did not result in altered PK and were not associated with apparent adverse effects.

The formation of anti-ALF-5755 antibodies could be expected due to the repeated administration of a recombinant protein of human origin to rats and monkeys. Anti-ALF-5755 antibodies were not associated with any clinical signs, any haematological changes or any abnormal histological findings in lymphoid organs.

In dogs, during the safety pharmacology study on the cardiovascular system, a marked decrease in arterial BP in three animals followed by a transient decrease in heart rate, for one animal, was observed. It was not considered to be a direct pharmacological effect, but instead to reflect the expected immunogenicity of the human recombinant protein ALF-5755 in dogs.

Finally, the development of specific antibodies in rats, monkeys and dogs is not predictive of the potential immunogenicity of ALF-5755 in humans.

Based on the data from the two 14-day repeated dose toxicity studies, ALF-5755 can be considered to be devoid of any immuno-suppressive potential.

The effect of ALF-5755 on cytokine release was measured from peripheral blood mononuclear cells from four donors after a 72 hr exposure to four increasing concentrations of ALF-5755. A dose dependent increase in the production of TNFα, interleukin-6 (IL-6), IL-8 and IL-10 was observed at 5 and 10 µg/mL with a large inter donor variability. Interferon-γ (IFNγ) was increased at the two highest concentrations tested. For all cytokines, ALF-5755 response remained lower than those induced by the positive controls, ipopolysaccharide (LPS) and phorbol myristate acetate (PMA)/ionomycin combination.

Standard carcinogenicity studies are generally not required for biotechnology derived pharmaceuticals. However, due to the mitogenic properties of the recombinant protein ALF-5755 on normal hepatic cells, and in accordance with the Agence Française de sécurité sanitaire des produits de santé (AFSSAPS) scientific advice, specific *in vivo* and *in vitro* studies were conducted. ALF-5755 did not promote neither liver, pancreas, small intestine, colon nor brain cancer emergence in HIP/PAP over-expressing mice after 24 months. Nor did it promote the growth of hepatic metastases of colorectal cancer in rats. Furthermore, it did not promote the growth of three tumor cell lines – 2 from humans (one hepatocarcinoma and one hepatoblastoma) and one from rats (one hepatic metastases of colorectal cancer). No tumorigenic potential in human tumor cell lines or tumor initiation or growth has been observed in preclinical studies.

Conclusion

In conclusion, pre-clinical toxicology data showed that ALF-5755 is safe and well tolerated up to 3.4 and 1.25 mg/kg when administered intravenously as a 14 day repeated dose to rats or monkeys, respectively.

The toxicokinetic analysis showed dose proportionality at the dose levels of 0.3 to 3.4 mg/kg in rats and 0.2 to 1.25 mg/kg in monkeys. Using survival as a primary end point in the Fas mediated acute liver failure model in the mouse, or normal liver cell proliferation in the mouse Fas model and in hepatotectomized rats injected with DHDK12 cancer cells, a statistically significant effect was shown from 250 µg/kg in mice and at 500 µg/kg in rats. These dose levels are well below the NOAEL of 3.4 mg/kg in the single-dose and the repeated dose studies in rodents.

It is also important to note that the ALF-5755 C_{max} measured in the repeated dose toxicity study in monkeys at the highest tested dose level (18 and 19 µg/mL in male and female animals, respectively) was more than 6,000 higher than the endogenous PAP level values (2.96 and 2.78 ng/mL in male and female animals, respectively) measured before ALF-5755 dosing in the same animals.

Further detailed information can be found in the Investigator’s Brochure.

6.5. CLINICAL DATA

The Phase I First In Man study: ‘A double-blind, placebo-controlled, single intravenous ascending dose study to evaluate the safety, tolerability and PK of ALF-5755 in healthy male volunteers’ was conducted to evaluate the clinical and laboratory safety and tolerability profiles of ALF-5755 and to determine PK parameters of ALF-5755 given as a single intravenous dose to healthy male subjects.

The study was monocentric, double-blind, placebo-controlled, and randomised 3:1, using a single intravenous dose (slow intraveous infusion of 10 minutes using automatic syringes) with a sequence of 5 escalating doses: 0.105 mg, 1.05 mg, 3.99 mg, 10.05 mg and 19.95 mg. In Groups 1 and 2, 3 subjects received ALF-5755 and 1 subject received placebo (physiological serum); in Groups 3, 4 and 5, 6 subjects received ALF-5755 and 2 subjects received placebo.

Safety results

The administration of a single intravenous dose of ALF-5755 was safe and well tolerated at the doses of 0.105 mg, 1.05 mg, 3.99 mg and 10.05 mg, and the safety was considered as satisfactory at the highest tested dose of 19.95 mg.

There were no serious adverse events (SAEs) and no adverse events (AEs) that required the withdrawal of a subject.

A total of 8 treatment-emergent AEs (TEAEs) were reported in 7 subjects:

- 2 TEAEs in 2 subjects receiving placebo,
- 0 TEAE in subjects receiving 0.105 mg of ALF-5755,
- 0 TEAE in subjects receiving 1.05 mg of ALF-5755,
- 1 TEAE in 1 subject receiving 3.99 mg of ALF-5755,
- 1 TEAE in 1 subject receiving 10.08 mg of ALF-5755,
- 4 TEAEs in 3 subjects receiving 19.95 mg of ALF-5755.

The most frequently affected system organ class (SOC) after receiving ALF-5755 related to gastrointestinal disorders, reported in 2 active subjects (abdominal pain in 1 subject receiving 10.08 mg of ALF-5755 and 1 subject receiving 19.95 mg of ALF-5755). Other affected SOCs related to general disorders and administration site conditions, reported in 1 active subject (feeling abnormal in 1 subject receiving 19.95 mg of ALF-5755) and to musculoskeletal and connective tissue disorders, reported in 1 active subject (myalgia in 1 subject receiving 3.99 mg of ALF-5755). Nervous system disorders SOC was affected in 2 subjects receiving placebo (headache and paraesthesia). The respiratory, thoracic and mediastinal disorders SOC (cough and bronchospam) was affected only in an atopic subject #503 improperly included in the group receiving 19.95 mg of ALF-5755. This type of reaction is frequent on an atopic field, whatever the administered drug. It consisted of 2 TEAEs which were the only TEAEs of moderate intensity and the subject recovering rapidly after receiving corrective treatment, whereas all other TEAEs were mild in intensity with subjects recovering spontaneously. All TEAEs were considered related to study drug (possibly or probably, each in 4 subjects).

There was no relationship between the administered dose and the number or intensity of TEAEs. The TEAEs which have been considered related to the tested drug could be expected in healthy subjects dosed according to the design of the study.

There was no clinically significant out-of-range value in haematology, blood chemistry or urinalysis parameters, no clinically significant out-of-range value in vital signs or electrocardiogram (ECG) parameters.

No antibodies against ALF-5755 were detected and local tolerance tests were and remained negative throughout the study.

Pharmacokinetic results

Following a single 0.105 mg intravenous dose of ALF-5755, plasma concentrations reflected only endogenous HIP/PAP protein.

Following single intravenous injections of ascending doses of 1.05, 3.99, 10.08 and 19.95 mg of ALF-5755 in healthy male volunteers, maximum ALF-5755 plasma concentrations were observed at the end of the infusion. The exposure measured by the AUC and the maximal

plasma concentrations increased in a dose proportional manner. The inter-individual variability was low.

The terminal half-life was close to 4 hours following doses of 1.05 to 19.95 mg of ALF-5755.

Clearance and volume of distribution were consistent over the dose range of 1.05 to 19.95 mg, with mean values close to 4 L/hour and 25 L, respectively.

Conclusion

The administration of a single intravenous dose of ALF-5755 over 10 minutes to healthy male subjects was safe and well tolerated at the doses of 0.105 mg, 1.05 mg, 3.99 mg and 10.05 mg. The safety of ALF-5755 was considered as satisfactory at the highest tested dose of 19.95 mg.

Following intravenous injection of a single 0.105 mg dose of ALF-5755 over 10 minutes to healthy male subjects, plasma concentrations reflected only endogenous HIP/PAP protein. Following single intravenous injections of ascending doses of 1.05 to 19.95 mg of ALF-5755, the maximum ALF-5755 plasma concentrations were observed at the end of infusion. The exposure measured by the AUC and the maximal plasma concentrations increased in a dose proportional manner. The inter-individual variability was low. The terminal half-life was close to 4 hours following doses ranging from 1.05 to 19.95 mg of ALF-5755. Clearance and volume of distribution were consistent over the dose range studied, with mean values close to 4 L/hour and 25 L, respectively.

6.6. DISEASE REVIEW AND RATIONALE FOR THE STUDY

ALF is a rare but dramatic disease of multiple etiologies, often affecting young subjects, with a median age of diagnosis of 38 years. Women are more affected than men (Cox 2009). ALF is marked by the sudden loss of hepatic function in a person without preexisting liver disease (Lee, Squires et al. 2008), and leads to a rapid deterioration of liver functions with the presence of coagulopathy and hepatic encephalopathy.

ALF is a life-threatening disease of multiples etiologies with a high rate of mortality in absence of liver transplantation, except for acetaminophen-induced acute liver failure for which N-acetyl cysteine is a registered antidote.

All patients with clinical or laboratory evidence of moderate to severe acute hepatitis should have immediate measurement of prothrombin time (PT) and careful evaluation for subtle alterations in mentation (Polson and Lee 1995).

The etiologies of acute liver failure are, depending on geographic locations:

- acetaminophen overdose: 20% in France (Samuel 2008), about 50% in the USA, and close to 60% in the UK and in Denmark (Lee, Squires et al 2008), almost none in Asia.
- viral hepatitis: 30% in France (Samuel 2008), and 11% in USA (Lee 2008), and leading etiology in Asia and Africa. Most frequent viruses are hepatitis B virus (HBV), 56%, and hepatitis A virus (HAV), 25%, in France as in Japan, and other viruses (herpes simplex virus [HSV] or hepatitis E viruses [HEV]) (Samuel 2008).
- drug-induced toxicity: 10% in France (Samuel 2008) and 11% in the USA (Lee 2008), mostly due to isoniazid, monoamine oxidase inhibitors (MAOIs), nonsteroidal anti-inflammatory drugs (NSAIDs), halothane, ecstasy, phenytoin.

- autoimmunity: 5% in the USA (Lee, Squires et al. 2008).
- others: 15% in France and 18% in the USA (Lee 2008). Etiologies are metabolic, cardiovascular, miscellaneous origins (Wilson's disease, ischemia, acute fatty liver pregnancy, amanita phalloid, herbal medicines)
- indeterminate: 14% in the USA, (Lee 2008), 17% in France (Samuel 2008).

ALF-5755, rhHIP/PAP, a C-type lectin, has been shown to promote cell survival after apoptotic or oxidative stress, and hepatocytes' regeneration in primary cultures and *in vivo*. ALF-5755 significantly increases survival in the Fas mediated ALF model in mice.

In the First In Man study on healthy volunteers treated with ALF-5755, the safety and PK of the following single doses of ALF-5755 were investigated: 0.1mg, 1 mg, 4 mg, 10 mg and 20 mg, administered as a slow intravenous infusion over 10 minutes. The study showed that ALF-5755 had a satisfactory tolerance in healthy volunteers up to the maximal tested dose of 20 mg, and the clearance and volume of distribution of ALF-5755 was consistent over the dose range studied, with mean values close to 4 L/hour and 25 L, respectively, and low inter-individual variability.

In this study, patients included will be affected by nonacetaminophen SAH and early stage ALF, and will be treated with 10 mg of ALF-5755 or placebo through an intravenous slow infusion over 10 minutes every 12 hours over 3 days.

Patients with a diagnosis of nonacetaminophen SAH and early phase ALF have been selected as no treatment is currently available outside symptomatic treatment. Placebo, physiological saline solution, has been chosen as the comparator. Patients with SAH and early phase ALF due to acetaminophen overdose were excluded as N-acetyl cysteine is a registered antidote.

Patients having an early phase ALF with grade I or II hepatic encephalopathy can be included as well as patients with SAH to evaluate the efficacy of a full course of 3-day treatment with ALF-5755 in preventing worsening of ALF or aggravation of SAH into ALF, which has a much poorer survival prognosis whatever its stage. Patients presenting an ALF with grade III or IV hepatic encephalopathy have been excluded as these patients are at high risk of early death or early liver transplantation before 72 hours after diagnosis.

The dose regimen of ALF-5755 will be 10 mg administered every 12 hours over 3 days. The dose of 20 mg daily was chosen as it is above the anticipated therapeutic dose of 4 mg and had a satisfactory tolerance when given in one infusion of 20 mg in healthy volunteers. Given in split dose of 10 mg twice a day, it enables better coverage of the 24-hour period as ALF-5755 was shown to have a short half-life of 4 hours in the first in man study. Accumulation is not anticipated as there was no accumulation in two species of animal PK models after a daily infusion of ALF-5755 over 14 days, and the NOAEL was above maximum tested dose in all species. ALF-5755 PK characteristics have shown to be similar amongst species including humans with low inter-individual variability.

The 3-day treatment period was chosen as most experimental or registered therapies as N-acetyl cysteine or medical device under clinical evaluation in ALF. It is also the time delay where physicians are assessing the prognosis of spontaneous recovery in patients having ALF, and deciding whether or not to list the patient on the registry for liver transplantation. This time period also covers the disease period where ALF-5755 efficacy will be shown, as the

efficacy of ALF-5755 has been demonstrated at 6 hours and above after induction of ALF in the Fas mice model.

The route and mode of administration, a slow intravenous infusion over 10 minutes, have been kept as in the First In Man study.

In conclusion, ALF-5755 may become, in this life-threatening dramatic disease with high unmet medical need, a future therapy for the treatment of patients suffering from SAH and ALF not due to acetaminophen, where liver transplantation is the sole treatment in the absence of spontaneous recovery.

6.7. RISK-BENEFIT ASSESSMENT

ALF is a rare and life-threatening disease with high rate mortality in the absence of spontaneous recovery or liver transplantation. Its outcome is related to the etiology, the degree of encephalopathy, and related complications. Unfortunately, despite aggressive treatment, many patients die from fulminant hepatic failure (Hoofnagle JH et al. 1995; Lee WM et al. 1999).

It is recognized that survival of patients suffering from ALF depends on the speed with which quiescent liver cells re-enter the cell cycle and proliferate to compensate for the functional loss of liver cells (Kondo, Suda et al. 1997). It is also known that the more the liver cells are preserved during the toxic phase the more likely it is that recovery is possible (Michalopoulos and DeFrances 1997).

Therefore, there are two approaches which are not mutually exclusive in terms of new drug-based therapies:

- To promote liver cell survival during the toxic phase,
- To increase/favour liver regeneration.

ALF-5755 exhibits, similarly to HIP/PAP (Lieu, Batteux et al. 2005; Lieu, Simon et al. 2006), anti-apoptotic, mitogenic and anti- oxidant properties both *in vitro* in hepatocytes and *in vivo* in a mouse model of lethal hepatic intoxication. In addition, HIP/PAP has been reported to have anti-inflammatory (Iovanna and Dagorn 2005), and bactericidal properties, which have also been shown with ALF-5755.

Therefore, ALF-5755 displays the biological properties expected for its efficacy in the treatment of ALF, since it exhibits anti-apoptotic and anti-oxidant activities, and stimulates primary hepatocyte proliferation. These pharmacodynamic (PD) properties make ALF-5755 suitable and of interest for its clinical development in the therapeutic indication of ALF.

Taking into consideration the findings in the Phase I study, no circulating antibodies against ALF-5755 have been detected in healthy volunteers after a single dose administration of ALF-5755. However, immunogenicity will be closely monitored in all patients included in the Phase II study. Patients will be closely monitored during and one hour after every infusion to treat potential immunoallergic side-effects, and will be informed of this potential risk prior to study inclusion. Every recruiting centre will have a resuscitation trolley, adequate equipment and trained medical staff available to treat potential emergency events.

Effects on embryonic development must be assumed and should be avoided by stipulating effective methods of contraception (as described in the exclusion). An effective method of

contraception will be prescribed by the investigator to every woman with childbearing potential.

In this study all subjects will receive the best available care for SAH and early stage ALF. ALF-5755 or placebo will be administered in a randomized, blinded manner, in addition to standard care. Since ALF-5755 is not expected to affect the underlying condition, use of best treatment is ethically mandated and randomization should balance any impact of specific treatments.

Safety parameters as outlined in the study protocol will be intensively monitored. All toxicologically remarkable clinical laboratory parameters, in particular hepatic biology parameters, are covered appropriately as well as regular clinical examinations. In addition, a Data Safety Monitoring Board (DSMB) will be set up to periodically evaluate the overall safety profile and will review the adverse reactions experienced by each individual patient.

In conclusion, the overall risk involved in the administration of ALF-5755 as a potential treatment for patients suffering from life-threatening SAH and early stage ALF without other established therapeutic alternatives outside liver transplantation is considered to be acceptable, and at the benefit of the patients.

7. OBJECTIVES

7.1. PRIMARY OBJECTIVE

- To evaluate the efficacy of ALF-5755 versus placebo, measured by rate of change of prothrombin ratio (PR) during the 72 hours following treatment initiation, in patients with nonacetaminophen SAH and early stage ALF.

7.2. SECONDARY OBJECTIVES

- To evaluate the safety and tolerability of ALF-5755 versus placebo in patients with nonacetaminophen SAH and early stage ALF.
- To determine the PK parameters of ALF-5755 versus placebo in patients with nonacetaminophen SAH and early stage ALF.

8. INVESTIGATIONAL PLAN

8.1. SUMMARY OF STUDY DESIGN

This is a prospective, multicentre, double-blind, placebo-controlled study of ALF-5755 in patients with nonacetaminophen SAH and early stage ALF, randomized in the ratio 1:1. A minimum of 60 patients will be recruited into the study in the following two treatment groups:

- Group A: approximately 30 patients will receive ALF-5755
- Group B: approximately 30 patients will receive placebo (physiological saline solution: 0.9% NaCl)

Patients will receive study treatment (either placebo or ALF-5755) every 12 hours over 3 days according to the dosage schedule in Table 8-1. The time between biological baseline assessment and first injection of ALF-5755 or placebo should be as short as possible, but should not exceed 12 hours.

Patients should continue to receive (in addition to ALF-5755 or placebo) the best standard treatment on assessment of investigators.

Table 8-1: Dosage Schedule

Group A: ALF-5755	Group B: Placebo
10 mg (25 ml)<br><br>given in slow intravenous infusion over 10 minutes with an automatic syringe at:<br>• H0<br>• H12<br>• H24<br>• H36<br>• H48<br>• H60	25 ml of physiological saline solution (0.9% NaCl)<br><br>given in slow intravenous infusion over 10 minutes with an automatic syringe at:<br>• H0<br>• H12<br>• H24<br>• H36<br>• H48<br>• H60

The study design is summarised in Figure 8-1.

Figure 8-1: Study Design

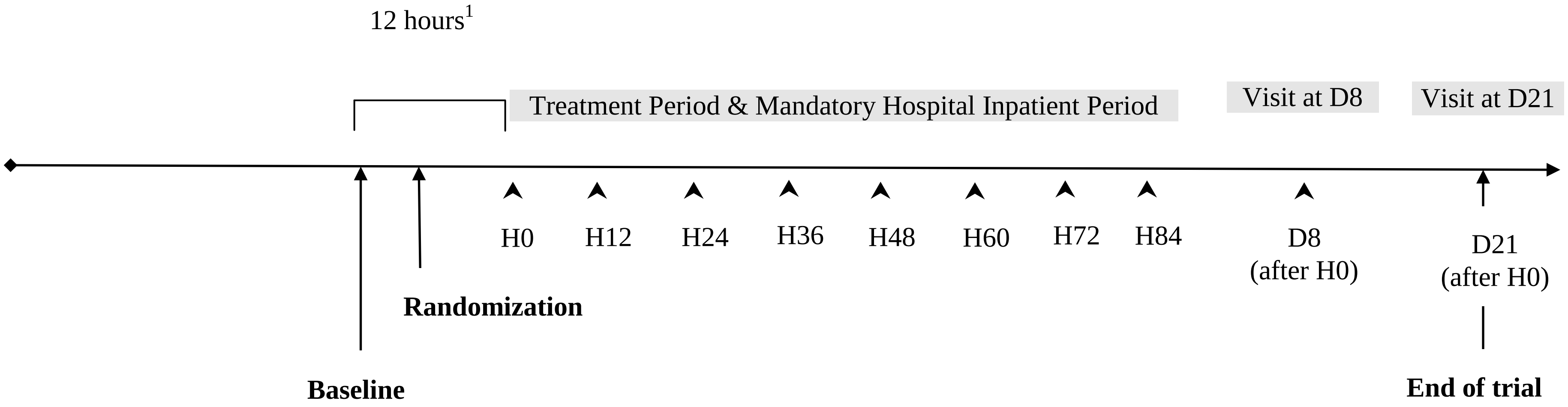

[1]1st infusion within 12 hours after biological baseline assessment

The primary endpoint of the study is:

- Rate of change of PR.

The secondary efficacy endpoints of the study are:

- Rate of change of Factor V (FV) plasma level,
- Rate of change of international normalized ratio (INR),
- Rate of change of alanine transaminases [ALT] plasma level,
- Rate of change of aspartate transaminases (AST) plasma level,
- Change of Hepatic Encephalopathy Grade (HE grade) during 72 hours following treatment initiation,
- Overall survival rate at D21,
- Transplant-free survival rate at D21,
- Liver transplant rate at D21,
- Length of hospitalisation (days).

The secondary safety endpoint of the study is:

- To determine the safety and tolerability of ALF-5755.

The secondary PK endpoint of the study is:

- Plasma concentrations of ALF-5755 will be measured as described in the PK Schedule (Appendix D), in the first 16 patients included at Paul Brousse Hospital and Beaujon Hospital (8 patients per centre).

The exploratory endpoint of the study is:

- Total and cleaved cytokeratin 18 plasma levels (CK18).

A schedule for the tests and evaluations to be conducted in this study is found in the flow chart in Appendix C.

Study Day 0 (D0) can be defined as the first day of treatment with either ALF-5755 or placebo. All days thereafter are defined as “Dn” (e.g. D1, D2, D3 etc). However, prior to D8, all timepoints are described as the hour (H) after the first infusion, where the first infusion takes place at H0.

A time window of ± 10 minutes is permitted for each timepoint from H0 to H84, except for PK measures where a time window of ± 2 minutes is permitted only for post-infusion dosing, and a time window of + 2 days is permitted for D8 and D21.

The expected duration of the trial for each patient is 21 days.

The baseline assessment visit to obtain informed consent **must** be conducted prior to randomization and prior to the commencement of any study activities requiring informed consent.

8.2. DISCUSSION OF TRIAL DESIGN

A double-blind, randomized, placebo-controlled study design has been chosen for this study to eliminate bias and to promote objectivity (refer to Sections 9.4 and 10.2 for further discussions of the randomization and blinding procedures).

8.3. TRIAL TIMETABLE (STUDY PERIODS)

The trial includes the following visits:

- Baseline assessment prior to randomization. (The time between baseline assessment of biological data and H0 should be the shortest as possible and should not exceed 12 hours)
- Treatment period under permanent medical and nursing supervision from H0 (1st injection) to H60 (last injection) (one infusion every 12 hours, e.g. H0, H12, H24, H36, H48, H60)
- A mandatory hospital inpatient follow-up period of 24 hours after the last infusion at H60, up to H84
- A visit on D8 on an inpatient or outpatient basis
- The End of Study visit will be on D21 on an inpatient or outpatient basis.

9. SELECTION AND WITHDRAWAL OF SUBJECTS

A minimum of 60 patients (approximately 30 patients per group) will be included in the study. Each patient must:

- meet **all** of the inclusion and exclusion criteria specified below (see Sections 9.2 and 9.3) within the specified time-frame,
- receive the allotted course of treatment (see Sections 10.4 and 9.5.2) and complete the required activities specified in this protocol,
- have his/her case report form (CRF) completed, received and accepted by ORION Clinical Services.

9.1. INFORMED CONSENT

Each potentially eligible patient will be informed of the study's objectives and overall requirements. Prior to conducting any of the pre-entry tests not performed routinely in the treatment of the patient, the Investigator will explain the study fully to the patient or his/her guardian (next of kin) using the Patient Informed Consent Documents (PICD). If the patient's cognitive function is impaired in a way that prevent obtaining of informed consent, his/her next of kin will be asked to provide consent. The patient will only provide consent if his/her status allows for this to be done.

If the patient is willing to participate in the study or, in the instance where required[1], his/her guardian(s) (next of kin) is willing for the patient to participate in the study, (s)he will be requested to give written informed consent. The Informed Consent will be signed and personally dated by **both** the patient or the guardian(s) and the Investigator. A copy of the signed form will be provided to the patient/guardian(s) and the original retained with the source documents. Although nursing staff may be involved in describing the study to a patient or his/her guardian(s), the Investigator must participate in discussions with the patient or his/her guardian(s) **and sign** and personally date the Informed Consent.

9.2. INCLUSION CRITERIA

To be eligible for inclusion into this study, each patient must fulfil **all inclusion criteria** prior to randomization:

1. A signed written informed consent from patient or from patient's next of kin
2. Early stage acute liver failure OR severe acute hepatitis defined as:
    - $15\% \leq PR < 50\%$
    - No hepatic encephalopathy, OR grade I or II encephalopathy (Appendix E)
    - Presumed acute illness onset of less than 26 weeks

[1] If the patient is capable, he/she will be required to sign and personally date the Informed Consent. However, the patient's guardian (next of kin) will be responsible to sign the PICD if the patient is not capable to do so.

- No evidence of underlying chronic liver disease

3. Patient who can receive first treatment dose within the first 12 hours after biological baseline assessment
4. Age ≥ 18 and ≤ 65 years
5. Contraception (only for females of childbearing potential) to be taken throughout the study until D21. Sole mechanic contraceptives, such as condoms, are advised. Note: Oral contraceptives may have contraindications in case of severe acute hepatitis and acute liver failure
6. Patient affiliated to social security insurance system.

9.3. EXCLUSION CRITERIA

To be eligible for inclusion into this study, each patient must violate none of the following exclusion criteria prior to randomisation:

1. Acetaminophen-induced hepatitis defined as acetaminophen intake > 4 g/day, at least once in the 7 days prior to baseline
2. Shock liver (ischemic hepatopathy) OR HELLP syndrome OR Budd-Chiari syndrome OR intrahepatic malignancy
3. Serum creatinine ≥ 180 µmol/L
4. Body Mass Index (BMI) ≥ 35
5. Sepsis defined as systemic response to proven or suspected infection manifested by two or more of the Systemic Inflammatory Response Syndrome (SIRS) criteria (i.e. Temperature > 38°C or < 36°C; Heart rate > 90 beats/min; Respiratory rate > 20 breaths/min or $PaCO_2$ < 32 mgHg; white blood cell (WBC) count > 12.000 cells/mm^3, < 4.000 cells/mm^3, or > 10% immature (band) forms) at baseline
6. Uncontrolled active bleeding
7. Patients who received fresh frozen plasma, PPSB (Prothrombine-Proconvertine-Stuart-B), or vitamin K infusion over the last 48 hours
8. Patient receiving liver support device treatment, including but not exclusively bioartificial liver (BAL), Extracorporeal Liver Assist Device (ELAD), transgenic pig perfusion
9. Patient receiving hemodialysis, hemofiltration or hemodiafiltration treatment
10. Intractable arterial hypotension (arterial systolic BP equal to or below 70 mmHg) present or require inotropic drugs at baseline
11. Human Immunodeficiency Virus (HIV) positive patient

12. Active cancer
13. Pregnancy or breast-feeding
14. Surgery within 4 weeks prior to baseline, or unsolved surgical disease outside liver transplantation
15. Patient included in another clinical trial within 4 weeks prior to baseline
16. Patient with organ or bone-marrow allograft
17. Absolute contra-indication to liver transplantation.

9.4. ASSIGNMENT OF PATIENT NUMBER

Patients will be randomly assigned to either or Group A (ALF-5755) or Group B (Placebo) in the randomization ratio 1:1.

Randomisation will be stratified by centre. The site pharmacist (or designee) will be unblinded as the pharmacist (or designee) has to reconstitute ALF-5755 vials as well as the syringes for intravenous infusion containing 25 ml of placebo or ALF-5755. No other site personnel will be unblinded. The site pharmacist (or designee) will be provided with envelopes containing treatment allocation. More envelopes than the expected number of patients will be provided to make provision for extra recruitment following patient discontinuation.

9.5. PREMATURE WITHDRAWAL OF PATIENTS FROM STUDY AND REPLACEMENT POLICY

9.5.1. Discontinuation criteria

Patients will be informed that they have the right to withdraw from the study at any time, without prejudice to their medical care, and are not obliged to state their reasons. Any withdrawals must be fully documented in the CRF and should be followed up by the Investigator.

Additionally, the Investigator may withdraw a patient at any time if he/she considers this to be in the patient's best interest.

The patient **must** be discontinued from the study for the following reasons:

- Non-compliance with treatment schedule (e.g., discontinuation of study treatment in the case of toxicity).

Additionally, patients may be discontinued for any of the following reasons:

- SAEs
- Death, serious intercurrent illness or significant worsening of intercurrent illness
- If the patient drops out of the study
- If the patient is lost-to-follow-up

- At their own request or at the request of their legally accepted representative
- Protocol violations, including non-compliance with study procedures
- If, in the investigator's opinion, continuation in the study would be detrimental to the well-being of the patient
- At the specific request of the sponsor.

If a patient fails to return for a scheduled visit/follow up, attempts should be made to contact the patient to ensure that the reason for not returning is not an AE. Likewise if a patient declares his/her wish to discontinue from the study e.g. for personal reasons, an attempt should be made to establish that the true reason is not an AE (bearing in mind the patient is not obliged to state his/her reasons).

If the Investigational Product (IP) therapy is prematurely discontinued, the primary reason for discontinuation must be recorded in the appropriate section of the CRF and all efforts will be made to complete and report the observations as thoroughly as possible, including extra sampling measurements. A complete final evaluation following the patient's withdrawal should be made, as described in Section 12.4, and any AEs followed up until resolution or a period of 21 days from the last dose of the IP has elapsed, whichever is the longer.

The study will be terminated if, in the opinion of the investigator and the sponsor, significant safety concerns arise during the conduct of the study.

9.5.2. Replacement policy

Recruitment will continue until 60 patients are evaluable, i.e. have no major deviation impacting the evaluability of the primary endpoint and have PR assessments allowing rate of change of PR calculation as described in Section 13.2.

10. STUDY MEDICATION

10.1. PRESENTATION, STORAGE, PACKAGING AND LABELLING

10.1.1. Presentation, Storage, Packaging and Labelling of Investigational Product

10.1.1.1. Presentation of Investigational Product ALF-5755

Nomenclature

ALF-5755 is a new biological entity. It is an *Escherichia coli* made recombinant protein with the amino acid sequence of the human protein HIP/PAP, (Lasserre, Christa et al. 1992; Christa, Felin et al. 1994; Lieu, Batteux et al. 2005; Lieu, Simon et al 2006).

ALF-5755 has not yet been attributed with a non proprietary name. ALF-5755 is the Alfact Innovation company code name of the *E. coli* made recombinant human HIP/PAP.

Structure

ALF-5755 has the sequence of the human HIP/PAP protein, whose accession number is NP_620355 (PAP precursor) in the Entrez Protein sequence database. For the sake of expression in E. coli, the first 26 amino acids corresponding to the signal sequence have been removed, and an additional methionine was added in position 1.

Figure 10-1: Amino acid sequence of ALF-5755.

```
Position    1          11         21         31         41
ALF-5755    MEEPQRELPS ARIRCPKGSK AYGSHCYALF LSPKSWTDAD LACQKRPSGN

Position    51          61        71         81         91
ALF-5755    LVSVLSGAEG SFVSSLVKSI GNSYSYVWIG LHDPTQGTEP NGEGWEWSSS

Position    101        111        121        131        141
ALF-5755    DVMNYFAWER NPSTISSPGH CASLSRSTAF LRWKDYNCNV RLPYVCKFTD
```

Cysteines having the same colour (#15 + #26, #43 + #143 and #121 + #146) have been shown to form intrachain bonds.

The IP, ALF-5755, will be supplied lyophilisated in a glass vial with halobutyl stopper and aluminium seal. The vial is stoppered under nitrogen with partial vacuum. Each vial contains 2.80 mg of ALF-5755. Reconstitution in the vial with 6.7 ml of water for injection gives a clear colourless solution at 0.40 mg/ml of ALF-5755. Some moderate foaming may occur, but the foam readily disappears if the solution is left to stand at room temperature for a maximum of 90 minutes. Needles and syringes will be supplied for use during the dilution procedure.

Complete dilution instructions will be provided as a separate Standard Operating Procedure (SOP).

A detailed description of ALF-5755 can be found in the Investigational Medicinal Product Dossier (IMPD).

The IP, ALF-5755, will be supplied in glass vials. A total of 4 vials are required for each 10-minute infusion. The composition of the formulation included in each vial is provided in Table 10-1 below.

Table 10-1: Composition of formulation

**Component**	**Quantity per vial**
ALF-5755	2.80 mg
Tris HCl pH8	10 mM
NaCl	50 mM
Sucrose	200 mM
Water for injection *qsp*	7 mL

10.1.1.2. Storage/Stability of ALF-5755

Before reconstitution, the IP, ALF-5755, will be stored -20 ± 5 °C unless otherwise specified by Alfact Innovation. Thanks to its lyophilisated nature, the product can be left for up to 24 hours at room temperature. Upon reconstitution and transfer into the provided syringe, the product can be stored for up to 24 hours at +4 ± 3 °C, if not infused within 2 hours after reconstitution. All IPs must be stored in a secure location, and may be dispensed only by the Investigator or by a member of staff specifically authorised by the Investigator, or by a pharmacist, as appropriate. Any deviations from the recommended storage conditions should be immediately reported to ORION and the use of drug interrupted until authorisation for its continued use has been given by Alfact Innovation.

10.1.1.3. Packaging/Labelling of ALF-5755

Bulk Product

The IP, ALF-5755, will be supplied in cardboard or shock proof foam boxes containing the quantity of vials forecast to be required for each clinical centre.

Labelling will be prepared to meet the local regulatory requirements.

The boxes will harbour a label with study number, subject number, IP details including number of vial, batch number, strength, retest date, brief instruction for use and storage, the phrase “for clinical trial use only” and the details of the sponsor.

Each vial will be labelled with study number, investigator name, subject number, IP details including name of the IP with the phrase for intravenous injection/infusion, batch number and strength, and name of sponsor.

Subject number will be completed according to randomization number, by the pharmacist (or designee) performing the randomization.

Example of Label for Outer Packaging

Study No:
Investigator:
Subject Number:
Batch: PX7-F8-ALF-5755
4 vials of 2.80 mg ALF-5755 lyophilised powder for intravenous injection/infusion
Reconstitute in water for injection prior to administration
For clinical trial use only
Store at -20°C ± 5°C
Retest Date: dd/mm/yyyy
Alfact Innovation - 320 rue Saint Honore - F-75001 Paris
phone: + 33 1 45 59 35 66

Example of Label for Vial

Study No.:
Subject No.:
2.80 mg ALF-5755 for intravenous injection/infusion
Batch No.: PX7-F8-ALF-5755
Alfact Innovation

The water for injection and physiological saline solution vials will be provided by an independent person in charge of study drug reconstitution at each centre.

The syringes and infusion lines will be provided by ALFACT INNOVATION & will be used by the pharmacy (or the designee) of the investigating centre in compliance with the protocol described in the Investigator's Brochure:

- Syringe OMNIFIX® B/BRAUN 50 mL Lock Centre reference 4617509F,
- Infusion lines.

Individual Syringes

Individual syringes will be prepared by the pharmacy (or designee) of the investigating centre in compliance with the protocol described in the Investigator's Brochure, bearing the following information on the labels:

- Phrase "Use as directed by Study No: ALF-5755_P2_ALF"
- Subject name
- ALF-5755 2.80 mg or placebo, intravenous administration only
- Storage conditions
- Phrase "For clinical research only"
- Phrase "single use only"
- Alfact Innovation
- Phrase "Use within 24 hours after reconstitution and storage at +4°C ± 3°C"
- Reconstitution time

10.1.2. Presentation, Storage, Packaging and Labelling of Placebo

Physiological saline solution will be used as a placebo. The storage of placebo, as well as the packaging/labelling of individual syringes will be similar to that of the IP (refer to Sections 10.1.1.2 and 10.1.1.3 for detailed information).

10.2. BLINDING

The pharmacist or designee will be the only personnel to have access to the randomization envelopes in order to prepare the drug for administration.

10.3. EMERGENCY UNBLINDING

In case of an emergency, when knowledge of the IP assignment is required for the medical management of an individual subject, the treatment for that subject may be unblinded. A set of emergency unblinding envelopes will be stored on site and at ORION Clinical Services. The Investigator must notify the Sponsor within 24 hours after determining that it is necessary to unblind the treatment assignment.

This documentation must include the name of the individual breaking the blind, the date on which the blind was broken, and a description of the event that led to the unblinding. The Investigator must also indicate in source documents and in the CRF that the blind was broken and provide the date, time, and reason for breaking the blind. Any AE or SAE associated with breaking the blind must be recorded and reported as specified in this protocol.

The Independent Drug Monitor (IDM) will routinely check the integrity of the envelopes that are stored at the site. The envelopes will be collected from the site prior to study close-out and sent to the Sponsor to ensure that they were all intact.

10.4. DOSE, ROUTE AND SCHEDULE OF INVESTIGATIONAL PRODUCT ADMINISTRATION

The IP, ALF-5755, and placebo will be administered as slow intravenous infusions, each a 10-minute infusion of 10 mg (25 mL) using automatic syringes every 12 hours from H0 until H60 (that is, at H0, H12, H24, H36, H48 and H60, all ± 10 minutes).

Immediately, after the end of the intravenous administration, the infusion lines will be washed with 3 to 5 mL of physiological saline solution.

10.5. DOSE JUSTIFICATION

The dose regimen of ALF-5755 will be 10 mg administered every 12 hours over 3 days.

The dose of 20 mg daily was chosen as it is above the anticipated therapeutic dose of 4 mg and had a satisfactory tolerance when given in one infusion of 20 mg in healthy volunteers. Given in split dose of 10 mg twice a day, it enables better coverage of the 24-hour period as ALF-5755 was shown to have a short half-life of 4 hours in the first in man study. Accumulation is not anticipated as there was no accumulation in two species of animal PK models after a daily infusion of ALF-5755 over 14 days, and the NOAEL was above maximum tested dose in all species. ALF-5755 PK characteristics have shown to be similar amongst species including humans with low inter-individual variability.

The 3-day treatment period was chosen as most experimental or registered therapies as N-acetyl cysteine or medical device under clinical evaluation in ALF. It is also the time delay where physicians are assessing the prognosis of spontaneous recovery in patients having ALF, and deciding whether or not to list the patient on the registry for liver transplantation. This time period also covers the disease period where ALF-5755 efficacy will be shown, as the efficacy of ALF-5755 has been demonstrated at 6 hours and above after induction of ALF in the Fas mice model.

The route and mode of administration, a slow intravenous infusion over 10 minutes, have been kept as in the First In Man study.

10.6. CRITERIA FOR SCHEDULE ADJUSTMENT/DOSE-MODIFICATION OF INVESTIGATIONAL PRODUCT

Dose-modification is not permitted during the course of this study.

10.7. INVESTIGATIONAL PRODUCT ACCOUNTABILITY

When the IP is received by the Investigator (or pharmacist), he/she will check for accurate delivery and acknowledge receipt by signing (or initialling) and dating the documentation provided by or on behalf of Alfact and returning it to Alfact or delegate. A copy will be retained for the Investigator file.

Supplies are shipped to the investigation site as needed. Drug accounting will be reviewed by the IDM during routine monitoring visits: The dispensing of the IP will be carefully recorded on appropriate drug accountability forms (these will be provided by ORION Clinical Services) and an accurate accounting will be available for verification by the IDM at each monitoring visit.

IP accountability records will include:

- confirmation of delivery of the IP to the trial site,
- the inventory at the site,
- the use by each patient,
- the return to the Sponsor or alternative disposition of unused product(s).

They should include dates, quantities, batch numbers, expiration dates (if applicable), and any unique code numbers assigned to the IP(s) and/or patients. Investigators should maintain records which document adequately that:

- the patients were provided the doses specified by the protocol/amendment(s),
- all IP provided by the Sponsor was fully reconciled.

Unused IP must not be discarded or used for any purpose other than the present study.

At the completion or termination of the study, a final drug accountability review and reconciliation must be completed, and any discrepancies must be investigated and their resolution documented. The IDM will review the drug accountability forms and check all IP returns (both unused and used vials) prior to making arrangements for their return to the Sponsor, or authorising their destruction by the study site. All full, partially full and empty containers of IP must be returned to the Sponsor with the appropriate form

IP that has been dispensed to a centre and returned unused must not be re-dispensed to a different centre.

10.8. TREATMENT COMPLIANCE

All doses will be administered in the clinical unit under direct supervision of the Investigator or an attending physician. All drug administration information will be recorded in the CRF and in the drug movement form.

11. CONCOMITANT THERAPY/MEDICAL MANAGEMENT OF ADVERSE EVENTS

Reasonable efforts will be made to determine all relevant treatments received by the patient within 30 days before IP administration. All relevant information must be recorded on the patient's CRF.

Any medications, with the exceptions noted in Section 11.1 below, which are considered necessary for the patient's welfare, and which will not interfere with the study medication, may be given at the discretion of the Investigator. Medications/therapies which MUST be administered as part of the study treatment schedule are listed in Section 11.2.

Administration of **all concomitant drugs** must be reported in the appropriate section of the CRF along with dosage information, dates of administration and reasons for use. Additionally any unplanned diagnostic, therapeutic or surgical procedure performed during the study period must be recorded in the comments section of the corresponding AE report.

11.1. MEDICATIONS THAT MAY NOT BE ADMINISTERED

The use of any herbal/natural products (including the use of vitamins, nutritional supplements) or other "folk remedies" should be discouraged, unless deemed necessary by the investigator (as part of symptom management or standard of care). The patients must be instructed that no additional medication will be allowed without the prior consent of the investigator. Any medication considered necessary for the patient's safety and wellbeing may be given at the discretion of the investigator. Use of these products and all other concomitant medications must be recorded in the CRF.

11.2. MANDATORY CONCOMITANT THERAPY

Patients should continue and receive (in addition to ALF-5755 or placebo) the best standard treatment on assessment of investigators.

12. SCHEDULE OF VISITS

12.1. PRE-STUDY EVALUATION

A complete pre-study baseline evaluation will be conducted including the procedures outlined in Section 12.1.1 below.

Baseline assessments will take place prior to randomization.

12.1.1. Study Baseline assessment (prior to randomization)

- Written Informed Consent
- Eligibility criteria (inclusion/exclusion criteria)
- Medical history
- Demographic data including age and gender
- Clinical examination
- Weight, height and BMI
- Vital signs (BP, heart rate [HR], respiratory rate [RR])
- Body temperature
- HE grade and Glasgow Coma Score (GCS)
- PR, FV, INR, ALT and AST
- Safety laboratory assessments, including haematology (haemoglobin, hematocrit, complete WBC count, red blood cell (RBC) count platelet count); biochemistry (fibrinogen, alkaline phosphatase, gamma-glutamyltransferase [GGT], total and conjugated bilirubin, ammonia, albumin, creatinine, urea, sodium, potassium, chloride, bicarbonates, lactates, calcium, magnesium, phosphorus, glucose, total proteins, amylase and lipase)
- ECG
- Beta human chorionic gonadotropin (hCG) blood pregnancy test for females of child-bearing potential
- Acetaminophen plasma level
- Alcohol plasma level
- Screening of acute and chronic viral infections (serology and/or polymerase chain reaction [PCR]): HAV, HBV, Hepatitis C Virus (HCV), Hepatitis D Virus (HDV), HEV, HSV, Varicella Zoster Virus (VZV) and HIV
- Autoimmune markers (antinuclear antibody [ANA], anti-smooth muscle antibody [ASMA], antibodies to liver and kidney microsomes [anti-LKM], and immunoglobulin [Ig] levels)

- Cupremia and ceruloplasmin plasma levels, and cupruria
- Recording of concomitant medication
- AE recording (AEs should be recorded from the timepoint that the PICD has been signed)

12.2. TREATMENT PERIOD

12.2.1. Schedule of visits for on-treatment period

Treatment will be administered once the patient has been confirmed as being eligible for inclusion in the study. The treatment period will last from H0 until H60. Patients will be hospitalised at least throughout the treatment period up to 84 hours (H84) after treatment initiation at H0.

12.2.2. Treatment administration/general procedures

Investigational Product, ALF-5755, and placebo will be administered every 12 hrs from H0 until H60 as per Section 10.4 as a slow intravenous infusion over 10 minutes with an automatic syringe.

During each infusion, the patient should be confined to bed.

Blood samples are to be taken from the patient's arm **not** containing the syringe for the study treatment infusion.

12.2.3. Assessments to be performed during the treatment period

Patients will follow a treatment period in the clinical hospital unit and under permanent medical and nursing supervision for 4 days from H0 (1st injection) to H84 (i.e. 84 hours after 1st injection), which includes a mandatory hospital in-patient follow-up up to 24 hours after the last infusion. All procedures to be performed during this treatment period are detailed in the Study Flow Chart, Appendix C. Further details are provided in the sections below.

- Randomization prior to the administration of study medication
- PK at H0 just before 1st infusion and H0 10 min (end of infusion), H1, H2, H6, H12 (just before 2nd infusion); H48 just before 5th infusion; H60 just before 6th infusion and H60 10 min (end of infusion), H61, H62, H66, H72, H84
- Study medication administration at H0 (time between biological baseline assessment and the first injection of ALF-5755 or placebo should not exceed 12 hrs), H12, H24, H36, H48 and H60
- Clinical examination at H0 before 1st infusion and 10 minutes after 1st infusion, H12 before 2nd infusion and 10 minutes after 2nd infusion, H24 before 3rd infusion and 10 minutes after 3rd infusion, H36 before 4th infusion and 10 minutes after 4th infusion, H48 before 5th infusion and 10 minutes after 5th infusion, H60 before 6th infusion and 10 minutes after 6th infusion, H72, H84

- Vital signs (BP, HR, RR) at H0 before 1st infusion, 10 minutes after infusion and HR and BP only during 1 hour after 1st infusion, H12 before 2nd infusion, 10 minutes after infusion and HR and BP only during 1 hour after 2nd infusion, H24 before 3rd infusion, 10 minutes after infusion and HR and BP only during 1 hour after 3rd infusion, H36 before 4th infusion, 10 minutes after infusion and HR and BP only during 1 hour after 4th infusion, H48 before 5th infusion, 10 minutes after infusion and HR and BP only during 1 hour after 5th infusion, H60 before, 10 minutes after infusion and HR and BP only during 1 hour after 6th infusion, H72, H84
- Body temperature at H0 before 1st infusion, H12 before 2nd infusion, H24 before 3rd infusion, H36 before 4th infusion, H48 before 5th infusion, H60 before 6th infusion, H72, H84
- HE grade and GCS at H0 before 1st infusion, H12 before 2nd infusion, H24 before 3rd infusion, H36 before 4th infusion, H48 before 5th infusion, H60 before 6th infusion, H72, H84
- PR, FV, INR, ALT, AST at H0 before 1st infusion, H12 before 2nd infusion, H24 before 3rd infusion, H36 before 4th infusion, H48 before 5th infusion, H60 before 6th infusion, H72, H84
- Safety laboratory assessments, including haematology (haemoglobin, hematocrit, RBC count, complete WBC count, platelet count) and biochemistry (fibrinogen, alkaline phosphatase, GGT, total and conjugated bilirubin, albumin, sodium, potassium, chloride, bicarbonates, calcium, magnesium, phosphorus, glucose, total proteins, ammonia, amylase, lipase, creatinine, urea) at H0 before 1st infusion, H12 before 2nd infusion, H24 before 3rd infusion, H36 before 4th infusion, H48 before 5th infusion, H60 before 6th infusion, H72; H84 and urinalysis (glucose, proteins, nitrites, ketones, leucocytes, blood density, pH) at H0 before 1st infusion, H24 before 3rd infusion, H48 before 5th infusion, H72
- ECG at H0 before 1st infusion, 10 minutes and 1 hour after 1st infusion, H24 before 3rd infusion, 10 minutes and 1 hour after 3rd infusion, H48 before 5th infusion, 10 minutes and 1 hour after 5th infusion, H72, H84
- Anti-ALF-5755 antibody plasma levels at H0 before 1st infusion
- CK18 at H0 before 1st infusion, H12 before 2nd infusion, H24 before 3rd infusion, H36 before 4th infusion, H48 before 5th infusion, H60 before 6th infusion, H72, H84
- Recording of concomitant medication and monitoring of AEs at all time points
- Arterial blood gas with lactates in case of grade I or II hepatic encephalopathy or GCS < 15

12.3. DAY 8 VISIT AND FINAL VISIT ON DAY 21

Patients will undergo an inpatient or outpatient hospital visit at D8 (after H0) that will include the following assessments:

- Clinical examination, vital signs (BP, HR, RR) and body temperature
- HE grade and GCS

- PR, FV, INR, ALT and AST
- Safety laboratory assessments, including haematology (haemoglobin, hematocrit, RBC count, complete WBC count, platelet count); biochemistry (fibrinogen, alkaline phosphatase, GGT, total and conjugated bilirubin, albumin, sodium, potassium, chloride, bicarbonates, calcium, magnesium, phosphorus, glucose, total proteins, ammonia, amylase, lipase, creatinine, urea) and urinalysis (glucose, proteins, nitrites, ketones, leucocytes, blood density, pH)
- ECG
- Anti-ALF-5755 antibodies plasma levels
- Recording of concomitant medication and monitoring of AEs at all time points
- Arterial blood gas with lactates in case of grade I or II hepatic encephalopathy or GCS < 15

The End of Study visit will be on D21 (after H0). Patients will undergo an inpatient or outpatient hospital visit that will include the following assessments:

- Clinical examination, vital signs (BP, HR, RR) and body temperature
- HE grade and GCS
- PR, FV, INR, ALT, AST
- Safety laboratory assessments, including haematology (haemoglobin, hematocrit, RBC count, complete WBC count, platelet count); biochemistry (fibrinogen, alkaline phosphatase, GGT, total and conjugated bilirubin, albumin, sodium, potassium, chloride, bicarbonates, calcium, magnesium, phosphorus, glucose, total proteins, ammonia, amylase, lipase, creatinine, urea) and urinalysis (glucose, proteins, nitrites, ketones, leucocytes, blood density, pH)
- ECG
- Anti-ALF-5755 antibodies plasma levels
- Beta hCG blood pregnancy test for females of child-bearing potential
- Recording of concomitant medication and monitoring of AEs
- Arterial blood gas with lactates in case of grade I or II hepatic encephalopathy or GCS < 15

12.4. PREMATURE DISCONTINUATION

If the patient withdraws from the study for any of the reasons described in Section 9.5.1 or is discharged from the hospital, the following assessments will be performed if at all possible:

- Clinical examination, vital signs (BP, HR, RR) and body temperature
- HE grade and GCS
- PR, FV, INR, ALT and AST

- Safety laboratory assessments, including haematology (haemoglobin, hematocrit, RBC count, complete WBC count, platelet count); biochemistry (fibrinogen, alkaline phosphatase, GGT, total and conjugated bilirubin, albumin, sodium, potassium, chloride, bicarbonates, calcium, magnesium, phosphorus, glucose, total proteins, ammonia, amylase, lipase, creatinine, urea) and urinalysis (glucose, proteins, nitrites, ketones, leucocytes, blood density, pH)
- ECG
- PK
- Anti-ALF-5755 antibodies plasma levels
- Beta hCG blood pregnancy test for females of child-bearing potential
- CK18
- Recording of concomitant medication and monitoring of AEs
- Arterial blood gas with lactates in case of grade I or II hepatic encephalopathy or GCS < 15

Study personnel need to ensure that all female patients of child bearing potential are on appropriate contraception at the time of discharge. No oral contraception will be allowed.

In the case of an ongoing AE, appropriate safety evaluations should be repeated more frequently and/or additional tests performed at any time when clinically indicated or at the discretion of the Investigator, until resolution or a period of 21 days from the last dose of IP has elapsed, whichever is the shorter. If the patient refuses for any of the above assessments to be performed, or if the patient is lost to follow up, then this should be noted in the CRF.

13. ASSESSMENTS OF EFFICACY

13.1. SPECIFICATION OF EFFICACY PARAMETERS

The following efficacy parameters will be assessed:

Primary endpoint:

- Rate of change of PR during 72 hours following treatment initiation

Secondary endpoints:

- Rate of change of FV during 72 hours following treatment initiation
- Rate of change of INR during 72 hours following treatment initiation
- Rate of change of ALT plasma level during 72 hours following treatment initiation
- Rate of change of AST plasma level during 72 hours following treatment initiation
- Change of HE grade during 72 hours following treatment initiation
- Overall survival rate at D21
- Transplant-free survival rate at D21
- Liver transplantation at D21
- Length of hospitalisation (days)

Exploratory endpoint:

- Total and cleaved CK18 plasma levels

13.2. METHODS AND TIMING FOR ASSESSING AND RECORDING EFFICACY PARAMETERS

Prothrombin Ratio

To measure the efficacy of ALF-5755 versus placebo, PR will be measured at baseline, H0 before 1st infusion, H12 before 2nd infusion, H24 before 3rd infusion, H36 before 4th infusion, H48 before 5th infusion, H60 before 6th infusion, H72, H84, D8 (after H0), D21 (after H0) or premature discontinuation. The rate of change during the course of the trial of PR will be measured.

Factor V

FV will be measured at baseline, H0 before 1st infusion; H12 before 2nd infusion, H24 before 3rd infusion, H36 before 4th infusion, H48 before 5th infusion, H60 before 6th infusion, H72, H84, D8 (after H0), D21 (after H0) or premature discontinuation. The rate of change during the course of the trial of FV will be measured.

International Normalized Ratio

INR will be measured at baseline, H0 before 1st infusion, H12 before 2nd infusion, H24 before 3rd infusion, H36 before 4th infusion, H48 before 5th infusion, H60 before 6th infusion, H72, H84, D8 (after H0), D21 (after H0) or premature discontinuation. The rate of change during the course of the trial of INR will be measured.

Transaminases

The rate of change during the course of the trial of ALT and the rate of change during the course of the trial of AST will each be measured. Both ALT and AST decreases will be measured by their slopes at baseline, H0 before 1st infusion, H12 before 2nd infusion, H24 before 3rd infusion, H36 before 4th infusion, H48 before 5th infusion, H60 before 6th infusion, H72, H84, D8 (after H0), D21 (after H0) or premature discontinuation.

Hepatic encephalopathy grade

The grade of HE during the course of the trial grade will be measured at baseline, H0 before 1st infusion, H12 before 2nd infusion, H24 before 3rd infusion, H36 before 4th infusion, H48 before 5th infusion, H60 before 6th infusion, H72, H84, D8 (after H0), D21 (after H0) or premature discontinuation. The change of HE during the course of the trial will be measured.

Survival

The number of patients alive will be considered at D21 to estimate survival within this study.

Liver Transplantation

Liver transplantation at D21 should be assessed.

Total and cleaved CK 18

CK18 will be measured at H0 before 1st infusion, H12 before 2nd infusion, H24 before 3rd infusion, H36 before 4th infusion, H48 before 5th infusion, H60 before 6th infusion, H72; H84 or premature discontinuation.

Methods for analyses of all efficacy variables are described in Section 15.4.

14. ASSESSMENTS OF SAFETY

14.1. SPECIFICATION OF SAFETY PARAMETERS

To determine the safety and tolerability of ALF-5755, evaluations will be included as described in the Study Flow Chart (Appendix C):

Comparison of the frequency of:

- local and systemic adverse events (AEs/SAEs) (AEs should be recorded from the timepoint that the PICD has been signed),

Assessment of:

- laboratory findings (including haematology, biochemistry and urinalysis),
- clinical examination, clinically significant vital signs (BP, HR, RR), body temperature,
- HE grades (Appendix E) and GCSs (Appendix F),
- ECG abnormalities,
- anti-ALF-5755 antibodies plasma levels,
- Beta hCG blood pregnancy test in women of childbearing potential,
- Arterial blood gas in case of grade I or II HE or GCS < 15.

14.1.1. Clinical examination, body temperature and vital signs

- The following baseline parameters will be measured at baseline only: Weight, height and BMI; patients' acetaminophen plasma and alcohol plasma levels; screening of acute and chronic viral infections (serology and/or PCR), including: HAV, HBV, HCV, HDV, HEV, HSV, VZV and HIV; autoimmune markers, including: ANA, ASMA, anti-LKM Abs and Ig levels; cupremia and ceruloplasmin plasma levels, and cupruria.
- Clinical examination at H0 before 1st infusion and 10 minutes after 1st infusion, H12 before 2nd infusion and 10 minutes after 2nd infusion, H24 before 3rd infusion and 10 minutes after 3rd infusion, H36 before 4th infusion and 10 minutes after 4th infusion, H48 before 5th infusion and 10 minutes after 5th infusion, H60 before 6th infusion and 10 minutes after 6th infusion, H72; H84, D8 (after H0), D21 (after H0) or premature discontinuation and body temperature at H0 before 1st infusion, H12 before 2nd infusion, H24 before 3rd infusion, H36 before 4th infusion, H48 before 5th infusion, H60 before 6th infusion, H72; H84 D8 (after H0), D21 (after H0) or premature discontinuation.
- Vital signs (BP, HR, RR) at baseline, H0 before 1st infusion, 10 minutes after infusion and HR and BP only during 1 hour after 1st infusion, H12 before 2nd infusion, 10 minutes after infusion and HR and BP only during 1 hour after 2nd infusion, H24 before 3rd infusion, 10 minutes after infusion and HR and BP only during 1 hour after 3rd infusion, H36 before 4th infusion, 10 minutes after infusion and HR and BP

only during 1 hour after 4th infusion, H48 before 5th infusion, 10 minutes after infusion and HR and BP only during 1 hour after 5th infusion, H60 before, 10 minutes after infusion and HR and BP only during 1 hour after 6th infusion, H72; H84, D8 (after H0), D21 (after H0) or premature discontinuation.

Qualified staff members will perform BP measurements. BP measurements will be taken from the same arm used for blood sample collection by automated BP monitor using the oscillometric method (e.g. Dinamap). If there is a clinically important change in BP from the previous recording, measurements will be repeated immediately to confirm the change. HR will be monitored with a heart rate monitoring device.

BP and HR will be recorded in supine position (after a 5 minutes rest).

The body temperature will be measured using a tympanic thermometer.

14.1.2. Safety laboratory assessments

All samples should be taken from the opposite arm to the one where the study treatment is infused.

Blood safety analyses include:

Haematology: haemoglobin, hematocrit, complete WBC count, RBC count and platelet count.

Biochemistry: fibrinogen, alkaline phosphatase, GGT, total and conjugated bilirubin, ammonia, albumin, creatinine, urea, sodium, potassium, chloride, bicarbonates, lactates, calcium, magnesium, phosphorus, glucose, total proteins, amylase and lipase.

All clinically significant (CS) abnormal laboratory test values identified after IP administration will be repeated until the values return to normal or baseline. If laboratory values do not return to normal or baseline within a reasonable period, the aetiology should be identified and the Sponsor notified.

The safety urinalysis will involve semi-quantitative (dipsticks): glucose, proteins, nitrites, ketones, leucocytes, blood density, pH. This will be followed by a microscopic urinalysis if required.

Haematology and biochemistry will be collected at baseline, H0 before 1st infusion, H12 before 2nd infusion, H24 before 3rd infusion, H36 before 4th infusion, H48 before 5th infusion, H60 before 6th infusion, H72; H84 D8 (after H0), D21 (after H0) or premature discontinuation. Urinalysis will be collected at H0 before 1st infusion, H24 before 3rd infusion, H48 before 5th infusion, H72; D8 (after H0), D21 (after H0) or premature discontinuation.

14.1.3. Electrocardiogram

Measured ECG parameters include: HR, PR, QRS duration, QRS axis deviation, QT, QTcF and QTcB.

Patients will always rest in the supine position for at least 10 minutes before the ECG recording is started. A qualified physician will review the ECGs promptly and any clinically important finding will be recorded on the appropriate CRF. The Investigator is responsible for providing the interpretation of all ECGs. This should include a description from the

Investigator assessing the ECG (normal, abnormal not CS [NCS], abnormal CS and abnormality described).

ECG will be measured at baseline, H0 before 1st infusion, 10 minutes and 1 hour after 1st infusion, H24 before 3rd infusion, 10 minutes and 1 hour after 3rd infusion, H48 before 5th infusion, 10 minutes and 1 hour after 5th infusion, H72; H84, D8 (after H0), D21 (after H0) or premature discontinuation.

14.1.4. Hepatic encephalopathy grade (HE grade) and Glasgow Coma Score (GCS)

HE grade and GCS will be measured at baseline, H0 before 1st infusion, H12 before 2nd infusion, H24 before 3rd infusion, H36 before 4th infusion, H48 before 5th infusion, H60 before 6th infusion, H72, H84, D8 (after H0), D21 (after H0) or premature discontinuation.

Refer to Appendix E and Appendix F for more information on HE grade and GCS, respectively.

14.1.5. Anti-ALF-5755 antibody plasma levels

Method of assessment

Blood samples (10 mL) will be collected in dry Vacutainer™ tubes (Becton Dickinson UK, Ltd., Oxford). The blood samples will be centrifuged within 60 minutes of collection at 1500 g and 4°C for 10 minutes. For the antibody analysis and the search for neutralising activity, 2 aliquots of approximately 1.25 mL each of serum will be withdrawn and placed into 4 polypropylene tubes (2 tubes for each laboratory), which will be labelled, frozen and stored at -20°C until analysis.

A total of 3 blood samples will be drawn for these analyses according to the following schedule: H0 (before first treatment infusion), Day 8 (after H0) and Day 21 (after H0) or premature discontinuation.

Laboratory antibody determinations

The analyses will be performed by:

ATLANBIO S.A.S
Route de Saint André des Eaux
Z.I de Brais - B.P 40309
44605 Saint Nazaire – FRANCE

Two serum samples will be shipped in a container filled with enough dry ice to ensure that the samples will be kept frozen up to delivery time at ATLANBIO S.A.S.

In case of presence of antibodies, the neutralising activity of these antibodies will be evaluated by:

INSERM (Institut National de la Santé et de la Recherche Médicale)
Unité INSERM U785
Directeur: Pr Didier Samuel
Responsable scientifique (point de contact): Dr Jamila Faivre
Centre HépatoBiliaire
12-14 Avenue Paul-Vaillant-Couturier

94800 Villejuif
Tel: 01 45 59 60 75
Email: jamila.faivre@inserm.fr

Two serum samples will be shipped in a container filled with enough dry ice to ensure that the samples will be kept frozen up to delivery time at Inserm U785.

14.2. ADVERSE EVENT REPORTING

It is of the utmost importance that all staff involved in the study is familiar with the content of this section. The Investigator at each study centre is responsible for ensuring compliance amongst the relevant staff members at his specific centre.

14.2.1. Definition of an Adverse Event

An AE is defined as any untoward medical occurrence in a patient or clinical investigation subject administered a pharmaceutical product, occurring at any dose and which does not necessarily have a causal relationship with the treatment. An AE can therefore be any unfavourable and unintended sign (including an abnormal laboratory finding), symptom, or disease temporally associated with the use of a medicinal (investigational) product, whether or not related to the medicinal (investigational) product[1].

An AE can refer to an untoward response to the administration of the IP, but can also occur as a result of the protocol-required procedures. Therefore, safety data will be collected after written informed consent has been obtained and before administration of the IP.

AEs include the following:

- All suspected adverse medication reactions.
- All reactions from medication overdose, abuse, withdrawal, sensitivity, or toxicity.
- Apparently unrelated illnesses, including the worsening of a pre-existing illness.
- Injury or accidents. Note that if a medical condition is known to have caused the injury or accident, the medical condition and the accident should be reported as two separate AEs.
- Abnormalities in physiological testing or physical examination findings that require clinical intervention or further investigation (beyond ordering a repeat [confirmatory] test).
- Laboratory abnormalities that require clinical intervention or further investigation (beyond ordering a repeat [confirmatory] test) unless they are associated with an already reported clinical event. Any new or aggravated clinically relevant abnormal medical finding at a physical examination as compared with the baseline assessment must be reported as an AE. Where possible the Investigator should use the clinical and not the laboratory term (e.g. hyperkalaemia versus high potassium).

14.2.2. Serious Adverse Events

14.2.2.1. Definition of a Serious Adverse Event

An SAE[1] is defined as any untoward medical occurrence that at any dose:

- Results in death,

  Death is an outcome of an AE, and not an AE in itself. In reports of death due to "Disease Progression", where no other information is provided, the death will be assumed to have resulted from progression of the disease being treated with the IP(s).

- Is life-threatening (i.e. the patient was at risk of death at the time of the event. It does not refer to an event which hypothetically might have caused death if it were more severe),
- Requires inpatient hospitalisation or prolongation of existing hospitalisation,

  Complications that occur during hospitalizations are AEs. If a complication prolongs hospitalization, it is a SAE.

  "In-patient hospitalization" means the patient has been formally admitted to a hospital for medical reasons, for any length of time. This may or may not be overnight. It does not include presentation and care within an emergency department.

- Results in persistent or significant disability/incapacity,
- Is a congenital anomaly/birth defect,
- Is an important medical event(s) that may not be immediately life-threatening or result in death or hospitalization but that may jeopardize the patient or require intervention to prevent one of the above outcomes.

Medical and scientific judgment should be exercised in deciding whether a case is serious.

"Occurring at any dose" does not imply that the patient is receiving IP at the time of the event. Dosing may have been given as treatment cycles or interrupted temporarily prior to the onset of the SAE, but may have contributed to the event.

14.2.3. Recording and Reporting of Adverse Events

All AEs, as defined above, encountered during the clinical study as well as any **SAEs** (see Section 14.2.2) will be reported in the appropriate section of the CRF. It is important that this includes the duration of the AE (onset/resolution dates), the severity, the relationship to the drug and any concomitant treatment dispensed (or other action taken) (see Sections 14.2.3.5, and 14.2.3.6 below). AE data should be obtained though observation of the patient, from any information volunteered by the patient, or through patient questioning. The subject may be asked "Do you have any health problems?" or "Have you had any health problems since your last clinic visit?"

14.2.3.1. Reporting of Signs and Symptoms versus a Diagnosis

Recording a diagnosis (when possible) is preferred to recording a list of associated signs and symptoms. However, if a diagnosis is known but there are associated signs or symptoms not generally attributed to the diagnosis, the diagnosis and each sign or symptom must be recorded separately.

14.2.3.2. Disease Progression

Disease progression can be considered as a worsening of a patient’s condition attributable to the condition for which the IP is being studied. It may be reflected by an increase in the severity of the condition or an increase in the symptoms. Disease progression and any events that are unequivocally due to disease progression should be recorded only in the CRF and should not be reported as an SAE.

14.2.3.3. Death

All deaths that occur during the AE Reporting period (refer to Section 14.2.5) must be reported as follows:

- Death (clearly) due to disease progression should be documented in the CRF but should not be reported as an SAE
- Death that is not due (or not clearly due) to disease progression should be documented in the CRF and as an SAE within 24 hours (see Section 14.2.3.7). The report should detail the main and contributory causes of death. This information should also be accompanied by a death certificate or autopsy report (if available)

14.2.3.4. Pregnancy

Should a pregnancy occur in a female patient, it must be reported in accordance with the procedures described in Section 14.2.3.7 and the patient will be followed-up to the end of her pregnancy. Pregnancy in itself is not regarded as an AE unless there is a suspicion that an IP may have interfered with the effectiveness of a contraceptive medication.

A beta hCG blood pregnancy test will be done at baseline, D21 (after H0) or premature discontinuation.

14.2.3.5. Definition of relationship of AEs to the Investigational Product

The Investigator must assess the possible relationship between the AE and the IP(s) and record that assessment in the CRF. The Investigator is to make his/her own assessment of each SAE to be recorded on the CRF and on the SAE form.

The Investigator should provide a Yes or No assessment as to whether there is a reasonable possibility that the event may have been caused by the IP.

The relationship should be assessed according to the criteria in Table 14-1 below:

Table 14-1: Relationship of the Adverse Event to the Investigational Product

None	A causal relationship to the IP is not suspected; the temporal relationship of the clinical event to study drug administration makes a causal relationship unlikely or other drugs, therapeutic interventions or underlying conditions provide sufficient explanation for the observed event
Related	A causal relationship to the study drug is suspected; the temporal relationship of the clinical event to study drug administration makes a causal relationship possible, and other drugs, therapeutic interventions or underlying conditions do not provide a sufficient explanation for the observed event

14.2.3.6. Definition of Severity of Adverse Events

Severity of any AE will be graded according to Common Toxicity Criteria (CTC) where applicable.

For each episode, the highest severity grade attained should be reported.

If an AE occurs that is not listed in the CTC the Investigator will evaluate its severity using the definitions in Table 14-2.

Table 14-2: Definition of Severity of Adverse Events

Mild	Grade 1 - Does not interfere with subject's usual function (awareness of symptoms or signs, but easily tolerated [acceptable]).
Moderate	Grade 2 - Interferes to some extent with subject's usual function (enough discomfort to interfere with usual activity [disturbing]).
Severe	Grade 3 - Interferes significantly with subject's usual function (incapacity to work or to do usual activities [unacceptable])
Life Threatening	Grade 4 - Results in risk of death, organ damage, or permanent disability (unacceptable)
Death	Grade 5 – Event has a fatal outcome

Note the distinction between serious and severe AEs. **Severe** is a measure of intensity whereas an AE must meet one of the criteria for seriousness listed in Section 14.2.2 to be considered **serious**; thus, a **severe** reaction is not necessarily a **serious** reaction. For example, a headache may be severe in intensity, but would not be classified as serious unless it met one

of the criteria for serious events listed in Section 14.2.2. An AE that is assessed as Grade 3 (severe) or Grade 4 (potentially life-threatening) should not be confused with a SAE.

14.2.3.7. SAE reporting procedure for Investigators to ORION

The Investigator must report all initial and follow-up reports regarding SAEs (including pregnancy and events described in Section 14.2.3), regardless of presumed causal relationship, to the ORION Clinical Services' Pharmacovigilance Department, by fax or phone. A fax of the relevant SAE form must be sent within 24 hours of awareness of the event. Details of the relevant fax number for SAEs are as follows ORION fax number: +44 (0)1753 695101 or +44 (0)1753 695124 (backup); ORION phone number: +44 (0)1753 578080.

If, for any reason, it is not possible to complete all sections of the SAE form within 24 hours, transmission of the form must not be delayed and the outstanding information should be sent on a follow-up SAE form.

If the SAE is reported by telephone, all points on the SAE form should be covered in the initial telephone report and followed by a completed and signed SAE form to verify the verbal information given previously. In addition, the event must be documented in the CRF.

Information on SAEs will be recorded on a specific SAE form. Blank copies are included in the study Investigator's File.

The SAE form must be completed as fully as possible with information relevant to the SAE(s) being reported. All fields should be populated or marked accordingly if no information is available.

For all SAEs where important or relevant information is missing, active follow-up should be undertaken. Investigators or other site personnel should inform the ORION Pharmacovigilance Department of any follow-up information on a previously reported SAE immediately but no later than 24 hours after they become aware of the SAE. The follow-up information must be presented on an SAE form marked as follow-up. It is necessary only to provide the new information, with the SAE form signed by an Investigator.

Investigators or other site personnel should send relevant CRF modules by fax to the ORION's Pharmacovigilance Department and any other relevant or requested supporting documentation (e.g., ECG, laboratory results, autopsy report).

The Investigator will ensure that all the necessary information is provided within the timelines stipulated by the ORION Pharmacovigilance Department when the request for information is made.

Follow-up reports (as many as required) should be completed and faxed following the same procedure above.

14.2.4. Reporting Serious Adverse Events to EC

The Sponsor is responsible for informing local Ethics Committees (ECs) of the applicable safety reports in compliance with local regulations. Copies of all correspondence relating to

reporting of any safety reports to the EC should be maintained in the Investigator's Files and provided to ORION Clinical Services.

The reference document for definition of expectedness is the IB for ALF-5755.

The Sponsor, or its designee, ORION Clinical Services, will inform Investigators, central ECs and regulatory authorities of applicable safety reports, as required.

14.2.5. Adverse Event Reporting Period

ALL AEs which occur during the treatment period, i.e. from the date of written informed consent to the final study visit, will be recorded in the CRF.

All AEs experienced by a patient, irrespective of the suspected causality, will be monitored until the event has resolved, any abnormal laboratory values have returned to baseline or stabilised at a level acceptable to the Investigator and Medical Monitor, until there is a satisfactory explanation for the changes observed, or until the patient is lost to follow-up.

In addition, any known untoward event that occurs subsequent to the AE reporting period that the Investigator assesses as serious and related to the IP should also be reported as an SAE.

14.3. PHARMACOKINETIC EVALUATION

This full PK evaluation in patients after the first and the last infusion will allow PK information to be obtained in patients in order to study possible modifications of ALF-5755 PK due to the patient's condition and to quantify a potential accumulation after repeated administration.

ALF-5755 plasma concentrations will be performed in the first 16 patients included at Paul Brousse Hospital and Beaujon Hospital. Baseline plasma concentrations measured at H0, reflecting endogenous HIP/PAP levels will be subtracted from each ALF-5755 plasma concentration. PK samples will be collected at the following times:

- 1st infusion: H0 just before 1st infusion, 10 minutes (end of infusion), H1, H2, H6, H12 (just before 2nd infusion)
- 5th infusion: H48 (just before 5th infusion)
- 6th infusion: H60 just before 6th infusion, H60 10 minutes (end of infusion), H61, H62, H66, H72, H84

PK parameters (C_{max}, t_{max}, AUC_{0-12}, AUC_{0-t} and $t_{1/2}$) of ALF-5755 will be determined from plasma concentrations measured after 1st infusion (H0) and after the 6th infusion (H60).

All PK blood samples (of 5 mL each) will always be taken from the opposite arm to the one where study treatment is infused.

Sample shipment and storage

The samples will be shipped in a container filled with enough dry ice to ensure that the samples are kept frozen.

Study samples will be shipped to:

ATLANBIO S.A.S
Route de Saint André des Eaux
Z.I de Brais - B.P 40309
44605 Saint Nazaire – FRANCE

Information regarding the vials the blood samples will be collected in the total volume of PK assays to be collected, the temperature and storage conditions will be detailed in a separate laboratory manual provided to the site.

14.4. DATA SAFETY MONITORING BOARD (DSMB)

The DSMB including experts will be created and will review key safety data. The DSMB can decide at any time to terminate the study.

Safety data will be reported to the DSMB following treatment completion in the first 2 patients; following treatment completion in the first 4 patients; and following treatment completion in every 10 patients thereafter (10, 20, 30, 40, 50, 60 and above as applicable).

Details about DSMB (e.g., composition, meeting information, data, etc.) will be described in the DSMB charter.

15. EVALUATION OF RESULTS

15.1. SAMPLE SIZE AND STUDY POWER

The sample size has been determined from the Beaujon series for patients that fulfilled the inclusion criteria for this study.

Rate of change of PR was calculated and the mean and standard deviation were compared by 24H intervals: 0-24H, 24-48H, 48-72H.

The rate of change in the second interval (24-48H) was slightly higher but the difference was not statistically significant. Therefore the time intervals were combined and the rate of change for the time period 0-72H was retained.

Table 15-1: PR evolution

**All groups**	**Slope 0-24**	**Slope 24-48**	**Slope 48-72**	**Slope 0-72**
**N**	28	25	24	23
**Mean**	0.12	0.29	0.17	0.22
**SD**	0.45	0.40	0.33	0.27

From these results, the number of patients to be included in the trial was calculated based on a student t test (G-power software) with the following parameters:

- α risk = 0.05
- β risk = 0.20
- Two-tailed test
- Treatment effect: Mean for ALF-5755 efficacy = mean for placebo efficacy x 2

Since the variance of PR rate of change in the ALF-5755 group is unknown, calculations of sample size were performed under various assumptions:

- Large standard deviation (0.44) equal to the expected mean in the ALF-5755 group which corresponds to what was observed in the Beaujon series. The number of required patients is then 45 per group for an effect-size of 0.60.
- For a smaller standard deviation (0,32) the number of required patients decreases to 30 per group for an effect-size of 0.74.

- For a standard deviation (0,27) equal to the standard deviation in the placebo group, the number of required patients decreases to 25 per group for an effect-size of 0.81.
- Results are presented in Table 15-2 below.

Table 15-2: Results

**PR rate of change 0-72H**	**Placebo**	**ALF 5755**	**Efficacy coefficient**	**N patients per group**	**Calculated effect size**
**Mean**	0.22	0.44	2		
**SD1**	0.27	0.44		**45**	0.60
**SD2**	0.27	0.32		**30**	0.74
**SD3**	0.27	0.27		**25**	0.81

By fixing the time interval at 72 hours and by hypothesizing that the rate of change of PR for patients on ALF-5755 will be double the rate of change of PR for patients on placebo, a sample size of about 30 patients per group is required. Considering the recruiting rate of eligible patients in the 2 major centres (Beaujon and CHB) and the 8 other centres that will participate to the trial, this sample size should be reached within 18 months.

15.2. STATISTICAL METHODS

Statistical analyses will be performed by ORION. Statistical analyses will be carried out using SAS®, Version 9.2 or later, SAS Institute, Cary, Northern Carolina, USA.

The statistical methods for this study, summarized below, will be described in a detailed statistical analysis plan (SAP), which will be finalized prior to database lock. Any deviations from the planned analysis as described in the SAP will be justified and recorded in the final study report.

The DSMB, including experts will be created and will review key safety data. This board will be blinded, and can decide at any time to terminate the study.

Safety data will be reviewed by the DSMB following treatment completion in the first 2 patients; following treatment completion in the first 4 patients; and following treatment completion in every 10 patients thereafter (10, 20, 30, 40, 50, 60 and above as applicable). Additional meetings will take place if necessary.

15.2.1. General considerations

Data will be summarized as follows: Continuous variables by descriptive statistics (number of patients [N], mean, standard deviation, minimum, median and maximum); categorical data by absolute and relative frequencies (n and %).

Unless indicated otherwise, summary statistics will be reported for observed data only. Missing data will not be imputed. If a baseline value is missing, no change from baseline will be calculated. Baseline is pre-treatment and will be defined in the SAP.

15.2.2. Populations for analysis

The following populations will be analysed:

Safety Population

The Safety Population will include all patients who are randomised to treatment and receive at least one administration of ALF-5755 or placebo. Subjects who receive the wrong treatment will be analysed as treated when using the Safety Population.

Intent-to-Treat (ITT) Population

The ITT Population will include all patients in the Safety Population who provide at least one post-dose efficacy assessment. Note that subjects who withdrew early due to worsening disease before the first post-baseline efficacy assessment WILL be included as their efficacy assessments will be imputed. Subjects who receive the wrong treatment will be analysed as randomised when using the ITT Population.

Per-Protocol (PP) Population

The PP Population will include all patients in the ITT Population who complete the double-blind treatment phase of the study with no major protocol violations. Protocol violations will be defined in the SAP and discussed and agreed in a blind review meeting prior to database lock.

15.3. BASELINE CHARACTERISTICS

Clinical profiles of ALF-5755 and placebo treated groups will be summarised to identify any baseline imbalance between the treatment groups. Age, gender, etiology, and severity of disease at inclusion will be presented.

15.4. EVALUATION OF EFFICACY

15.4.1. Evaluation of primary efficacy endpoint

The primary endpoint of the study will be:

- **Rate of change of PR** calculated as follows:

[PR at 72 hours (i.e. 12 hours after the last 6th infusion) – PR at H0 pre-dose] / 72 hours

If PR reaches the hospital laboratory normal range of values before 72 hours, the formula will be modified as follows:

[Hospital laboratory normal range of values of PR – PR at H0 pre-dose] / Time (hours) to first reach hospital laboratory normal range of values of PR since H0

If the patient has no recorded PR at 72 hours but does have an earlier post-H0 assessment, the formula will be modified as follows:

[Last measured value of PR – PR at H0 pre-dose] / Time (hours) elapsed when last PR value was measured since H0

If the patient withdraws early, an extra PR sampling at time of withdrawal will be done if possible, and used as the last measured value of PR in this formula.

Rate of change of PR during the 72 hours following treatment initiation in ALF-5755 and placebo arms will be compared by a non parametric test (Wilcoxon). In addition the comparison will be adjusted for sub-groups based on initial PR and etiology using the Cochran-Mantel-Haenszel test.

Analyses will be performed with the ITT Population and the PP Population. A p-value below 0.05 will be considered as significant.

15.4.2. Evaluation of secondary efficacy endpoints

Secondary endpoints of the study will be:

Rate of change of Factor V (FV) plasma level calculated as follows:

[FV at 72 hours (i.e. 12 hours after the last infusion) – FV at H0 pre-dose] / 72 hours

If FV reaches the hospital laboratory normal range of values before 72 hours, the formula will modified as follows:

[Hospital laboratory normal range of values of FV – FV at H0 pre-dose] / Time (hours) to first reach hospital laboratory normal range of values of FV since H0

If the patient has no recorded FV at 72 hours but does have an earlier post-H0 assessment, the formula will be modified as follows:

[Last measured value of FV – FV at H0 pre-dose] / Time (hours) elapsed when last FV value was measured since H0

If the patient withdraws early, an extra FV sampling at time of withdrawal will be done if possible, and used as the last measured value of FV in this formula.

- **Rate of change of international normalized ratio (INR)** calculated as follows:

[INR at 72 hours (i.e. 12 hours after the last infusion) – INR at H0 pre-dose] / 72 hours

If INR reaches the hospital laboratory normal range of values before 72 hours, the formula will be modified as follows:

[Hospital laboratory normal range of values of INR – INR at H0 pre-dose] / Time (hours) to first reach hospital laboratory normal range of value of INR since H0

If the patient has no recorded INR at 72 hours but does have an earlier post-H0 assessment, the formula will be modified as follows:

[Last measured value of INR – INR at H0 pre-dose] / Time (hours) elapsed when last INR value was measured since H0

If the patient withdraws early, an extra INR sampling at time of withdrawal will be done if possible, and used as the last measured value of INR in this formula.

- **Rate of change of alanine transaminases [ALT] plasma level** calculated as follows:

[ALT at 72 hours (i.e. 12 hours after the last infusion) – ALT at H0] / 72 hours

If ALT reaches the hospital laboratory normal range of values before 72 hours, the formula will be modified as follows:

[Hospital laboratory normal range of values of ALT – ALT at H0 pre-dose] / Time (hours) to first reach hospital laboratory normal range of values of ALT since H0

If the patient has no recorded ALT at 72 hours but does have an earlier post-H0 assessment, the formula will be modified as follows:

[Last measured value of ALT – ALT at H0 pre-dose] / Time (hours) elapsed when last ALT value was measured since H0

If the patient withdraws early, an extra ALT sampling at time of withdrawal will be done if possible, and used as the last measured value of ALT in this formula.

- **Rate of change of aspartate transaminases (AST) plasma level** calculated as follows:

[AST at 72 hours (i.e. 12 hours after the last infusion) – AST at H0] / 72 hours

If AST reaches the hospital laboratory normal range of values before 72 hours, the formula will be modified as follows:

[Hospital laboratory normal range of values of AST – AST at H0 pre-dose] / Time (hours) to first reach hospital laboratory normal range of values of AST since H0

If the patient has no recorded AST at 72 hours but does have an earlier post-H0 assessment, the formula will be modified as follows:

[Last measured value of AST – AST at H0 pre-dose] / Time (hours) elapsed when last PR value was measured since H0.

If the patient withdraws early, an extra AST sampling at time of withdrawal will be done if possible, and used as the last measured value of AST in this formula.

- **Change of HE grade** during 72 hours following treatment initiation
- **Overall survival rate** at D21
- **Transplant-free survival rate** at D21
- **Liver transplant rate** at D21
- **Length of hospitalisation (days)**

Change in HE grade between the treatment groups will be compared using the Cochran-Mantel-Haenszel test.

Overall survival rate, transplant-free survival rate and transplantation rate will be analyzed by the Chi² test and the log rank test. Kaplan-Meier estimates will be presented. Analyses will also be adjusted for etiology.

Rate of change of FV, INR, ALT and AST will be calculated using the same formula as for PR and analysed in the same way. Analyses will use the ITT Population. A p-value below 0.05 will be considered as significant.

15.4.1 Evaluation of exploratory endpoint(s)

Total and cleaved CK18 will be compared amongst the two groups of patients receiving ALF-5755 and placebo.

15.4.2 Sub-group evaluation of efficacy and exploratory endpoint*(s)*

Sub-group analyses of efficacy and exploratory endpoint(s) will be performed. The subgroups will be defined in the SAP.

15.5. OTHER EVALUATIONS

15.5.1. Pharmacokinetic evaluation

Plasma blood levels of ALF-5755 will be determined at several times during infusions in 16 patients. This full PK evaluation in patients after the first and the last infusion will allow PK information to be obtained in patients in order to study possible modifications of ALF-5755 PK due to the patient's condition and to quantify a potential accumulation after repeated administration.

Baseline plasma concentrations measured at H0, reflecting endogenous HIP/PAP levels, will be subtracted from each ALF-5755 plasma concentration.

The following PK parameters will be determined from baseline corrected ALF-5755 plasma concentrations using non-compartmental analysis:

- **C_{max} (ng/mL)**: observed maximal blood concentration.
- **T_{max} (h)**: first time to reach C_{max}.
- **$t_{1/2}$ (h)**: apparent plasma elimination half-life.
- **AUC_{0-12} (ng/mL.h)**: area under the plasma concentration curve from the beginning of infusion up to concentration measured 12 h after the infusion start.
- **AUC_{0-t} (ng/mL.h)**: area under the plasma concentration curve from the beginning of infusion up to the last quantifiable concentration at time t.

Descriptive statistics will be performed on these PK parameters.

The achievement of steady-state will be assessed by comparison of ALF-5755 concentrations on H48, H60 and H72.

The extent of accumulation will be assessed by comparison of AUC_{0-12} at H0 and AUC_{0-12} at H60.

15.6. INTERIM ANALYSIS

Safety data will be reported to the DSMB following treatment completion in the first 2 patients; following treatment completion in the first 4 patients; and following treatment completion in every 10 patients thereafter (10, 20, 30, 40, 50, 60 and above as applicable).

No interim analysis is planned.

16. OBLIGATIONS OF THE PRINCIPAL INVESTIGATOR

The study will be performed in accordance with the protocol, *the Declaration of Helsinki*[2,3] (see Appendix A), International Conference on Harmonisation (ICH) Harmonised Tripartite Guideline for Good Clinical Practice[4] (ICH GCP - see Appendix B for Section on Investigator's Responsibilities) and any local regulations.

16.1. INDEPENDENT ETHICS COMMITTEE (IEC)

Approval of the trial protocol/amendments should be obtained from the Independent Ethics Committee (IEC). Prior to the initiation of the study, the following will need to be submitted:

- the study protocol and any amendments,
- the Patient Informed Consent Documents (PICD) and any other written documents to be provided to the patient,
- the Investigator Brochure,
- details of any compensation to patients,
- the current CV of the Principal Investigator,
- patient recruitment procedures e.g. adverts,
- any other requested document(s),

to an IEC for approval. A copy of the approval will be sent to the Sponsor along with **all** other correspondence with the IEC, including the submission documents. ORION should file all correspondence with the IEC in the Investigator Site File.

The study will not be started until approval of the protocol and the PICD has been obtained from the appropriate IEC. The letter of approval should be dated, and should specify the protocol number and date of the protocol or amendment which was reviewed and approved. It should also specify the date of the PICD that was reviewed and approved.

A dated list of the voting members of the IEC who were present when the protocol was reviewed and approved, including their titles/occupations and institutional affiliations should be included in the Investigator Site File and a copy of the list will be provided by ORION Clinical Services to the Sponsor prior to study initiation. ORION Clinical Services will make all attempts to ensure that the IEC is constituted and operates in accordance with the ICH GCP[3] and any local regulations.

ORION Clinical Services will submit any protocol amendments to the IEC (and other local authorities, according to local regulations) prior to implementation.

ORION Clinical Services will submit required progress reports to the IEC that approved the protocol at least annually, as well as report any SAEs, life-threatening problems or deaths, to comply with ICH GCP[3]. ORION Clinical Services will also inform the IEC of reports of SAEs (provided to him/her by the sponsor) that occurred in other clinical studies conducted with the Investigational Product. ORION Clinical Services must inform the IEC of the termination of the study.

16.2. REGULATORY BODY APPROVAL

The study will not be started until receipt by ORION Clinical Services of approval from relevant regulatory body. ORION Clinical Services will provide the Investigator with a copy of the relevant document on behalf of the Sponsor.

16.3. INFORMED CONSENT AND SCREENING DATA

PICDs will normally be based on a master document provided by ORION Clinical Services and must be approved by ORION Clinical Services and the study Sponsor prior to submission to the IEC. The content of the PICD should reflect that described in Section 4.8.10 of the ICH Guidelines[3] (see Appendix B) and any local requirements e.g. IEC. Any changes requested by the IEC must be approved by ORION Clinical Services prior to the documents being used. A copy of the final, IEC-approved consent form will be submitted by ORION Clinical Services to the Sponsor prior to initiation of this study. The Investigator should file the signed PICDs for possible review by ORION Clinical Services CRAs.

Written informed consent will be obtained from each patient prior to inclusion in the trial, and prior to any study-related assessments are performed, as described in Sections 9.1.

16.4. CASE REPORT FORMS AND SOURCE DOCUMENT VERIFICATION

CRFs of a design mutually agreed upon by the Sponsor and ORION Clinical Services will be supplied by ORION Clinical Services. CRFs are the sole property of ORION Clinical Services (for the Sponsor) and should not be made available in any form to third parties, except for authorised representatives of appropriate Health/Regulatory Authorities, without written permission from ORION Clinical Services or the Sponsor.

A CRF is required and should be completed for each included (consented) patient. The Investigator will be responsible for the accuracy of the data entered in the CRFs. All entries must be written *in* ***ENGLISH*** in black ballpoint pen. Corrections of data should be made using one single line, leaving the corrected data clearly visible. The accurate data should be entered next to the inaccurate data. All changes must be initialled and dated. Correction fluids are not allowed. The Investigator only need sign the withdrawal/completion CRF page(s). If a clinically significant change is made on any of the CRF pages after the Investigator has signed the study withdrawal/completion CRF page, the Investigator must re-sign the study withdrawal/completion page to document that he agrees with the change.

The relevant completed CRF pages must be available for review/collection to designated ORION Clinical Services representatives at each scheduled monitoring visit.

The Investigator will allow designated ORION Clinical Services representatives and regulatory bodies to have direct access to the source documents to verify the data reported in the CRFs.

Source documents are the originals of any documents used by the Investigator or hospital/institution that allow verification of the existence of the subject and substantiate the integrity of the data collected during the trial.

Source documents should be available to support all the data recorded in the CRF.

16.5. CONFIDENTIALITY

The Investigator must ensure that the subjects' anonymity will be maintained. On the CRFs or other documents submitted to ORION Clinical Services, patients should NOT be identified by their names, but by the assigned patient number and their initials.

If patient names are included on copies of documents submitted to ORION Clinical Services, the names (except for initials) will be obliterated and the assigned patient number added to the document.

The Investigator should keep a separate log (Patient Master List) of patient's codes, names, addresses, telephone numbers and hospital numbers (if applicable). Documents not for submission to ORION Clinical Services (e.g. signed PICDs) should be maintained by the Investigator in strict confidence.

16.6. STAFF INFORMATION & RESPONSIBILITIES

It is the responsibility of the Investigator to ensure that all personnel involved in the study are fully informed of all relevant aspects of the study, including detailed knowledge of and training in all procedures to be followed.

The Investigator will provide a list of delegated responsibility to ORION Clinical Services, detailing the various study tasks to be performed by each member of his/her study staff. Each staff member should sign in agreement to their performing each of the tasks delegated to them on the list. ORION Clinical Services should ensure that the staff have the required knowledge and training for the tasks delegated to them.

16.7. DOCUMENTATION REQUIRED PRIOR TO INITIATION

In addition to the documents mentioned above in Sections 16.1 and 16.2, the following will be required from the Investigator prior to the initiation visit:

- Current, signed and dated *Curriculum Vitae* of Principal Investigator and any Sub-Investigators/co-workers,
- Reference ranges of all laboratory tests to be performed at the study site and a recent certification or accreditation of established quality control (or other documentation of established quality control or external quality assessment or other validation),
- A signed original of the final protocol and any amendments,
- List of delegated responsibility (Investigator Site Staff Signature and Task Delegation Log),
- QP release certificate for Investigational Product to each site.

ORION Clinical Services undertakes to obtain (on behalf of the Sponsor) the necessary approval from the applicable regulatory authority prior to initiation of the study.

16.8. DOCUMENTATION REQUIRED DURING THE STUDY

The following will be required from the Investigator during the study:

- Current, signed and dated *Curriculum Vitae* of Principal Investigator and any Co-Investigators/co-workers who are delegated protocol related responsibilities after study initiation,
- Updates of reference ranges (including the dates from which they become effective) of all laboratory tests to be performed at the study site and updates in certification or accreditation of established quality control (or other documentation of established quality control or external quality assessment or other validation),
- A signed original of any protocol amendments,
- Copies of any approvals from or correspondence with the IEC,
- Renewals of certificates of Insurance, as applicable per country regulations,
- Updated list of delegated responsibility (Investigator Site Staff Signature and Task Delegation Log).

16.9. ESSENTIAL DOCUMENT RETENTION

The Investigator will retain copies of all the essential documents (as defined by ICH GCP[3]) until at least 2 years after the last approval of a marketing application in an ICH region, and until there are no pending or contemplated marketing applications in an ICH region, or at least 2 years have elapsed since the formal discontinuation of clinical development of the investigational product. These documents should be retained for a longer period, however, if required by the applicable regulatory requirements (e.g. EEC Directive 91/507 requires retention of patient codes for at least 15 years after the completion or discontinuation of a trial and retention of hospital records and other source data for the maximum time permitted by the institution where the study takes place). The Investigator should take measures to prevent accidental or premature destruction of these documents.

The essential documents include: the signed protocol, copies of the completed CRFs, signed PICDs from all patients who consented, hospital records, diary cards and other source documents, IEC approval and all related correspondence, including approved documents, and all other documentation included in the Investigator Site File and Dispensing File.

The Investigator will inform the Sponsor of the storage location of these essential documents and must contact the Sponsor before disposing of any. If the Investigator wishes to assign the files to someone else (e.g. if he/she retires) or to remove them to another location, the Sponsor Project Manager should be consulted about this change.

ORION Clinical Services will inform the Investigator in writing when these documents no longer need to be retained.

17. STUDY MANAGEMENT

17.1. MONITORING

Prior to study commencement, the Investigator will be informed of the anticipated frequency of the monitoring visits. He/she will also receive a notification prior to each monitoring visit during the course of the study. It is expected that the Investigator and/or his/her sub-Investigator(s) and other appropriate staff will be available on the day of the visit to discuss study conduct. ORION Clinical Services is responsible for ensuring the proper conduct of the clinical trial with regards protocol adherence and validity of the data recorded in the CRFs.

17.2. QUALITY ASSURANCE AND QUALITY CONTROL

An independent audit of the study may be conducted during the study or after completion. The audit may be conducted by either ORION Clinical Services' or the Sponsor's QA department or an independent auditor or a regulatory authority.

17.2.1. Quality Control

Quality Control is defined as the operational techniques and activities undertaken within the quality assurance system to verify that the requirements for quality of the trial-related activities have been fulfilled.

Quality Control should be applied to each stage of data handling to ensure that all data are reliable and have been processed correctly.

17.2.2. Quality Assurance

Quality Assurance is defined as the planned and systematic actions that are established to ensure that the trial is performed and the data are generated, documented (recorded) and reported in compliance with Good Clinical Practice and the applicable regulatory requirements.

17.2.3. Audit

The Investigator will permit an audit mandated by the sponsor after reasonable notice. The purpose of an audit is to confirm that the study is conducted as per protocol, GCP and applicable regulatory requirements, that the rights and well being of patients enrolled have been protected and that all data relevant for the evaluation of the investigational product captured, processed and reported in compliance with the planned arrangements. The Investigator will permit direct access to all study documents, IP accountability records, medical records and source data. The Investigator and his/her study team will also be available for discussion regarding study progress and procedures during the audit (both during the audit and at the end of the audit for an "exit" discussion).

17.3. DATA QUERY PROCESS

Data management of the CRFs will be performed by ORION Clinical Services on behalf of Alfact.

CRFs will be data entered into the study database, and the data will be verified for missing data, inconsistencies, and for any necessary medical clarifications. Queries arising from these checks will be sent to the Investigator for response and signature. All possible attempts should be made by the Investigator to complete and return the signed responses as instructed to ORION Data Management within the requested timeframes. If the Investigator is unsure about the meaning of a query, or what data is required with the response, then he/she should seek clarification from the ORION Clinical Services CRA assigned to his/her centre.

Once all data queries have been resolved, the study will be declared to be “clean”, and the study database will be locked ready for statistical analysis. After clean-file status has been achieved, the Investigator may archive the copies of the CRFs retained at the centre. The original CRFs collected by ORION Clinical Services will be archived by the Sponsor. The data management, data handling, and analysis will be conducted in accordance with good clinical, scientific and data management principles and in compliance with ORION Clinical Services’ Standard Operating Procedures.

17.4. PROTOCOL DEVIATIONS/AMENDMENTS

Any deviation from the protocol that has not been approved by Alfact and the IEC could result in a discontinuation from the study at the centre involved. Any amendment(s) to the protocol must be approved by both Alfact and the IEC which granted the original approval of the study **prior** to their implementation (unless **only** logistical or administrative aspects of the trial are involved).

However, in the event of any medical emergency, the Investigator is free to institute any medical procedure he/she deems appropriate. Such events and procedures must be promptly reported to ORION Clinical Services representatives.

17.5. DISCONTINUATION OF THE STUDY

The DSMB including experts will be created and will review key safety data and can decide at any time to terminate the study.

Additionally, the Sponsor reserves the right to stop the study at any time on the basis of new information regarding safety or efficacy, or if study progress is unsatisfactory, or for other valid administrative reasons. After such a decision is made, the investigator must inform all patients who received study treatment as soon as possible. All delivered study materials must be collected and all CRFs completed to the extent possible.

17.6. PUBLICATIONS

Any manuscript, abstract or other publication or presentation of results or information arising in connection with the study (including ancillary study involving trial patients) must be prepared in conjunction with the study Sponsor and must be submitted to the Sponsor for review and comment at least 45 days prior to submission for publication or presentation.

18. STUDY TIMETABLE

- Projected starting date (first-patient-in [FPI]): July 2010
- Projected number of patients: 60 patients
- Projected completion of patient accrual (last-patient-in [LPI]): July 2011
- Patient study end date (last-patient-last-visit [LPLV]): August 2011

19. REFERENCES

1. Christa, L., M. Felin, et al. (1994). "The human HIP gene, overexpressed in primary liver cancer encodes for a C-type carbohydrate binding protein with lactose binding activity." FEBS Lett 337(1): 114-8.

2. Cox NR & Mohanty SR, Acute Liver Failure, Hospital Physician *July/August 2009*.

3. Hoofnagle JH, Carithers RL Jr, Shapiro C, Ascher N. Fulminant hepatic failure: summary of a workshop. Hepatology. Jan 1995;21(1):240-52.

4. Iovanna, J. L. and J. C. Dagorn (2005). "The multifunctional family of secreted proteins containing a C-type lectin-like domain linked to a short N-terminal peptide." Biochim Biophys Acta 1723(1-3): 8-18.

5. Kondo, T., T. Suda, et al. (1997). "Essential roles of the Fas ligand in the development of hepatitis." Nat Med 3(4): 409-13.

6. Lasserre, C., L. Christa, et al. (1992). "A novel gene (HIP) activated in human primary liver cancer." Cancer Res 52(18): 5089-95.

7. Lee WM, Schiodt FV. Fulminant hepatic failure. In: Schiff ER, Sorrell, MF, Maddrey WC, eds. Schiff's Diseases of the Liver. 8th ed. Baltimore, Md: Lippincott Williams & Wilkins; 1999.

8. Lee, W. M., R. H. Squires, Jr., et al. (2008). "Acute liver failure: Summary of a workshop." Hepatology **47**(4): 1401-15.

9. Lieu, H. T., F. Batteux, et al. (2005). "HIP/PAP accelerates liver regeneration and protects against acetaminophen injury in mice." Hepatology 42(3): 618-26.

10. Lieu, H. T., M. T. Simon, et al. (2006). "Reg2 inactivation increases sensitivity to Fas hepatotoxicity and delays liver regeneration post-hepatectomy in mice." Hepatology 44(6): 1452-64.

11. Michalopoulos, G. K. and M. C. DeFrances (1997). "Liver regeneration." Science 276(5309): 60-6.

12. Polson, J. and W. M. Lee (2005). "AASLD position paper: the management of acute liver failure." Hepatology **41**(5): 1179-97.

13. Samuel, D. (2008). Hopital Paul Brousse, Villejuif, France , Personnal Communication.

14. Simon, M. T., A. Pauloin, et al. (2003). "HIP/PAP stimulates liver regeneration after partial hepatectomy and combines mitogenic and anti-apoptotic functions through the PKA signaling pathway." FASEB J 17(11): 1441-50.

1 Clinical safety data management: Definitions and Standards for Expedited Reporting (E2A - Approved by the CPMP in November 1994 for studies commencing after 1st June 1995 [Note for Guidance 3C614A]; Approved by FDA with effective date 1st March 1995 [60 FR 11284]).

2 Declaration of Helsinki (as adopted by the 18th World Medical Assembly, 1964 and as revised by the 29th World Medical Assembly, Tokyo 1975, the 35th World Medical Assembly, Venice 1983, the 41st World Medical Assembly, Hong Kong 1989, and the 48th General Assembly, Somerset West, Republic of South Africa, 1996).

3 Declaration of Helsinki (as adopted by the 18th World Medical Assembly, 1964 and as revised by the 29th World Medical Assembly, Tokyo 1975, the 35th World Medical Assembly, Venice 1983, the 41st World Medical Assembly, Hong Kong 1989, and the 48th General Assembly, Somerset West, Republic of South Africa, 1996, 52nd WMA General Assembly, Edinburgh, Scotland, October 2000).

4 ICH Harmonised Tripartite Guideline for Good Clinical Practice (June 1996), as recommended for adoption to the three regulatory parties to ICH. Transmitted to the CPMP in July 1996 and approved on 17th July 1996 (CPMP/ICH/135/95). The proposed date for coming into operation is (for studies commencing after) 17th January 1997. Approved by FDA on 9th May 1997 and effective 9th May 1997 (62 FR 25692).

20. APPENDICES

Appendix A: World Medical Association Declaration of Helsinki (2008 version)

Ethical Principles for Medical Research Involving Human Subjects

Adopted by the 18th World Medical Assembly, Helsinki, Finland. 1964, and amended by the 29th World Medical Assembly, Tokyo, Japan, 1975; 35th World Medical Assembly, Venice, Italy, 1983; 41st World Medical Assembly, Hong Kong, 1989; and the 48th General Assembly, Somerset West, Republic of South Africa, 1996; 52nd WMA General Assembly, Edinburgh, Scotland, October 2000; 53th WMA General Assembly, Washington 2002 (Note of Clarification on paragraph 29 added); 55th WMA General Assembly, Tokyo 2004 (Note of Clarification on Paragraph 30 added); 59th WMA General Assembly, Seoul, October 2008

A. INTRODUCTION

1. The World Medical Association (WMA) has developed the Declaration of Helsinki as a statement of ethical principles for medical research involving human subjects, including research on identifiable human material and data.

   The Declaration is intended to be read as a whole and each of its constituent paragraphs should not be applied without consideration of all other relevant paragraphs.

2. Although the Declaration is addressed primarily to physicians, the WMA encourages other participants in medical research involving human subjects to adopt these principles.

3. It is the duty of the physician to promote and safeguard the health of patients, including those who are involved in medical research. The physician's knowledge and conscience are dedicated to the fulfilment of this duty.

4. The Declaration of Geneva of the WMA binds the physician with the words, “The health of my patient will be my first consideration,” and the International Code of Medical Ethics declares that, “A physician shall act in the patient's best interest when providing medical care.”

5. Medical progress is based on research that ultimately must include studies involving human subjects. Populations that are underrepresented in medical research should be provided appropriate access to participation in research.

6. In medical research involving human subjects, the well-being of the individual research subject must take precedence over all other interests.

7. The primary purpose of medical research involving human subjects is to understand the causes, development and effects of diseases and improve preventive, diagnostic and therapeutic interventions (methods, procedures and treatments). Even the best current interventions must be evaluated continually through research for their safety, effectiveness, efficiency, accessibility and quality.

8. In medical practice and in medical research, most interventions involve risks and burdens.

9. Medical research is subject to ethical standards that promote respect for all human subjects and protect their health and rights. Some research populations are particularly vulnerable and need special protection. These include those who cannot give or refuse consent for themselves and those who may be vulnerable to coercion or undue influence.

10. Physicians should consider the ethical, legal and regulatory norms and standards for research involving human subjects in their own countries as well as applicable international norms and standards. No national or international ethical, legal or regulatory requirement should reduce or eliminate any of the protections for research subjects set forth in this Declaration.

B. PRINCIPLES FOR ALL MEDICAL RESEARCH

11. It is the duty of physicians who participate in medical research to protect the life, health, dignity, integrity, right to self-determination, privacy, and confidentiality of personal information of research subjects.

12. Medical research involving human subjects must conform to generally accepted scientific principles, be based on a thorough knowledge of the scientific literature, other relevant sources of information, and adequate laboratory and, as appropriate, animal experimentation. The welfare of animals used for research must be respected.

13. Appropriate caution must be exercised in the conduct of medical research that may harm the environment.

14. The design and performance of each research study involving human subjects must be clearly described in a research protocol. The protocol should contain a statement of the ethical considerations involved and should indicate how the principles in this Declaration have been addressed. The protocol should include information regarding funding, sponsors, institutional affiliations, other potential conflicts of interest, incentives for subjects and provisions for treating and/or compensating subjects who are harmed as a consequence of participation in the research study. The protocol should describe arrangements for post-study access by study subjects to interventions identified as beneficial in the study or access to other appropriate care or benefits.

15. The research protocol must be submitted for consideration, comment, guidance and approval to a research ethics committee before the study begins. This committee must be independent of the researcher, the sponsor and any other undue influence. It must take into consideration the laws and regulations of the country or countries in which the research is to be performed as well as applicable international norms and standards but these must not be allowed to reduce or eliminate any of the protections for research subjects set forth in this Declaration. The committee must have the right to monitor ongoing studies. The researcher must provide monitoring information to the committee, especially information about any serious adverse events. No change to the protocol may be made without consideration and approval by the committee.

Appendix A: World Medical Association Declaration of Helsinki (2008 version) CONTINUED

16. Medical research involving human subjects must be conducted only by individuals with volunteers requires the supervision of a competent and appropriately qualified physician or other health care professional. The responsibility for the protection of research subjects must always rest with the physician or other health care professional and never the research subjects, even though they have given consent.

17. Medical research involving a disadvantaged or vulnerable population or community is only justified if the research is responsive to the health needs and priorities of this population or community and if there is a reasonable likelihood that this population or community stands to benefit from the results of the research.

18. Every medical research study involving human subjects must be preceded by careful assessment of predictable risks and burdens to the individuals and communities involved in the research in comparison with foreseeable benefits to them and to other individuals or communities affected by the condition under investigation.

19. Every clinical trial must be registered in a publicly accessible database before recruitment of the first subject.

20. Physicians may not participate in a research study involving human subjects unless they are confident that the risks involved have been adequately assessed and can be satisfactorily managed. Physicians must immediately stop a study when the risks are found to outweigh the potential benefits or when there is conclusive proof of positive and beneficial results.

21. Medical research involving human subjects may only be conducted if the importance of the objective outweighs the inherent risks and burdens to the research subjects.

22. Participation by competent individuals as subjects in medical research must be voluntary. Although it may be appropriate to consult family members or community leaders, no competent individual may be enrolled in a research study unless he or she freely agrees.

23. Every precaution must be taken to protect the privacy of research subjects and the confidentiality of their personal information and to minimize the impact of the study on their physical, mental and social integrity.

24. In medical research involving competent human subjects, each potential subject must be adequately informed of the aims, methods, sources of funding, any possible conflicts of interest, institutional affiliations of the researcher, the anticipated benefits and potential risks of the study and the discomfort it may entail, and any other relevant aspects of the study. The potential subject must be informed of the right to refuse to participate in the study or to withdraw consent to participate at any time without reprisal. Special attention should be given to the specific information needs of individual potential subjects as well as to the methods used to deliver the information. After ensuring that the potential subject has understood the information, the physician or another appropriately qualified individual must then seek the potential subject's freely-given informed consent, preferably in writing. If the consent cannot be expressed in writing, the non-written consent must be formally documented and witnessed.

25. For medical research using identifiable human material or data, physicians must normally seek consent for the collection, analysis, storage and/or reuse. There may be situations where consent would be impossible or impractical to obtain for such research or would pose a threat to the validity of the research. In such situations the research may be done only after consideration and approval of a research ethics committee.

26. When seeking informed consent for participation in a research study the physician should be particularly cautious if the potential subject is in a dependent relationship with the physician or may consent under duress. In such situations the informed consent should be sought by an appropriately qualified individual who is completely independent of this relationship.

27. For a potential research subject who is incompetent, the physician must seek informed consent from the legally authorized representative. These individuals must not be included in a research study that has no likelihood of benefit for them unless it is intended to promote the health of the population represented by the potential subject, the research cannot instead be performed with competent persons, and the research entails only minimal risk and minimal burden.

28. When a potential research subject who is deemed incompetent is able to give assent to decisions about participation in research, the physician must seek that assent in addition to the consent of the legally authorized representative. The potential subject's dissent should be respected.

29. Research involving subjects who are physically or mentally incapable of giving consent, for example, unconscious patients, may be done only if the physical or mental condition that prevents giving informed consent is a necessary characteristic of the research population. In such circumstances the physician should seek informed consent from the legally authorized representative. If no such representative is available and if the research cannot be delayed, the study may proceed without informed consent provided that the specific reasons for involving subjects with a condition that renders them unable to give informed consent have been stated in the research protocol and the study has been approved by a research ethics committee. Consent to remain in the research should be obtained as soon as possible from the subject or a legally authorized representative.

30. Authors, editors and publishers all have ethical obligations with regard to the publication of the results of research. Authors have a duty to make publicly available the results of their research on human subjects and are accountable for the completeness and accuracy of their reports. They should adhere to accepted guidelines for ethical reporting. Negative and inconclusive as well as positive results should be published or otherwise made publicly available. Sources of funding, institutional affiliations and conflicts of interest should be declared in the publication. Reports of research not in accordance with the principles of this Declaration should not be accepted for publication.

Appendix A: World Medical Association Declaration of Helsinki (2008 version) CONTINUED

C. ADDITIONAL PRINCIPLES FOR MEDICAL RESEARCH COMBINED WITH MEDICAL CARE

31. The physician may combine medical research with medical care only to the extent that the research is justified by its potential preventive, diagnostic or therapeutic value and if the physician has good reason to believe that participation in the research study will not adversely affect the health of the patients who serve as research subjects.

32. The benefits, risks, burdens and effectiveness of a new intervention must be tested against those of the best current proven intervention, except in the following circumstances:

- The use of placebo, or no treatment, is acceptable in studies where no current proven intervention exists; or
- Where for compelling and scientifically sound methodological reasons the use of placebo is necessary to determine the efficacy or safety of an intervention and the patients who receive placebo or no treatment will not be subject to any risk of serious or irreversible harm. Extreme care must be taken to avoid abuse of this option.

33. At the conclusion of the study, patients entered into the study are entitled to be informed about the outcome of the study and to share any benefits that result from it, for example, access to interventions identified as beneficial in the study or to other appropriate care or benefits.

34. The physician must fully inform the patient which aspects of the care are related to the research. The refusal of a patient to participate in a study or the patient’s decision to withdraw from the study must never interfere with the patient-physician relationship.

35. In the treatment of a patient, where proven interventions do not exist or have been ineffective, the physician, after seeking expert advice, with informed consent from the patient or a legally authorized representative, may use an unproven intervention if in the physician's judgement it offers hope of saving life, re-establishing health or alleviating suffering. Where possible, this intervention should be made the object of research, designed to evaluate its safety and efficacy. In all cases, new information should be recorded and, where appropriate, made publicly available.

Appendix B: ICH-GCP – Investigator's responsibilities

ICH Harmonised Tripartite Guideline for Good Clinical Practice (June 1996), as recommended for adoption to the three regulatory parties to ICH. Transmitted to the CPMP in July 1996 and approved on 17th July 1996 (CPMP/ICH/135/95). The proposed date for coming into operation is (for studies commencing after) 17th January 1997. Approved by FDA on 9th May 1997 and effective 9th May 1997 (62 FR 25692).

4. INVESTIGATOR

4.1 Investigator's Qualifications and Agreements

4.1.1 The investigator(s) should be qualified by education, training, and experience to assume responsibility for the proper conduct of the trial, should meet all the qualifications specified by the applicable regulatory requirement(s), and should provide evidence of such qualifications through up-to-date curriculum vitae and/or other relevant documentation requested by the sponsor, the IRB/IEC, and/or the regulatory authority(ies).

4.1.2 The investigator should be thoroughly familiar with the appropriate use of the investigational product(s), as described in the protocol, in the current Investigator's Brochure, in the product information and in other information sources provided by the sponsor.

4.1.3 The investigator should be aware of, and should comply with, GCP and the applicable regulatory requirements.

4.1.4 The investigator/institution should permit monitoring and auditing by the sponsor, and inspection by the appropriate regulatory authority(ies).

4.1.5 The investigator should maintain a list of appropriately qualified persons to whom the investigator has delegated significant trial-related duties.

4.2 Adequate Resources

4.2.1 The investigator should be able to demonstrate (e.g. based on retrospective data) a potential for recruiting the required number of suitable subjects within the agreed recruitment period.

4.2.2 The investigator should have sufficient time to properly conduct and complete the trial within the agreed trial period.

4.2.3 The investigator should have available an adequate number of qualified staff and adequate facilities for the foreseen duration of the trial to conduct the trial properly and safely.

4.2.4 The investigator should ensure that all persons assisting with the trial are adequately informed about the protocol, the investigational product(s), and their trial-related duties and functions.

4.3 Medical Care of Trial Subjects

4.3.1 A qualified physician (or dentist, when appropriate), who is an investigator or a sub-investigator for the trial, should be responsible for all trial-related medical (or dental) decisions.

4.3.2 During and following a subject's participation in a trial, the investigator/institution should ensure that adequate medical care is provided to a subject for any adverse events, including clinically significant laboratory values, related to the trial. The investigator/institution should inform a subject when medical care is needed for intercurrent illness(es) of which the investigator becomes aware.

4.3.3 It is recommended that the investigator inform the subject's primary physician about the subject's participation in the trial if the subject has a primary physician and if the subject agrees to the primary physician being informed.

4.3.4 Although a subject is not obliged to give his/her reason(s) for withdrawing prematurely from a trial, the investigator should make a reasonable effort to ascertain the reason(s), while fully respecting the subject's rights.

4.4 Communication with IRB/IEC

4.4.1 Before initiating a trial, the investigator/institution should have written and dated approval/favourable opinion from the IRB/IEC for the trial protocol, written informed consent form, consent form updates, subject recruitment procedures (e.g. advertisements), and any other written information to be provided to subjects.

Appendix B: ICH-GCP – Investigator's responsibilities

4.4.2 As part of the investigator's/institution's written application to the IRB/IEC, the investigator/institution should provide the IRB/IEC with a current copy of the Investigator's Brochure. If the Investigator's Brochure is updated during the trial, the investigator/institution should supply a copy of the updated Investigator's Brochure to the IRB/IEC.

4.4.3 During the trial the investigator/institution should provide to the IRB/IEC all documents subject to review.

4.5 Compliance with Protocol

4.5.1 The investigator/institution should conduct the trial in compliance with the protocol agreed to by the sponsor and, if required, by the regulatory authority(ies) and which was given approval/favourable opinion by the IRB/IEC. The investigator/institution and the sponsor should sign the protocol, or an alternative contract, to confirm agreement.

4.5.2 The investigator should not implement any deviation from, or changes of the protocol without agreement by the sponsor and prior review and documented approval/favourable opinion from the IRB/IEC of an amendment, except where necessary to eliminate an immediate hazard(s) to trial subjects, or when the change(s) involves only logistical or administrative aspects of the trial (e.g. change in monitor(s), change of telephone number(s)).

4.5.3 The investigator, or person designated by the investigator, should document and explain any deviation from the approved protocol.

4.5.4 The investigator may implement a deviation from, or a change of, the protocol to eliminate an immediate hazard(s) to trial subjects without prior IRB/IEC approval/favourable opinion. As soon as possible, the implemented deviation or change, the reasons for it, and, if appropriate, the proposed protocol amendments should be submitted:

- to the IRB/IEC for review and approval/favourable opinion,
- to the sponsor for agreement and, if required,
- to the regulatory authority(ies).

4.6 Investigational Product(s)

4.6.1 Responsibility for investigational product(s) accountability at the trial site(s) rests with the investigator/institution.

4.6.2 Where allowed/required, the investigator/institution may/should assign some or all of the investigator's/institution's duties for investigational product(s) accountability at the trial site(s) to an appropriate pharmacist or another appropriate individual who is under the supervision of the investigator/institution.

4.6.3 The investigator/institution and/or a pharmacist or other appropriate individual, who is designated by the investigator/institution, should maintain records of the product's delivery to the trial site, the inventory at the site, the use by each subject, and the return to the sponsor or alternative disposition of unused product(s). These records should include dates, quantities, batch/serial numbers, expiration dates (if applicable), and the unique code numbers assigned to the investigational product(s) and trial subjects. Investigators should maintain records that document adequately that the subjects were provided the doses specified by the protocol and reconcile all investigational product(s) received from the sponsor.

4.6.4 The investigational product(s) should be stored as specified by the sponsor (see 5.13.2 and 5.14.3) and in accordance with applicable regulatory requirement(s).

4.6.5 The investigator should ensure that the investigational product(s) are used only in accordance with the approved protocol.

4.6.6 The investigator, or a person designated by the investigator/institution, should explain the correct use of the investigational product(s) to each subject and should check, at intervals appropriate for the trial, that each subject is following the instructions properly.

Appendix B: ICH-GCP – Investigator's responsibilities

4.7 RANDOMISATION PROCEDURES AND UNBLINDING

The investigator should follow the trial's randomisation procedures, if any, and should ensure that the code is broken only in accordance with the protocol. If the trial is blinded, the investigator should promptly document and explain to the sponsor any premature unblinding (e.g. accidental unblinding, unblinding due to a serious adverse event) of the investigational product(s).

4.8 Informed Consent of Trial Subjects

4.8.1 In obtaining and documenting informed consent, the investigator should comply with the applicable regulatory requirement(s), and should adhere to GCP and to the ethical principles that have their origin in the Declaration of Helsinki. Prior to the beginning of the trial, the investigator should have the IRB/IEC's written approval/favourable opinion of the written informed consent form and any other written information to be provided to subjects.

4.8.2 The written informed consent form and any other written information to be provided to subjects should be revised whenever important new information becomes available that may be relevant to the subject's consent. Any revised written informed consent form, and written information should receive the IRB/IEC's approval/favourable opinion in advance of use. The subject or the subject's legally acceptable representative should be informed in a timely manner if new information becomes available that may be relevant to the subject's willingness to continue participation in the trial. The communication of this information should be documented.

4.8.3 Neither the investigator, nor the trial staff, should coerce or unduly influence a subject to participate or to continue to participate in a trial.

4.8.4 None of the oral and written information concerning the trial, including the written informed consent form, should contain any language that causes the subject or the subject's legally acceptable representative to waive or to appear to waive any legal rights, or that releases or appears to release the investigator, the institution, the sponsor, or their agents from liability for negligence.

4.8.5 The investigator, or a person designated by the investigator, should fully inform the subject or, if the subject is unable to provide informed consent, the subject's legally acceptable representative, of all pertinent aspects of the trial including the written information given approval/favourable opinion by the IRB/IEC.

4.8.6 The language used in the oral and written information about the trial, including, the written informed consent form, should be as non-technical as practical and should be understandable to the subject or the subject's legally acceptable representative and the impartial witness, where applicable.

4.8.7 Before informed consent may be obtained, the investigator, or a person designated by the investigator, should provide the subject or the subject's legally acceptable representative ample time and opportunity to inquire about details of the trial and to decide whether or not to participate in the trial. All questions about the trial should be answered to the satisfaction of the subject or the subject's legally acceptable representative.

4.8.8 Prior to a subject's participation in the trial, the written informed consent form should be signed and personally dated by the subject or by the subject's legally acceptable representative, and by the person who conducted the informed consent discussion.

4.8.9 If a subject is unable to read or if a legally acceptable representative is unable to read, an impartial witness should be present during the entire informed consent discussion. After the written informed consent form and any other written information to be provided to subjects, is read and explained to the subject or the subject's legally acceptable representative, and after the subject or the subject's legally acceptable representative has orally consented to the subject's participation in the trial and, if capable of doing so, has signed and personally dated the informed consent form, the witness should sign and personally date the consent form. By signing the consent form, the witness attests that the information in the consent form and any other written information was accurately explained to, and apparently understood by, the subject or the subject's legally acceptable representative, and that informed consent was freely given by the subject or the subject's legally acceptable representative.

Appendix B: ICH-GCP – Investigator's responsibilities

4.8.10 Both the informed consent discussion and the written informed consent form and any other written information to be provided to subjects should include explanations of the following:

a) That the trial involves research.

b) The purpose of the trial.

c) The trial treatment(s) and the probability for random assignment to each treatment.

d) The trial procedures to be followed, including all invasive procedures.

e) The subject's responsibilities.

f) Those aspects of the trial that are experimental.

g) The reasonably foreseeable risks or inconveniences to the subject and, when applicable, to an embryo, foetus, or nursing infant.

h) The reasonably expected benefits. When there is no intended clinical benefit to the subject, the subject should be made aware of' this.

i) The alternative procedure(s) or course(s) of treatment that may be available to the subject, and their important potential benefits and risks.

j) The compensation and/or treatment available to the subject in the event of trial-related injury.

k) The anticipated prorated payment, if any, to the subject for participating in the trial.

l) The anticipated expenses, if any, to the subject for participating in the trial.

m) That the subject's participation in the trial is voluntary and that the subject may refuse to participate or withdraw from the trial, at any time, without penalty or loss of benefits to which the subject is otherwise entitled.

n) That the monitor(s), the auditor(s), the IRB/IEC, and the regulatory authority(ies) will be granted direct access to the subject's original medical records for verification of clinical trial procedures and/or data, without violating the confidentiality of the subject, to the extent permitted by the applicable laws and regulations and that, by signing a written informed consent form, the subject or the subject's legally acceptable representative is authorising such access.

o) That records identifying the subject will be kept confidential and, to the extent permitted by the applicable laws and/or regulations, will not be made publicly available. If the results of the trial are published, the subject's identity will remain confidential.

p) That the subject or the subject's legally acceptable representative will be informed in a timely manner if information becomes available that may be relevant to the subject's willingness to continue participation in the trial.

q) The person(s) to contact for further information regarding the trial and the rights of trial subjects, and whom to contact in the event of trial-related injury.

r) The foreseeable circumstances and/or reasons under which the subject's participation in the trial may be terminated.

s) The expected duration of the subject's participation in the trial.

t) The approximate number of subjects involved in the trial.

4.8.11 Prior to participation in the trial, the subject or the subject's legally acceptable representative should receive a copy of the signed and dated written informed consent form and any other written information provided to the subjects. During a subject's participation in the trial, the subject or the subject's legally acceptable representative should receive a copy of the signed and dated consent form updates and a copy of any amendments to the written information provided to subjects.

Appendix B: ICH-GCP – Investigator's responsibilities

4.8.12 When a clinical trial (therapeutic or non-therapeutic) includes subjects who can only be enrolled in the trial with the consent of the subject's legally acceptable representative (e.g. minors, or patients with severe dementia), the subject should be informed about the trial to the extent compatible with the subject's understanding and, if capable, the subject should sign and personally date the written informed consent.

4.8.13 Except as described in 4.8.14, a non-therapeutic trial (i.e. a trial in which there is no anticipated direct clinical benefit to the subject), should be conducted in subjects who personally give consent and who sign and date the written informed consent form.

4.8.14 Non-therapeutic trials may be conducted in subjects with consent of a legally acceptable representative provided the following conditions are fulfilled:

a) The objectives of the trial can not be met by means of a trial in subjects who can give informed consent personally.

b) The foreseeable risks to the subjects are low.

c) The negative impact on the subject's well-being is minimised and low.

d) The trial is not prohibited by law.

e) The approval/favourable opinion of the IRB/IEC is expressly sought on the inclusion of such subjects, and the written approval/favourable opinion covers this aspect.

f) Such trials, unless an exception is justified, should be conducted in patients having a disease or condition for which the investigational product is intended. Subjects in these trials should be particularly closely monitored and should be withdrawn if they appear to be unduly distressed.

4.8.15 In emergency situations, when prior consent of the subject is not possible, the consent of the subject's legally acceptable representative, if present, should be requested. When prior consent of the subject is not possible, and the subject's legally acceptable representative is not available, enrolment of the subject should require measures described in the protocol and/or elsewhere, with documented approval/favourable opinion by the IRB/IEC, to protect the rights, safety and well-being of the subject and to ensure compliance with applicable regulatory requirements. The subject or the subject's legally acceptable representative should be informed about the trial as soon as possible and consent to continue and other consent as appropriate (see 4.8.10) should be requested.

4.9 Records and Reports

4.9.1 The investigator should ensure the accuracy, completeness, legibility, and timeliness of the data reported to the sponsor in the CRFs and in all required reports.

4.9.2 Data reported on the CRF, that are derived from source documents, should be consistent with the source documents or the discrepancies should be explained.

4.9.3 Any change or correction to a CRF should be dated, initialled, and explained (if necessary) and should not obscure the original entry (i.e. an audit trail should be maintained); this applies to both written and electronic changes or corrections (see 5.18.4 (n)). Sponsors should provide guidance to investigators and/or the investigators' designated representatives on making such corrections. Sponsors should have written procedures to assure that changes or corrections in CRFs made by sponsor's designated representatives are documented, are necessary, and are endorsed by the investigator. The investigator should retain records of the changes and corrections.

4.9.4 The investigator/institution should maintain the trial documents as specified in Essential Documents for the Conduct of a Clinical Trial (see 8.) and as required by the applicable regulatory requirement(s). The investigator/institution should take measures to prevent accidental or premature destruction of these documents.

4.9.5 Essential documents should be retained until at least 2 years after the last approval of a marketing application in an ICH region and until there are no pending or contemplated marketing applications in an ICH region or at least 2 years have elapsed since the formal discontinuation of clinical development of the investigational product. These documents should be retained for a longer period however if required by the applicable regulatory requirements or by an agreement with the sponsor. It is the responsibility of the sponsor to inform the investigator/institution as to when these documents no longer need to be retained (see 5.5.12).

Appendix B: ICH-GCP – Investigator's responsibilities

4.9.6 The financial aspects of the trial should be documented in an agreement between the sponsor and the investigator/institution.

4.9.7 Upon request of the monitor, auditor, IRB/IEC, or regulatory authority, the investigator/institution should make available for direct access all requested trial-related records.

4.10 Progress Reports

4.10.1 The investigator should submit written summaries of the trial status to the IRB/IEC annually, or more frequently, if requested by the IRB/IEC.

4.10.2 The investigator should promptly provide written reports to the sponsor, the IRB/IEC (see 3.3.8) and, where applicable, the institution on any changes significantly affecting the conduct of the trial, and/or increasing the risk to subjects.

4.11 Safety Reporting

4.11.1 All serious adverse events (SAEs) should be reported immediately to the sponsor except for those SAEs that the protocol or other document (e.g. Investigator's Brochure) identifies as not needing immediate reporting. The immediate reports should be followed promptly by detailed, written reports. The immediate and follow-up reports should identify subjects by unique code numbers assigned to the trial subjects rather than by the subjects' names, personal identification numbers, and/or addresses. The investigator should also comply with the applicable regulatory requirement(s) related to the reporting of unexpected serious adverse drug reactions to the regulatory authority(ies) and the IRB/IEC.

4.11.2 Adverse events and/or laboratory abnormalities identified in the protocol as critical to safety evaluations should be reported to the sponsor according to the reporting requirements and within the time periods specified by the sponsor in the protocol.

4.11.3 For reported deaths, the investigator should supply the sponsor and the IRB/IEC with any additional requested information (e.g. autopsy reports and terminal medical reports).

4.12 Premature Termination or Suspension of a Trial

If the trial is prematurely terminated or suspended for any reason, the investigator/institution should promptly inform the trial subjects, should assure appropriate therapy and follow-up for the subjects, and, where required by the applicable regulatory requirement(s), should inform the regulatory authority(ies). In addition:

4.12.1 If the investigator terminates or suspends a trial without prior agreement of the sponsor, the investigator should inform the institution where applicable, and the investigator/institution should promptly inform the sponsor and the IRB/IEC, and should provide the sponsor and the IRB/IEC a detailed written explanation of the termination or suspension.

4.12.2 If the sponsor terminates or suspends a trial (see 5.21), the investigator should promptly inform the institution where applicable and the investigator/institution should promptly inform the IRB/IEC and provide the IRB/IEC a detailed written explanation of the termination or suspension.

4.12.3 If the IRB/IEC terminates or suspends its approval/favourable opinion of a trial (see 3.1.2 and 3.3.9), the investigator should inform the institution where applicable and the investigator/institution should promptly notify the sponsor and provide the sponsor with a detailed written explanation of the termination or suspension.

4.13 Final Report(s) by Investigator

Upon completion of the trial, the investigator, where applicable, should inform the institution; the investigator/institution should provide the IRB/IEC with a summary of' the trial's outcome, and the regulatory authority(ies) with any reports required.

Appendix C: Study Flow Chart

Study assessment & exams	Baseline		H0 ± 10 min	H12 ± 10 min	H24 ± 10 min	H36 ± 10 min	H48 ± 10 min	H60 ± 10 min	H72 ± 10 min	H84 ± 10 min	D8 (after H0) +2 days	D21 (after H0) +2 days	Premature Discont.
Informed consent signed	X	RANDOMIZATION											
Check Incl./excl. Criteria	X												
Medical history	X												
Demographics (age, gender)	X												
Weight, height & BMI	X												
Viral serologies and/or PCR (HAV, HBV, HCV, HDV, HEV, HSV,VZV, HIV)	X												
Acetaminophen, alcohol plasma levels	X												
Cupremia, ceruloplasmin plasma levels, and cupruria	X												
Autoimmune markers (ANA, ASMA, anti-LKM Ab, Ig levels)	X												
Beta hCG blood pregnancy test for women of childbearing potential only	X											X	X
ALF-5755/ Placebo infusions			Within 12 hrs after baseline	X	X	X	X	X					
Clinical examination	X		Before, 10 min after infusion	Before, 10 min after infusion	Before, 10 min after infusion	Before, 10 min after infusion	Before, 10 min after infusion	Before, 10 min after infusion	X	X	X	X	X
Vital signs (BP, HR, RR)	X		Before infusion, 10 min + HR and BP only during 1 hr after infusion	Before infusion, 10 min + HR and BP only during 1 hr after infusion	Before infusion, 10 min + HR and BP only during 1 hr after infusion	Before infusion, 10 min + HR and BP only during 1 hr after infusion	Before infusion, 10 min + HR and BP only during 1 hr after infusion	Before infusion, 10 min + HR and BP only during 1 hr after infusion	X	X	X	X	X
Body temperature	X		Before infusion	Before infusion	Before infusion	Before infusion	Before infusion	Before infusion	X	X	X	X	X
ECG	X		Before, 10 min and 1 hr after infusion		Before and 10 min after infusion		Before and 10 min after infusion		X	X	X	X	X
Hepatic Encephalopathy grade & GCS	X		Before infusion	Before infusion	Before infusion	Before infusion	Before infusion	Before infusion	X	X	X	X	X
Arterial blood gas with lactates: if grade I or II hepatic encephalopathy or GCS < 15													
PR, FV, INR, ALT, AST	X		Before infusion	Before infusion	Before infusion	Before infusion	Before infusion	Before infusion	X	X	X	X	X
Haematology, Biochemistry	X		Before infusion	Before infusion	Before infusion	Before infusion	Before infusion	Before infusion	X	X	X	X	X
Urinalysis			Before infusion		Before infusion		Before infusion		X		X	X	X
Anti-ALF-5755 antibodies			Before infusion								X	X	X
Concomitant medications & AE recording (from signed PICD)	X		X	X	X	X	X	X	X	X	X	X	X
CK18			Before infusion	Before infusion	Before infusion	Before infusion	Before infusion	Before infusion	X	X			X

Appendix D: Pharmacokinetics Schedule

ALF-5755 plasma concentrations will be performed in the first 16 patients included at Paul Brousse Hospital and Beaujon Hospital at the following times, i.e. 8 patients per centre:

**H0**	**H0 10 min**	**H1**	**H2**	**H6**	**H12**	**H48**	**H60**	**H60 10 min**	**H61**	**H62**	**H66**	**H72**	**H84**	**Premature discont.**
X<br>just before 1st infusion	X<br>end of 1st infusion	X	X	X	X<br>just before 2nd infusion	X<br>just before 5th infusion	X<br>just before 6th infusion	X<br>end of 6th infusion	X	X	X	X	X	X

Appendix E: Grades of hepatic encephalopathy

**Grade**	**Neurological signs**
0	None
I	Slowness of mentation
II	Drowsiness and presence of asterixis
III	Marked confusion with disorientation or reaction only to vocal stimuli
IV	Deep coma without reaction to vocal stimuli

References

Bismuth H, Didier S, Bastaing D, et al. Orthotopic liver transplantation in fulminant and subfulminant hepatitis: the Paul Brousse experience. Ann Surg 1995; 222:109-119.

Trey C, Davidson CS. The management of fulminant hepatic failure. In Popper H, Shaffner F, eds. Progress in Liver Diseases. New York: Grune and Stratton, 1970, pp 282-298.

Appendix F: Glasgow Coma Scale

Eye Opening Response	Verbal Response	Motor Response
- Spontaneous--open with blinking at baseline: **4 points**<br>- To verbal stimuli, command, speech: **3 points**<br>- To pain only (not applied to face): **2 points**<br>- No response: **1 point**	- Oriented: **5 points**<br>- Confused conversation, but able to answer questions: **4 points**<br>- Inappropriate words: **3 points**<br>- Incomprehensible speech: **2 points**<br>- No response: **1 point**	- Obeys commands for movement: **6 points**<br>- Purposeful movement to painful stimulus: **5 points**<br>- Withdraws in response to pain: **4 points**<br>- Flexion in response to pain (decorticate posturing): **3 points**<br>- Extension response in response to pain (decerebrate posturing): **2 points**<br>- No response: **1 point**

References

Teasdale G, Jennett B. Assessment of coma and impaired consciousness. Lancet 1974; 81-84.
Teasdale G, Jennett B. Assessment and prognosis of coma after head injury. Acta Neurochir 1976; 34:45-55.
Teasdale G. et al. Adding up the Glasgow Coma Score. Acta Neurochir. Suppl. 1979;28:13-6.
