# Supplementary material for: A Proof of Concept, Phase II Randomized European Trial, on the Efficacy of ALF-5755, a Novel Extracellular Matrix-Targeted Antioxidant in Patients with Acute Liver Diseases
Source: PLoS One. 2016 Mar 16;11(3):e0150733. doi: 10.1371/journal.pone.0150733 (PMC4794150; doi:10.1371/journal.pone.0150733)
Supplement: S2 Text — (PDF) [file pone.0150733.s004.pdf]

afssaps
Agence française de sécurité sanitaire des produits de santé

AUTORISATION D'ESSAI CLINIQUE DE MEDICAMENT A USAGE HUMAIN

Nombre de pages : 1
(incluant la page de garde)

Envoi par Télécopie

Date : 0 9 JUIL. 2010

Identifiants de l'essai clinique					
Titre	A multicentre, double-blind, randomized, placebo-controlled study to evaluate the efficacy and the safety of ALF-5755 in patients with nonacetaminophen severe acute hepatitis and early stage acute liver failure				
Promoteur	Alfact Innovation			Réf. CPP	Non disponible
Réf. Promoteur	ALF-5755_P2_ALF	N° EudraCT	2010-020657-14	Réf. Afssaps	**A100518-24**

Expéditeur
AFSSAPS / DEMEB / Département de l'évaluation des essais cliniques et des médicaments à statut particulier<br>**Unité essais cliniques médicaments et hors produits de santé**
Dossier suivi par : Stéphanie VALLET<br>Tél : 33 (0) 1 55 87 36 41 / Fax : 33 (0) 1 55 87 36 42

Destinataire (demandeur : nom / société / tél.)	
Amit SINGLA<br>ORION Clinical Services Limited<br>+44 17 53 57 80 80	
Fax	**+44 17 53 57 80 81**

CPP destinataire en copie	Ile-de-France VII (Le Kremlin-Bicêtre)	Fax	01.45.21.21.45	Code 31

Vu le code de la santé publique et notamment ses articles L. 1123-8, L. 1123-12 et vu le dossier de demande d'autorisation d'essai clinique adressé à l'Agence française de sécurité sanitaire des produits de santé (Afssaps) ;

Vu les compléments versés par le promoteur en dates des 28 juin et 8 juillet 2010 et notamment le protocole de l'essai cité en objet modifié (addendum 1 daté du 24 juin 2010, addendum 2 daté du 28 juin 2010), suite à la demande de l'Afssaps ;

Vu l'engagement du promoteur reçu par courriels les 28 juin et 8 juillet 2010 visant à :
- placer les prochains lots de substance active en stabilité suivant le même protocole que celui décrit pour la substance active dans le tampon « formulation buffer 2 » ;
- modifier, pour les prochains lots d'ALF-5755, la limite d'acceptation de la charge microbiologique avant filtration stérilisante du produit fini pour être en accord avec les lignes directrices européennes CPMP/QWP/486/95, soit ≤10CFU/100 mL

L'autorisation mentionnée à l'article L. 1123-8 du code de la santé publique est accordée pour l'essai clinique cité en objet sous réserve du respect de l'engagement sus visé. Cette autorisation est valable pour toute la durée de l'essai à compter de la date de la présente décision.

Toutefois, conformément à l'article R. 1123-33 du code de la santé publique, la présente autorisation devient caduque si la recherche n'a pas débuté dans un délai d'un an.

Pour le Directeur Général et par délégation
Dr C. BELORGEY-BISMUT
Chef du Dpt de l'Evaluation des Essais Cliniques et des Médicaments à Statut Particulier

0 9 JUIL. 2010

Si vous ne recevez pas toutes les pages de cette télécopie, veuillez contacter le secrétariat de Unité essais cliniques médicaments et hors produits de santé au : 33 (0) 1 55 87 36 41.

Confidentialité
Cette transmission est à l'attention exclusive du(des) destinataires ci-dessus mentionné(s) et peut contenir des informations privilégiées et/ou confidentielles. Si vous n'êtes pas le destinataire voulu ou une personne mandatée pour lui remettre cette transmission, vous avez reçu ce document par erreur et toute utilisation, révélation, copie ou communication de son contenu est interdite. Si vous avez reçu cette transmission par erreur, veuillez nous en informer par téléphone immédiatement et nous retourner le message original par courrier. Merci.

Confidentiality
This transmission is intended to the addressee(s) listed above only and may contain preferential or/and confidential information. If you are not the intended recipient, you are hereby notified that you have received the document by mistake and any use, disclosure, copying or communication of the content of this transmission is prohibited. If you have received this transmission by mistake, please call us immediately and return the original message by mail. Thank you.

afssaps

Agence française de sécurité sanitaire des produits de santé

REPUBLIQUE FRANÇAISE

ATTESTATION EN VUE D'UNE IMPORTATION DE MEDICAMENTS NECESSAIRES A LA REALISATION D'UNE RECHERCHE BIOMEDICALE – AAI 160708

ENVOI PAR TELECOPIE

Nombre de pages : 1
(incluant la page de garde)

**Expéditeur**	DEMEB / Département de l'évaluation des essais cliniques et des médicaments à statut particulier<br>**Unité essais cliniques médicaments et hors produits de santé**

**Destinataire** (Demandeur) Nom adresse-téléphone	Matthew Davis<br>ORION Clinical Services Ltd<br>7 Bath Road<br>Slough<br>Berkshire<br>SL1 3UA<br><br>+44 1753578081	**N° de télécopie**	+44 1753578081

Les médicaments listés ci-après, nécessaires à la réalisation de la recherche biomédicale ci-dessous référencée, peuvent être importés sur la base de la présente attestation à présenter lors du contrôle par les agents des douanes. Cette attestation est délivrée conformément aux articles L. 5124-13, R. 5121-108 et R. 5121-114 du code de la santé publique. Elle n'est valable que pour des médicaments utilisés que dans le cadre de cette recherche, autorisée par l'Afssaps en vertu de l'article L. 1123-8 du code précité, et pendant toute la durée de celle-ci.

REFERENCES DE LA RECHERCHE BIOMEDICALE AUTORISEE PAR L'AFSSAPS			
Titre	**A multicentre, double-blind, randomised, placebo-controlled study to evaluate the efficacy and the safety of ALF-5755 in patients with nonacetaminophen severe acute hepatitis and early stage acute liver failure**		
Promoteur	**Alfact Innovation**	N° EudraCT	**2010-020657-14**

LISTE DES MEDICAMENTS IMPORTES[1] [2]

**Nom du médicament [3]**	**Substance active [3]**	**Nom de code du médicament [3]**	**Dosage(s) [4] et forme pharmaceutique du médicament**
-	rhHIP/PAP	ALF-5755	2.8 mg par fiole sous forme lyophilisée

[1] Médicaments expérimentaux et médicaments non expérimentaux utilisés dans le cadre de la recherche

[2] Editer des lignes supplémentaires si nécessaire

[3] Le cas échéant

[4] Noter sur une même ligne tous les dosages d'une même forme pharmaceutique d'un même médicament

Partie réservée à l'Afssaps (date/signature)

Cette attestation a[illegible] : non applicable

[illegible] Général et par délégation
Dr C. BELORGEY-BISMUT
Chef du Dpt de l'Evaluation des Essais Cliniques
et des Médicaments à Statut Particulier

09 JUIL. 2010

Si vous ne recevez pas toutes les pages de cette télécopie, veuillez contacter le secrétariat de l'Unité essais cliniques médicaments et hors produits de santé au : 33 (0) 1 55 87 36 41.

Confidentialité
Cette transmission est à l'attention exclusive du(des) destinataire(s) ci-dessus mentionné(s) et peut contenir des informations privilégiées et/ou confidentielles. Si vous n'êtes pas le destinataire voulu ou une personne mandatée pour lui remettre cette transmission, vous avez reçu ce document par erreur et toute utilisation, révélation, copie ou communication de son contenu est interdite. Si vous avez reçu cette transmission par erreur, veuillez nous en informer par téléphone immédiatement et nous retourner le message original par courrier. Merci.

Confidentiality
This transmission is intended to the addressee(s) listed above only and may contain preferential or/and confidential information. If you are not the intended recipient, you are hereby notified that you have received the document by mistake and any use, disclosure, copying or communication of the content of this transmission is prohibited. If you have received this transmission by mistake, please call us immediately and return the original message by mail. Thank you.
