# Supplementary material for: A Proof of Concept, Phase II Randomized European Trial, on the Efficacy of ALF-5755, a Novel Extracellular Matrix-Targeted Antioxidant in Patients with Acute Liver Diseases
Source: PLoS One. 2016 Mar 16;11(3):e0150733. doi: 10.1371/journal.pone.0150733 (PMC4794150; doi:10.1371/journal.pone.0150733)
Supplement: S3 Text — (PDF) [file pone.0150733.s005.pdf]

Klinische Prüfung

BfArM
Bundesinstitut für Arzneimittel
und Medizinprodukte

BfArM • Kurt-Georg-Kiesinger-Allee 3 • D-53175 Bonn

ORION
Clinical Services Limited
Herrn Matthew Davis
7, Bath Road
SL1 3UA SLOUGH, Berkshire
UNITED KINGDOM

per Fax: 0044 1753 578 081

Postanschrift:
Kurt-Georg-Kiesinger-Allee 3
D-53175 Bonn
http://www.bfarm.de
Telefon: (0228) 207-4318
(0228) 99307-4318
Telefax: (0228) 207-5207
(0228) 99307-5207
e-mail: klinpruefung@bfarm.de

Nachrichtlich:
Landesbehörde Nordrhein-Westfalen
Ethikkommission der Med. Fakultät Universität Duisburg-Essen

Ihre Zeichen und Nachricht vom	Gesch.Z.: Bitte bei Antwort angeben	(0228) 99307-	Bonn,
28.02.2011<br>25.03.2011<br>28.05.2011<br>20.06.2011	61-3910-**4037156**	4318	-4. Juli 2011

Betr.: Antrag auf Genehmigung einer klinischen Prüfung § 42 Abs. 2 AMG, Inhaltliche Prüfung der Antragsunterlagen gemäß § 9 Abs. 1 GCP-V

**Eingangsdatum BfArM**	:	**02.03.2011**
**Mängelschreiben**	:	**14.03.2011**
**Eingang nach Formalprüfung**	:	**28.03.2011**
**Eingang nach Bescheid**	:	**01.06.2011**
**Eingang nach Bescheid 2 mit Bedingungen**	:	**20.06.2011**
**EudraCT-Nummer**	:	**2010-020657-14**
**Vorlage-Nummer**	:	**4037156**
**Sponsor**	:	**Alfact Innovation**
**Legal representative Sponsor**	:	**./.**
**Prüfplancode des Sponsors**	:	**ALF-5755_P2_ALF**
**Protokollversion**	:	**02 vom 11.02.2011**
**IMP Nummer PR01**	:	ALF-5755, 2,8 mg, Konzentrat zur Herstellung einer Infusionslösung
**IMP Nummer PL01**	:	Placebo zu PR1, 0,9% NaCl-Lösung, Infusionslösung, alle in Deutschland zugelassenen Fertigprodukte

Titel der klinischen Prüfung :
A multicentre, double-blind, randomized, placebo-controlled study to evaluate the efficacy and the safety of ALF-5755 in patients with nonacetaminophen severe acute hepatitis and early stage acute liver failure

Leiter/in der klinischen Prüfung gemäß § 40 Abs. 1 Nr. 5 AMG:
Dr. med. Guido Gerken, Essen

Bescheid

Mit den bei uns am 20.06.2011 eingegangenen von Ihnen nachgereichten Unterlagen, wurde der Nachweis erbracht, dass alle in unserer Genehmigung mit aufschiebenden Bedingungen vom 16.06.2011 benannten Forderungen erfüllt werden. Die Durchführung der oben genannten klinischen Prüfung wird genehmigt.

Rechtsbehelfsbelehrung:
Gegen diesen Bescheid kann innerhalb eines Monats nach Bekanntgabe Widerspruch erhoben werden. Der Widerspruch ist bei dem Bundesinstitut für Arzneimittel und Medizinprodukte, Kurt-Georg-Kiesinger-Allee 3, 53175 Bonn, schriftlich oder zur Niederschrift einzulegen.

Die Unterlagen werden unter der o. g. Vorlagenummer verwahrt. Bitte geben Sie die Vorlage-Nr. und den Code des Prüfplans stets im weiteren Schriftverkehr zur o.g. klinischen Prüfung an und verwenden Sie als Postanschrift:

Bundesinstitut für Arzneimittel und Medizinprodukte,
Fachregistratur „Klinische Prüfungen"
Kurt-Georg-Kiesinger-Allee 3, D-53175 Bonn,
Tel. +49 (0)228-207-4318, Fax +49 (0)228-207-5207

Mit freundlichen Grüßen
im Auftrag
i.V. M. Schyns

PD Dr. med. Thomas Sudhop
