# Supplementary material for: A Proof of Concept, Phase II Randomized European Trial, on the Efficacy of ALF-5755, a Novel Extracellular Matrix-Targeted Antioxidant in Patients with Acute Liver Diseases
Source: PLoS One. 2016 Mar 16;11(3):e0150733. doi: 10.1371/journal.pone.0150733 (PMC4794150; doi:10.1371/journal.pone.0150733)
Supplement: S4 Text — (PDF) [file pone.0150733.s006.pdf]

Hôpital de Bicêtre – 78 rue du général Leclerc – 94275 Le Kremlin Bicêtre Cedex
Président : Jacques CARRE – Secrétaire : Brigitte PILATE

Projet de Recherche N° : *10-016* Kremlin Bicêtre le 12 juillet 2010

Le Comité a été saisi le 25 mai 2010

Madame ERNAULT-ROSEAU de ORION Santé pour le compte du promoteur ALFACT INNOVATION –320 rue Saint Honoré –75001 PARIS concernant le projet de recherche intitulé :

«ETUDE MULTICENTRIQUE, RANDOMISEE, EN DOUBLE AVEUGLE, CONTROLEE CONTRE PLACEBO EVALUANT L'EFFICACITE ET LA TOLERANCE DE L'ALF-5755 CHEZ LES PATIENTS PRESENTANT UNE HEPATITE AIGUË SEVERE OU UNE HEPATITE FULMINANTE NON LIEE AU PARACETAMOL »

« A MULTICENTRE, DOUBLE-BLIND, RANDOMIZED, PLACEBO-CONTROLLED STUDY TO EVALUATE THE EFFICACITY AND THE SAFETY OF ALF-5755 IN PATIENTS WITH NONACETAMINOPHEN SEVERE ACUTE HEPATITIS AND EARLY STAGE ACUTE LIVER FAILURE"

(Référence du promoteur :N° ALF-5755_P2_ALF-2010-020657-14)

dont le coordonnateur national est le Professeur SAMUEL – Centre Hépatobiliaire – Hôpital Paul Brousse – 12 avenue Paul Vaillant Couturier – 94804 VILLEJUIF CEDEX

Le comité a examiné les informations relatives à ce projet (version 01 du 7 mai 2010 AVEC Addendum 1 du 24 juin 2010) et les réponses que vous lui avez apporté issues des questions de la première lecture lors des séances du 2 juin 2010 et du 7 juillet 2010 avec lettre d'information et de consentement pour le patient version française 1.1 du 24 juin 2010, lettre d'information et de consentement pour la personne de confiance version française 1.1 du 24 juin 2010 et la liste des centres participants à l'étude du 18 mai 2010

Membres présents lors de la délibération de votre protocole

Premier Collège :

- Recherche biomédicale : V. GAJDOS, pédiatre (T), A. LAFLANCHE, épidémiologiste (T) M. PUCHEAULT (T) et M. BOTTLAENDER (S)
- Pharmacien hospitalier : A. M. TABURET (T)
- Infirmière : C. ASTOUL (T)

Deuxième collège :

- Ethique : P. CASAURANG (S)
- Psychologue : S. SCHWAB (T)
- Travailleur social : A. M. PETIT (S)
- Juriste : F. BOISSY (T) et V. A. LAFOY (T)
- Associations agréées : J. P. ESCANDE (S)

Le Comité :

- **considérant l'intérêt du projet de recherche**
- **le respect d'une méthodologie adaptée à la question posée**
- **considérant le respect d'un consentement libre et éclairé formulé au regard d'une note d'information adaptée**
- **considérant la balance bénéfice risque pour le patient positive**

AVIS FAVORABLE sans restriction

Docteur Vincent GAJDOS
Président de la séance du 7 juillet 2010
