# Supplementary material for: A Proof of Concept, Phase II Randomized European Trial, on the Efficacy of ALF-5755, a Novel Extracellular Matrix-Targeted Antioxidant in Patients with Acute Liver Diseases
Source: PLoS One. 2016 Mar 16;11(3):e0150733. doi: 10.1371/journal.pone.0150733 (PMC4794150; doi:10.1371/journal.pone.0150733)
Supplement: S5 Text — (PDF) [file pone.0150733.s007.pdf]

MEDIZINISCHE FAKULTÄT
DER UNIVERSITÄT DUISBURG-ESSEN

**Ethik-Kommission**
Robert-Koch-Str. 9-11 45147 Essen
Fax: +49 (0) 201/723-5837
ethikkommission@uk-essen.de
www.uni-due.de/ethikkommission

**Vorsitzender:** Prof. Dr. W. Havers
☎ +49 (0) 201 / 723-1847

**Geschäftsführerin:** K. Weidler
☎ +49 (0) 201 / 723-3834
Katrin.weidler@uk-essen.de
Sekretariat: M. Onlangue-Ndza
☎ +49 (0) 201 / 723-3637
monika.onlangue-ndza@uk-essen.de

**Wiss. Mitarbeiterin:** Dr. S. Bein
☎ +49 (0) 201 / 723-3448
sabine.bein@uk-essen.de

**Wiss. Mitarbeiterin:** Dr. K. Appel
☎ +49 (0) 201 / 723-3113
katrin.appel@uk-essen.de

Ethik-Kommission Medizinische Fakultät der Universität Duisburg- Essen

Orion Clinical Services Ltd.
Mr. Matthew Davis
7 Bath Road, Slough
Berkshire SL1 3UA
United Kingdom

Post + FAX
11.04.11
[illegible]

Essen, den 07. April 11 / SB

☒ BfArM
☒ Herrn Prof. Dr. Guido Gerken, Klinik für Gastroenterologie und Hepatologie, Universitätsklinikum Essen

| | |
|---|---|
| **Studie:** | **A multicentre, double-blind, randomized, placebo-controlled study to evaluate the efficacy and the safety of ALF-5755 in patients with nonacetaminophen severe acute hepatitis and early stage acute liver failure** |
| **EudraCT-Nr.:** | **2010-020657-14** |
| **Protokoll:** | **ALF-5755_P2_ALF** |
| **Sponsor:** | **Alfact Innovation, France** |
| **Antragsteller:** | **Orion Clinical Services GmbH, UK** |
| **Unser Zeichen:** | **11-4652** |

Sehr geehrter Herr Davis,

die Ethik-Kommission der Medizinischen Fakultät der Universität Duisburg-Essen hat die o. g. klinische Prüfung in ihrer Sitzung am 14.03.2011 beraten.

**Die Ethik-Kommission erteilt eine zustimmende Bewertung.**

Begründung: Versagungsgründe gemäß § 42 Abs. 1 AMG liegen nicht vor.

Die zustimmende Bewertung wird an den Eintritt nachfolgender Bedingungen geknüpft:

Allgemeine Hinweise:
Es muss gewährleistet sein, dass nur einwilligungsfähige Patienten an der Studie teilnehmen.

Hinweise zur Patienteninformation und Einwilligungserklärung:

1. Folgende Begriffe sind laienverständlich zu erklären oder durch einen allgemeinverständlichen Begriff zu ersetzen: HIV-Serologie (Seite 3), Tubenligatur (Seite 4), kontraindiziert (Seite 4), Infektionen (Seite 4).
2. Der Abschnitt zur Patientenversicherung (Seite 5 - 6) ist um folgende Angaben zu ergänzen:
   - Versicherungshöchstsumme pro Person
   - Hinweis auf weitere Obliegenheiten in den Versicherungsbedingungen

- Hinweis, dass dem Patienten eine Kopie der Versicherungsbestätigung und der allgemeinen Versicherungsbedingungen zusammen mit der Patienteninformation ausgehändigt wird.

  Die Sätze „Der Umfang des Versicherungsschutzes … ausgehändigt bekommen." (Seite 5) und „Auf Wunsch … Versicherungsbedingungen." (Seite 6) sind zu löschen.

3. In der Einwilligungserklärung ist auf Seite 8 und Seite 10 „…Versicherungsbedingungen habe ich erhalten." durch „…Versicherungsbedingungen und eine Kopie der Versicherungsbestätigung habe ich erhalten." zu ersetzen.

Bitte lassen Sie der Ethik-Kommission die entsprechend modifizierte Fassung der Patienteninformation und Einwilligungserklärung mit den eindeutig gekennzeichneten Änderungen in einfacher Ausfertigung zukommen.

Der Bewertung liegen die in Anhang 1 aufgeführten Unterlagen zugrunde.

Die Bewertung ist für die in Anhang 2 aufgeführten Prüferinnen / Prüfer und Prüfstelle gültig.

An der Abstimmung haben die in Anhang 3 aufgeführten Kommissionsmitglieder teilgenommen.

Der Bescheid für die zu zahlenden Gebühren für die Bearbeitung der Studie durch die Ethik-Kommission wurde direkt an Orion Deutschland geschickt.

Mit freundlichen Grüßen

Prof. Dr. W. Havers

**Allgemeine Hinweise:**

- Die ethische und rechtliche Verantwortung für die Durchführung dieser klinischen Prüfung verbleibt beim Sponsor, bei der Leiterin/dem Leiter der klinischen Prüfung und bei den Prüferinnen/Prüfern.
- Zusammensetzung und Arbeitsweise der Ethik-Kommission entsprechen nationalen Gesetzen, Vorschriften und der ICH-GCP-Leitlinie in der jeweils gültigen Fassung.
- Ab dem 1.11.2007 ist aufgrund des 2. Gesetzes zum Bürokratieabbau (Bürokratieabbaugesetz II) vom 9.10.2007 – GV.NRW.2007 S. 393 – gegen Verwaltungsakte kein Widerspruch mehr möglich. Sollten Sie mit der mit diesem Schreiben verbundenen Entscheidung nicht einverstanden sein, müssten Sie hiergegen binnen eines Monats Klage beim zuständigen Verwaltungsgericht erheben. Näheres entnehmen Sie bitte der nachstehenden Rechtsmittelbelehrung.

**Rechtsmittelbelehrung:**

Gegen das vorliegende Votum kann innerhalb eines Monats nach Bekanntgabe Klage beim Verwaltungsgericht Gelsenkirchen, Bahnhofsvorplatz 3, 45879 Gelsenkirchen erhoben werden. Die Klage ist schriftlich einzureichen oder zur Niederschrift des Urkundsbeamten der Geschäftsstelle zu erheben.

Falls durch das Verschulden eines von Ihnen Bevollmächtigten die Frist versäumt werden sollte, würde dieses Verschulden Ihnen zugerechnet werden.

**Anlagen:** Anhänge 1 - 3

**<u>Anhang 1.1:</u> Vorgelegte Unterlagen (Eingang am 01.03.2011):**

1. Anschreiben vom 24.02.2011
2. Checkliste
3. Modul 1 vom 22.02.2011
4. Modul 2 vom 24.02.2011
5. Bestätigungsschreiben EudraCTNummer vom 05.05.2010
6. Prüfplan ALF-5755_P2_ALF Version 02 vom 11.02.2011
7. Deutsche Synopse Version 1.0 vom 15.02.2011
8. Patienteninformation und Einwilligungserklärung Version 1.0 vom 08.02.2011
9. IB ALF-5755 Version 02 vom 11.05.2010
10. Versicherungsbestätigung vom 22.02.2011
11. Allgemeine Versicherungsbedingungen
12. Unterlagen (Qualifikationsnachweis der Prüfstelle, Lebensläufe, GCP-Zertifikate und Financial disclosures der Prüfärzte) des Prüfzentrums Essen
13. Datenschutzerklärung vom 17.02.2011
14. CD-ROM mit o. g. Dokumenten

**<u>Anhang 1.2:</u> Nachgereichte Unterlagen (Eingang am 16.03.2011):**

1. Anschreiben vom 15.03.2011
2. korrigiertes Modul 1
3. Mustervertrag
4. Versicherungsbestätigung vom 03.03.2011
5. DSMB Charter Version 1 vom 12.10.2010
6. CD-ROM mit o. g. Dokumenten

**<u>Anhang 2:</u> Zustimmend bewertete Prüferinnen / Prüfer und Prüfstelle:**

1. Prof. Dr. Guido Gerken (Hauptprüfer / LKP), Prof. Dr. Ali Canbay und Dr. Christoph Jochum am Prüfzentrum Klinik für Gastroenterologie und Hepatologie, Universitätsklinikum Essen, Hufelandstr. 55, 45122 Essen

**<u>Anhang 3:</u> Bei der Sitzung am 14.03.2011 anwesende Mitglieder der Ethik-Kommission der Medizinischen Fakultät der Universität Duisburg-Essen:**

| | |
|---|---|
| Prof. Dr. W. Havers | Klinik für Kinderheilkunde III, Universitätsklinikum Essen |
| Prof. Dr. Th. Bajanowski | Institut für Rechtsmedizin, Universitätsklinikum Essen |
| Prof. Dr. B. Hauffa | Klinik für Kinderheilkunde II, Universitätsklinikum Essen |
| Dr. K. Naßenstein | Institut für Radiologie, Universitätsklinikum Essen |
| Dr. D. Schneider-Stengel | Pastoralreferent, Bistum Essen |
| S. van Bömmel-Wegmann | Apotheke, Universitätsklinikum Essen |
| Dr. R. Weber | Klinik für Neurologie, Universitätsklinikum Essen |
| Ass. jur. K. Weidler | Stabsstelle Recht und Drittmittel, Universitätsklinikum Essen |
| J. Westhaus | Patientenvertreter |

**Schriftliche Stellungnahme:**

| | |
|---|---|
| Dr. W. Eberhardt | Innere Klinik (Tumorforschung), Universitätsklinikum Essen |
| PD Dr. Th. Lauenstein | Institut für Radiologie, Universitätsklinikum Essen |
