# Supplementary material for: A Proof of Concept, Phase II Randomized European Trial, on the Efficacy of ALF-5755, a Novel Extracellular Matrix-Targeted Antioxidant in Patients with Acute Liver Diseases
Source: PLoS One. 2016 Mar 16;11(3):e0150733. doi: 10.1371/journal.pone.0150733 (PMC4794150; doi:10.1371/journal.pone.0150733)
Supplement: S6 Text — (PDF) [file pone.0150733.s008.pdf]

Statistical Analysis Plan

ALF-5755_P2_ALF

A multicentre, double-blind, randomised, placebo-controlled study to evaluate the efficacy and the safety of ALF-5755 in patients with nonacetaminophen severe acute hepatitis and early stage acute liver failure

Prepared for:

Alfact Innovation

Prepared by:

ORION Clinical Services Ltd.
7 Bath Road, Slough
Berkshire SL1 3UE UK
Tel: +44 (0) 1753 578080 Fax: +44 (0) 1753 578081

CONFIDENTIAL

**Version**	**Author**
Version 2 Final	Susan Bogle

Statistical Analysis Plan

ALF-5755_P2_ALF

A multicentre, double-blind, randomised, placebo-controlled study to evaluate the efficacy and the safety of ALF-5755 in patients with nonacetaminophen severe acute hepatitis and early stage acute liver failure

Author: Susan Bogle
Version: 2 Final

The undersigned have reviewed and revised this SAP and find it to be consistent with the study requirements:

Paul Amouyal, President, Alfact Innovation	Date
Susan Bogle, Director of Biometrics, ORION Clinical Services Ltd.	Date

TABLE OF CONTENTS

1 INTRODUCTION 7

1.1 General 7
1.2 Changes from Protocol 7
1.3 Changes from Previous Version of the SAP 7

2 STUDY OBJECTIVES 7

3 STUDY DESIGN 7

3.1 Overview 7
3.2 Study Timepoints 8
3.3 Inclusion and Exclusion Criteria 8
3.4 Sample Size Considerations 8
3.5 Randomisation 9

4 STUDY VARIABLES AND COVARIATES 9

4.1 Primary Variable 9
4.2 Secondary Efficacy Variables 9
4.3 Exploratory Efficacy Variables 10
4.4 Safety Variables 10
4.5 Pharmacokinetic Variables 11
4.6 Other Outcome Variables 11

5 DEFINITIONS 11

6 STUDY POPULATIONS 11

6.1 Safety Population 11
6.2 Intent-to-Treat (ITT) Population 12
6.3 Per Protocol (PP) Population 12
6.4 PK Population 12

7 SAFETY MONITORING 12

8 INTERIM ANALYSES 12

9 DATA 12

9.1 CRF Data 12
9.2 External Data 13
9.2.1 PK Data 13
9.2.2 CK18 Data and Other Biomarkers 13
9.3 Randomisation list 13
9.4 Programming and Data Review 13

10 STATISTICAL METHODS 13

10.1 General Principles 13
10.2 Missing Data 14
10.3 Pooling of Sites 14
10.4 Multiple Comparisons 14
10.5 Subgroup Analyses 14
10.6 Statistical Issues 14

11 STATISTICAL OUTPUT 15

11.1 Patient Disposition 15
11.2 Demographic and Baseline Characteristics 15

11.3 Medical History and Current Medical Conditions 15
11.4 Prior and Concomitant Medication 15
11.5 Concomitant Procedures and Surgery 16
11.6 Study Drug Exposure 16
11.7 Efficacy Analyses 16
11.7.1 Primary Variable 16
11.7.2 Secondary Variables 17
11.8 Evaluation of Exploratory Endpoint(s) 17
11.9 Subgroup Analyses 18
11.10 Post-hoc Exploratory Analyses 18
11.11 PK Analyses 18
11.11.1 Plasma Concentration Data 18
11.11.2 PK Parameters 19
11.12 Safety Analyses 19
11.12.1 Adverse Events 19
11.12.2 Laboratory Data 20
11.12.3 Anti-ALF-5755 antibodies 20
11.12.4 Clinical Examination 20
11.12.5 Vital Signs 20
11.12.6 Electrocardiogram 20
11.12.7 Arterial Blood Gas with Lactates 21

12 VALIDATION 21

13 LITERATURE CITATIONS/REFERENCES 21

14 LIST OF TABLES, FIGURES AND LISTINGS 22

14.1 List of Tables 22
14.2 List of Figures 24
14.3 List of Listings 25

15 SHELLS FOR TABLES, FIGURES AND LISTINGS 26

16 STUDY FLOW CHART 94

GLOSSARY OF ABBREVIATIONS

%CV	Coefficient of Variation
Abs	Antibodies
AE	Adverse Event
ALF	Acute Liver Failure
ALT	Alanine Transaminase (also SGPT)
AM	Arithmetic Mean
ANA	Antinuclear Antibody
anti-LKM	Antibodies to Liver and Kidney Microsomes
AP	Alkaline Phosphatase
ASMA	Anti-smooth Muscle Antibody
AST	Aspartate Transaminase (also SGOT)
ATC	Anatomical Therapeutic Chemical
AUC	Area Under the Plasma Concentration Curve
BMI	Body Mass Index
BP	Blood Pressure
CI	Confidence Interval
CK	Cytokeratin
C_{max}	Maximum Observed Plasma Concentration
CRA	Clinical Research Associate
CRF	Case Report Form
CS	Clinically Significant
CSR	Clinical Study Report
CTC	Common Toxicity Criteria
CTCAE	Common Toxicity Criteria Adverse Event
D	Day
DSMB	Data Safety Monitoring Board
ECG	Electrocardiogram
FV	Factor V
GCS	Glasgow Coma Scores
GGT	Gamma-glutamyl-transferase
GM	Geometric Mean
H	Hour
HAV	Hepatitis A Virus
HBV	Hepatitis B Virus
hCG	Human Chorionic Gonadotropin
HCV	Hepatitis C Virus
HDV	Hepatitis D Virus
HE	Hepatic Encephalopathy
HEV	Hepatitis E Virus
HEENT	Head, Eyes, Ears, Nose, Throat
HIV	Human Immunodeficiency Virus
HSV	Herpes Simplex Virus
ICH	International Conference on Harmonisation
Ig	Immunoglobulin
INR	International Normalised Ratio
ITT	Intention-to-treat
k	Terminal Rate Constant
LNR	Limit of Hospital Laboratory Normal Range
MedDRA	Medical Dictionary for Regulatory Activities
MELD	Model for End-Stage Liver Disease
N	Number of Patients
n	Number of Events

NCS	Not Clinically Significant
PCR	Polymerase Chain Reaction
PICD	Patient Informed Consent Document
PK	Pharmacokinetics
PP	Per Protocol
PR	Prothrombin Ratio
PT	Preferred Term
QC	Quality Control
R	Accumulation Ratio
RBC	Red Blood Cell
SAE	Serious Adverse Event
SAH	Severe Acute Hepatitis
SAP	Statistical Analysis Plan
SD	Standard Deviation
SOC	System Organ Class
$t_{1/2}$(h)	Terminal phase half-life
TEAE	Treatment-Emergent Adverse Event
T_{max}	Time from Dosing to the Maximum Observed Plasma Concentration
VZV	Varicella Zoster Virus
WBC	White Blood Cell
WHO	World Health Organisation
WHODD	World Health Organisation Drug Dictionary

1 INTRODUCTION

1.1 General

This statistical analysis plan (SAP) describes the statistical methods to be used during the reporting and analyses of data collected under Alfact Innovation Protocol ALF-5755_P2_ALF and should be read in conjunction with the study protocol and case report form (CRF).

This version of the plan has been developed using the protocol Version 4 dated 13 February 2012 and CRF dated 15 July 2010 with revised page 4 dated on 11 March 2011, page 9 dated on 15 Sept. 2011, page 58 dated on 11 March 2011, page 63 dated on 15 Sept. 2011 and page 77 dated 16 Sept 2010. Any further changes to the protocol or CRF will be reviewed for potential impact on the SAP which will be amended if it is deemed necessary.

At the time of writing this version of the SAP, recruitment is ongoing.

Draft 1 was reviewed by the ORION project manager, medical writer and independent statistician. The analysis plan was finalised and approved by the sponsor prior to database lock. Additional exploratory analyses may be performed following review of the results.

1.2 Changes from Protocol

Model for End-Stage Liver Disease (MELD) has been added as an additional secondary efficacy variable as detailed in Section 4.2.

1.3 Changes from Previous Version of the SAP

The use of planned assessment times and LNR=80 in the derivations of the primary efficacy variable has been clarified.

The modification using baseline when H0 was not recorded for the primary efficacy variable has been defined.

Only hepatitis etiology will be included as a factor in the efficacy analyses.

Details of the survival analysis have been clarified.

2 STUDY OBJECTIVES

Primary:

- to evaluate the efficacy of ALF-5755 versus placebo, measured by rate of change of prothrombin ratio (PR) during the 72 hours following treatment initiation, in patients with nonacetaminophen Severe Acute Hepatitis (SAH) and early stage Acute liver failure (ALF).

Secondary:

- to evaluate the safety and tolerability of ALF-5755 versus placebo in patients with nonacetaminophen SAH and early stage ALF.
- to determine the pharmacokinetic (PK) parameters of ALF-5755 versus placebo in patients with nonacetaminophen SAH and early stage ALF.

3 STUDY DESIGN

3.1 Overview

This is a prospective, multicentre, double-blind, placebo-controlled study.

Male and female adult patients with nonacetaminophen severe acute hepatitis and early stage acute liver failure will receive slow intravenous infusions of 10 mg (25 ml) of ALF-5755 or placebo

(physiological saline solution: 0.9% NaCl) over 10 minutes using automatic syringes. The infusions will be repeated every 12 hours for 3 days (6 doses: H0, H12, H24, H36, H48 and H60). Patients will then be followed until Day 21.

A minimum of 60 adult male and female patients with nonacetaminophen SAH and early stage ALF will be recruited into the study and randomised in the ratio 1:1.

Recruitment will continue until 60 patients are evaluable, i.e. have no major deviation impacting the evaluability of the primary endpoint and have PR assessments allowing rate of change of PR calculation.

3.2 Study Timepoints

The time between baseline assessments, randomisation and first injection should be as short as possible and should not exceed 48 hours. The treatment phase consists of 3 days of 12 hourly treatments (6 infusions) with the primary endpoint at H72. Follow up is at H84, D8 and D21.

Visit	Day	Hour	Window
Screening/Baseline		Pre-randomisation	
Infusion		H0	± 10 min
Infusion		H12	± 10 min
Infusion		H24	± 10 min
Infusion		H36	± 10 min
Infusion		H48	± 10 min
Infusion		H60	± 10 min
Follow up (primary)		H72	± 10 min
Follow up		H84	± 10 min
Follow up	D8		± 2 days
Follow up	D21		± 2 days

.
See Section 16 for the Study Schedule.

3.3 Inclusion and Exclusion Criteria

To be eligible for inclusion into this study, each patient must fulfill all inclusion criteria and not violate any exclusion criteria (for the protocol under which they are entered) during screening prior to randomisation. Details of the inclusion and exclusion criteria are presented in the protocol and amendments.

3.4 Sample Size Considerations

The sample size has been determined from the Beaujon series for patients that fulfilled the inclusion criteria for this study. The mean (0.22) and standard deviation (0.27) of the PR slope (0-72H) from this series were assumed for the placebo group. The standard deviation for the active group was assumed to be 0.32. (See the protocol for the justification of this assumption.) Based on these assumptions, a sample of size 60 (30 per group) will have 80% power to detect a treatment effect of doubling the PR slope (mean slope for ALF-5755 = 0.44) with 5% significance using a 2-sided 2-sample t-test.

Considering the recruiting rate of eligible patients in the 2 major centres (Beaujon and CHB) and the 8 other centres planned to participate to the trial, this sample size should be reached within 18 months.

3.5 Randomisation

Randomisation will be in the ratio 1:1 stratified by site. Randomisation numbers will be allocated to patients sequentially within site following eligibility assessment and consent. Allocation to treatment will be by the site pharmacist (or designee) opening a randomisation allocation envelope.

The randomisation will be programmed by the study statistician at ORION. The final list and the allocation envelopes will be generated by the unblinded statistician.

4 STUDY VARIABLES AND COVARIATES

4.1 Primary Variable

Prothrombin Ratio (PR) will be measured and recorded on the CRF at baseline, H0 before 1st infusion, H12 before 2nd infusion, H24 before 3rd infusion, H36 before 4th infusion, H48 before 5th infusion, H60 before 6th infusion, H72, H84, D8 (after H0), D21 (after H0) or premature discontinuation.

The primary variable for statistical comparison between treatment groups will be **rate of change of PR during 72 hours following treatment initiation** calculated as:

(PR at H72 – PR at H0 pre-dose) / 72.

Assessments will be included for the primary (ITT) analysis whether or not they are within the visit window.

If PR reaches the limit of the hospital laboratory normal range of values (LNR = 80) before H72, the formula will be modified as follows:

(80 – PR at H0 pre-dose) / nominal[1] time to first reach 80

If the patient has no recorded PR at H72 but does have an earlier post-H0 assessment, the formula will be modified as follows:

(Last recorded PR – PR at H0 pre-dose) / nominal[2] time to last recorded PR

If the patient has no recorded PR at H0 but does have an earlier pre-H0 assessment (baseline), the relevant formula above will be modified to use PR at baseline instead of PR at H0 pre-dose, and to add 12 hours to the time in the denominator to reflect the time between baseline and H0[3].

If the patient withdraws early, an extra PR sampling at time of withdrawal will be done if possible, and used as the last recorded value of PR in this formula.

It is expected that all patients will have at least one post-baseline assessment of PR. If any patients have no post-baseline assessment of PR, a decision will be made on a case by case basis at the blind data review meeting before database lock whether or not to impute values for the analysis.

4.2 Secondary Efficacy Variables

MELD will be calculated from serum bilirubin (mg/dL), International Normalised Ratio (INR) and serum creatinine (mg/dL) using the formula:

---

[1] Planned assessment time, i.e. 12 hours, 24 hours,etc

[2] Planned assessment time, i.e. 12 hours, 24 hours,etc

[3] This applied to just one patient (1205), whose baseline assessment was reported by Alfact to have been done 12 hours pre-dose. The decision was made before unblinding. If the patient is on placebo, this will give a reliable estimate of rate of change of PR; if the patient is on ALF5755, it will give a conservative estimate.

$$MELD = 3.78 \ln(bilirubin) + 11.2 \ln(INR) + 9.57 \ln(creatinine) + 6.43$$

with the following modifications:

- Any value (bilirubin, INR or creatinine) less than 1 will be scored as 1
- If the patient has been dialyzed twice within the previous 7 days, then the value of creatinine used will be 4.0 mg/d|L. (Evidence of no dialysis before enrolment will be based on Exclusion Criterion 9. If dialysis is performed after enrolment, it will be recorded on the concomitant procedures page of the CRF. This information will be used to determine the appropriate value for creatinine to use in the formula.)

The following secondary efficacy variables will be analysed:

- Rate of change of Factor V (FV) during 72 hours following treatment initiation; defined as for the primary including the modifications
- Rate of change of International Normalised Ratio (INR) during 72 hours following treatment initiation; defined as for the primary including the modifications
- Rate of change of MELD during 72 hours following treatment initiation; defined as for the primary including the modifications
- Rate of change of Alanine Transaminase (ALT) plasma level during 72 hours following treatment initiation; defined as for the primary including the modifications
- Rate of change of Aspartate Transaminase (AST) plasma level during 72 hours following treatment initiation; defined as for the primary including the modifications
- Change of Hepatic Encephalopathy (HE) grade during 72 hours following treatment initiation
- Overall survival rate at D21
- Transplant-free survival rate at D21
- Liver transplantation at D21
- Length of hospitalisation (days)

4.3 Exploratory Efficacy Variables

The following exploratory efficacy variable will be analysed:

- Total and cleaved Cytokeratin (CK) 18 plasma levels
- Other biomarkers

4.4 Safety Variables

Safety will be evaluated by the following parameters:

- Adverse events (AEs) recorded from the timepoint that the Patient Informed Consent Document (PICD) has been signed
- Safety laboratory assessments, including haematology (haemoglobin, hematocrit, complete WBC count, red blood cell [RBC] count platelet count); biochemistry (fibrinogen, alkaline phosphatase, gamma-glutamyltransferase [GGT], total and conjugated bilirubin, ammonia, albumin, creatinine, urea, sodium, potassium, chloride, bicarbonates, lactates, calcium, magnesium, phosphorus, glucose, total proteins, amylase and lipase)
- Vital signs (body temperature, systolic and diastolic blood pressure, heart rate and respiratory rate)
- Physical examination

- ECG
- Recording of concomitant medication
- Beta human chorionic gonadotropin (hCG) blood pregnancy test for females of child-bearing potential

4.5 Pharmacokinetic Variables

ALF-5755 plasma concentrations will be performed in the first 16 patients included at Paul Brousse Hospital and Beaujon Hospital and other centres if necessary for adequate recruitment. Baseline plasma concentrations measured at H0, reflecting endogenous HIP/PAP levels will be subtracted from each ALF-5755 plasma concentration. PK samples will be collected at the following times:

- 1st infusion: H0 just before 1st infusion, 10 minutes (end of infusion), H1, H2, H6, H12 (just before 2nd infusion)
- 5th infusion: H48 (just before 5th infusion)
- 6th infusion: H60 just before 6th infusion, H60 10 minutes (end of infusion), H61, H62, H66, H72, H84

PK parameters (C_{max}, t_{max}, AUC_{0-12}, AUC_{0-t} and $t_{1/2}$) of ALF-5755 will be determined from plasma concentrations measured after 1st infusion (H0) and after the 6th infusion (H60) and provided to ORION CS for statistical analysis. T_{max} is expected to just reflect the time of the sample collection at the end of infusion.

All PK blood samples (of 5 mL each) will always be taken from the opposite arm to the one where study treatment is infused.

4.6 Other Outcome Variables

None

5 DEFINITIONS

Study Drug. Study drug is taken to mean either ALF-5755 or placebo.

Baseline. Baseline for efficacy and safety analyses is defined by patient and by variable as the last non-missing value before the first dose of study drug. This will normally be the pre-dose assessment at the H0 visit but if this assessment is missing then the assessment at the Baseline visit will be used instead if available.

Treatment Exposure*.* Treatment exposure is the total volume of all infusions at H0, H12, H24, H36, H48 and H60.

Protocol Deviations and Violations. The terms “protocol deviation” and “protocol violation” are considered interchangeable in the context of the statistical analysis, and refer to any deviation recorded by the Project Manager or Clinical Research Associate (CRA), or detected by Data Management or by Statistical programming checks as described in Section 9.4.

6 STUDY POPULATIONS

6.1 Safety Population

The Safety Population will include all patients who are randomised to treatment and receive at least one administration of ALF-5755 or placebo. Subjects who receive the wrong treatment will be analysed as treated when using the Safety Population.

6.2 Intent-to-Treat (ITT) Population

The ITT Population will include all patients in the Safety Population who provide at least one post-dose efficacy assessment or for whom the primary endpoint is imputed. (See Section 4.1.) Subjects who receive the wrong treatment will be analysed as randomised when using the ITT Population.

6.3 Per Protocol (PP) Population

The PP Population will include all patients in the ITT Population who complete the double-blind treatment phase of the study with no major protocol deviations (i.e. impacting the primary endpoint).

The following deviations will be programmed initially:

1. Failed any inclusion/exclusion criteria (as entered on the relevant page of the CRF)
2. Did not receive at least 5 complete injections (80% of the treatment)
3. Did not attend visits within the visit windows
4. Premature termination of the study due to protocol violation, including non compliance with study procedures

Definition of major/minor deviations and allocation to the study populations will be agreed before database lock and unblinding.

The primary analysis will be repeated using the PP Population. If there are no exclusions from the ITT Population, the table using the PP Population will not be produced.

6.4 PK Population

The PK Population is defined as those patients who have any PK results.

7 SAFETY MONITORING

The Data Safety Monitoring Board (DSMB) was set up to periodically evaluate the overall safety profile and review the adverse reactions experienced by each individual patient.

It was planned to report safety data to the DSMB following treatment completion (D3) in the first 2, 4, 10, 20, 30, 40, 50 patients with additional meetings if necessary. In practice this schedule was varied slightly.

Details about the DSMB composition and meeting information are described in the DSMB charter.

The DSMB will be blinded. Exceptionally the DSMB may request unblinded data.

At each scheduled meeting, the DSMB will review disposition, demographic and safety data.

8 INTERIM ANALYSES

No interim analysis is planned.

9 DATA

9.1 CRF Data

CRF data will be provided by ORION data management to the statistics department as ORION standard raw SAS datasets. Populated datasets will be available when programming starts. These may contain dummy data if real data is not yet available.

9.2 External Data

9.2.1 PK Data

An Excel spreadsheet, containing patient id information and PK sample times (nominal and actual) for patients who have PK samples taken, will be provided to Atlanbio by ORION Data Management. This will be populated by Atlanbio with dummy ALF-5755 and anti-ALF-5755 concentration data and returned to ORION to upload to a SAS dataset for programming of the statistical outputs. The updated spreadsheet showing all patients on the clinical database (without any PK data) will be sent to Atlanbio immediately following database lock and unblinding of the study. Atlanbio will populate this with the final PK concentration data and again send to ORION to allow production of the final outputs.The PK parameters for ALF-5755 will be derived by ORION and saved as a SAS dataset for programming of the statistical outputs.

9.2.2 CK18 Data and Other Biomarkers

An Excel spreadsheet containing patient id information and CK-18 sample times (nominal and actual) will be provided to Inserm by ORION Data Management. This will be populated by Inserm with dummy CK-18 data and returned to ORION to upload to a SAS dataset for programming of the statistical outputs. The updated spreadsheet showing all patients on the clinical database (without any CK-18 data) will be sent to Inserm immediately following database lock and unblinding of the study. Inserm will populate this with the final CK-18 data and again send to ORION to allow production of the final outputs.

Other biomarker parameters will be included in the spreadsheet provided by Inserm in the same format.

9.3 Randomisation list

The randomisation list will be uploaded to a SAS dataset following database lock.

9.4 Programming and Data Review

Once the SAP is finalised, programming and review of analysis datasets, tables, figures and listings will be ongoing during the data management of the study. Outputs for the DSMB will be reviewed, but not fully QCd.

When the final data is considered clean, key listings (to be agreed) will be run and distributed to the study team for review. A blind data review meeting will be held to discuss the outcome of this review, the imputations for the primary endpoint and the protocol deviations. Once all data issues have been resolved and the analysis populations approved, the database will be locked. The final run of outputs and quality control (QC) will then take place. This will be done in 2 stages: The main batch will be delivered first; the remaining outputs (identified in Section 14) will be programmed and delivered on request by Alfact.

10 STATISTICAL METHODS

10.1 General Principles

All statistical methods will be based on the International Conference on Harmonisation (ICH) E9 document “Statistical Principles for Clinical Trials”.

Data will be summarised by treatment group. A total column showing all patients will be included for baseline and safety summaries. Where appropriate, data will also be summarised by visit. The format of the summaries is defined in the shells at the end of this document.

In summary and analysis tables of continuous variables, standard descriptive statistics (N, mean, standard deviation [SD], median, minimum and maximum) will be presented. 95% confidence

Interval (CI) will be presented in the statistical analysis outputs as appropriate. For PK summaries, arithmetic mean (AM), geometric mean (GM) and coefficient of variation (%CV) will be used to summarise the data. The minimum and maximum statistics will be presented in summary tables to the same number of significant figures as the original data. The mean/AM, median, GM, CI and SD will be presented to one more significant figure than the original data.

In summary tables of categorical variables, the number of non-missing observations by category will be presented with percentages. The number of missing observations will also be presented when non-zero. The denominator for each percentage will be the number of non-missing observations within the analysis set and treatment group (unless otherwise specified). All percentages will be presented to one decimal place.

Patient line plots will use actual time on the time axis. Mean plots will use a linear time scale for the nominal times of the visits and will be labeled by visit.

Classifications of medical history, concomitant medication and adverse events will be sorted alphabetically within the summary tables.

CRF data collected will be presented within data listings. The data listings will be sorted by treatment group, country, centre number, patient number and visit/week/day. Visits outside the visit windows will be identified within the listings.

The date format for all output presentations will be ‘ddMMMyyyy’.

All statistical analysis will be performed using SAS 9.2 or higher.

All hypothesis testing will be carried out at the 5% (2-sided) significance level unless stated otherwise.

P-values will be rounded to four decimal places. P-values less than 0.0001 will be reported as <0.0001 in tables.

If any of the assumptions underlying the formal statistical methods proposed are violated during the analysis on the final data, alternative statistical methods will be used and any changes documented in the statistical methods section of the clinical study report (CSR), including the rationale for use.

10.2 Missing Data

The imputations/modifications to be used for missing data are defined in Sections 4.1 and 4.2.

10.3 Pooling of Sites

Sites will be pooled for all analyses. There will be no adjustment for centre effect or treatment by centre interaction.

10.4 Multiple Comparisons

There will be no corrections to nominal p-values for multiple comparisons.

10.5 Subgroup Analyses

Subgroups will be defined based on initial PR, hepatitis etiology, hepatic encephalopathy and N-acetyl cysteine treatment (from the concomitant medication page of the CRF). (See Section 11.9.)

10.6 Statistical Issues

None

11 STATISTICAL OUTPUT

General principles for layout of the statistical output are described in Section 10.1, including specification of the table columns, and these are illustrated for each unique table in the table shells in Section 15. For clarity and brevity in this document the phrase “by treatment group” is understood for all summaries and is not included within the text of this section.

11.1 Patient Disposition

Information about screening failures will be provided by the project manager and presented in the CSR as a brief summary of the most frequent reasons for failure.

The patient disposition table will summarise the following data for all available subjects:

- The number (%) of patients in the Safety Population
- The number (%) of patients in the ITT Population
- The number (%) of patients in the PP Population
- The number (%) of patients who completed the study

The number (%) of patients who withdraw from the study and the main reason for withdrawal will be summarised.

A listing of all patients with protocol deviations will be presented. A data listing presenting the eligibility for the analysis sets for each patient will also be presented.

11.2 Demographic and Baseline Characteristics

Age will be calculated using Date of Birth (DOB) and Date of first treatment (H0 Visit) and presented as age at last birthday as an integer.

BMI is the patient’s body weight in kilograms divided by the square of the patient’s height in metres.

Age, gender, race, height, weight and BMI will be summarised using the ITT Population.

The following baseline factors will also be summarized using the ITT Population:

- Hepatitis etiology (Viral hepatitis A / Viral Hepatitis B / Viral Hepatitis E / Autoimmune hepatitis / Other etiology / Undetermined etiology / Drug-induced etiology)
- Hepatic encephalopathy grade (0 / I / II / III / IV)[4]
- N-acetyl cysteine treatment (yes / no)

Other parameters collected at baseline will be listed only.

11.3 Medical History and Current Medical Conditions

The number (%) of patients with at least one past or ongoing condition will be presented and broken down by body system using the ITT Population.

11.4 Prior and Concomitant Medication

All medications taken by patients on entry to the study or during the study will be recorded in the CRF. Medications will be classified using the version of the World Health Organisation Drug Dictionary (WHODD) coding dictionary defined in the Data Handling Manual. The Anatomical Therapeutic Chemical (ATC) Classification and WHO-DRUG Preferred Term (PT) will be used to list and summarise the data.

[4] Only classifications 0, I and II should be represented at baseline

Prior medications are defined as medication that started and stopped before Day 1. Only medications where the stop date is prior to Day 1 will be considered prior. If the stop date is unknown or incomplete and the medications cannot definitely be considered as stopped prior to Day 1 then the medications will be considered as concomitant medications at randomisation or change in concomitant medication, depending on the start date.

Concomitant medications at randomisation are defined as medications that started before Day 1 and either stopped on Day 1 or continued into the study. Partial start dates where the medication cannot definitely be considered as starting prior to Day 1 will lead to a categorisation of the medications as having started on or after dosing.

Change in concomitant medication is defined as medication that started on or after Day 1. If the medication start or stop dates are partial then the rules for prior and concomitant medication, detailed above will be observed prior to assigning a category.

The number (%) of patients reporting the use of any prior medications and the number (%) of patients taking each drug by ATC classification and PT will be summarised using the ITT Population. This table will be repeated for concomitant medications at randomisation and for change in concomitant medication.

11.5 Concomitant Procedures and Surgery

The number of concomitant procedures and the number of concomitant surgeries will be tabulated for the 30 day period before the first infusion and for the period from first infusion until the final Day 21 Visit.

11.6 Study Drug Exposure

Infusion volume (total and by infusion) will be summarised. Reason for volume other than 25 ml will be listed.

For each timepoint, infusion delay (yes, no) will be sumarised. Reason for delay will be listed.

11.7 Efficacy Analyses

Treatment comparisons will be ALF-5755 vs, placebo. The main analysis set for the efficacy analyses will be the ITT Population.

The primary efficacy analysis is the treatment comparison of rate of change of PR from H0 to H72 using the Wilcoxon rank-sum test and the ITT Population. A p-value below 0.05 will be considered as significant. This analysis will be repeated for the PP Population.

11.7.1 Primary Variable

Rate of change of PR (hours^{-1}) during the 72 hours following treatment initiation will be summarised. The treatment groups will be compared using the Wilcoxon rank-sum (Mann-Whitney-Wilcoxon) test using the ITT Population.

In addition the group comparison will be adjusted for the following etiology factor using the Cochran-Mantel-Haenszel test (with modified ridit scores):

- Hepatitis etiology
    - Viral hepatitis A
    - Viral Hepatitis B or Autoimmune Hepatitis
    - Other: This will principally be Viral Hepatitis E or Other Etiology or Undetermined Etiology or Drug-induced Etiology

It is expected that these categories will be mutually exclusive. If any patient is classified as more than one factor level then the appropriate classification will be agreed at or before the data review

meeting before database lock and unblinding. All classifications will be reviewed and approved at that time.

Results for PR and changes from baseline will also be summarised. Median change from baseline will be presented in a line graph.

The summaries and analyses will be repeated using the PP Population.

11.7.2 Secondary Variables

The following secondary variables will be analysed in the same way as the primary variable:

- Rate of change of FV
- Rate of change of INR
- Rate of change of MELD
- Rate of change of ALT
- Rate of change of AST

Change in HE grade from H0 to each subsequent timepoint will be summarised and the treatment groups will be compared using the the Cochran-Mantel-Haenszel test (with modified ridit scores), unadjusted and adjusted for the same etiology factor. HE grade will be summarized by visit.

The Glasgow Coma Scale (GCS) will be listed.

Overall survival will be analysed as follows:

- Overall survival at D21 will be tabulated and the treatment groups will be compared using the Chi-squared test.
- Survival time from start of treatment will be compared between the treatment groups using the log rank test with time censored at D21 (20 days from start of treatment). If survival status is unknown at D21, time will be censored at date last known alive. The number (%) of patients with a response and the number (%) censored will be presented. Kaplan-Meier estimates of the median and quartiles and corresponding 95% confidence intervals will be presented for each treatment group. A plot of the Kaplan-Meier survival function by treatment group will be presented.
- Survival time from start of treatment will be compared between the treatment groups adjusting for the etiology risk factor used for the primary analysis based on the Cox proportional hazards model, with time censored at D21 (20 days from start of treatment). If survival status is unknown at D21, time will be censored at date last known alive. The hazard ratio and associated 95% confidence interval will be presented.

Transplant-free survival will be analysed in the same way as overall survival.

Time to transplantation will be analysed in the same way as overall survival, with patients who die being censored at the time of death. The numbers of patients assessed as requiring liver transplantation by D21 will also be presented.

Hospital-free days (i.e. days alive following hospital discharge) up to D21 will be summarised and the treatment groups will be compared using the Wilcoxon rank-sum (Mann-Whitney-Wilcoxon) test. Note that it is not expected that any patients will be readmitted during this period.

11.8 Evaluation of Exploratory Endpoint(s)

Total and cleaved CK18 will be summarised at each assessment. Rate of change from H0 to H72 (i.e. change divided by 72) will also be presented. The treatment groups will be compared using the Wilcoxon rank-sum (Mann-Whitney-Wilcoxon) test.

During data review, it was noted that some samples labeled “pre-dose” were in fact taken after dosing in error. These will be excluded from the summaries and analyses, but will be included (and flagged) in the listings.

Other biomarker parameters will be presented in the same way.

11.9 Subgroup Analyses

The following subgroups are defined (see Section 10.5):

- Viral Hepatitis B or Autoimmune Hepatitis
- Viral Hepatitis E or Other Etiology or Undetermined Etiology or Drug-induced Etiology
- Viral hepatitis A
- Viral Hepatitis B
- Viral Hepatitis E
- Autoimmune hepatitis
- Other etiology (including Viral Hepatitis E) – relevant boxes on the CRF to be defined
- Other etiology (excluding Viral Hepatitis E) – relevant boxes on the CRF to be defined
- Undetermined etiology
- Drug-induced etiology
- Initial PR above or equal to the median
- Initial PR below the median
- Hepatic encephalopathy grade 0
- Hepatic encephalopathy grade I or II
- N-acetyl cysteine treatment
- No N-acetyl cysteine treatment

The primary efficacy analysis (ITT population) will be repeated for these subgroups. Only the first two will be presented as part of the main delivery; the others will be programmed and presented on request by Alfact. (See Section 14)

11.10 Post-hoc Exploratory Analyses

Additional exploratory analyses will be performed following review of the main results. These may include:

- repeating the primary and/or secondary analyses for different combinations of the subgroups
- repeating the primary and/or secondary analyses for biomarker subgroups e.g. based on CK18, IL6 GC globulin, HIP/PAP at admission.

11.11 PK Analyses

11.11.1 Plasma Concentration Data

Pharmacokinetic samples will be collected at the following times for 16 patients:

- H0, H0+10mins, H1, H2, H6
- H12
- H48
- H60, H60+10mins, H61, H62, H66
- H72
- H84

Plasma concentrations of ALF-5755 will be summarised using descriptive statistics (N, AM, GM, SD, %CV, median, minimum, maximum) by scheduled time points.

Individual patient profile plots of plasma concentration vs. actual time points will be presented for ALF-5755 from H0 to H12 (Day 1) and from H60 to H72 (Day 5) on the same graph. The axis will be labelled with the planned sample time points but the concentration will be presented at the actual time point the sample was taken.

11.11.2 PK Parameters

The following PK parameters will be evaluated from the plasma concentrations for ALF-5755 (active group only) for the first and sixth infusion using actual sample times[5]:

C_{max} (ng/mL) – Maximum Plasma Concentration. The maximum plasma concentration measured during the 12 hour post-dose period.

T_{max} (h) – Time to Maximum Plasma Concentration. The time at which the highest plasma concentration was measured during the 12 hour post-dose period. T_{max} will be used as an estimate of the onset of absorption. This will normally be at H0+10mins and at H60+10mins.

k – Terminal Rate Constant. The negative of the slope of the natural log (ln) concentration-time curve in the terminal elimination phase, where the points comprising the ln concentration-time curve form a straight line.

$t_{1/2}$ (h) – Terminal Half-life. ln(2)/k.

AUC_{0-12} (ng/mL) – Area Under the Plasma Concentration Time Curve. The area-under-the-curve, from time zero to 12 hours post-dose, calculated by the log-linear trapezoidal rule (the linear method will be adopted for concentrations rising to the maximum concentration and the logarithmic method for all subsequent data points).

AUC_{0-t} (ng/mL) – Area Under the Plasma Concentration Time Curve. The area-under-the-curve, from time zero to the last quantifiable concentration at time t, calculated by the log-linear trapezoidal rule (the linear method will be adopted for concentrations rising to the maximum concentration and the logarithmic method for all subsequent data points).

Each plasma PK parameter will be summarised using descriptive statistics (N, AM, GM, SD, %CV, median, minimum, maximum) by visit using the PK Population.

The achievement of steady-state will be assessed by comparison of ALF-5755 concentrations on H48, H60 and H72. No formal analysis will be performed.

The extent of accumulation will be assessed by comparison of AUC_{0-12} at H0 and AUC_{0-12} at H60. The accumulation ratio R = (AUC_{0-12} at H60)/(AUC_{0-12} at H0) will be calculated and summarised with the other PK parameters.

11.12 Safety Analyses

11.12.1 Adverse Events

All adverse events (AE) will be classified using the version of the MedDRA coding dictionary specified in the DHM.

Events will be classified as treatment-emergent if they started or increased in severity on or after the first date and time of medication dosing and up to study closure or withdrawal date. If an event start date is partial, then the start day, month, year or stop date will be used to determine if the event is treatment-emergent. If the classification of the AE cannot be determined from the data available, then the event will be considered treatment-emergent.

Treatment-emergent adverse events (TEAEs) will be further classified as follows:

Severe TEAEs: Severity classified as at least ‘severe’ or Common Toxicity Criteria [CTC] grade 3, 4 or 5) or missing.

Serious TEAEs: Serious classified as ‘yes’ or missing.

Drug-related TEAEs: Relationship to study drug classified as ‘possible’, ‘probable’ or ‘definite’ or missing.

---
[5] i.e. the times when the samples were taken, not the nominal times, 12 hours, 24 hours etc.

Serious drug-related TEAEs: Both serious and drug-related, as specified above.

TEAEs leading to withdrawal from study: Action taken classified as 'discontinued'.

TEAEs overall and in each of the above classifications will be summarised.

Summaries for each level of severity will also be presented.

Summaries by system organ class (SOC) and preferred term will also be presented for treatment-emergent events. Similar tables will be presented for each of the classifications of treatment-emergent events above.

All AE summary tables will show the number (%) of patients having at least one event and the number of events in each treatment group and overall. Note: If a patient has multiple AEs with the same preferred term, these will be summarised once within the count for N (%) of patients, but each event will be counted within the number of reports n of each AE.

The following will be presented in listing format within the data summaries:

- Deaths
- Serious Adverse Events
- Adverse Events which Led to Withdrawal

All adverse events (including non-treatment-emergent events) recorded on the CRF will be listed within the data listings.

11.12.2 Laboratory Data

Routine clinical laboratory tests will be carried out throughout the study.

Laboratory data listings will be presented in two ways:

- Abnormal values (presented within the data summaries)
- All laboratory data (presented within the data listings)

The absolute values of each parameter will be summarised at each visit.

Mean plots will be presented for a selection of laboratory parameters, to be agreed before DBL.

11.12.3 Anti-ALF-5755 antibodies

Plasma concentrations of anti-ALF-5755 antibodies will be collected before infusion at H0 on D1, D8 and D21. Results will be summarized at each visit.

11.12.4 Clinical Examination

A clinical examination will be conducted throughout the study and pre- and post-infusion as specified in the study schedule.

For each site (01=General Appearance, 02=Head, Eyes, Ears, Nose, Throat [HEENT] etc) shift tables will be presented showing changes from baseline to each assessment for normal / abnormal clinical examination results.

11.12.5 Vital Signs

Systolic blood pressure, diastolic blood pressure, heart rate and respiratory rate are collected throughout the study and pre- and post-dose. Body temperature is collected throughout the study.

The absolute values of the vital signs will be summarised at each assessment.

11.12.6 Electrocardiogram

A 12-lead ECG will be completed throughout the study and pre- and post-dose.

The number and percentage of the patients with Normal / Abnormal Not Clinically Significant (NCS) / Abnormal Clinically Significant (CS) ECG results will be summarised.

11.12.7 Arterial Blood Gas with Lactates

Results for arterial blood gas (pH, PaO_2, $PaCo_2$, HCO_3, bass excess and lactates) will be listed.

12 VALIDATION

All tables, figures and listings will be subject to independent quality control and visual review. Unique tables will be independently programmed. Findings will be documented in a quality control form and actions taken will also be documented.

The completed form will be reviewed and signed by both programmers and by the head of biostatistics.

13 LITERATURE CITATIONS/REFERENCES

None

14 LIST OF TABLES, FIGURES AND LISTINGS

Outputs to be programmed and delivered on request by Alfact are presented in italics and shaded.

14.1 List of Tables

Demographic Data

Table 14.1.1	Patient Disposition	All Available Subjects
Table 14.1.2	Study Termination and Primary Reason for Withdrawal	All Randomised Patients
Table 14.1.3.1	Demographics and Baseline Characteristics	ITT Population
Table 14.1.3.2	Baseline Factors	ITT Population
Table 14.1.4.1	*Medical History*	*ITT Population*
Table 14.1.4.2	*Current Medical Conditions*	*ITT Population*
Table 14.1.5.1	*Prior Medications*	*ITT Population*
Table 14.1.5.2	*Concomitant Medications*	*ITT Population*
Table 14.1.5.3	*Changes in Concomitant Medications*	*ITT Population*
Table 14.1.5.4	*Concomitant Procedures and Surgery*	*ITT Population*
Table 14.1.6	*Study Drug Exposure*	*Safety Population*

Efficacy Data

Table 14.2.1.1	Analysis of PR (0-72 hours)	ITT Population
Table 14.2.1.2	Analysis of PR (0-72 hours)	PP Population
Table 14.2.2.1	*Analysis of FV (0-72 hours)*	*ITT Population*
Table 14.2.2.2	Analysis of INR (0-72 hours)	ITT Population
Table 14.2.2.3	Analysis of MELD (0-72 hours)	ITT Population
Table 14.2.2.4	*Analysis of ALT (0-72 hours)*	*ITT Population*
Table 14.2.2.5	*Analysis of AST (0-72 hours)*	*ITT Population*
Table 14.2.3.1	*Change in HE Grade*	*ITT Population*
Table 14.2.3.2	*HE Grade*	*ITT Population*
Table 14.2.4.1	*Overall Survival*	*ITT Population*
Table 14.2.4.2	Transplant-free Survival	ITT Population
Table 14.2.4.3	Time to Transplantation	ITT Population
Table 14.2.5	Hospital-free Days	ITT Population
Table 14.2.6.1	Total CK18	ITT Population
Table 14.2.6.2	Cleaved CK18	ITT Population
Table 14.2.6.3	*Biomarkers*	*ITT Population*

Subgroup Analyses

Table 14.2.1.1.1	Analysis of PR (0-72 hours)	ITT Population with Viral Hepatitis B or Autoimmune Hepatitis
Table 14.2.1.1.2	Analysis of PR (0-72 hours)	ITT Population with Viral Hepatitis E, Other Etiology, Undetermined Etiology or Drug-induced Etiology
Table 14.2.1.1.3	*Analysis of PR (0-72 hours)*	*ITT Population with Viral Hepatitis A*
Table 14.2.1.1.4	*Analysis of PR (0-72 hours)*	*ITT Population with Viral Hepatitis B*
Table 14.2.1.1.5	*Analysis of PR (0-72 hours)*	*ITT Population with Viral Hepatitis E*
Table 14.2.1.1.6	*Analysis of PR (0-72 hours)*	*ITT Population with Autoimmune Hepatitis*
Table 14.2.1.1.7	*Analysis of PR (0-72 hours)*	*ITT Population with Other Etiology (including Viral Hepatitis E)*
Table 14.2.1.1.8	*Analysis of PR (0-72 hours)*	*ITT Population with Other Etiology (excluding Viral Hepatitis E)*
Table 14.2.1.1.9	*Analysis of PR (0-72 hours)*	*ITT Population with Undetermined Etiology*
Table 14.2.1.1.10	*Analysis of PR (0-72 hours)*	*ITT Population with Drug-induced Etiology*
Table 14.2.1.1.11	*Analysis of PR (0-72 hours)*	*ITT Population with Initial PR ≥ the median*
Table 14.2.1.1.12	*Analysis of PR (0-72 hours)*	*ITT Population with Initial PR < the median*
Table 14.2.1.1.13	*Analysis of PR (0-72 hours)*	*ITT Population with HE Grade 0*
Table 14.2.1.1.14	*Analysis of PR (0-72 hours)*	*ITT Population with HE Grade I or II*
Table 14.2.1.1.15	*Analysis of PR (0-72 hours)*	*ITT Population with NAC*
Table 14.2.1.1.16	*Analysis of PR (0-72 hours)*	*ITT Population without NAC*

PK Data

Table 14.2.7.1	Plasma Concentration of ALF-5755	PK Population
Table 14.2.7.2	PK Parameters for ALF-5755	PK Population

Safety Data

Table 14.3.1.1	Treatment-Emergent Adverse Events	Safety Population
Table 14.3.1.2	Treatment-Emergent Adverse Events, by SOC and PT	Safety Population
Table 14.3.1.3	*Severe Treatment-Emergent Adverse Events, by SOC and PT*	*Safety Population*
Table 14.3.1.4	*Serious Treatment-Emergent Adverse Events, by SOC and PT*	*Safety Population*
Table 14.3.1.5	*Drug-Related Treatment-Emergent Adverse Events, by SOC and PT*	*Safety Population*
Table 14.3.1.6	*Serious Drug-Related Treatment-Emergent Adverse Events, by SOC and PT*	*Safety Population*
Table 14.3.1.7	*Treatment-Emergent Adverse Events Leading to Withdrawal from the Study, by SOC and PT*	*Safety Population*
Table 14.3.2.1	Listing of Deaths	
Table 14.3.2.2	Listing of Serious Adverse Events	
Table 14.3.2.3	Listing of Withdrawals Due to Adverse Events	
14.3.3	*Narratives of deaths, other serious and certain other significant adverse events (to be presented in the CSR)*	
Table 14.3.4	Listing of Abnormal Laboratory Values	
Table 14.3.5.1	Haematology	Safety Population
Table 14.3.5.2	Biochemistry	Safety Population
Table 14.3.5.3	*Urinalysis*	*Safety Population*
Table 14.3.5.4	*Plasma Concentration of anti-ALF-5755 Antibodies*	*Safety Population*
Table 14.3.6	*Clinical Examination*	*Safety Population*
Table 14.3.7	*Vital Signs*	*Safety Population*
Table 14.3.8	*12-lead Electrocardiogram*	*Safety Population*

14.2 List of Figures

Figure 1.1	PR: Median Change from Baseline	ITT Population
Figure 1.2	*FV: Median Change from Baseline*	*ITT Population*
Figure 1.3	INR: Median Change from Baseline	ITT Population
Figure 1.4	MELD: Median Change from Baseline	ITT Population
Figure 1.5	*ALT: Median Change from Baseline*	*ITT Population*
Figure 1.6	*AST: Median Change from Baseline*	*ITT Population*
Figure 2.1	*Kaplan-Meier Plot of Survival*	*ITT Population*
Figure 2.2	Kaplan-Meier Plot of Transplant-Free Survival	ITT Population
Figure 2.3	*Kaplan-Meier Plot of Time to Transplantation*	*ITT Population*
Figure 3	Individual Patient PK Plots of ALF-5755 Concentration	PK Population

Figure 4	Mean Plots of Laboratory Parameters	Safety Population

14.3 List of Listings

Patient Data Listings

Listing 16.2.1	Discontinued Patients
Listing 16.2.2	Protocol Deviations
Listing 16.2.3	Patients in the Analysis Populations
Listing 16.2.4.1	Demographic Data
Listing 16.2.4.2	Baseline Data
Listing 16.2.4.3	Hepatitis Etiology
Listing 16.2.4.4	Baseline Serology Data
Listing 16.2.5	Study Drug Exposure
Listing 16.2.6	Efficacy Response Data
Listing 16.2.7	Adverse Events
Listing 16.2.8.1	Haematology
Listing 16.2.8.2	Biochemistry
Listing 16.2.8.3	Urinalysis
Listing 16.2.8.4	CK18
Listing 16.2.8.5	Anti-ALF-5755 Antibodies

Individual Patient Data Listings (Archive)

Listing 16.4.1	Final Status
Listing 16.4.2	Visit Dates
Listing 16.4.3.1	Inclusion Criteria
Listing 16.4.3.2	Exclusion Criteria
Listing 16.4.3.3	Eligibility Confirmation and Randomisation Status
Listing 16.4.4	Medical History
Listing 16.4.5.1	Concomitant Medications
Listing 16.4.5.2	Concomitant Procedures
Listing 16.4.5.3	Concomitant Surgery
Listing 16.4.6.1	Hemostasis
Listing 16.4.6.2	Transplantation and Hospitalisation
Listing 16.4.6.3	Hepatic Encephalopathy and Glasgow Coma Scale
Listing 16.4.6.4	Survival Status
Listing 16.4.7.1	PK Data
Listing 16.4.7.2	PK Parameters
Listing 16.4.8	CK18 Sampling

Listing 16.4.9 Clinical Examination
Listing 16.4.10 Vital Signs
Listing 16.4.11 12-Lead Electrocardiogram
Listing 16.4.12 Arterial Blood Gas with Lactates
Listing 16.4.13 Other Sampling Collection Data
Listing 16.4.14 BP and HR Monitoring

15 SHELLS FOR TABLES, FIGURES AND LISTINGS

The intended layouts for tables, figures and listings are presented. However, it may be necessary to change the layouts, as appropriate, upon review of the data available.

Output will be produced as rich text Word documents (.rtf files).

Subject to this, the following will apply:

- Layout will be landscape, fixed width font in font size 8.
- Each output will have the heading:
  ALF-5755_P2_ALF (left); date ddMMMyyyy (right)
- Table headings will define the analysis set used for the summary/analysis.
- Footnotes will be included where appropriate.
  All outputs will have a footnote specifying the SAS program path and filename and the rtf path and filename (left); page x/y (right)
- Patient number and treatment group will be included in all listings.

ALF-5755_P2_ALF ddMMMyyyy

Table 14.1.1 Patient Disposition (All Available Subjects)

	ALF-5755 (N=xx)	Placebo (N=xx)	Total (N=xx)
Screening			xx
Safety Population	xx	xx	xx
ITT Population	xx (xx.x%)	xx (xx.x%)	xx
PP Population	xx (xx.x%)	xx (xx.x%)	xx
Completed	xx (xx.x%)	xx (xx.x%)	xx

Source: Listing 16.x.x

Path\Filename Page x/y

ALF-5755_P2_ALF ddMMMyyyy

Table 14.1.2 Study Termination and Primary Reason for Withdrawal (All Randomised Patients)

	ALF-5755 (N=xx)	Placebo (N=xx)	Total (N=xx)
Completed	xx (xx.x%)	xx (xx.x%)	xx (xx.x%)
Early withdrawal	xx (xx.x%)	xx (xx.x%)	xx (xx.x%)
Main reason for early withdrawal			
Adverse event	xx (xx.x%)	xx (xx.x%)	xx (xx.x%)
Etc	xx (xx.x%)	xx (xx.x%)	xx (xx.x%)

Source: Listing 16.x.x

Path\Filename Page x/y

ALF-5755_P2_ALF ddMMMyyyy

Table 14.1.3.1 Demographics and Baseline Characteristics (ITT Population)

		ALF-5755<br>(N=xx)	Placebo<br>(N=xx)	Total<br>(N=xx)
Age (years)	N	xx	xx	xx
	Mean	xx.xx	xx.xx	xx.xx
	SD	xx.xx	xx.xx	xx.xx
	Median	xx.xx	xx.xx	xx.xx
	Maximum	xx.x	xx.x	xx.x
	Minimum	xx.x	xx.x	xx.x
Gender	Male	xx (xx.x%)	xx (xx.x%)	xx (xx.x%)
	Female	xx (xx.x%)	xx (xx.x%)	xx (xx.x%)
Race	Caucasian	xx (xx.x%)	xx (xx.x%)	xx (xx.x%)
	Etc			
Height (cm)	N			
	Etc			
Weight (kg)	N			
	Etc			
BMI (kg/cm^2)	N			
	Etc			

Source: Listing 16.x.x

Path\Filename Page x/y

ALF-5755_P2_ALF ddMMMyyyy

Table 14.1.3.2 Baseline Factors (ITT Population)

		ALF-5755 (N=xx)	Placebo (N=xx)	Total (N=xx)
Hepatitis etiology	Hepatitis A	xx (xx.x%)	xx (xx.x%)	xx (xx.x%)
	Hepatitis B	xx (xx.x%)	xx (xx.x%)	xx (xx.x%)
	Hepatitis E	xx (xx.x%)	xx (xx.x%)	xx (xx.x%)
	Autoimmune hepatitis	xx (xx.x%)	xx (xx.x%)	xx (xx.x%)
	Other etiology	xx (xx.x%)	xx (xx.x%)	xx (xx.x%)
	Undetermined etiology	xx (xx.x%)	xx (xx.x%)	xx (xx.x%)
	Drug-induced hepatitis	xx (xx.x%)	xx (xx.x%)	xx (xx.x%)
	None of the above	xx (xx.x%)	xx (xx.x%)	xx (xx.x%)
Hepatic encephalopathy	Grade O	xx (xx.x%)	xx (xx.x%)	xx (xx.x%)
	Grade I	xx (xx.x%)	xx (xx.x%)	xx (xx.x%)
	Grade II	xx (xx.x%)	xx (xx.x%)	xx (xx.x%)
N-acetyl cysteine treatment	Yes	xx (xx.x%)	xx (xx.x%)	xx (xx.x%)
	No	xx (xx.x%)	xx (xx.x%)	xx (xx.x%)

Source: Listing 16.x.x

Path\Filename Page x/y

ALF-5755_P2_ALF ddMMMyyyy

Table 14.1.4.1 Medical History (ITT Population)

	ALF-5755 (N=xx)	Placebo (N=xx)	Total (N=xx)
Patients with any history	xx (xx.x%)	xx (xx.x%)	xx (xx.x%)
Patients with any history related to liver disease	xx (xx.x%)	xx (xx.x%)	xx (xx.x%)
General appearance	xx (xx.x%)	xx (xx.x%)	xx (xx.x%)
Head, Eyes, Ears, Nose, Throat	xx (xx.x%)	xx (xx.x%)	xx (xx.x%)
Etc			
Patients with any history not related to liver disease	xx (xx.x%)	xx (xx.x%)	xx (xx.x%)
General appearance	xx (xx.x%)	xx (xx.x%)	xx (xx.x%)
Head, Eyes, Ears, Nose, Throat	xx (xx.x%)	xx (xx.x%)	xx (xx.x%)
Etc			

Source: Listing 16.x.x

Path\Filename Page x/y

This layout also applies to:
Table 14.1.4.2

ALF-5755_P2_ALF ddMMMyyyy

Table 14.1.5.1 Prior Medications (ITT Population)

	ALF-5755 (N=xx)	Placebo (N=xx)	Total (N=xx)
Any Prior Medication	xx (xx.x%)	xx (xx.x%)	xx (xx.x%)
ATC classification	xx (xx.x%)	xx (xx.x%)	xx (xx.x%)
Preferred Term	xx (xx.x%)	xx (xx.x%)	xx (xx.x%)
Preferred Term	xx (xx.x%)	xx (xx.x%)	xx (xx.x%)
Etc	xx (xx.x%)	xx (xx.x%)	xx (xx.x%)
ATC classification	xx (xx.x%)	xx (xx.x%)	xx (xx.x%)
Preferred Term	xx (xx.x%)	xx (xx.x%)	xx (xx.x%)
Preferred Term	xx (xx.x%)	xx (xx.x%)	xx (xx.x%)
Etc	xx (xx.x%)	xx (xx.x%)	xx (xx.x%)
Etc			

Source: Listing 16.x.x

Path\Filename Page x/y

This layout also applies to:
Table 14.1.5.2
Table 14.1.5.3

ALF-5755_P2_ALF ddMMMyyyy

Table 14.1.5.4 Concomitant Procedures and Surgery (ITT Population)

		ALF-5755 (N=xx)	Placebo (N=xx)	Total (N=xx)
Concomitant Procedures				
30 day period before 1st infusion	0	xx (xx.x%)	xx (xx.x%)	xx (xx.x%)
	1	xx (xx.x%)	xx (xx.x%)	xx (xx.x%)
	Etc	xx (xx.x%)	xx (xx.x%)	xx (xx.x%)
1st infusion to D21	0	xx (xx.x%)	xx (xx.x%)	xx (xx.x%)
	1	xx (xx.x%)	xx (xx.x%)	xx (xx.x%)
	Etc	xx (xx.x%)	xx (xx.x%)	xx (xx.x%)
Concomitant Surgery				
30 day period before 1st infusion	0	xx (xx.x%)	xx (xx.x%)	xx (xx.x%)
	1	xx (xx.x%)	xx (xx.x%)	xx (xx.x%)
	Etc	xx (xx.x%)	xx (xx.x%)	xx (xx.x%)
1st infusion to D21	0	xx (xx.x%)	xx (xx.x%)	xx (xx.x%)
	1	xx (xx.x%)	xx (xx.x%)	xx (xx.x%)
	Etc	xx (xx.x%)	xx (xx.x%)	xx (xx.x%)

Source: Listing 16.x.x

Path\Filename Page x/y

ALF-5755_P2_ALF ddMMMyyyy

Table 14.1.6 Study Drug Exposure (Safety Population)

		ALF-5755<br>(N=xx)	Placebo<br>(N=xx)	Total<br>(N=xx)
Infusion volume				
Total	N	xx	xx	xx
	Mean	xx.xx	xx.xx	xx.xx
	SD	xx.xx	xx.xx	xx.xx
	Median	xx.xx	xx.xx	xx.xx
	Maximum	xx.x	xx.x	xx.x
	Minimum	xx.x	xx.x	xx.x
H0	N			
	Etc			
H12	N			
	Etc			
Etc				
Infusion delay				
H0	Yes	xx (xx.x%)	xx (xx.x%)	xx (xx.x%)
	No	xx (xx.x%)	xx (xx.x%)	xx (xx.x%)
H12	Yes	xx (xx.x%)	xx (xx.x%)	xx (xx.x%)
	No	xx (xx.x%)	xx (xx.x%)	xx (xx.x%)
Etc				

Source: Listing 16.x.x

Path\Filename Page x/y

ALF-5755_P2_ALF ddMMMyyyy

Table 14.2.1.1 Analysis of PR (0-72 hours) (ITT Population)

		ALF-5755 (N=xx)	Placebo (N=xx)	p-value
Rate of change of PR	N	xx	xx	
	Mean	xx.xx	xx.xx	
	SD	xx.xx	xx.xx	
	Median	xx.xx	xx.xx	
	Maximum	xx.x	xx.x	
	Minimum	xx.x	xx.x	
Wilcoxon test				x.xxx
CMH test adjusting for hepatitis etiology[1]				x.xxx
PR				
Baseline	N	xx	xx	
	Mean	xx.xx	xx.xx	
	SD	xx.xx	xx.xx	
	Median	xx.xx	xx.xx	
	Maximum	xx.x	xx.x	
	Minimum	xx.x	xx.x	
H0	N			
	Etc			
H12	Etc			
Change from H0 to H12				
H24				
Change from H0 to H24				
Etc				

[1] Hepatitis etiology factor levels: Viral Hepatitis A; Viral Hepatitis B or Autoimmune Hepatitis; Other

Source: Listing 16.x.x

Path\Filename Page x/y

This layout also applies to:
Table 14.2.1.2
Table 14.2.2.1
Table 14.2.2.2
Table 14.2.2.3
Table 14.2.2.4
Table 14.2.2.5

ALF-5755_P2_ALF ddMMMyyyy

Table 14.2.1.1.1 Analysis of PR (0-72 hours) (ITT Population with Viral Hepatitis B or Autoimmune Hepatitis)

		ALF-5755 (N=xx)	Placebo (N=xx)	p-value
Rate of change of PR	N	xx	xx	
	Mean	xx.xx	xx.xx	
	SD	xx.xx	xx.xx	
	Median	xx.xx	xx.xx	
	Maximum	xx.x	xx.x	
	Minimum	xx.x	xx.x	
Wilcoxon test				x.xxx
PR				
Baseline	N	xx	xx	
	Mean	xx.xx	xx.xx	
	SD	xx.xx	xx.xx	
	Median	xx.xx	xx.xx	
	Maximum	xx.x	xx.x	
	Minimum	xx.x	xx.x	
H0	N			
	Etc			
H12	Etc			
Change from H0 to H12				
H24				
Change from H0 to H24				
Etc				

Source: Listing 16.x.x

This layout also applies to:
Table 14.2.1.1.2 to 14.2.1.1.16

ALF-5755_P2_ALF ddMMMyyyy

Table 14.2.3.1 Change in HE grade (ITT Population)

		ALF-5755 (N=xx)	Placebo (N=xx)	p-value
Change 0-12 hours	Increase 4 grades	xx (xx.x%)	xx (xx.x%)	
	Increase 3 grades	xx (xx.x%)	xx (xx.x%)	
	Increase 2 grades	xx (xx.x%)	xx (xx.x%)	
	Increase 1 grades	xx (xx.x%)	xx (xx.x%)	
	No change	xx (xx.x%)	xx (xx.x%)	
	Decrease 1 grades	xx (xx.x%)	xx (xx.x%)	
	Etc			
CMH test				x.xxx
CMH test adjusting for hepatitis etiology[1]				x.xxx
Change 0-24 hours	Increase 4 grades	xx (xx.x%)	xx (xx.x%)	
	Increase 3 grades	xx (xx.x%)	xx (xx.x%)	
	Increase 2 grades	xx (xx.x%)	xx (xx.x%)	
	Increase 1 grades	xx (xx.x%)	xx (xx.x%)	
	No change	xx (xx.x%)	xx (xx.x%)	
	Decrease 1 grades	xx (xx.x%)	xx (xx.x%)	
	Etc			
CMH test				x.xxx
CMH test adjusting for hepatitis etiology[1]				x.xxx
Change 0-36 hours	Increase 4 grades	xx (xx.x%)	xx (xx.x%)	
	Etc			
Etc				

[1] Hepatitis etiology factor levels: Viral Hepatitis A; Viral Hepatitis B or Autoimmune Hepatitis; Other

Source: Listing 16.x.x

Path\Filename Page x/y

ALF-5755_P2_ALF ddMMMyyyy

Table 14.2.3.2 HE grade (ITT Population)

		ALF-5755<br>(N=xx)	Placebo<br>(N=xx)	p-value
Baseline	Grade 0	xx (xx.x%)	xx (xx.x%)	
	Grade I	xx (xx.x%)	xx (xx.x%)	
	Grade II	xx (xx.x%)	xx (xx.x%)	
	Grade III	xx (xx.x%)	xx (xx.x%)	
	Grade IV	xx (xx.x%)	xx (xx.x%)	
H0	Grade 0	xx (xx.x%)	xx (xx.x%)	
	Etc			
H12	Grade 0	xx (xx.x%)	xx (xx.x%)	
	Etc			
Etc				

Source: Listing 16.x.x

Path\Filename Page x/y

ALF-5755_P2_ALF ddMMMyyyy

Table 14.2.4.1 Overall Survival (ITT Population)

		ALF-5755 (N=xx)	Placebo (N=xx)	p-value
Survived to D21	Yes	xx (xx.x%)	xx (xx.x%)	
	No	xx (xx.x%)	xx (xx.x%)	
	Chi-squared test			x.xxxx
Kaplan-Meier estimate (days)	Upper quartile	xx.x	xx.x	
	95% CI	xx.x, xx.x	xx.x, xx.x	
	Median	xx.x	xx.x	
	95% CI	xx.x, xx.x	xx.x, xx.x	
	Lower quartile	xx.x	xx.x	
	95% CI	xx.x, xx.x	xx.x, xx.x	
	Log Rank Test			x.xxxx
	Number (%) of responses	xx (xx.x%)	xx (xx.x%)	
	Number (%) censored	xx (xx.x%)	xx (xx.x%)	
	Missing	xx	xx	
Cox regession analysis adjusting for hepatitis etiology[1]	Hazard ratio			xx.xx
	95% CI			xx.xx, xx.xx
	p-value			x.xxxx

[1] Hepatitis etiology factor levels: Viral Hepatitis A; Viral Hepatitis B or Autoimmune Hepatitis; Other

Source: Listing 16.x.x

Path\Filename Page x/y

This layout also applies to:
Table 14.2.4.2

ALF-5755_P2_ALF ddMMMyyyy

Table 14.2.4.3 Time to Transplantation (ITT Population)

		ALF-5755 (N=xx)	Placebo (N=xx)	p-value
Survived to D21 with no transplant required	Yes	xx (xx.x%)	xx (xx.x%)	
	No	xx (xx.x%)	xx (xx.x%)	
	Chi-squared test			x.xxxx
Survived to D21 with no transplant performed	Yes	xx (xx.x%)	xx (xx.x%)	
	No	xx (xx.x%)	xx (xx.x%)	
	Chi-squared test			x.xxxx
Kaplan-Meier estimate (days)	Upper quartile	xx.x	xx.x	
	95% CI	xx.x, xx.x	xx.x, xx.x	
	Median	xx.x	xx.x	
	95% CI	xx.x, xx.x	xx.x, xx.x	
	Lower quartile	xx.x	xx.x	
	95% CI	xx.x, xx.x	xx.x, xx.x	
	Log Rank Test			x.xxxx
	Number (%) of responses	xx (xx.x%)	xx (xx.x%)	
	Number (%) censored	xx (xx.x%)	xx (xx.x%)	
	Missing	xx	xx	
Cox regession analysis adjusting for hepatitis etiology[1]	Hazard ratio			xx.xx
	95% CI			xx.xx, xx.xx
	p-value			x.xxxx

[1] Hepatitis etiology factor levels: Viral Hepatitis A; Viral Hepatitis B or Autoimmune Hepatitis; Other

Source: Listing 16.x.x

Path\Filename Page x/y

ALF-5755_P2_ALF ddMMMyyyy

Table 14.2.5 Hospital-free Days (ITT Population)

		ALF-5755 (N=xx)	Placebo (N=xx)	p-value
Hospital-free days up to D21[1]	N	xx	xx	
	Mean	xx.xx	xx.xx	
	SD	xx.xx	xx.xx	
	Median	xx.xx	xx.xx	
	Maximum	xx.x	xx.x	
	Minimum	xx.x	xx.x	
	Wilcoxon test			x.xxx

[1] Days alive following hospital discharge

Source: Listing 16.x.x

Path\Filename Page x/y

ALF-5755_P2_ALF ddMMMyyyy

Table 14.2.6.1 Total CK18 (ITT Population)

		ALF-5755 (N=xx)	Placebo (N=xx)	p-value
Change from H0 to H72	N	xx	xx	
	Mean	xx.xx	xx.xx	
	SD	xx.xx	xx.xx	
	Median	xx.xx	xx.xx	
	Maximum	xx.x	xx.x	
	Minimum	xx.x	xx.x	
	Wilcoxon test			x.xxx
H0	N	xx	xx	
	Mean	xx.xx	xx.xx	
	SD	xx.xx	xx.xx	
	Median	xx.xx	xx.xx	
	Maximum	xx.x	xx.x	
	Minimum	xx.x	xx.x	
H12	N			
	Etc			
Etc				

Source: Listing 16.x.x

Path\Filename Page x/y

This layout also applies to:
Table 14.2.6.2
Table 14.2.6.3

ALF-5755_P2_ALF ddMMMyyyy

Table 14.2.7.1 Plasma Concentration of ALF-5755 (PK Population)

		ALF-5755 (N=xx)
H0 (pre-infusion)	N	xx
	AM	xx.xx
	GM	xx.xx
	SD	xx.xx
	%CV	xx.x
	Median	xx.xx
	Maximum	xx.x
	Minimum	xx.x
H0+10 mins (end of infusion)	N	xx
	AM	xx.xx
	GM	xx.xx
	SD	xx.xx
	%CV	xx.x
	Median	xx.xx
	Maximum	xx.x
	Minimum	xx.x
Etc		

Source: Listing 16.x.x

Path\Filename Page x/y

ALF-5755_P2_ALF ddMMMyyyy

Table 14.2.7.2 PK Parameters for ALF-5755 (PK Population)

		ALF-5755 (N=xx)
C_{max} (ng/mL)		
1st infusion	N	xx
	AM	xx.xx
	GM	xx.xx
	SD	xx.xx
	%CV	xx.x
	Median	xx.xx
	Maximum	xx.x
	Minimum	xx.x
6th infusion	N	xx
	AM	xx.xx
	GM	xx.xx
	SD	xx.xx
	%CV	xx.x
	Median	xx.xx
	Maximum	xx.x
	Minimum	xx.x
T_{max} (mins)		
1st infusion	N	
	Etc	
Etc		

Source: Listing 16.x.x

Path\Filename Page x/y

Programming note: Include Accumulation ratio, as defined in Section 11.10

ALF-5755_P2_ALF ddMMMyyyy

Table 14.3.1.1 Treatment-Emergent Adverse Events (Safety Population)

	ALF-5755 (N=xx)			Placebo (N=xx)			Total (N=xx)		
	n	N	%	n	N	%	n	N	%
Treatment Emergent Adverse Events	xx	xx	(xx.x%)	xx	xx	(xx.x%)	xx	xx	(xx.x%)
Severe Treatment Emergent Adverse Events	xx	xx	(xx.x%)	xx	xx	(xx.x%)	xx	xx	(xx.x%)
Serious Treatment Emergent Adverse Events	xx	xx	(xx.x%)	xx	xx	(xx.x%)	xx	xx	(xx.x%)
Drug-Related Treatment Emergent Adverse Events	xx	xx	(xx.x%)	xx	xx	(xx.x%)	xx	xx	(xx.x%)
Serious Drug-Related Treatment Emergent Adverse Events	xx	xx	(xx.x%)	xx	xx	(xx.x%)	xx	xx	(xx.x%)
Treatment Emergent Adverse Events leading to withdrawal	xx	xx	(xx.x%)	xx	xx	(xx.x%)	xx	xx	(xx.x%)
Severity									
CTCAE grade 1 or Mild	xx	xx	(xx.x%)	xx	xx	(xx.x%)	xx	xx	(xx.x%)
CTCAE grade 2 or Moderate	xx	xx	(xx.x%)	xx	xx	(xx.x%)	xx	xx	(xx.x%)
CTCAE grade 3 or Severe	xx	xx	(xx.x%)	xx	xx	(xx.x%)	xx	xx	(xx.x%)
CTCAE grade 4 or Life-threatening	xx	xx	(xx.x%)	xx	xx	(xx.x%)	xx	xx	(xx.x%)
CTCAE grade 5 or Death	xx	xx	(xx.x%)	xx	xx	(xx.x%)	xx	xx	(xx.x%)

n=number of events, N=number of patients, %=percentage of patients
Drug related is defined as relationship to study drug=possible, probable or definite

Source: Listing 16.x.x

Path\Filename Page x/y

ALF-5755_P2_ALF ddMMMyyyy

Table 14.3.1.2 Treatment-Emergent Adverse Events, by SOC and PT (Safety Population)

	ALF-5755 (N=xx)			Placebo (N=xx)			Total (N=xx)		
	n	N	%	n	N	%	n	N	%
Any Adverse Event	xx	xx	(xx.x%)	xx	xx	(xx.x%)	xx	xx	(xx.x%)
SOC	xx	xx	(xx.x%)	xx	xx	(xx.x%)	xx	xx	(xx.x%)
PT	xx	xx	(xx.x%)	xx	xx	(xx.x%)	xx	xx	(xx.x%)
PT	xx	xx	(xx.x%)	xx	xx	(xx.x%)	xx	xx	(xx.x%)
Etc	xx	xx	(xx.x%)	xx	xx	(xx.x%)	xx	xx	(xx.x%)
SOC	xx	xx	(xx.x%)	xx	xx	(xx.x%)	xx	xx	(xx.x%)
PT	xx	xx	(xx.x%)	xx	xx	(xx.x%)	xx	xx	(xx.x%)
PT	xx	xx	(xx.x%)	xx	xx	(xx.x%)	xx	xx	(xx.x%)
Etc	xx	xx	(xx.x%)	xx	xx	(xx.x%)	xx	xx	(xx.x%)
Etc									

n=number of events, N=number of patients, %=percentage of patients

Source: Listing 16.x.x

Path\Filename Page x/y

This layout also applies to:
Tables 14.3.1.3 to 14.3.1.7

ALF-5755_P2_ALF ddMMMyyyy

Table 14.3.2.1 Listing of Deaths

Treatment	Centre/ Patient number	Date of first infusion	Date of death
xxxxxxx	xx-xxxx	ddMMMyyyy	ddMMMyyyy
	xx-xxxx	ddMMMyyyy	ddMMMyyyy
	Etc		
Etc			

Repeat for Placebo

Path\Filename Page x/y

ALF-5755_P2_ALF ddMMMyyyy

Table 14.3.2.2 Listing of Serious Adverse Events

ALF-5755

Centre/ Patient number	Adverse Event Preferred Term SOC Term	Start date	Study day	Stop date /ongoing	Duration of adverse event (days)	Grade[1]	Serious	Relationship to study drug	Treatment	Action taken regarding study drug	Outcome
xxx xxxx	xxxxxxxxx xxxxxxxx xxxxxxx	ddMMMyyyy	xx	ddMMMyyyy	xx	x	Yes /No	None/Related	None/ medication/ Surgery/ Other: xxxxx	None /Discontinued Etc	Recovered without sequelae/ Etc
	xxxxxxxxx xxxxxxxx xxxxxxx	ddMMMyyyy	xx	ddMMMyyyy	xx	x	Yes /No	None/Related	None/ medication/ Surgery/ Other: xxxxx	None /Discontinued Etc	Recovered without sequelae/ Etc
	Etc										
xxx xxxx	xxxxxxxxx xxxxxxxx xxxxxxx	ddMMMyyyy	xx	ddMMMyyyy	xx	x	Yes /No	None/Related	None/ medication/ Surgery/ Other: xxxxx	None /Discontinued Etc	Recovered without sequelae/ Etc
	Etc										
Etc											

[1] CTCAE grade if appropriate or 1=Mild, 2=Moderate, 3=Severe, 4=Life-threatening, 5=Death

Repeat for Placebo

Path\Filename Page x/y

This layout also applies to:
Table 14.3.2.3

ALF-5755_P2_ALF ddMMMyyyy

Listing 14.3.4 Listing of Abnormal Laboratory Values

Treatment received	Parameter	Centre/ Patient number	Visit	Result	Unit	Reference range		Comments
xxxxxx	xxxxxxxxxxxx	xx-xxxx	xxx	xxx.xx	xxxx	xxx.xx, xxx.xx	H/L	xxxxxxxxxxxxxxxxxx
			xxx	xxx.xx	xxxx	xxx.xx, xxx.xx	H/L	xxxxxxxxxxxxxxxxxx
			xxx	xxx.xx	xxxx	xxx.xx, xxx.xx	H/L	xxxxxxxxxxxxxxxxxx
		xx-xxxx	xxx	xxx.xx	xxxx	xxx.xx, xxx.xx	H/L	xxxxxxxxxxxxxxxxxx
			xxx	xxx.xx	xxxx	xxx.xx, xxx.xx	H/L	xxxxxxxxxxxxxxxxxx
			xxx	xxx.xx	xxxx	xxx.xx, xxx.xx	H/L	xxxxxxxxxxxxxxxxxx
			xxx	xxx.xx	xxxx	xxx.xx, xxx.xx	H/L	xxxxxxxxxxxxxxxxxx
			Etc					
		Etc						

Path\Filename Page x/y

ALF-5755_P2_ALF ddMMMyyyy

Table 14.3.5.1 Haematology (Safety Population)

		ALF-5755 (N=xx)	Placebo (N=xx)	Total (N=xx)
Haemoglobin				
Baseline	N	xx	xx	xx
	Mean	xx.xx	xx.xx	xx.xx
	SD	xx.xx	xx.xx	xx.xx
	Median	xx.xx	xx.xx	xx.xx
	Maximum	xx.x	xx.x	xx.x
	Minimum	xx.x	xx.x	xx.x
H0	N			
	Etc			
Etc				
Hematocrit	Etc			
Etc				

Source: Listing 16.x.x

Path\Filename Page x/y

This layout also applies to:
Table 14.3.5.2
Table 14.3.5.3
Table 14.3.5.4

ALF-5755_P2_ALF ddMMMyyyy

Table 14.3.6 Clinical Examination (Safety Population)

		ALF-5755 (N=xx)		Placebo (N=xx)		Total (N=xx)	
		Baseline[1]		Baseline[1]		Baseline[1]	
		Normal	Abnormal	Normal	Abnormal	Normal	Abnormal
General appearance							
HO pre-infusion	Normal	xx (xx.x%)	xx (xx.x%)	xx (xx.x%)	xx (xx.x%)	xx (xx.x%)	xx (xx.x%)
	Abnormal	xx (xx.x%)	xx (xx.x%)	xx (xx.x%)	xx (xx.x%)	xx (xx.x%)	xx (xx.x%)
	Not done[2]	xx	xx	xx	xx	xx	xx
Etc							

[1] Baseline is the last non-missing value before the first dose of study drug.
[2] Not done box ticked or missing. Patients with no clinical examination at baseline are not included in the summary table

Source: Listing 16.x.x

Path\Filename Page x/y

ALF-5755_P2_ALF ddMMMyyyy

Table 14.3.7 Vital Signs (Safety Population)

		ALF-5755 (N=xx)	Placebo (N=xx)	Total (N=xx)
Systolic BP (mmHg)				
Baseline	N	xx	xx	xx
	Mean	xx.xx	xx.xx	xx.xx
	SD	xx.xx	xx.xx	xx.xx
	Median	xx.xx	xx.xx	xx.xx
	Maximum	xx.x	xx.x	xx.x
	Minimum	xx.x	xx.x	xx.x
H0 pre-infusion	N	xx	xx	xx
	Mean	xx.xx	xx.xx	xx.xx
	SD	xx.xx	xx.xx	xx.xx
	Median	xx.xx	xx.xx	xx.xx
	Maximum	xx.x	xx.x	xx.x
	Minimum	xx.x	xx.x	xx.x
Etc				
Diastolic BP (mmHg)	Etc			
Heart rate (beats/min)	Etc			
Respiratory rate (breaths/min)	Etc			
Temperature (˚C)	Etc			

Source: Listing 16.x.x

Path\Filename Page x/y

ALF-5755_P2_ALF ddMMMyyyy

Table 14.3.8 12-lead Electrocardiogram (Safety Population)

		ALF-5755<br>(N=xx)	Placebo<br>(N=xx)	Total<br>(N=xx)
Baseline	Normal	xx (xx.x%)	xx (xx.x%)	xx (xx.x%)
	Abnormal NCS	xx (xx.x%)	xx (xx.x%)	xx (xx.x%)
	Abnormal CS	xx (xx.x%)	xx (xx.x%)	xx (xx.x%)
HO	Normal	xx (xx.x%)	xx (xx.x%)	xx (xx.x%)
	Abnormal NCS	xx (xx.x%)	xx (xx.x%)	xx (xx.x%)
	Abnormal CS	xx (xx.x%)	xx (xx.x%)	xx (xx.x%)
Etc				

Source: Listing 16.x.x

Path\Filename Page x/y

ALF-5755_P2_ALF ddMMMyyyy

Figure 1.1 PR: Median Change from Baseline (ITT Population)

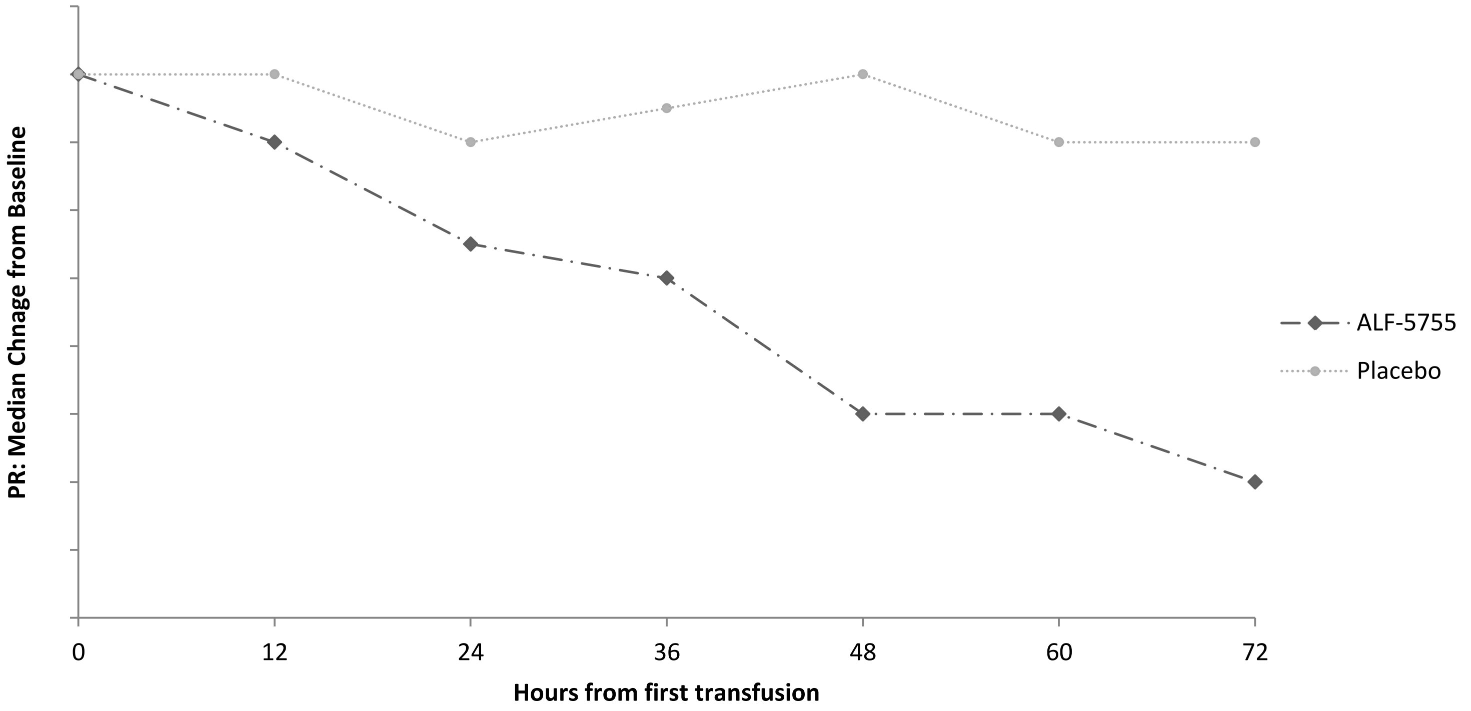

Source: Listing 16.x.x

Path\Filename Page x/y

This layout also applies to:
Figures 1.2, 1.3, 1.4, 1.5, 1.6

ALF-5755_P2_ALF ddMMMyyyy

Figure 2.1 Kaplan-Meier Plot of Survival (ITT Population)

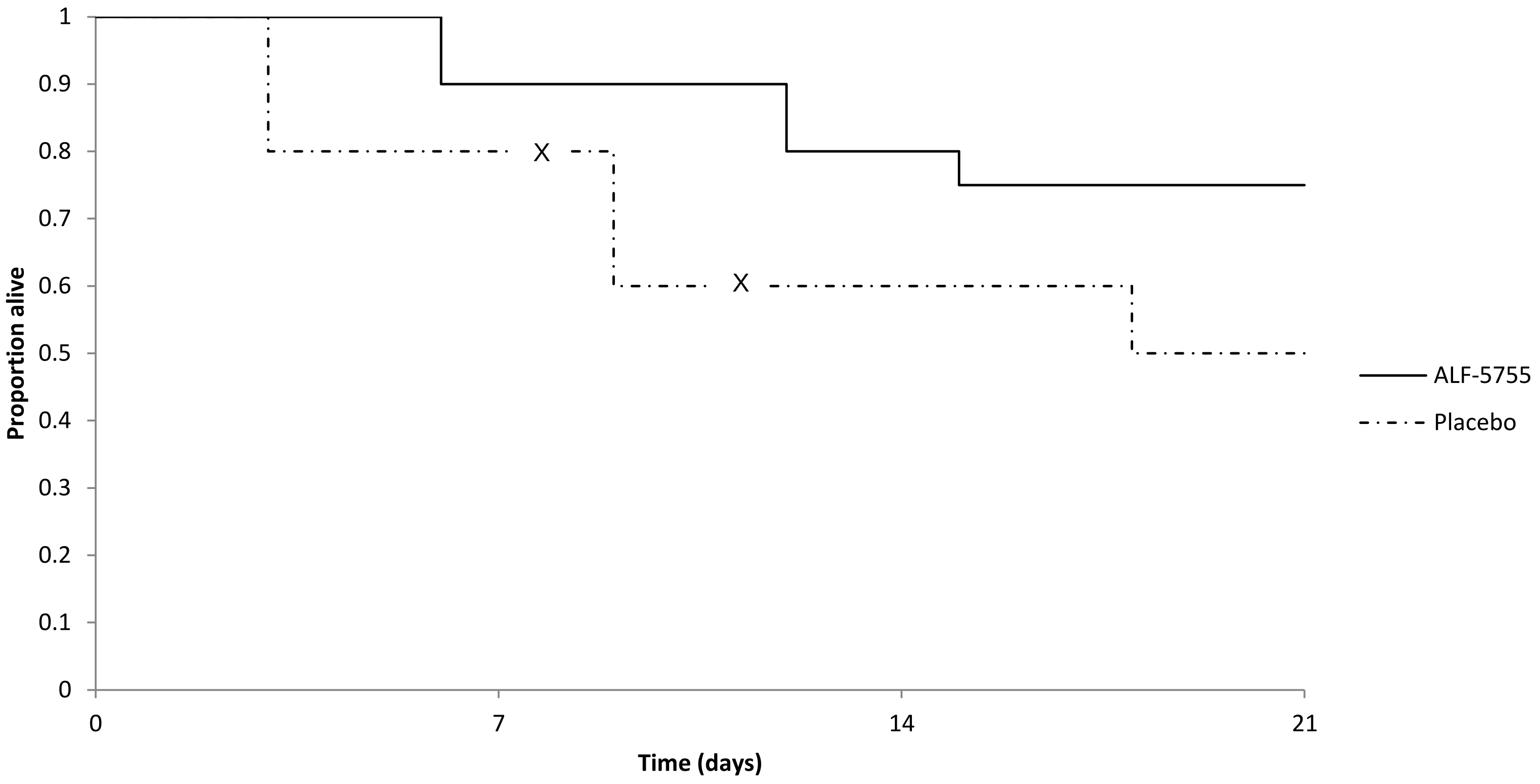

Number known to be alive						
ALF-5755	xx	xx	xx	xx	xx	xx
Placebo	xx	xx	xx	xx	xx	xx

X indicates censored timed

Source: Listing 16.x.x
Path\Filename Page x/y

This layout also applies to:
Figure 2.2
Figure 2.3

ALF-5755_P2_ALF ddMMMyyyy

Figure 3 Individual Patient PK Plots of ALF-5755 Concentration (PK Population)

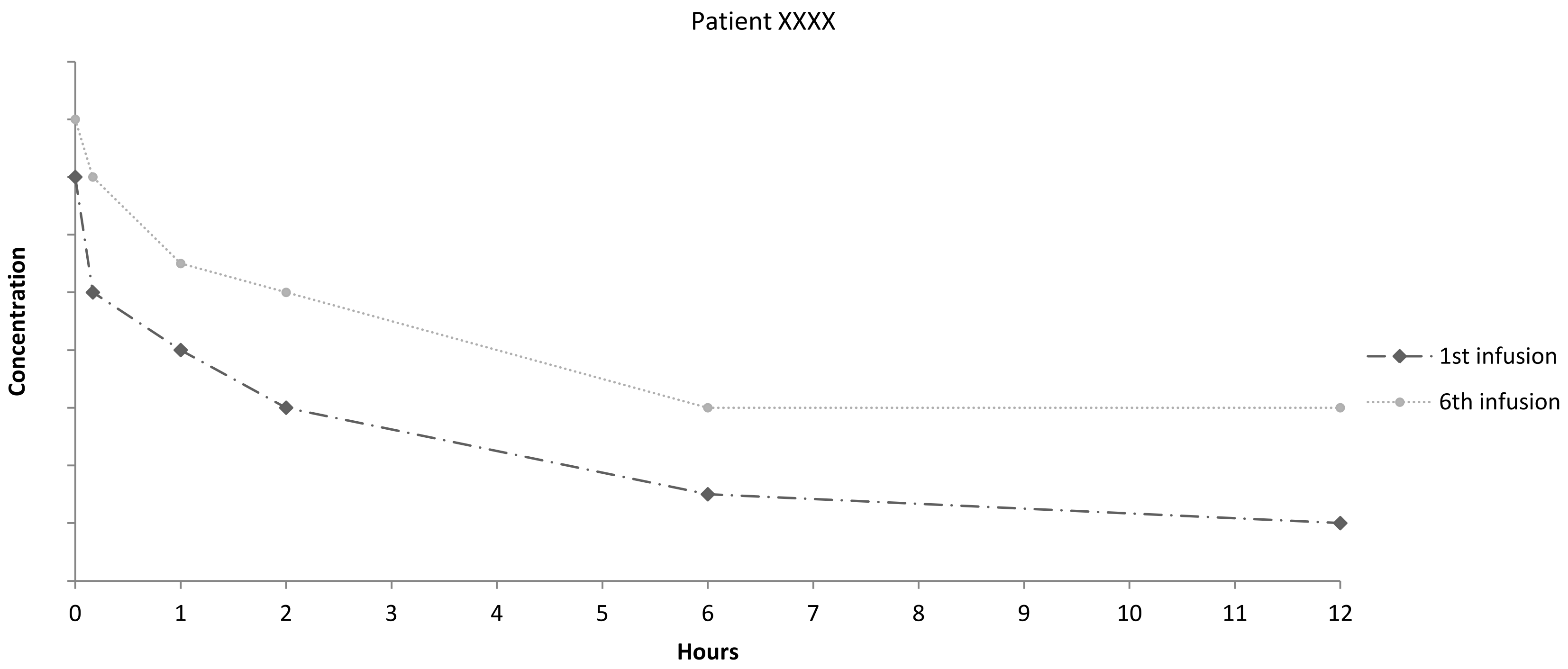

Source: Listing 16.x.x

Path\Filename Page x/y

ALF-5755_P2_ALF ddMMMyyyy

Figure 4 Mean Plots of Laboratory Parameters (Safety Population)

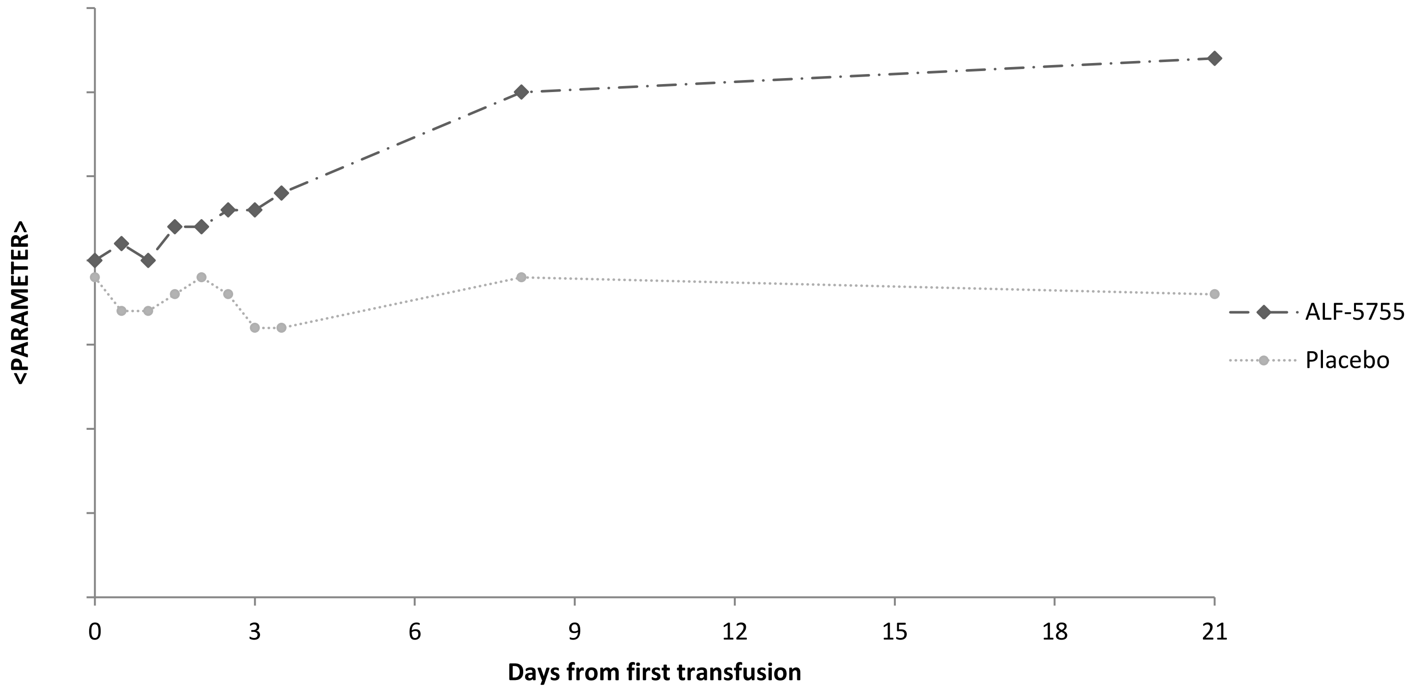

REPEAT FOR PARAMETERS AS AGREED

Source: Listing 16.x.x

Path\Filename Page x/y

ALF-5755_P2_ALF ddMMMyyyy

Listing 16.2.1 Discontinued Patients

Treatment	Centre/ Patient number	First dose date	Last dose date	Date of withdrawal	Main reason for withdrawal	Other reasons for withdrawal
xxxxxx	xx-xxxx	ddMMMyyyy	ddMMMyyyy	ddMMMyyyy	xxxxxxxxxxxxxxxxxxxxxxxxx	
xxxxxx	xx-xxxx	ddMMMyyyy	ddMMMyyyy	ddMMMyyyy	xxxxxxxxxxxxxxxxxxxxxxxxx	xxxxxxxxxxxxxxxxxxxxxxxxx xxxxxxxxxxxxxxxxxxxxxxxxx
xxxxxx	xx-xxxx	ddMMMyyyy	ddMMMyyyy	ddMMMyyyy	xxxxxxxxxxxxxxxxxxxxxxxxx	xxxxxxxxxxxxxxxxxxxxxxxxx
Etc						

Path\Filename Page x/y

Note to programmer: If reason is AE, include AE number(s)

ALF-5755_P2_ALF ddMMMyyyy

Listing 16.2.2 Protocol Deviations

Treatment	Centre/ Patient number	Major deviation	Protocol deviation
xxxxxx	xx-xxxx	Yes/No	xxxxxxxxxxxxxxxxxxxxxxxxxxxxxxxxxxxxxxxxxxxxxxxxxxxxxxxxxxxxxxxxxxxxxxxxxxxxxxxx
	xx-xxxx	Yes/No	xxxxxxxxxxxxxxxxxxxxxxxxxxxxxxxxxxxxxxxxxxxxxxxxxxxxxxxxxxxxxxxxxxxxxxxxxxxxxxxx
	xx-xxxx	Yes/No	xxxxxxxxxxxxxxxxxxxxxxxxxxxxxxxxxxxxxxxxxxxxxxxxxxxxxxxxxxxxxxxxxxxxxxxxxxxxxxxx
	xx-xxxx	Yes/No	xxxxxxxxxxxxxxxxxxxxxxxxxxxxxxxxxxxxxxxxxxxxxxxxxxxxxxxxxxxxxxxxxxxxxxxxxxxxxxxx
Etc			

Page x/y

Path\Filename

ALF-5755_P2_ALF ddMMMyyyy

Listing 16.2.3 Patients in the Analysis Populations

Treatment	Centre/ Patient number	Safety Population	ITT Population	PP Population	PK Population
xxxxxx	xx-xxxx	Yes/No	Yes/No	Yes/No	Yes/No
	xx-xxxx	Yes/No	Yes/No	Yes/No	Yes/No
	xx-xxxx	Yes/No	Yes/No	Yes/No	Yes/No
	xx-xxxx	Yes/No	Yes/No	Yes/No	Yes/No
Etc					

Page x/y

Path\Filename

ALF-5755_P2_ALF ddMMMyyyy

Listing 16.2.4.1 Demographic Data

Treatment	Centre/ Patient number	Date of randomisation	Date of birth	Age (years)	Gender	Beta hCH pregnancy test (date and result)	Race	Height (cm)	Weight (kg)	BMI (kg/m^2)
xxxxxx	xx-xxxx	ddMMMyyyy	ddMMMyyyy	xx	Male/ Female: xxxx	ddMMMyyyy: Negative	White/ Black/ Etc	xxx.x	xxx.x	xxx.x
	xx-xxxx	ddMMMyyyy	ddMMMyyyy	xx	Male/ Female: xxxx		White/ Black/ Etc	xxx.x	xxx.x	xxx.x
	xx-xxxx	ddMMMyyyy	ddMMMyyyy	xx	Male/ Female: xxxx	Not done	White/ Black/ Etc	xxx.x	xxx.x	xxx.x
Etc										

Page x/y

Path\Filename

ALF-5755_P2_ALF ddMMMyyyy

Listing 16.2.4.2 Baseline Data

Treatment	Centre/ Patient number	Jaundice (date of onset)	Liver disease diagnosis	Diagnosis date	Acetaminophen in previous 7 days	>4 g/day at least once	Acetaminophen plasma level result	Alcohol plasma level result
xxxxxx	xx-xxxx	Yes (ddMMMyyyy)/No	Severe acute hepatitis / Etc	ddMMMyyyy	Yes/No	Yes/No	xxxx <unit>	xxxx <unit>
	xx-xxxx	Yes (ddMMMyyyy)/No	Severe acute hepatitis / Etc	ddMMMyyyy	Yes/No	Yes/No	xxxx <unit>	xxxx <unit>
	xx-xxxx	Yes (ddMMMyyyy)/No	Severe acute hepatitis / Etc	ddMMMyyyy	Yes/No	Yes/No	xxxx <unit>	xxxx <unit>
	xx-xxxx	Yes (ddMMMyyyy)/No	Severe acute hepatitis / Etc	ddMMMyyyy	Yes/No	Yes/No	xxxx <unit>	xxxx <unit>
	Etc							
Etc								

Page x/y

Path\Filename

ALF-5755_P2_ALF ddMMMyyyy

Listing 16.2.4.3 Hepatitis Etiology

Treatment	Centre/<br>Patient number	Type 1	Type 2	Type 3	Specify
xxxxxx	xx-xxxx	Viral hepatitis	Hepatitis A		
			Herpes virus	HSV1	xxxxxxxxx
		Etc			
	Etc				
Etc					

Page x/y

Path\Filename

Note to programmer: Only list if the box is ticked

ALF-5755_P2_ALF ddMMMyyyy

Listing 16.2.4.4 Baseline Serology Data

Treatment	Centre/<br>Patient number	Parameter type	Parameter	Collection date time	Test result
xxxxxx	xx-xxxx	Hepatitis A	Anti-HAV-IgM	ddMMMyyyy hh:mm	Positive/Negative
			Anti-HAV-total (IgM & IgG)	ddMMMyyyy hh:mm	Positive/Negative
		Hepatitis B	HBsAg	ddMMMyyyy hh:mm	Positive/Negative
			Etc		
		Etc			
			Ant-HBs	ddMMMyyyy hh:mm	xxx <unit>
	Etc				
Etc					

Page x/y

Path\Filename

ALF-5755_P2_ALF ddMMMyyyy

Listing 16.2.5 Study Drug Exposure

Treatment	Centre/ Patient number	Nominal time	Start time of infusion	Duration	Infusion delayed	Reason	Infusion volume (ml)	Reason for modification	Total volume (mL)
xxxxxx	xx-xxxx	H0	hh:mm	hh:mm	Yes/No	xxxxxxx	xx	xxxxxxxxxxx	
		H12	hh:mm	hh:mm	Yes/No	xxxxxxx	xx	xxxxxxxxxxx	
		H24	hh:mm	hh:mm	Yes/No	xxxxxxx	xx	xxxxxxxxxxx	
		H36	hh:mm	hh:mm	Yes/No	xxxxxxx	xx	xxxxxxxxxxx	
		H48	hh:mm	hh:mm	Yes/No	xxxxxxx	xx	xxxxxxxxxxx	
		H60	hh:mm	hh:mm	Yes/No	xxxxxxx	xx	xxxxxxxxxxx	xxx.x
	xx-xxxx	H0	hh:mm	hh:mm	Yes/No	xxxxxxx	xx	xxxxxxxxxxx	
		Etc							
Etc									

*Outside the visit window

Page x/y

Path\Filename

ALF-5755_P2_ALF ddMMMyyyy

Listing 16.2.6 Efficacy Response Data

Treatment	Centre/ Patient number	Rate of change of PR (0-72H)	Rate of change of FV (0-72H)	Rate of change of INR (0-72H)	Rate of change of ALT (0-72H)	Rate of change of AST (0-72H)	Change in HE grade (0-72H)	Status at D21	Days in hospital
xxxxxx	xx-xxxx	xx.x	xx.x	xx.x	xx.x	xx.x	x	Transplant-free/ Transplanted/Dead	xx
	xx-xxxx	xx.x	xx.x	xx.x	xx.x	xx.x	x	Transplant-free/ Transplanted/Dead	xx
	xx-xxxx	xx.x	xx.x	xx.x	xx.x	xx.x	x	Transplant-free/ Transplanted/Dead	xx
	xx-xxxx	xx.x	xx.x	xx.x	xx.x	xx.x	x	Transplant-free/ Transplanted/Dead	xx

Etc

*Outside the visit window

Page x/y

Path\Filename

ALF-5755_P2_ALF ddMMMyyyy

Listing 16.2.7 Adverse Events

ALF-5755

Centre/ Patient number	AE no	Adverse Event Preferred Term SOC Term	Start date	Study day	Stop date /ongoing	Duration of adverse event (days)	Grade[1]	Serious	Relationship to study drug	Treatment	Action taken regarding study drug	Outcome
xxx xxxx	xx	xxxxxxxxx xxxxxxxx xxxxxxx	ddMMMyyyy	xx	ddMMMyyyy	xx	x	Yes /No	None/Related	None/ medication/ Surgery/ Other: xxxxx	None /Discontinued Etc	Recovered without sequelae/ Etc
	xx	xxxxxxxxx xxxxxxxx xxxxxxx	ddMMMyyyy	xx	ddMMMyyyy	xx	x	Yes /No	None/Related	None/ medication/ Surgery/ Other: xxxxx	None /Discontinued Etc	Recovered without sequelae/ Etc
		Etc										
xxx xxxx	xx	xxxxxxxxx xxxxxxxx xxxxxxx	ddMMMyyyy	xx	ddMMMyyyy	xx	x	Yes /No	None/Related	None/ medication/ Surgery/ Other: xxxxx	None /Discontinued Etc	Recovered without sequelae/ Etc
		Etc										
Etc												

[1] CTCAE grade if appropriate or 1=Mild, 2=Moderate, 3=Severe, 4=Life-threatening, 5=Death

Repeat for Placebo

Path\Filename Page x/y

ALF-5755_P2_ALF ddMMMyyyy

Listing 16.2.8.1 Haematology

Treatment received	Centre/ Patient number	Parameter	Visit	Result	Unit	Reference range		Clinical significance
xxxxxx	xx-xxxx	xxxxxxxxxxx	xxx	xxx.xx	xxxx	xxx.xx, xxx.xx	H/L	CS
			xxx	xxx.xx	xxxx	xxx.xx, xxx.xx	H/L	
			xxx	xxx.xx	xxxx	xxx.xx, xxx.xx	H/L	
		xxxxxxxxxxx	xxx	xxx.xx	xxxx	xxx.xx, xxx.xx	H/L	
			xxx	xxx.xx	xxxx	xxx.xx, xxx.xx	H/L	CS
			*xxx	xxx.xx	xxxx	xxx.xx, xxx.xx	H/L	
			xxx	xxx.xx	xxxx	xxx.xx, xxx.xx	H/L	
			Etc					
	Etc							

*Outside the visit window

Path\Filename Page x/y

This layout also applies to:
Listing 16.2.8.2
Listing 16.2.8.4
Listing 16.2.8.5

ALF-5755_P2_ALF ddMMMyyyy

Listing 16.2.8.3 Urinalysis

Treatment	Centre/ Patient number	Visit	Dipstick result	Local lab result
xxxxxx	xx-xxxx	xxx	Normal/Abnormal/Not done	
		xxx	Normal/Abnormal/Not done	
		xxx	Normal/Abnormal/Not done	Normal/Abnormal NCS/Abnormal CS
		xxx	Normal/Abnormal/Not done	
Etc				

*Outside the visit window

Page x/y

Path\Filename

ALF-5755_P2_ALF ddMMMyyyy

Listing 16.4.1 Final Status

Treatment	Centre/ Patient number	First dose date	Last dose date	Completed treatment	Completed all visits	Primary reason for discontinuation	Secondary reasons for discontinuation
xxxxxx	xx-xxxx	ddMMMyyyy	ddMMMyyyy	Yes/No	Yes/No	xxxxxxxxxxxxxxxxxxxx	xxxxxxxxxxxxxxxxxxxx xxxxxxxxxxxxxxxxxxxx
	xx-xxxx	ddMMMyyyy	ddMMMyyyy	Yes/No	Yes/No	xxxxxxxxxxxxxxxxxxxx	xxxxxxxxxxxxxxxxxxxx
	xx-xxxx	ddMMMyyyy	ddMMMyyyy	Yes/No	Yes/No	xxxxxxxxxxxxxxxxxxxx	xxxxxxxxxxxxxxxxxxxx
Etc							

Page x/y

Path\Filename

Note to programmer: Under reason include all additional information provided, including AE nos, date of last contact and other:specify

ALF-5755_P2_ALF ddMMMyyyy

Listing 16.4.2 Visit Dates

Treatment	Centre/ Patient number	Visit	Date
xxxxxx	xx-xxxx	xx	ddMMMyyyy
		xx	ddMMMyyyy
		*xx	ddMMMyyyy
		xx	ddMMMyyyy
Etc			

*Outside the visit window

Page x/y

Path\Filename

Note to programmer: include date of consent and assessment/collection dates if not listed elsewhere

ALF-5755_P2_ALF ddMMMyyyy

Listing 16.4.3.1 Inclusion Criteria

Definition of criteria	
1.	xxxxxxxxxxxxxxxxxxxxxx
2.	xxxxxxxxxxxxxxxxxxxxxx
	Etc

Page x/y

Path\Filename

Note: The list of criteria will be presented on the first pages of the listing. Patient data will start on the subsequent page.

Note to programmer: List criteria for each version of the protocol

ALF-5755_P2_ALF ddMMMyyyy

Listing 16.4.3.1 Inclusion Criteria

Treatment	Centre/ Patient number	Protocol version	Criteria					
			1	2	3	4	5	6
xxxxxx	xx-xxxx	xxxxxxxx	Yes/No	Yes/No	Yes/No	Yes/No	Yes/No/NA	Yes/No
	xx-xxxx	xxxxxxxx	Yes/No	Yes/No	Yes/No	Yes/No	Yes/No/NA	Yes/No
	xx-xxxx	xxxxxxxx	Yes/No	Yes/No	Yes/No	Yes/No	Yes/No/NA	Yes/No
Etc								

Page x/y

Path\Filename

This layout also applies to:
Listing 16.4.3.2

ALF-5755_P2_ALF ddMMMyyyy

Listing 16.4.3.3 Eligibility Confirmation and Randomisation Status

Treatment	Centre/ Patient number	Date of randomisation	Randomisation number	PK blood sampling	CK18 blood sampling
xxxxxx	xx-xxxx	ddMMMyyyy	Rxxxxx	Yes/No	Yes/No
	xx-xxxx	ddMMMyyyy	Rxxxxx	Yes/No	Yes/No
	xx-xxxx	ddMMMyyyy	Rxxxxx	Yes/No	Yes/No
	xx-xxxx	ddMMMyyyy	Rxxxxx	Yes/No	Yes/No
Etc					

Page x/y

Path\Filename

ALF-5755_P2_ALF ddMMMyyyy

Listing 16.4.4 Medical History

Treatment	Centre/ Patient number	Body system	Description	Start date	Related to liver disease	Ongoing	Current CTC grade	Currently treated
xxxxxx	xx-xxxx	xx-xxxxxxxx	xxxxxxxx	ddMMMyyyy	Yes/No	Yes/No	x	Yes/No
	xx-xxxx	xx-xxxxxxxx	xxxxxxxx	ddMMMyyyy	Yes/No	Yes/No	x	Yes/No
	xx-xxxx	xx-xxxxxxxx	xxxxxxxx	ddMMMyyyy	Yes/No	Yes/No	x	Yes/No
	xx-xxxx	xx-xxxxxxxx	xxxxxxxx	ddMMMyyyy	Yes/No	Yes/No	x	Yes/No
Etc								

Page x/y

Path\Filename

ALF-5755_P2_ALF ddMMMyyyy

Listing 16.4.5.1 Concomitant Medications

Treatment	Centre/ Patient number	Med no	AE no	Therapy ATC Code PT	Route	Total daily dose	Unit	Start date (Stop date/ongoing)	Time in relation to treatment	Reason for medication
xxxxxx	xx-xxxx	xx	xx	xxxxxxxxxxxxxxx xxxxxxxxxxxxx xxxxxxxxxxx	xxx	xx.x	xxx	ddMMMyyyy (ddMMMyyyy/ ongoing)	Prior/ Concomitant/ Change	xxxxxxxxxxxxxxxx xxxxxx
		xx	xx	xxxxxxxxxxxxxxx xxxxxxxxxxxxx xxxxxxxxxxx	xxx	xx.x	xxx	ddMMMyyyy (ddMMMyyyy/ ongoing)	Prior/ Concomitant/ Change	xxxxxxxxxxxxxxxx xxxxxx
		xx	xx	xxxxxxxxxxxxxxx xxxxxxxxxxxxx xxxxxxxxxxx	xxx	xx.x	xxx	ddMMMyyyy (ddMMMyyyy/ ongoing)	Prior/ Concomitant/ Change	xxxxxxxxxxxxxxxx xxxxxx
		xx	xx	xxxxxxxxxxxxxxx xxxxxxxxxxxxx xxxxxxxxxxx	xxx	xx.x	xxx	ddMMMyyyy (ddMMMyyyy/ ongoing)	Prior/ Concomitant/ Change	xxxxxxxxxxxxxxxx xxxxxx

Etc

Page x/y

Path\Filename

ALF-5755_P2_ALF ddMMMyyyy

Listing 16.4.5.2 Concomitant Procedures

Treatment	Centre/ Patient number	Procedure no.	AE no.	Procedure	Frequency	Start date (Stop date/ongoing)	Time in relation to treatment	Reason for procedure
xxxxxx	xx-xxxx	xx	xx	xxxxxxxxxxxxxx	xx	ddMMMyyyy (ddMMMyyyy/ ongoing)	Prior/ Concomitant/ Change	xxxxxxxxxxxxxxxx xxxxxx
		xx	xx	xxxxxxxxxxxxxx	xx	ddMMMyyyy (ddMMMyyyy/ ongoing)	Prior/ Concomitant/ Change	xxxxxxxxxxxxxxxx xxxxxx
		xx	xx	xxxxxxxxxxxxxx	xx	ddMMMyyyy (ddMMMyyyy/ ongoing)	Prior/ Concomitant/ Change	xxxxxxxxxxxxxxxx xxxxxx
		xx	xx	xxxxxxxxxxxxxx	xx	ddMMMyyyy (ddMMMyyyy/ ongoing)	Prior/ Concomitant/ Change	xxxxxxxxxxxxxxxx xxxxxx

Etc

Page x/y

Path\Filename

ALF-5755_P2_ALF ddMMMyyyy

Listing 16.4.5.3 Concomitant Surgery

Treatment	Centre/ Patient number	Surgery no.	AE no.	Surgery description	Date	Time in relation to treatment	Reason for surgery
xxxxxx	xx-xxxx	xx	xx	xxxxxxxxxxxxxx	ddMMMyyyy	Prior/ Concomitant/ Change	xxxxxxxxxxxxxxxxxxxxxx
		xx	xx	xxxxxxxxxxxxxx	ddMMMyyyy	Prior/ Concomitant/ Change	xxxxxxxxxxxxxxxxxxxxxx
		xx	xx	xxxxxxxxxxxxxx	ddMMMyyyy	Prior/ Concomitant/ Change	xxxxxxxxxxxxxxxxxxxxxx
		xx	xx	xxxxxxxxxxxxxx	ddMMMyyyy	Prior/ Concomitant/ Change	xxxxxxxxxxxxxxxxxxxxxx

Etc

Page x/y

Path\Filename

ALF-5755_P2_ALF ddMMMyyyy

Listing 16.4.6.1 Hemostasis

Treatment	Centre/ Patient number	Visit	Test	result	Units	Outside normal range	Clinically significant
xxxxxx	xx-xxxx	Baseline	Prothrombin ratio	xxx		Yes/No	Yes/No
			Factor V	xxx	%	Yes/No	Yes/No
			INR	xxx		Yes/No	Yes/No
			Fibrinogen	xxx	g/L	Yes/No	Yes/No
		HO	Prothrombin ratio	xxx		Yes/No	Yes/No
			Factor V	xxx	%	Yes/No	Yes/No
			INR	xxx		Yes/No	Yes/No
			Fibrinogen	xxx	g/L	Yes/No	Yes/No
		Etc					
Etc							

*Outside the visit window

Page x/y

Path\Filename

ALF-5755_P2_ALF ddMMMyyyy

Listing 16.4.6.2 Transplantation and Hospitalisation

Treatment	Centre/ Patient number	Liver transplant required	Transplantation by D21	Date of transplant	Reason	Hospitalised on Day 8	Hospitalised on D21	Date of discharge
xxxxxx	xx-xxxx	Yes/No	Yes/No	ddMMMyyyy	xxxxxxxxx	Yes/No	Yes/No	ddMMMyyyy
		Yes/No	Yes/No	ddMMMyyyy	xxxxxxxxx	Yes/No	Yes/No	ddMMMyyyy
		Yes/No	Yes/No	ddMMMyyyy	xxxxxxxxx	Yes/No	Yes/No	ddMMMyyyy
Etc								

*Outside the visit window

Page x/y

Path\Filename

ALF-5755_P2_ALF ddMMMyyyy

Listing 16.4.6.3 Hepatic Encephalopathy and Glasgow Coma Scale

Treatment	Centre/ Patient number	Visit	HE grade	GCS
xxxxxx	xx-xxxx	Baseline	x	xx
		D1	x	xx
		D12	x	xx
		Etc		
Etc				

*Outside the visit window

Page x/y

Path\Filename

ALF-5755_P2_ALF ddMMMyyyy

Listing 16.4.6.4 Survival Status

Treatment	Centre/ Patient number	Alive on D21	Date of death	Autopsy	Primary cause of death
xxxxxx	xx-xxxx	Yes/No	ddMMMyyyy	Yes/No/Unknown	xxxxxxxxx
		Yes/No	ddMMMyyyy	Yes/No/Unknown	xxxxxxxxx
		Yes/No	ddMMMyyyy	Yes/No/Unknown	xxxxxxxxx
Etc					

*Outside the visit window

Page x/y

Path\Filename

ALF-5755_P2_ALF ddMMMyyyy

Listing 16.4.7.1 PK Data

Treatment	Centre/ Patient number	Visit	Collection date and time	On time as per protocol	Concentration	Units
xxxxxx	xx-xxxx	H0	ddMMMyyyy hh:mm	Yes/No: specify	xx.x	xx
		H0 10 mins	ddMMMyyyy hh:mm	Yes/No: specify	xx.x	xx
		H1	ddMMMyyyy hh:mm	Yes/No: specify	xx.x	xx
		H2	ddMMMyyyy hh:mm	Yes/No: specify	xx.x	xx
		Etc				
Etc						

*Outside the visit window

Page x/y

Path\Filename

ALF-5755_P2_ALF ddMMMyyyy

Listing 16.4.7.2 PK Parameters

Treatment	Centre/ Patient number	Visit	C_{max}	T_{max}	k	$t_{½}$	AUC_{0-12}	AUC_{0-t}	R
xxxxxx	xx-xxxx	1st infusion	xx.x	xx	x.xx	xx	xx.x	xx.x	
		6th infusion	xx.x	xx	x.xx	xx	xx.x	xx.x	x.xx
	xx-xxxx	1st infusion	xx.x	xx	x.xx	xx	xx.x	xx.x	
		6th infusion	xx.x	xx	x.xx	xx	xx.x	xx.x	x.xx
		Etc							
Etc									

Page x/y

Path\Filename

ALF-5755_P2_ALF ddMMMyyyy

Listing 16.4.8 CK18 Sampling

Treatment	Centre/ Patient number	Visit	Sample collected	Collection date and time	On time as per protocol
xxxxxx	xx-xxxx	H0	Yes/No: specify	ddMMMyyyy hh:mm	Yes/No: specify
		H12	Yes/No: specify	ddMMMyyyy hh:mm	Yes/No: specify
		H24	Yes/No: specify	ddMMMyyyy hh:mm	Yes/No: specify
		Etc			
Etc					

Page x/y

Path\Filename

ALF-5755_P2_ALF ddMMMyyyy

Listing 16.4.9 Clinical Examination

Treatment	Centre/ Patient number	Visit	Record no	Body system	Status	Abnormality
xxxxxx	xx-xxxx	xx	xx	General appearance	Normal/Abnormal/Not done	xxxxxxxxxxxxxxxxxxxxxxxxxxx
				Skin	Normal/Abnormal/Not done	xxxxxxxxxxxxxxxxxxxxxxxxxxx
				HEENT	Normal/Abnormal/Not done	xxxxxxxxxxxxxxxxxxxxxxxxxxx
				Lymph nodes	Normal/Abnormal/Not done	xxxxxxxxxxxxxxxxxxxxxxxxxxx
				Etc		
		*xx	*xx	General appearance	Normal/Abnormal/Not done	xxxxxxxxxxxxxxxxxxxxxxxxxxx
				Skin	Normal/Abnormal/Not done	xxxxxxxxxxxxxxxxxxxxxxxxxxx
				HEENT	Normal/Abnormal/Not done	xxxxxxxxxxxxxxxxxxxxxxxxxxx
				Lymph nodes	Normal/Abnormal/Not done	xxxxxxxxxxxxxxxxxxxxxxxxxxx
Etc				Etc		

*Outside the visit window

Page x/y

Path\Filename

ALF-5755_P2_ALF ddMMMyyyy

Listing 16.4.10 Vital Signs

Treatment	Centre/ Patient number	Visit	Systolic BP (mmHg)	Diastolic BP (mmHg)	Heart rate (beats/min)	Respiratory rate (breaths/min)	Body temperature (˚C)
xxxxxx	xx-xxxx	xx	xxx	xxx	xxx	xx	xx.x
		xx	xxx	xxx	xxx	xx	xx.x
		*xx	xxx	xxx	xxx	xx	xx.x
		xx	xxx	xxx	xxx	xx	xx.x

Etc

*Outside the visit window

Page x/y

Path\Filename

ALF-5755_P2_ALF ddMMMyyyy

Listing 16.4.11 12-Lead Electrocardiogram

Treatment	Centre/ Patient number	Visit	Evaluation	Abnormality	Clinical significance
xxxxxx	xx-xxxx	Baseline	Normal/ Abnormal	xxxxxxxxxxx	CS
		H0 Pre	Normal/ Abnormal	xxxxxxxxxxx	
		H0 Post			
		H1	Normal/ Abnormal	xxxxxxxxxxx	CS
Etc					

Page x/y

Path\Filename

ALF-5755_P2_ALF ddMMMyyyy

Listing 16.4.12 Arterial Blood Gas with Lactates

Treatment	Centre/ Patient number	Date and time of collection	pH	PaO_2	$PaCo_2$	HCO_3	Bass excess	Lactates
xxxxxx	xx-xxxx	ddMMMyyyy HH:MM	xxx	xxx	xxx	xxx	xxx	xxx
	xx-xxxx	ddMMMyyyy HH:MM	xxx	xxx	xxx	xxx	xxx	xxx
	xx-xxxx	ddMMMyyyy HH:MM	xxx	xxx	xxx	xxx	xxx	xxx
	xx-xxxx	ddMMMyyyy HH:MM	xxx	xxx	xxx	xxx	xxx	xxx

Etc

Page x/y

Path\Filename

ALF-5755_P2_ALF ddMMMyyyy

Listing 16.4.13 Other Sampling Collection Data

Treatment	Centre/<br>Patient number	Sample	Visit	Inaccordance with<br>guidelines	Specify
xxxxxx	xx-xxxx	xxxxxxxxxx	xx	Yes/No	xxxxxxxxx
		xxxxxxxxxx	xx	Yes/No	xxxxxxxxx
		xxxxxxxxxx	xx	Yes/No	xxxxxxxxx
Etc					

Page x/y

Path\Filename

ALF-5755_P2_ALF ddMMMyyyy

Listing 16.4.14 BP and HR Monitoring

Treatment	Centre/ Patient number	Visit	Start date and time	Duration	CS worsening
xxxxxx	xx-xxxx	xxx	ddMMMyyyy hh:mm	hh:mm	Yes/No
		xxx	ddMMMyyyy hh:mm	hh:mm	Yes/No
		xxx	ddMMMyyyy hh:mm	hh:mm	Yes/No
Etc					

Page x/y

Path\Filename

16 STUDY FLOW CHART

Study assessment & exams	Baseline	RANDOMISATION	H0 ± 10 min	H12 ± 10 min	H24 ± 10 min	H36 ± 10 min	H48 ± 10 min	H60 ± 10 min	H72 ± 10 min	H84 ± 10 min	D8 (after H0) +2 days	D21 (after H0) +2 days	Premature Discont.
Informed consent signed	X												
Check Incl./excl. Criteria	X												
Medical history	X												
Demographics (age, gender)	X												
Weight, height & BMI	X												
Viral serologies and/or PCR (HAV, HBV, HCV, HDV, HEV, HSV,VZV, HIV)	X												
Acetaminophen, alcohol plasma levels	X												
Cupremia, ceruloplasmin plasma levels, and cupruria	X												
Autoimmune markers (ANA, ASMA, anti-LKM Ab, Ig levels)	X												
Beta hCG blood pregnancy test for women of childbearing potential only	X											X	X
ALF-5755/ Placebo infusions			Within 48 hrs after baseline	X	X	X	X	X					
Clinical examination	X		Before, 10 min after infusion	Before, 10 min after infusion	Before, 10 min after infusion	Before, 10 min after infusion	Before, 10 min after infusion	Before, 10 min after infusion	X	X	X	X	X
Vital signs (BP, HR, RR)	X		Before infusion, 10 min + HR and BP only during 1 hr after infusion	Before infusion, 10 min + HR and BP only during 1 hr after infusion	Before infusion, 10 min + HR and BP only during 1 hr after infusion	Before infusion, 10 min + HR and BP only during 1 hr after infusion	Before infusion, 10 min + HR and BP only during 1 hr after infusion	Before infusion, 10 min + HR and BP only during 1 hr after infusion	X	X	X	X	X
Body temperature	X		Before infusion	Before infusion	Before infusion	Before infusion	Before infusion	Before infusion	X	X	X	X	X
ECG	X		Before, 10 min and 1 hr after infusion		Before, 10 min and 1 hr after infusion		Before, 10 min and 1 hr after infusion		X	X	X	X	X
Hepatic Encephalopathy grade & GCS	X		Before infusion	Before infusion	Before infusion	Before infusion	Before infusion	Before infusion	X	X	X	X	X
Arterial blood gas with lactates: if grade I or II hepatic encephalopathy or GCS < 15													
PR, FV, INR, ALT, AST	X		Before infusion	Before infusion	Before infusion	Before infusion	Before infusion	Before infusion	X	X	X	X	X
Haematology, Biochemistry	X		Before infusion	Before infusion	Before infusion	Before infusion	Before infusion	Before infusion	X	X	X	X	X
Urinalysis			Before infusion		Before infusion		Before infusion		X		X	X	X
Anti-ALF-5755 antibodies[1]			Before infusion								X	X	

Concomitant medications & AE recording (from signed PICD)	X		X	X	X	X	X	X	X	X	X	X	X
CK18 (only at Paul Bousse and Beaujo[illegible] hospital)			Before infusion	Before infusion	Before infusion	Before infusion	Before infusion	Before infusion	X	X			X

ALF-5755 plasma concentrations:

**H0**	**H0 10 min**	**H1**	**H2**	**H6**	**H12**	**H48**	**H60**	**H60 10 min**	**H61**	**H62**	**H66**	**H72**	**H84**	**Premature discont.**
X just before 1st infusion	X end of 1st infusion	X	X	X	X just before 2nd infusion	X just before 5th infusion	X just before 6th infusion	X end of 6th infusion	X	X	X	X	X	X
