# Supplementary material for: A Proof of Concept, Phase II Randomized European Trial, on the Efficacy of ALF-5755, a Novel Extracellular Matrix-Targeted Antioxidant in Patients with Acute Liver Diseases
Source: PLoS One. 2016 Mar 16;11(3):e0150733. doi: 10.1371/journal.pone.0150733 (PMC4794150; doi:10.1371/journal.pone.0150733)
Supplement: S7 Text — (PDF) [file pone.0150733.s009.pdf]

CLINICAL STUDY REPORT

1 TITLE PAGE

**Study Title:**	A multicentre, double-blind, randomized, placebo-controlled study to evaluate the efficacy and the safety of ALF-5755 in patients with nonacetaminophen severe acute hepatitis and early stage acute liver failure.
**Investigational Product:**	ALF-5755
**Indication Studied:**	Nonacetaminophen severe acute hepatitis (SAH) and early stage acute liver failure (ALF)
**Study Design:**	Prospective, multicentre, double-blind, placebo-controlled study of ALF-5755 in patients with nonacetaminophen SAH and early stage ALF, randomized in the ratio 1:1
**Name of Sponsor:**	Alfact Innovation<br>320 rue Saint Honoré, 75001 Paris, France
**Protocol Number:**	ALF-5755_P2_ALF
**Development Phase:**	II
**Studied Period:**	First Patient Screened: 20-Oct-2010<br>First Patient Enrolled: 20-Oct-2010<br>Last Patient Completed: 24-Apr-2013
**Principal Investigator:**	Didier Samuel, MD PhD<br>Centre Hépatobiliaire<br>Hôpital Paul Brousse<br>Villejuif, France
**Sponsor Signatory:**	Paul Amouyal, MD<br>President<br>Alfact Innovation<br>Tel: +33 1 45 59 35 66<br>Fax: + 33 1 53 40 60 52
**GCP Compliance:**	This study and the archiving of essential documents were performed in compliance with Good Clinical Practice (GCP).
**Version/Date of Report:**	Final Version 01/16 December 2013

Confidentiality Statement

This confidential document is the property of Alfact Innovation. No unpublished conformation contained herein may be disclosed without prior written approval from Alfact Innovation. Access to this document must be restricted to relevant parties.

ORION
Clinical Services Ltd.
7 Bath Road
Slough
BERKS
SL1 3UA
UK

SIGNATURE PAGE

A multicentre, double-blind, randomized, placebo-controlled study to evaluate the efficacy and the safety of ALF-5755 in patients with nonacetaminophen severe acute hepatitis and early stage acute liver failure

I have read this report and confirm that to the best of my knowledge it accurately describes the conduct and results of the study.

Sponsor Representatives

Dr Gilles Amouyal
Chief Executive Officer
Alfact Innovation
320 rue Saint Honoré
75001, Paris
France

_______________ _________________________

Date Signature

Paul Amouyal, MD
President
Alfact Innovation
320 rue Saint Honoré
75001, Paris
France

_______________ _________________________

Date Signature

Principal Investigator

Didier Samuel, MD PhD
Centre Hépatobiliaire
Hôpital Paul Brousse
12 Avenue Paul-Vaillant-Couturier
94800, Villejuif
France

_______________ _________________________

Date Signature

Biostatistician
Dr Bertrand Nalpas
Director of Research
Paris Biopark
7 rue Watt
75013, Paris
France

Date Signature

Medical Writer
Matthew Davis
ORION Clinical Services Ltd.
Head of Regulatory and Medical Writing
7 Bath Road, Slough
Berkshire
SL1 3UA
UK

Date Signature

Biostatistician
Dr Bertrand Nalpas
Director of Research
Paris Biopark
7 rue Watt
75013, Paris
France

Date Signature

Medical Writer
Matthew Davis
ORION Clinical Services Ltd.
Head of Regulatory and Medical Writing
7 Bath Road, Slough
Berkshire
SL1 3UA
UK

20 DEC 2013
Date Signature

Name of Sponsor/Company: Alfact Innovation<br>Name of Finished Product: ALF-5755<br>Name of Active Ingredient: recombinant human hepatocarcinoma-intestine-pancreas/pancreatitis-associated protein (rhHIP/PAP)	Individual Study Table Referring to Part of the Dossier<br><br>Volume<br>Page	(For National Authority Use only)

email: aabergel@chu-clermontferrand.fr	phone: 04 26 10 93 39,<br>email: si-nafaa.si-ahmed@chu-lyon.fr<br>(PI until 25-Jun-2013)<br>New PI: Docteur Sylvie RADENNE, Principal Investigator, Hôpital Croix-Rousse,<br>103 grande rue de la croix Rousse,<br>Service Hépatologie et Gastro-Entérologie,<br>Service du Pr Zoulim Bâtiment R, 69317,<br>Lyon cedex 04,<br>phone: 04 26 10 93 59, fax: 04 26 73 26 76,<br>email: sylvie.radenne@chu-lyon.fr
Site 07:<br>Professeur Dominique LARREY, Principal Investigator, Hôpital Saint-Eloi, 80, avenue Augustin Fliche,<br>Pôle Digestif, Service Hépato-Gastro-Entérologie, 34295, Montpellier Cedex 5,<br>phone: +33.4.67.33.70.62, fax: +33.4.67.33.02.57,<br>email: dom-larrey@chu-montpellier.fr	Site 08:<br>Professeur Jean GUGENHEIM, Principal Investigator, Hôpital de l'Archet 2,<br>151 Route Saint Antoine Ginestiere,<br>Service de Chirurgie Digestive, 06202, Nice,<br>phone: +33.4.92.03.64.76,<br>email: gugenheim.j@chu-nice.fr<br>(Site closed on 05-Sep-2011. No pt included)
Site 09:<br>Professeur Danielle BOTTA FRIDLUND,<br>Principal Investigator,<br>Hôpital Conception, 147, boulevard Baille, Service Hépato-Gastro-Entérologie et Pancréatologie, 13385, Marseille Cedex ,<br>phone: +33.4.91.38.36.95 ou 96, fax: +33.4.91.38.36.92,<br>email: danielle.botta@ap-hm.fr<br>(Site closed on 19-Sep-2011. No pt included)	Site 10:<br>Professeur Vincent Di MARTINO,<br>Principal Investigator,<br>CHU de Besançon, Hôpital Jean Minjoz, 3,<br>bd Alexandre Fleming,<br>Service d'Hépatologie, 25030, Besançon Cedex,<br>phone: +33.3.81.66.84.21 / +33.3.81.66.93.26,<br>fax: +33.3.81.66.84.17,<br>email: vdimartino@chu-besancon.fr<br>(Site closed on 01-Sep-2011. No pt included)
Site 11:<br>Docteur Vincent LEROY, Principal Investigator,<br>CHU de Grenoble, BP 217,<br>Département d'Hépato-Gastroentérologie, 38043, Grenoble Cedex 9,<br>phone: +33.4.76.76.93.68, fax: +33.4.76.76.51.79,	Site 12:<br>Professeur Philippe MATHURIN,<br>Principal Investigator,<br>Hôpital Claude Huriez, Rue Polonovski,<br>Service des maladies de l'appareil digestif,<br>1e étage - Aile EST, 59037, Lille cedex,

**Name of Sponsor/Company:**<br>Alfact Innovation	**Individual Study Table Referring to Part of the Dossier**<br><br>**Volume**<br><br>**Page**	**(For National Authority Use only)**
**Name of Finished Product:**<br>ALF-5755		
**Name of Active Ingredient:**<br>recombinant human hepatocarcinoma-intestine-pancreas/pancreatitis-associated protein (rhHIP/PAP)		

email: vleroy@chu-grenoble.fr	phone: 03.20.44.68.91, fax: +33.3.20.44.55.64,<br>email: philippe.mathurin@chru-lille.fr
Site 14:<br>Docteur Christophe CAMUS Principal Investigator,<br>Hôpital Pontchaillou, CHRU Rennes,<br>2 Rue Henri Le Guilloux,<br>Service des maladies infectieuses, 35033,<br>Rennes Cedex 9,<br>phone: +33 2 99 28 42 48, fax: +33 2 99 28 41 64,<br>email: christophe.camus@chu-rennes.fr	Site 15:<br>Professeur Christophe DUVOUX,<br>Principal Investigator, CHU Henri Mondor,<br>51 Avenue Marechal de Lattre de Tassigny,<br>Service d'Hépato-Gastro-Entérologie, 94010, Créteil,<br>email: christophe.duvoux@hmn.aphp.fr
Site 13:<br>Docteur Anne GUILLYGOMARC'H,<br>Principal Investigator, CHU de Rennes,<br>Hôpital Pontchaillou, 2 Rue Henri Le Guilloux,<br>Service des Maladies du Foie, 35033, Rennes Cedex 9,<br>email: brissot@univ-rennes1.fr<br>(investigator declined on 8-July-2010. No SIV done)	Site 20:<br>Prof. Dr. Guido GERKEN, Principal Investigator,<br>Universitätsklinikum Essen, Hufelandstraße 55,<br>Medizinisches Zentrum,<br>Klinik für Gastroenterologie und Hepatologie,<br>Studienambulanz, 1.OG, Raum 1.245, D-45122, Essen,<br>phone: +49.201.723-3610, fax: +49.201.723-5971,<br>email: guido.gerken@uk-essen.de
Site 21:<br>Prof. Dr. Christian TRAUTWEIN,<br>Principal Investigator, Medizinische Klinik III,<br>Universitätsklinikum Aachen, Pauwelsstr. 30,<br>Studienzentrum Viszeralmedizin, Ebene 3, Flur 30,<br>Raum 3, z. Hd. Frau A. Schroeder, D-52074, Aachen,<br>phone: +49.241.80-80866, fax: +49.241.80-82455,<br>email: ctrautwein@ukaachen.de	Site 22:<br>Prof. Dr. Michael P. MANNS, Principal Investigator,<br>Medizinische Hochschule Hannover,<br>Zentrum Innere Medizin, Carl-Neuberg-Str. 1,<br>Klinik für Gastroenterologie,<br>Hepatologie und Endokrinologie, D-30625, Hannover,<br>phone: +49.511.532-3305, fax: +49-511-532-4896,<br>email: Manns.Michael@mh-hannover.de
**Publication (Reference):**<br>Not applicable	
**Study Period:**<br>20-Oct-2010 (First Patient Screened) to<br>24-Apr-2013 (Last Patient Completed)	**Phase of Development:**<br>Phase II

**Name of Sponsor/Company:** Alfact Innovation	**Individual Study Table Referring to Part of the Dossier**	**(For National Authority Use only)**
**Name of Finished Product:** ALF-5755	**Volume**	
**Name of Active Ingredient:** recombinant human hepatocarcinoma-intestine-pancreas/pancreatitis-associated protein (rhHIP/PAP)	**Page**	

Objectives:

Primary objective:

- To evaluate the efficacy of ALF-5755 versus placebo, measured by rate of change of prothrombin ratio (PR) during the 72 hours following treatment initiation, in patients with nonacetaminophen SAH and early stage ALF.

Secondary objectives:

- To evaluate the safety and tolerability of ALF-5755 versus placebo in patients with nonacetaminophen SAH and early stage ALF;
- To determine the pharmacokinetic (PK) parameters of ALF-5755 versus placebo in patients with nonacetaminophen SAH and early stage ALF.

Methodology and Criteria for Evaluation:

This was a prospective, multicentre, double-blind, placebo-controlled study of ALF-5755 in patients with nonacetaminophen SAH and early stage ALF, randomized in the ratio 1:1. A minimum of 60 patients were to be recruited into the study in the following two treatment groups:

- Group A: approximately 30 patients received ALF-5755;
- Group B: approximately 30 patients received placebo (physiological saline solution: 0.9% NaCl).

Patients received the investigational product (IP) (either placebo or ALF-5755) every 12 hours over 3 days. The time between biological baseline assessment and first injection of ALF-5755 or placebo was to be as short as possible, but not to exceed 48 hours.

Patients were to continue to receive (in addition to ALF-5755 or placebo) the best standard treatment upon assessment by the Investigators.

The statistical analysis was based on the following analysis data sets:

- Safety Population: all patients who were randomised to treatment and received at least

**Name of Sponsor/Company:**<br>Alfact Innovation	**Individual Study Table Referring to Part of the Dossier**<br><br>**Volume**<br>**Page**	**(For National Authority Use only)**
**Name of Finished Product:**<br>ALF-5755		
**Name of Active Ingredient:**<br>recombinant human hepatocarcinoma-intestine-pancreas/pancreatitis-associated protein (rhHIP/PAP)		

one administration of ALF-5755 or placebo. Patients who received the wrong treatment were to be analysed as treated when using the Safety Population;

- Intention-to-treat (ITT) Population: all patients in the Safety Population who provided at least one post-dose efficacy assessment. Note that patients who withdrew early due to worsening disease before the first post-baseline efficacy assessment were to be included as their efficacy assessments were to be imputed. Patients who received the wrong treatment were to be analysed as randomised when using the ITT Population;
- Per-protocol (PP) Population: all patients in the ITT Population who completed the double blind treatment phase of the study with no major protocol violations. Protocol violations were to be defined in the SAP, and discussed and agreed in a blind review meeting prior to database lock;
- All patients who received the complete treatment (i.e., patients who received 6 infusions). This population was planned and defined after database lock.

<u>Number of patients:</u>

Planned: A minimum of 60 patients (approximately 30 patients per group)

Analysed:

- 59 patients randomised, of which 57 patients were treated (2 patients were randomised by mistake and did not receive treatment); 56 patients were included in the per protocol population;
- 45 patients completed the study;
- 22 patients with Viral Hepatitis B (HBV) or Autoimmune Hepatitis (AIH).

<u>Efficacy:</u>

All efficacy variables and endpoints were predefined in the study protocol and/or the statistical analysis plan (SAP).

**Name of Sponsor/Company:**<br>Alfact Innovation	**Individual Study Table Referring to Part of the Dossier**<br><br>**Volume**<br>**Page**	**(For National Authority Use only)**
**Name of Finished Product:**<br>ALF-5755		
**Name of Active Ingredient:**<br>recombinant human hepatocarcinoma-intestine-pancreas/pancreatitis-associated protein (rhHIP/PAP)		

Primary variable:

- Rate of change of PR during 72 hours following treatment initiation. PR was measured and recorded on the CRF at baseline, H0 before 1st infusion, H12 before 2nd infusion, H24 before 3rd infusion, H36 before 4th infusion, H48 before 5th infusion, H60 before 6th infusion, H72, H84, D8 (after H0), D21 (after H0) or premature discontinuation.

Secondary variables:

- Rate of change of MELD during 72 hours following treatment initiation and raw values at various time-point of model for end-stage liver disease (MELD);
- Rate of change of Factor V (FV) during 72 hours following treatment initiation;
- Rate of change of International Normalised Ratio (INR) during 72 hours following treatment initiation;
- Rate of change of Alanine Transaminase (ALT) plasma level during 72 hours following treatment initiation;
- Rate of change of Aspartate Transaminase (AST) plasma level during 72 hours following treatment initiation;
- Change of Hepatic Encephalopathy Grade (HE grade) during 72 hours following treatment initiation;
- Overall survival rate at Day 21;
- Transplant-free survival rate at Day 21;
- Liver transplant rate at Day 21;
- Hospital-free days;
- Length of hospitalisation.

Exploratory variables:

- Total and cleaved Cytokeratin (CK) 18 plasma levels. Rate of change of CK 18 was

**Name of Sponsor/Company:** Alfact Innovation	**Individual Study Table Referring to Part of the Dossier** **Volume** **Page**	**(For National Authority Use only)**
**Name of Finished Product:** ALF-5755		
**Name of Active Ingredient:** recombinant human hepatocarcinoma-intestine-pancreas/pancreatitis-associated protein (rhHIP/PAP)		

defined as for the primary endpoint including the modifications other than LNR. Other biomarkers such as GCglobulin and total bilirubin at inclusion;

Subgroup analysis

Subgroups were defined based on initial PR, hepatitis etiology, a combination of HBV and AIH etiologies, hepatic encephalopathy and N-acetyl cysteine (NAC) treatment (from the concomitant medication page of the CRF).

Additional subgroup analysis was requested for HBV and AIH patients following database lock:

- PR 0-72h - Population who received 6 infusions of treatment.

Complementary analyses

After the unblinding of the study data, Alfact decided to have additional efficacy analyses conducted for different endpoints. Variables included:

- Rate of change of PR from 0-12 h, i.e. early efficacy;
- Other time-points for efficacy markers (Day 8 and Day 21); i.e. long term efficacy;
- Comparison of patient liver function status at inclusion according to population (ALF-5755 versus placebo in HBV AIH subgroup);
- Time for PR to reach 50%.

Pharmacokinetics (PK):

C_{max} (ng/mL); T_{max} (h); $t_{1/2}$ (h); AUC_{0-12} (ng/mL.h); AUC_{0-t} (ng/mL.h).

Safety:

Safety assessments involved: the comparison of: local and systemic AEs (AEs/SAEs) (AEs were to be recorded from the time point that the PICD had been signed); assessment of: laboratory findings (including haematology, biochemistry and urinalysis); clinical examination, clinically significant vital signs (BP, HR, RR), body temperature; HE grades and Glasgow Coma Scores (GCSs); ECG abnormalities; anti-ALF-5755 antibodies plasma levels; beta hCG

**Name of Sponsor/Company:**<br>Alfact Innovation	**Individual Study Table Referring to Part of the Dossier**<br><br>**Volume**<br>**Page**	**(For National Authority Use only)**
**Name of Finished Product:**<br>ALF-5755		
**Name of Active Ingredient:**<br>recombinant human hepatocarcinoma-intestine-pancreas/pancreatitis-associated protein (rhHIP/PAP)		

blood pregnancy test in women of childbearing potential; arterial blood gas with lactate at H24 before infusion, H48 before infusion, H72 in case of grade I or II HE or GCS < 15 or if required from Investigator's assessment or in case of abnormality at screening.

Diagnosis and Main Criteria for Inclusion:

Patients included were those with early stage ALF or SAH aged ≥ 18 to ≤ 75 years; patients excluded were those with: acetaminophen-induced hepatitis; shock liver (ischemic hepatopathy) or HELLP syndrome or Budd-Chiari syndrome or intrahepatic malignancy; serum creatinine ≥ 180 µmol/L; body mass index (BMI) ≥ 35; septic shock requiring administration of inotropic drugs; uncontrolled active bleeding; who were receiving fresh frozen plasma/vitamin K infusion in the preceding 24 hours/liver support device treatment or haemodialysis; had intractable arterial hypotension; positive for HIV; had active cancer; were pregnant or lactating women; surgery 4 weeks prior to baseline/organ transplant or allograft.

Test Product, Dose and Mode of Administration, Batch Number:

ALF-5755 was supplied lyophilised in a glass vial with halobutyl stopper and aluminium seal. The vial was stoppered under nitrogen with partial vacuum. Each vial contained 2.80 mg of ALF-5755. Reconstitution in the vial with 6.6 mL of water for injection gave a clear colourless solution at 0.40 mg/mL of ALF-5755. Needles and syringes were supplied for use during the dilution procedure. A total of 4 vials were required for each 10 minute slow infusion.

The following batch of ALF-5755 was used for dosing: IMP Batch no. PX7-F8-ALF-5755 (as stated in the IMP labels).

Duration of Treatment:

A total of 10 mg (25 mL) was administered every 12 hours over 3 days.

ALF-5755, and placebo were administered as slow intravenous infusions, each a 10-minute infusion of 10 mg (25 mL) using automatic syringes every 12 hours from H0 until H60 (H0, H12, H24, H36, H48 and H60, all ± 10 minutes).

**Name of Sponsor/Company:**<br>Alfact Innovation	**Individual Study Table Referring to Part of the Dossier**<br><br>**Volume**<br>**Page**	**(For National Authority Use only)**
**Name of Finished Product:**<br>ALF-5755		
**Name of Active Ingredient:**<br>recombinant human hepatocarcinoma-intestine-pancreas/pancreatitis-associated protein (rhHIP/PAP)		

Reference Therapy, Dose and Mode of Administration, Batch Number:

Placebo, physiological saline solution (0.9% NaCl), has been chosen as the comparator. The physiological saline solution vials was provided by an independent person in charge of study drug reconstitution at each centre.

Placebo not provided by the sponsor.

Statistical Methods:

Rate of change of PR, MELD, FV, INR, ALT and AST during 72 hours following treatment initiation in ALF-5755 and placebo arms were compared by a non parametric test (Mann-Whitney-Wilcoxon) and the Cochran-Mantel-Haenszel test adjusting for aetiology.

HE grade was compared using the Cochran-Mantel-Haenszel test unadjusted and adjusting for aetiology.

Mortality and liver transplantation frequencies were compared by a Chi-squared test and their time-to-event by a log-rank test and using the Cox proportional hazards model to adjust for aetiology.

Hospital-free days (and length of hospitalisation) were compared using the Mann-Whitney-Wilcoxon test.

The achievement of steady-state was assessed by comparison of ALF-5755 concentrations on H48, H60 and H72. Accumulation was assessed by comparison of AUC_{0-12} between H0 and H12 and AUC_{0-12} between H60 and H72.

Summary – Results and conclusions:

Efficacy:

ITT population:

<u>Primary variables:</u>

The analysis of PR from 0 to 72 hours showed no significant changes or obvious effect for the ITT population with complete treatment.

**Name of Sponsor/Company:** Alfact Innovation	**Individual Study Table Referring to Part of the Dossier**	**(For National Authority Use only)**
**Name of Finished Product:** ALF-5755		
**Name of Active Ingredient:** recombinant human hepatocarcinoma-intestine-pancreas/pancreatitis-associated protein (rhHIP/PAP)	**Volume** **Page**	

Secondary variables:

No significant changes or obvious effects were seen for patients in the ITT population treated with ALF-5755 versus placebo for MELD, INR, transplant-free survival and total and cleaved levels of CK18.

Similarly, no significant changes were noted in the number of hospital-free days in the ALF-5755 group compared to the placebo group.

For the ITT population (alive with no transplant), the mean number of days in the hospital was lower the ALF-5755 group (9.14 days) compared to the placebo group (12.00 days). The difference in days in hospital was not significant at the 5% level for the Wilcoxon test (p=0.169).

Days in Hospital (ITT Population, Alive with No Liver Transplant)

		**ALF-5755 (N=21)**	**Placebo (N=25)**	**p-value**
Days in Hospital up to D21[1]	N	21	25	
	Mean (SD)	9.14 (4.63)	12.00 (6.36)	
	Median	8	11	
	Range	5 - 21	4 - 21	
	Wilcoxon test			0.169

Source: Table 14.2.5.2

[1] Up to and including date of hospital discharge, maximum of 21 days.

Complementary analyses:

Early efficacy:
Median values recorded for analysis of PR 0-12 hours for the ITT Population showed an increase in ALF-5755 treated group (0.292) when compared to Placebo group (0.167), although of no significance due to large standard deviations.

**Name of Sponsor/Company:** Alfact Innovation	**Individual Study Table Referring to Part of the Dossier**	**(For National Authority Use only)**
**Name of Finished Product:** ALF-5755	**Volume**	
**Name of Active Ingredient:** recombinant human hepatocarcinoma-intestine-pancreas/pancreatitis-associated protein (rhHIP/PAP)	**Page**	

Analysis of PR (0-12 hours) (ITT Population)

		**ALF-5755 (N=28)**	**Placebo (N=29)**	**p-value**
Rate of change of PR	N	27	29	
	Mean (SD)	0.356 (0.419)	0.187 (0.517)	
	Median	0.292	0.167	
	Range	-0.17- 1.58	-1.00- 1.42	
Wilcoxon test				0.1724
CMH test adjusting for aetiology[1]				0.0585

[1] Hepatitis etiology factor levels: Viral Hepatitis A; Viral Hepatitis B or Autoimmune Hepatitis; Other
Source: Table 14.2.1.1.23
SD: Standard deviation

HBV/AIH subgroup:

<u>Primary variables:</u>

Median values recorded in the analysis of PR (0-72 hours) in the ITT Population with HBV or AIH showed an increase in the primary endpoint for ALF-5755 versus placebo, although of no significance (0.028 versus -0.042).

Analysis of PR (0-72 hours) (ITT Population with HBV or AIH)

Name of Sponsor/Company: Alfact Innovation	Individual Study Table Referring to Part of the Dossier	(For National Authority Use only)
Name of Finished Product: ALF-5755	Volume	
Name of Active Ingredient: recombinant human hepatocarcinoma-intestine-pancreas/pancreatitis-associated protein (rhHIP/PAP)	Page	

		ALF-5755 (N=13)	Placebo (N=9)	p-value
Rate of change of PR	N	13	9	
	Mean (SD)	0.000 (0.118)	-0.032 (0.096)	
	Median	0.028	-0.042	
	Range	-0.25 - 0.14	-0.25 - 0.08	
Wilcoxon test				0.2696
CMH test				0.4956

Source: Table 14.2.1.1.1

Median values recorded in the analysis of PR (0-72 hours) in the ITT Population with HBV or AIH who received completed treatment showed an increase in the primary endpoint for ALF-5755 versus placebo (0.042 versus -0.042) that can be considered significant (p=0.0449).

Analysis of PR (0-72 hours) (ITT Population with HBV or AIH and who received 6 infusions of treatment)

		ALF-5755 (N=10)	Placebo (N=8)	p-value
Rate of change of PR	N	10	8	
	Mean (SD)	0.046 (0.067)	-0.040 (0.099)	
	Median	0.042	-0.042	
	Range	-0.08- 0.14	-0.25- 0.08	
Wilcoxon test				0.0449
CMH test adjusting for aetiology[1]				0.0474

Source: Table 14.2.1.1.20

SD: Standard deviation

Secondary variables:

**Name of Sponsor/Company:**<br>Alfact Innovation	**Individual Study Table Referring to Part of the Dossier**<br><br>**Volume**<br>**Page**	**(For National Authority Use only)**
**Name of Finished Product:**<br>ALF-5755		
**Name of Active Ingredient:**<br>recombinant human hepatocarcinoma-intestine-pancreas/pancreatitis-associated protein (rhHIP/PAP)		

No significant changes or obvious effects were seen for patients in the ITT population with HBV or AIH treated with ALF-5755 versus placebo for MELD, INR, and total and cleaved levels of CK18.

There was no significant difference in transplant-free survival between the ALF-5755- and the placebo groups; however, Baseline values could be considered a factor here since the highest number of patients with total bilirubin >500 at inclusion were in the ALF-5755 group .

Stratification of patients according to GCglobulin showed that no liver transplants were required in patients treated with ALF-5755 group if GCglob>155.

Plot of Baseline GCGlobulin (ITT Population with HBV or AIH)

**Name of Sponsor/Company:** Alfact Innovation	**Individual Study Table Referring to Part of the Dossier** **Volume** **Page**	**(For National Authority Use only)**
**Name of Finished Product:** ALF-5755		
**Name of Active Ingredient:** recombinant human hepatocarcinoma-intestine-pancreas/pancreatitis-associated protein (rhHIP/PAP)		

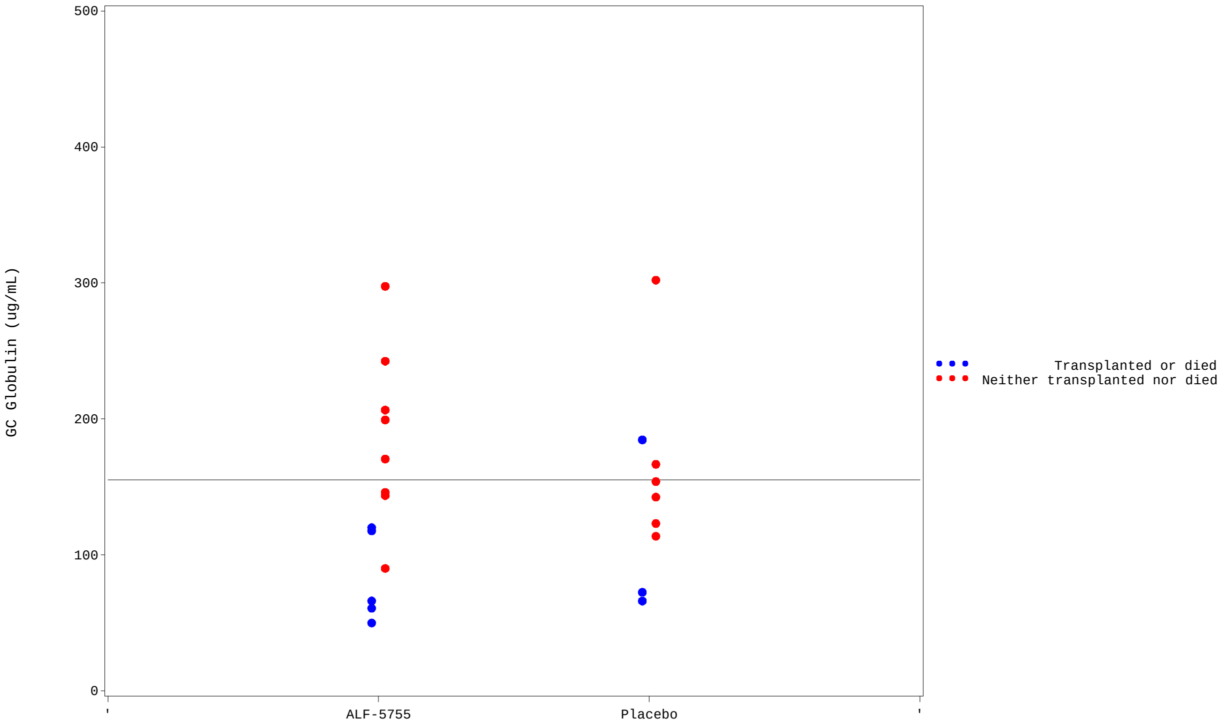

Source: Figure 6.2

In addition, stratification of patients according to total bilirubin showed similar results: that no liver transplants were required in patients treated with ALF-5755 group if Total Bilirubin<400.

Plot of Baseline Total Bilirubin (ITT Population with HBV or AIH)

Name of Sponsor/Company: Alfact Innovation	Individual Study Table Referring to Part of the Dossier	(For National Authority Use only)
Name of Finished Product: ALF-5755	Volume	
Name of Active Ingredient: recombinant human hepatocarcinoma-intestine-pancreas/pancreatitis-associated protein (rhHIP/PAP)	Page	

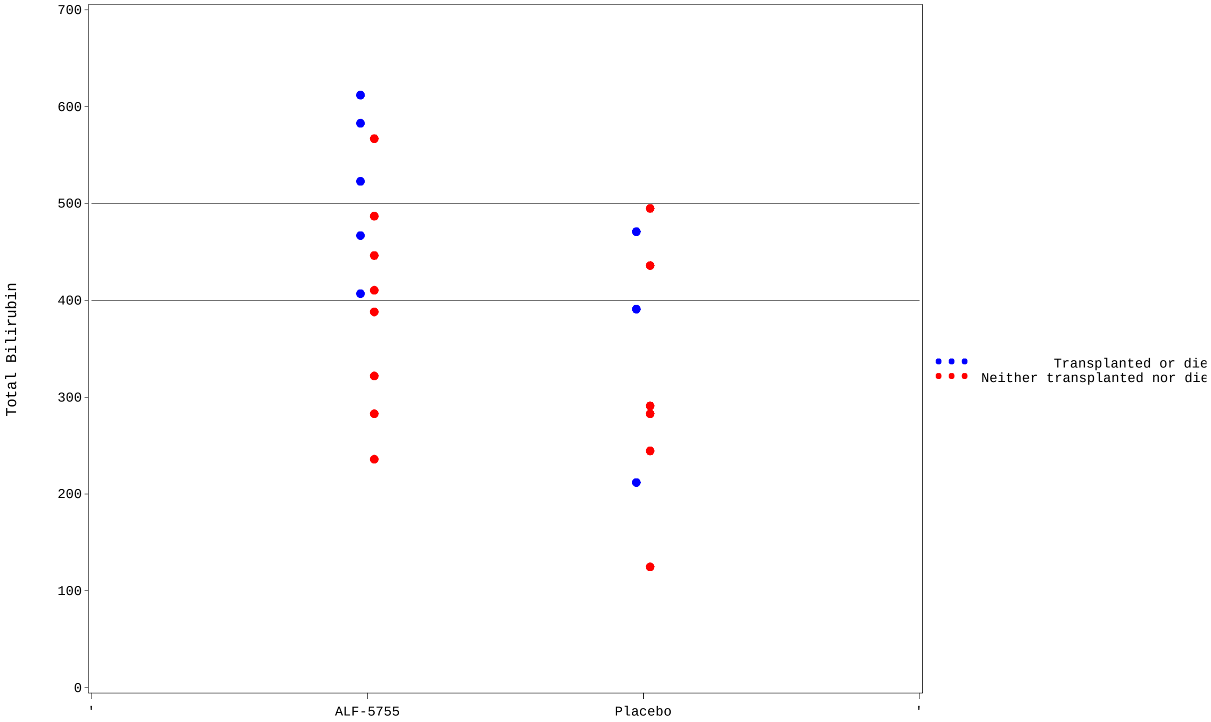

Source: Figure 6.1

In the ITT Population with HBV or AIH (alive with no liver transplant), the mean number of days in the hospital was lower the ALF-5755 group (10 days) compared to the placebo group (17.17 days). The difference in days in hospital was significant at the 5% level for the Wilcoxon test (p=0.023).

Days in Hospital (ITT Population with HBV or AIH, Alive with No Liver Transplant)

**Name of Sponsor/Company:**<br>Alfact Innovation	**Individual Study Table Referring to Part of the Dossier**<br><br>**Volume**<br><br>**Page**	**(For National Authority Use only)**
**Name of Finished Product:**<br>ALF-5755		
**Name of Active Ingredient:**<br>recombinant human hepatocarcinoma-intestine-pancreas/pancreatitis-associated protein (rhHIP/PAP)		

		**ALF-5755 (N=8)**	**Placebo (N=6)**	**p-value**
Days in Hospital up to D21[1]	N	8	6	
	Mean (SD)	10 (4.34)	17.17 (5.27)	
	Median	8.5	19.5	
	Range	5 - 18	8 - 21	
	Wilcoxon test			0.023

Source: Table 14.2.5.4

[1] Up to and including date of hospital discharge, maximum of 21 days.

<u>Complementary analyses:</u>

Early efficacy: Median values recorded in the analysis of PR (0-12 hours) in the ITT Population with HBV or AIH showed a significant increase in the primary endpoint (0.167 versus -0.167 for placebo; p=0.0385).

Analysis of PR (0-12 hours) (ITT Population with HBV or AIH)

		**ALF-5755 (N=13)**	**Placebo (N=9)**	**p-value**
Rate of change of PR	N	12	9	
	Mean (SD)	0.177 (0.246)	-0.167 (0.400)	
	Median	0.167	-0.167	
	Range	-0.17- 0.67	-1.00- 0.25	
Wilcoxon test				0.0385
CMH test adjusting for aetiology[1]				0.0291

Source: Table 14.2.1.1.18

SD: Standard deviation

In the ITT Population with HBV or AIH and who received 6 infusions of treatment, median

Name of Sponsor/Company: Alfact Innovation	Individual Study Table Referring to Part of the Dossier	(For National Authority Use only)
Name of Finished Product: ALF-5755	Volume	
Name of Active Ingredient: recombinant human hepatocarcinoma-intestine-pancreas/pancreatitis-associated protein (rhHIP/PAP)	Page	

values recorded in the analysis of PR (0-12 hours) showed an increase in primary endpoint (0.250 versus -0.208 for placebo; p=0.0295).

Analysis of PR (0-12 hours) (ITT Population with HBV or AIH and who received 6 infusions of treatment)

		ALF-5755 (N=10)	Placebo (N=8)	p-value
Rate of change of PR	N	9	8	
	Mean (SD)	0.153 (0.219)	-0.198 (0.415)	
	Median	0.250	-0.208	
	Range	-0.17- 0.42	-1.00- 0.25	
Wilcoxon test				0.0295
CMH test adjusting for aetiology[1]				0.0469

Source: Table 14.2.1.1.26

SD: Standard deviation

Long term efficacy: Median values recorded for the analysis of PR 0-Day 8 for patients in the ITT population with HBV or AIH showed an increase in ALF-5755 treated group (0.068) when compared to Placebo group (0.010) although of no significance.

Analysis of PR (0-Day 8) (ITT Population with HBV or AIH)

Name of Sponsor/Company: Alfact Innovation	Individual Study Table Referring to Part of the Dossier	(For National Authority Use only)
Name of Finished Product: ALF-5755	Volume	
Name of Active Ingredient: recombinant human hepatocarcinoma-intestine-pancreas/pancreatitis-associated protein (rhHIP/PAP)	Page	

		ALF-5755 (N=13)	Placebo (N=9)	p-value
Rate of change of PR	N	13	9	
	Mean (SD)	0.063 (0.154)	0.022 (0.075)	
	Median	0.068	0.010	
	Range	-0.25- 0.26	-0.07- 0.20	
Wilcoxon test				0.1088
CMH test adjusting for aetiology[1]				0.4601

Source: Table 14.2.1.1.28

SD: Standard deviation

In the ITT Population in patients with HBV or AIH and who received the complete treatment, median values recorded for the analysis of PR 0-Day 8 showed an increase in the ALF-5755 treated group (p=0.0067).

Analysis of PR (0-Day 8) (ITT Population with HBV or AIH and who received 6 infusions of treatment)

		ALF-5755 (N=10)	Placebo (N=8)	p-value
Rate of change of PR	N	10	8	
	Mean (SD)	0.127 (0.086)	0.021 (0.081)	
	Median	0.099	0.010	
	Range	0.02- 0.26	-0.07- 0.20	
Wilcoxon test				0.0067
CMH test adjusting for aetiology[1]				0.0216

Source: Table 14.2.1.1.30

SD: Standard deviation

All biological markers for liver function for the ITT Population with HBV or AIH show that

Name of Sponsor/Company: Alfact Innovation	Individual Study Table Referring to Part of the Dossier	(For National Authority Use only)
**Name of Finished Product:** ALF-5755	Volume	
**Name of Active Ingredient:** recombinant human hepatocarcinoma-intestine-pancreas/pancreatitis-associated protein (rhHIP/PAP)	Page	

patients assigned to the ALF-5755 arm were in a more severe condition at baseline than those in the placebo arm.

Baseline biological markers for liver function: INR, MELD, ALT, AST, Total Bilirubin, PR (ITT Population with HBV or AIH)

		ALF-5755 (N=13)	Placebo (N=9)
INR	Mean (SD)	2.6015 (0.9989)	2.4756 (0.9889)
	Range	1.500 - 4.530	1.200- 3.990
MELD	Mean (SD)	29.98 (6.19)	26.62 (5.95)
	Range	22.8- 41.5	16.0- 34.0
ALT	Mean (SD)	2307.8 (1781.6)	1491.2 (912.3)
	Range	369- 5548	537- 2899
AST	Mean (SD)	1535.0 (1111.8)	1149.2 (546.6)
	Range	350- 3490	376- 2167
Conjugated Bilirubin	Mean (SD)	314.8 (84.0)	230.0 (91.8)
	Range	151 - 433	99 - 372
Total Bilirubin	Mean (SD)	440.9 (115.8)	327.6 (127.0)
	Range	236- 612	125- 495
PR	Mean (SD)	32.4 (9.8)	35.6 (14.3)
	Range	16- 46	17- 64

Source: Tables 14.2.2.14, 14.2.2.15, 14.2.2.16, 14.2.2.17, 14.2.2.18, 14.2.2.19, 14.2.1.1.18

The median time to PR of 50% in the ALF-5755 group is 8 days, whilst the median time to PR of 50% for the placebo group was greater than the cut off time of 21 days. There was a significantly higher percentage of censored responses in the placebo group (85.7%) compared to the ALF-5755 group (38.5%) (p=0.0301).

Name of Sponsor/Company: Alfact Innovation	Individual Study Table Referring to Part of the Dossier	(For National Authority Use only)
Name of Finished Product: ALF-5755	Volume	
Name of Active Ingredient: recombinant human hepatocarcinoma-intestine-pancreas/pancreatitis-associated protein (rhHIP/PAP)	Page	

Time to PR of 50% (ITT Population with HBV or AIH)

		ALF-5755 (N=13)	Placebo (N=9)	p-value
Kaplan-Meier estimate (days)	Upper quartile	20.0		
	95% CI	(8.0, 28.0)	(20.0,)	
	Median	8.0		
	95% CI	(7.0, 20.0)	(20.0,)	
	Lower quartile	7.0	20.0	
	95% CI	(0.5, 8.0)	(20.0,)	
	Log Rank Test			0.0301
	Number (%) of responses	8 (61.5%)	1 (14.3%)	
	Number (%) censored	5 (38.5%)	6 (85.7%)	
	Missing	0	2	

Source: Table 14.2.4.4
Footnotes: 2 patients in Placebo group excluded from analysis because baseline PR >50%
Calculations are based on actual times of assessments (not scheduled times).
Baseline value was used instead of H0 for patient 1205 since the H0 result was missing.
Patients who are transplanted censored at the last assessment for PR before the transplant.
SD: Standard deviation

Although results of the primary efficacy analysis (i.e., the rate of change of PR during 72 hours following treatment initiation) did not show any effects on the ITT population, results within the pre-defined HBV/AIH subgroup showed some benefit.

- The primary endpoint (i.e., the rate of change of PR during 72 hours following treatment initiation) was achieved for the population receiving the complete treatment

**Name of Sponsor/Company:** Alfact Innovation	**Individual Study Table Referring to Part of the Dossier**	**(For National Authority Use only)**
**Name of Finished Product:** ALF-5755	**Volume**	
**Name of Active Ingredient:** recombinant human hepatocarcinoma-intestine-pancreas/pancreatitis-associated protein (rhHIP/PAP)	**Page**	

(p=0.0449);

- Early efficacy (0-12 hours) for the analysis of PR in the ITT Population with HBV or AIH (p=0.0385). In the ITT Population with HBV or AIH and who received 6 infusions, early efficacy (0-12 hours) for the analysis of PR were also observed (p=0.0295);
- Long term efficacy (0-Day 8) for the analysis of PR were observed in patients with HBV or AIH and who received the complete treatment (p=0.0067);
- Amongst the transplant free patients, the number of days in the hospital was significantly lower in the ALF-5755 group (compared to the placebo group) (p=0.023).

Pharmacokinetics (PK):

The plasma concentrations of ALF-5755 following infusions at H1 and H60 were all higher at H84 compared to baseline, as expected. The concentrations of ALF 5755 following transfusion, show the classic pattern of absorption, distribution and elimination.

Plasma concentrations of ALF-5755 following infusions at H1 and H60 were higher at H84 compared to baseline, and all PK parameters (t_{max}, $t_{1/2}$, AUC_{0-t}, and AUC_{0-12}) were higher by the sixth infusion compared to the first; C_{max} remained stable throughout. This increase has no specific meaning or consequences in terms of PK. Steady state was achieved by H48.

Safety:

The number of TEAEs was balanced between the two treatment groups. Severe TEAEs were higher in the ALF-5755 group, however serious TEAEs were higher in the placebo group. The majority of TEAEs were mild or moderate in severity. Two (2) patients died during the study, one in the ALF-5755 group which was deemed related to the study drug, and one in the

**Name of Sponsor/Company:**<br>Alfact Innovation	**Individual Study Table Referring to Part of the Dossier**<br><br>**Volume**<br>**Page**	**(For National Authority Use only)**
**Name of Finished Product:**<br>ALF-5755		
**Name of Active Ingredient:**<br>recombinant human hepatocarcinoma-intestine-pancreas/pancreatitis-associated protein (rhHIP/PAP)		

placebo group which was deemed not related to the study drug.

The number of SAEs and the number of patients who experienced SAEs were higher in the placebo group, indicating that ALF-5755 had an equal if not a slightly better safety profile compared to placebo.

No difference between the two treatments was seen in any laboratory parameter, neither in average values at a visit nor with respect to average changes between the end and the start of the treatment period.

Anti-ALF5755 antibodies were analysed at Days 0, 8 and 21, but no antibodies were detected.

Date of the Report:

01 November 2013

3 TABLE OF CONTENTS

1 TITLE PAGE 1
2 SYNOPSIS 4
3 TABLE OF CONTENTS 26
4 LIST OF ABBREVIATIONS AND DEFINITION OF TERMS 33
5 ETHICS 35
5.1 Independent Ethics Committee (IEC) 35
5.2 Ethical Conduct of the Study 35
5.3 Patient Information and Consent 35
6 INVESTIGATORS AND STUDY ADMINISTRATIVE STRUCTURE 36
7 INTRODUCTION 38
8 STUDY OBJECTIVES 40
8.1 Primary Objective 40
8.2 Secondary Objectives 40
9 INVESTIGATIONAL PLAN 41
9.1 Overall Study Design and Plan - Description 41
9.2 Discussion of Study Design, Including the Choice of Control Groups 42
9.3 Selection of Study Population 42
9.3.1 Inclusion Criteria 42
9.3.2 Exclusion Criteria 43
9.3.3 Removal of Patients from Therapy or Assessment 44
9.3.4 Replacement Policy 45
9.4 Treatments 45
9.4.1 Treatments Administered 45
9.4.2 Identity of Investigational Product(s) 46
9.4.3 Method of Assigning Patients to Treatment Groups 47
9.4.4 Selection of Doses in the Study 47
9.4.5 Selection and Timing of Dose for Each Patient 48
9.4.6 Blinding 48
9.4.7 Prior and Concomitant Therapy 49
9.4.8 Treatment Compliance 50
9.5 Efficacy and Safety Variables 50
9.5.1 Efficacy and Safety Measurements Assessed and Flow Chart 50

9.5.2 Safety Assessments 53
9.5.3 Primary Efficacy Variable....55
9.5.4 Drug Concentration Measurements 55
9.6 Data Quality Assurance....57
9.6.1 Quality Control 57
9.6.2 Quality Assurance 57
9.6.3 Audit....57
9.7 Statistical Methods Planned in the Protocol and Determination of Sample Size....57
9.7.1 Statistical and Analytical Plans....57
9.7.2 Determination of Sample Size 65
9.8 Changes in the Conduct of the Study or Planned Analyses....65
9.8.1 Protocol Amendments....65
9.8.2 Other Changes in Study Conduct....67
9.8.3 Changes in Planned Analysis....68
10 STUDY PATIENTS 70
10.1 Disposition of Patients 70
10.2 Protocol Deviations....71
11 EFFICACY EVALUATION 73
11.1 Data Sets Analysed 73
11.2 Demographic and Other Baseline Characteristics....73
11.3 Measurements of Treatment Compliance 78
11.4 Efficacy Results and Tabulations of Individual Patient Data 79
11.4.1 Analysis of Efficacy....79
11.4.2 Statistical/Analytical Issues 110
11.4.3 Tabulation of Individual Response Data....111
11.4.4 Drug Dose, Drug Concentration, and Relationships to Response 112
11.4.5 Efficacy Conclusions 126
12 SAFETY EVALUATION....129
12.1 Extent of Exposure....129
12.2 Adverse Events....129
12.2.1 Brief Summary of Adverse Events 129
12.2.2 Display of Adverse Events....129
12.2.3 Analysis of Adverse Events 130
12.2.4 Listing of Adverse Events by Patient....131
12.3 Deaths, Other Serious Adverse Events, and Other Significant Adverse Events...132

12.3.1 Listing of Deaths, Other Serious Adverse Events, and Other Significant Adverse Events....132
12.3.2 Narratives of Deaths, Other Serious Adverse Events, and Certain Other Significant Adverse Events....133
12.3.3 Analysis and Discussion of deaths, Other Serious Adverse Events and Other Significant Adverse Events....135
12.4 Clinical Laboratory Evaluation....135
12.4.1 Listing of Individual Laboratory Measurements by Patient and Each Abnormal Laboratory Value....135
12.4.2 Evaluation of each Laboratory Parameter....135
12.5 Vital Signs, Physical Findings and Other Observations Related to Safety....136
12.5.1 Vital Signs....136
12.5.2 Other Safety Evaluations....136
12.5.3 Special Safety Topics....136
12.6 Safety Conclusions....136
13 DISCUSSION AND OVERALL CONCLUSIONS....138
14 TABLES, FIGURES, AND GRAPHS REFERRED TO BUT NOT INCLUDED IN THE TEXT....140
14.1 Demographic Data....140
14.2 Efficacy Data....140
14.2.1 Subgroup Analyses....141
14.3 Safety Data....144
14.3.1 Display of Adverse Events....144
14.3.2 Safety Data....144
14.3.3 Listings of Deaths, Other Serious and Significant Adverse Events....144
14.3.4 Narratives of Deaths, Other Serious and Certain Other Significant Adverse Events....145
14.3.5 Laboratory Values....145
14.3.6 Abnormal Laboratory Value Listing (each patient)....145
14.3.7 Clinical Examination, Vital Signs and ECG....145
14.4 PK Data....145
15 REFERENCE LIST....146
16 APPENDICES....147
16.1 Study Information....147
16.1.1 Protocol and Protocol Amendments....147
16.1.2 Sample Case Report Form....147

16.1.3 List of ECs or IRBs and Representative Written Information for Patient and Sample Consent Forms 147
16.1.4 List and Description of Investigators and Other Important Participants in the Study, Including *Curricula Vitae* or Equivalent Summaries of Training and Experience Relevant to the Performance of the Clinical Study....147
16.1.5 Signatures of Principal or Coordinating Investigator(s) or Alfact Responsible Medical Officer....147
16.1.6 Listing of Patients Receiving Test Drug(s)/Investigational Product(s) from Specific Batches....147
16.1.7 Randomisation Scheme and Codes....147
16.1.8 Audit Certificates....147
16.1.9 Documentation of Statistical Methods....147
16.1.10 Documentation of Inter-Laboratory Standardisation Methods and Quality Assurance Procedures....147
16.1.11 Publications Based on the Study....147
16.1.12 Important Publications Referenced in the Report....147
16.2 Patient Data Listings....147
16.3 Case Report Forms....147
16.3.1 Case Report Forms for Deaths, Other Serious Adverse Events and Withdrawals for Adverse Events....147
16.3.2 Other Case Report Forms Submitted....147
16.4 Individual Patient Data Listings....147

LIST OF IN-TEXT TABLES

Table 6-1: Study Administrative Structure....36
Table 9-1: Dosage Schedule....41
Table 9-2: Composition of Formulation....46
Table 9-3: Flowchart of Schedule of Evaluations....51
Table 9-4: Schedule of PK....56
Table 10-1: Patient Disposition (All Available Patients)....70
Table 10-2: Study Termination and Primary Reason for Withdrawal (All Randomised Patients)....71
Table 11-1: Demographics and Baseline Characteristics (ITT Population)....74
Table 11-2: Baseline Factors (ITT Population)....75
Table 11-3: Baseline biological markers for liver function: INR, MELD, ALT, AST, Total Bilirubin, PR (ITT Population)....76
Table 11-4: Baseline biological markers for liver function: INR, MELD, ALT, AST, Total Bilirubin, PR (ITT Population with HBV or AIH)....77
Table 11-5: Analysis of Prothrombin Ratio (PR) (0-72 hours) (ITT Population)....79
Table 11-6: Analysis of International Normalized Ratio (INR) (0-72 hours) (ITT Population)....81
Table 11-7: Analysis of Model for End-stage Liver Disease (MELD) (0-72 hours) (ITT Population)....82
Table 11-8: Total CK18 (ITT Population)....84
Table 11-9: Cleaved CK18 (ITT Population)....84
Table 11-10: Transplant-Free Survival (ITT Population)....88
Table 11-11: Time to Transplantation (ITT Population)....90
Table 11-12: Hospital-Free Days (ITT Population)....91
Table 11-13: Days in Hospital (ITT Population, Alive with No Liver Transplant)....91
Table 11-14: Analysis of PR (0-12 hours) (ITT Population)....92
Table 11-15: Analysis of PR (0-Day 8) (ITT Population)....93
Table 11-16: Analysis of PR (0-72 hours) (ITT Population with HBV or AIH)....98
Table 11-17: Analysis of PR (0-72 hours) (ITT Population with HBV or AIH and who received 6 infusions of treatment)....98
Table 11-18: Hospital-Free Days (ITT Population with HBV and AIH)....104
Table 11-19: Days in Hospital (ITT Population with HBV or AIH, Alive with No Liver Transplant)....104
Table 11-20: Analysis of PR (0-12 hours) (ITT Population with HBV or AIH)....105
Table 11-21: Analysis of PR (0-12 hours) (ITT Population with HBV or AIH and who received 6 infusions of treatment)....105
Table 11-22: Analysis of PR (0-Day 8) (ITT Population with HBV or AIH)....106
Table 11-23: Analysis of PR (0-Day 8) (ITT Population with HBV or AIH and who received 6 infusions of treatment)....106
Table 11-24: Time to PR of 50% (ITT Population with HBV or AIH)....109

Table 11-25: Plasma Concentration of ALF-5755 (PK Population) 113
Table 11-26: PK Parameters for ALF-5755 (PK Population) 116
Table 12-1: Treatment-Emergent Adverse Events (Safety Population) 130
Table 12-2: Treatment-Emergent Adverse Events by Preferred Term (Safety Population) 131

LIST OF IN-TEXT FIGURES

Figure 9-1: Study Design....42
Figure 9-2: Amino Acid Sequence of ALF-5755....47
Figure 11-1: Plot of Baseline Total Bilirubin (ITT Population with HBV or AIH)....78
Figure 11-2: Kaplan-Meier Plot of Survival (ITT Population)....85
Figure 11-3: Plot of Baseline GCGlobulin (ITT Population)....86
Figure 11-4: Plot of Baseline Total Bilirubin (ITT Population)....87
Figure 11-5: Kaplan-Meier Plot of Transplant-Free Survival (ITT Population)....89
Figure 11-6: PR: Mean Plot to Day 21 (ITT population)....96
Figure 11-7: INR: Mean Plot to Day 21 (ITT population)....97
Figure 11-8: Kaplan-Meier Plot of Survival (ITT Population with HBV and AIH)....100
Figure 11-9: Plot of Baseline GCGlobulin (ITT Population with HBV or AIH)....101
Figure 11-10: Plot of Baseline Total Bilirubin (ITT Population with HBV or AIH)....102
Figure 11-11: Kaplan-Meier Plot of Transplant-Free Survival (ITT Population with HBV or AIH)....103
Figure 11-12: PR: Mean Plot to Day 21 (ITT population with HBV or AIH)....107
Figure 11-13: `INR: Mean Plot to Day 21 (ITT population with HBV or AIH)....108
Figure 11-14: Kaplan-Meier Plot of Time to Prothrombin Ratio (PR) of 50% (ITT Population with HBV and AIH)....110
Figure 11-15: Individual Patient PK Plots of ALF-5755 (PK Population): Patient 0101....118
Figure 11-16: Individual Patient PK Plots of ALF-5755 (PK Population): Patient 0102....119
Figure 11-17: Individual Patient PK Plots of ALF-5755 (PK Population): Patient 0108....120
Figure 11-18: Individual Patient PK Plots of ALF-5755 (PK Population): Patient 0112....121
Figure 11-19: Individual Patient PK Plots of ALF-5755 (PK Population): Patient 0113....122
Figure 11-20: Individual Patient PK Plots of ALF-5755 (PK Population): Patient 0114....123
Figure 11-21: Individual Patient PK Plots of ALF-5755 (PK Population): Patient 0201....124
Figure 11-22: Individual Patient PK Plots of ALF-5755 (PK Population): Patient 0204....125

4 LIST OF ABBREVIATIONS AND DEFINITION OF TERMS

%CV	Coefficient of Variation
AE	Adverse Event
ALF	Acute Liver Failure
ALT	Alanine Transaminases
AST	Aspartate Transaminases
AM	Arithmetic Mean
CI	Confidence Interval
CRF	Case Report Form
CTC	Common Toxicity Criteria
DBP	Diastolic Blood Pressure
FV	Factor V
GCP	Good Clinical Practice
GCS	Glasgow Coma Scores
GM	Geometric Mean
HE	Hepatic Encephalopathy
HIP/PAP	Hepatocarcinoma-Intestine-Pancreas/Pancreatitis-Associated Protein
HR	Heart Rate
IB	Investigator's Brochure
IEC	Independent Ethics Committee
IP	Investigational Product
INR	International Normalized Ratio
ITT	Intention-To-Treat
LNR	Limit of Hospital Laboratory Normal Range
MELD	Model for End Stage Liver Disease
NAC	N-acetyl cysteine
PICD	Patient Informed Consent Document
PK	Pharmacokinetic

PP	Per-Protocol
PR	Prothrombin Ratio
PT	Prothrombin Time
RR	Respiratory Rate
SAE	Serious Adverse Event
SAH	Severe Acute Hepatitis
SAP	Statistical Analysis Plan
SBP	Systolic Blood Pressure
SD	Standard Deviation
SOC	System Organ Class
TEAE	Treatment-Emergent Adverse Events

5 ETHICS

5.1 Independent Ethics Committee (IEC)

The clinical study protocol, its three amendments, and patient informed consent documents (PICDs), the current version of the Investigator's Brochure (IB), Investigators' current *curriculum vitae*, details of any compensation to patients, patient recruitment procedures e.g. adverts, and any other requested documents, were approved by each centre's Independent Ethics Committee (IEC). Full details of these committees can be found in Appendix 16.1.3.1.

5.2 Ethical Conduct of the Study

The study was conducted according to the clinical study protocol, the current International Conference on Harmonization (ICH) Good Clinical Practice (GCP) guidelines, any local guidelines, and the Declaration of Helsinki 2008.

5.3 Patient Information and Consent

Patient informed consent documents were based on a master document provided and approved by ORION Clinical Services and the study Sponsor prior to submission to the IEC. Any changes requested by the IEC were approved by ORION Clinical Services prior to the documents being used. A copy of the final IEC-approved consent form was submitted by ORION Clinical Services to the Sponsor prior to initiation of this study. The Investigator filed the signed PICDs for possible review by clinical research associates from ORION Clinical Services.

The Investigator or their assigned delegate was to fully explain the study to the patient using the PICD. If the patient’s cognitive function was impaired in a way that prevent obtaining of informed consent, his/her next of kin or an authorized person according to local procedures was asked to provide consent. After having been given sufficient time for consideration, if the patient (next of kin as appropriate) was willing to participate in the study, they were required to give written informed consent by signing the PICD. The Investigator (or medically qualified designee) was also to sign the PICD, prior to patient screening.

A representative PICD is provided in Appendix 16.1.3.2.

6 INVESTIGATORS AND STUDY ADMINISTRATIVE STRUCTURE

Table 6-1: Study Administrative Structure

Principal Investigator<br>Didier Samuel, MD PhD<br>Centre Hépatobiliaire<br>Hôpital Paul Brousse<br>12 Avenue Paul-Vaillant-Couturier<br>94800, Villejuif<br>France	Clinical Research Organization (CRO)<br>ORION Clinical Services<br>7 Bath Road, Slough, Berkshire,<br>SL1 3UA<br>United Kingdom
Clinical Trial Supply Management<br>Eurofins Pharma Quality Control<br>23,avenue de la Baltique<br>Villebon sur Yvette<br>91953 Courtaboeuf Cedex<br><br>Note: Eurofins was also in charge of supply lab kits for centralized samples.	Data Safety Monitoring Board (DSMB):<br>Stanislas Pol<br>DSMB Chairman<br>Hôpital Cochin<br>Service Hépatologie médicale<br>27, rue du Faubourg Saint-Jacques 75679 PARIS 14ème, France<br>Tel : +33.1.58.41.30.01<br>Fax : +33.1.58.41.30.15<br>e-mail : stanislas.pol@cch.aphp.fr
Clinical Laboratories<br>PK/ADA<br>ATLANBIO S.A.S<br>Route de St André des eaux<br>Z.I. de Brais – BP 40309<br>44605 Saint-Nazaire	Clinical Laboratories<br>CK18, IL-6 and GC-Globulin<br>INSERM (Institut National de la Santé et de la Recherche Médicale) -Unité INSERM U785<br>Hôpital Paul Brousse, Porte 64<br>CHB Aile Sud 2ème étage<br>14 avenue Paul Vaillant Couturier<br>94807 Villejuif Cedex
Biostatistics<br>Dr Bertrand Nalpas<br>Director of Research<br>Paris Biopark<br>7 rue Watt<br>75013, Paris<br>France	Study Report Preparation<br>Matthew Davis<br>ORION Clinical Services Ltd.<br>Head of Regulatory and Medical Writing<br>7 Bath Road, Slough<br>Berkshire<br>SL1 3UA<br>United Kingdom

DSMB Members & DSMB support group contact details are included in the DSMB Charter in Appendix 16.1.3. A list of Investigators and other important participants in the study, their affiliations and copies of their *curricula vitae*, are provided in Appendix 16.1.4.

7 INTRODUCTION

Acute liver failure (ALF) is a rare and life-threatening disease with high rate mortality in the absence of spontaneous recovery or liver transplantation. ALF affects young adults, is diagnosed in those with a median age of 38 years, and is more prevalent in women than men (Cox, 2009). Its outcome is related to the aetiology, the degree of encephalopathy, and related complications. Unfortunately, despite aggressive treatment, many patients die from fulminant hepatic failure (Hoofnagle et al., 1995; Lee et al,. 1999).

ALF is marked by the sudden loss of hepatic function in a person without pre-existing liver disease (Lee et al., 2008), and leads to a rapid deterioration of liver functions with the presence of coagulopathy and hepatic encephalopathy. All patients with clinical or laboratory evidence of moderate to severe acute hepatitis (SAH) should have immediate measurement of prothrombin time (PT) and careful evaluation for subtle alterations in mentation (Polson and Lee, 1995).

PT evolution over the first 72 hours has been recently demonstrated to accurately predict the outcome of ALF (Nalpas et al, Gut, 2011). Also, the prognosis of ALF markedly depends on the etiological factor; thus, those patients with acetaminophen-related ALF will show a much better prognosis, as compared with other etiologies. In this context, investigating a very large number of patients with acetaminophen-related ALF would be required to demonstrate efficacy of a given therapy. Finally, it has been now well established that SAH and ALF are not distinct entities. Instead, there is a continuous progression from SAH to early ALF and subsequently late stage ALF; whatever the therapy, the prognosis of ALF will be significantly worsened in patients with late stage ALF and selection of patients with SAH and early ALF should be favoured in novel therapeutic trials.

The survival of patients suffering from ALF depends on the speed with which quiescent liver cells re-enter the cell cycle and proliferate to compensate for the functional loss of liver cells (Kondo et al., 1997). It is also known that the more the liver cells are preserved during the toxic phase the more likely it is that recovery is possible (Michalopoulos and DeFrances, 1997). Therefore, two approaches not mutually exclusive in terms of new drug-based therapies are followed: to promote liver cell survival during the toxic phase; and to increase/favour liver regeneration. Moreover the importance of reactive oxygen species in the liver cell necrosis and apoptosis, as well as the importance of the extra-cellular matrix in controlling liver cell survival and proliferation have been well established.

ALF-5755, recombinant human hepatocarcinoma-intestine-pancreas/pancreatitis-associated protein (rhHIP/PAP), has been developed to become a future therapeutic specialty for the treatment of patients suffering from SAH and ALF. ALF-5755 exhibits similarly to HIP/PAP (Lieu et al., 2005; 2006), promoting both cell survival after apoptotic or oxidative stress and regeneration of hepatocytes in primary cultures and in vivo. ALF-5755 also significantly increased survival in a Fas-mediated ALF model in mice. In addition, HIP/PAP has been

reported to have anti-inflammatory (Iovanna and Dagorn, 2005), and bactericidal properties, which have also been shown with ALF-5755.

Importantly, it has been now established that ALF-5755 effects on liver cells, particularly its anti-oxydant properties, are largely dependent on its capacity to bind to and accumulate on the extra-cellular matrix. Thus, ALF 5755 shows a novel mechanism of therapeutical effect. In addition, it might be of interest in the future when treating patients with acute on chronic liver diseases.

In this study all patients received the best available care for nonacetaminophen SAH and early-stage ALF. ALF-5755 or placebo was administered in a randomized, blinded manner, in addition to standard care. Since ALF-5755 was not expected to affect the underlying condition, use of best treatment was ethically mandated and randomisation balanced any impact of specific treatments.

8 STUDY OBJECTIVES

8.1 Primary Objective

- To evaluate the efficacy of ALF-5755 versus placebo, measured by rate of change of prothrombin ratio (PR) during the 72 hours following treatment initiation, in patients with nonacetaminophen SAH and early stage ALF.

8.2 Secondary Objectives

- To evaluate the safety and tolerability of ALF-5755 versus placebo in patients with nonacetaminophen SAH and early stage ALF;
- To determine the pharmacokinetic (PK) parameters of ALF-5755 versus placebo in patients with nonacetaminophen SAH and early stage ALF.

9 INVESTIGATIONAL PLAN

9.1 Overall Study Design and Plan - Description

This was a prospective, multicentre, double-blind, placebo-controlled study of ALF-5755 in patients with nonacetaminophen SAH and early stage ALF, randomized in the ratio 1:1. A minimum of 60 patients were to be recruited into the study in the following two treatment groups:

- Group A: approximately 30 patients receiving ALF-5755;
- Group B: approximately 30 patients receiving placebo (physiological saline solution: 0.9% NaCl).

Patients received the investigational product (IP) (either placebo or ALF-5755) every 12 hours over 3 days according to the dosage schedule in Table 9-1~~Table 9-1~~. The time between biological baseline assessment and first injection of ALF-5755 or placebo was to be as short as possible, but not to exceed 48 hours.

Patients were to continue to receive (in addition to ALF-5755 or placebo) the best standard treatment upon assessment by the Investigators.

Table 9-1: Dosage Schedule

Group A: ALF-5755	Group B: Placebo
10 mg<br>(25 mL)<br><br>given in slow intravenous infusion over 10 minutes with an automatic syringe at:<br><br>H0<br>H12<br>H24<br>H36<br>H48<br>H60	25 mL of physiological saline solution<br>(0.9% NaCl)<br><br>given in slow intravenous infusion over 10 minutes with an automatic syringe at:<br><br>H0<br>H12<br>H24<br>H36<br>H48<br>H60

The study design is summarised in Figure 9-1~~Figure 9-1~~.

Figure 9-1: Study Design

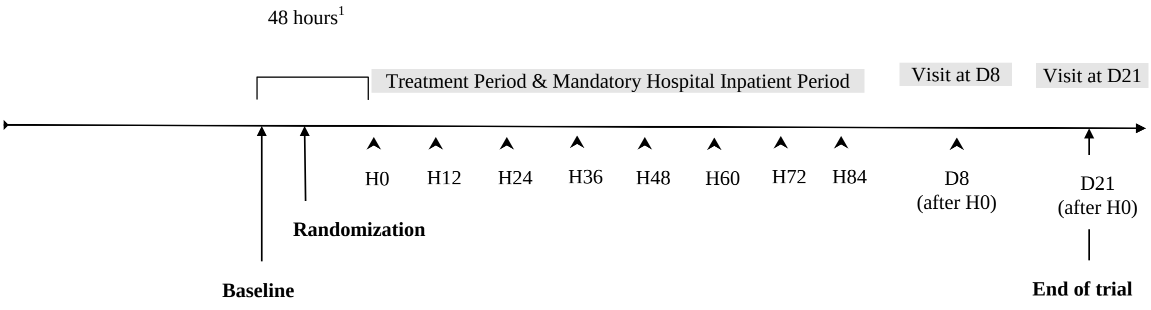

[1]First infusion within 48 hours after biological baseline assessment

The trial included the following visits:

- Baseline assessment prior to randomisation (the time between baseline assessment of biological data and H0 was to be as short as possible and not exceed 48 hours);
- Treatment period under permanent medical and nursing supervision from H0 (first injection) to H60 (last injection) (one infusion every 12 hours, e.g. H0, H12, H24, H36, H48, H60);
- A mandatory hospital inpatient follow-up period of 24 hours after the last infusion at H60, up to H84;
- A visit on Day 8 on an inpatient or outpatient basis;
- The end of study visit was on Day 21 on an inpatient or outpatient basis.

9.2 Discussion of Study Design, Including the Choice of Control Groups

A double-blind, randomized, placebo-controlled study design was chosen for this study to eliminate bias and to promote objectivity (Sections 9.4.3~~9.4.3~~ and 9.4.6~~9.4.6~~).

9.3 Selection of Study Population

9.3.1 Inclusion Criteria

A minimum of 60 patients (approximately 30 patients per group) were to be included in the study. Each patient had to:

- Meet all of the inclusion and exclusion criteria specified below (Section 9.4.6~~9.4.6~~) within the specified time-frame;

- Receive the allotted course of treatment (Sections 9.3.49.3.4 and 9.4.19.4.1) and complete the required activities specified in this protocol;
- Have their case report form (CRF) completed, received and accepted by ORION Clinical Services.

To be eligible for inclusion into this study, each patient had to fulfil all inclusion criteria prior to randomisation:

1. A signed written informed consent from patient or from patient's next of kin or from an authorized person according to local procedures;
2. Early stage ALF or SAH defined as:
   - 15% ≤ PR < 50%;
   - No hepatic encephalopathy, or grade I or II encephalopathy;
   - Presumed acute illness onset of less than 26 weeks;
   - No evidence of underlying cirrhosis.
3. Patients who could receive first treatment dose within the first 48 hours after biological baseline assessment;
4. Age ≥ 18 and ≤ 75 years;
5. Contraception (only for females of childbearing potential) to be taken throughout the study until Day 21. Sole mechanic contraceptives, such as condoms, were advised. Note: Oral contraceptives could have had contraindications in case of SAH and ALF;
6. Patients affiliated with a social security insurance system.

9.3.2 Exclusion Criteria

To be eligible for inclusion into this study, each patient was to not violate any of the following exclusion criteria prior to randomisation:

1. Acetaminophen-induced hepatitis defined as acetaminophen intake > 4 g/day, at least once in the 7 days prior to baseline;
2. Shock liver (ischemic hepatopathy) or HELLP syndrome or Budd-Chiari syndrome or intrahepatic malignancy;
3. Serum creatinine ≥ 180 µmol/L;
4. Body Mass Index (BMI) ≥ 35;
5. Septic shock requiring administration of inotropic drugs;
6. Uncontrolled active bleeding;
7. Patients who received fresh frozen plasma, prothrombine-proconvertine-stuart-B (PPSB), or vitamin K infusion over the last 24 hours;

8. Patients receiving liver support device treatment, including but not exclusively bioartificial liver (BAL), extracorporeal liver assist device (ELAD), transgenic pig perfusion;
9. Patient receiving haemodialysis, hemofiltration or haemodiafiltration treatment;
10. Intractable arterial hypotension (arterial systolic blood pressure [SBP] equal to or below 70 mmHg) present or require inotropic drugs at baseline;
11. Human immunodeficiency virus (HIV) positive patient;
12. Active cancer;
13. Pregnancy or breast-feeding;
14. Surgery within 4 weeks prior to baseline, or unsolved surgical disease outside liver transplantation;
15. Patient included in another clinical trial within 4 weeks prior to baseline;
16. Patient with organ or bone-marrow allograft.

9.3.3 Removal of Patients from Therapy or Assessment

Patients were informed that they had the right to withdraw from the study at any time, without prejudice to their medical care, and were not obliged to state their reasons. Any withdrawals were to be fully documented in the CRF and followed up by the Investigator.

However, the patient was informed that for safety reasons, anti-ALF-5755 antibody plasma levels had to be performed at Day 8 and Day 21 (Appendix 16.1.1).

Additionally, the Investigator could withdraw a patient at any time if they considered this to be in the patient's best interest.

The patient was to be discontinued from the study for the following reasons:

- Non-compliance with treatment schedule (e.g., discontinuation of study treatment in the case of toxicity);
- Death;
- At the specific request of the sponsor.

Additionally, patients could be discontinued for any of the following reasons:

- SAEs;
- Serious intercurrent illness or significant worsening of intercurrent illness, At the Investigator's discretion including not exclusively:
  - Serum creatinine ≥ 180 µmol/L;
  - Septic shock requiring administration of inotropic drugs;
  - Uncontrolled active bleeding;

- Intractable arterial hypotension (arterial SBP ≤ 70 mmHg) present or requiring inotropic drugs.

- If the patient was lost-to-follow-up;
- At their own request or at the request of their legally accepted representative;
- Protocol violations, including non-compliance with study procedures;
- If in the Investigator's opinion continuation in the study would be detrimental to the well-being of the patient.

If a patient failed to return for a scheduled visit/follow up, attempts were to be made to contact the patient to ensure that the reason for not returning was not an AE. Likewise if a patient declared their wish to discontinue from the study e.g. for personal reasons, an attempt was to be made to establish that the true reason was not an AE (bearing in mind the patient was not obliged to state their reasons).

If the IP therapy was prematurely discontinued, the primary reason for discontinuation was recorded in the appropriate section of the CRF and all efforts were made to complete and report the observations as thoroughly as possible, including extra sampling measurements. A complete final evaluation following the patient's withdrawal was to be made (Appendix 16.1.1), and any AEs followed up until resolution or a period of 18 days from the last dose of the IP had elapsed, whichever was the longer.

The study was to be terminated if, in the opinion of the Investigator and the Sponsor, significant safety concerns arose during the conduct of the study.

9.3.4 Replacement Policy

Recruitment continued until 60 patients were evaluable, i.e. had no major deviations impacting the evaluability of the primary endpoint and had PR assessments allowing rate of change of PR calculation (Appendix 16.1.1).

9.4 Treatments

9.4.1 Treatments Administered

ALF-5755 was supplied lyophilised in a glass vial with halobutyl stopper and aluminium seal. The vial was stoppered under nitrogen with partial vacuum. Each vial contained 2.80 mg of ALF-5755. Reconstitution in the vial with 6.6 mL of water for injection gave a clear colourless solution at 0.40 mg/mL of ALF-5755. Needles and syringes were supplied for use during the dilution procedure.

Immediately, after the end of the intravenous administration, the infusion lines were washed with 3 to 5 mL of physiological saline solution.

ALF-5755 was supplied in glass vials. A total of 4 vials were required for each 10-minute slow infusion. The composition of the formulation included in each vial is provided in Table **9-2**~~Table **9-2**~~ below.

Table 9-2: Composition of Formulation

Component	Quantity per vial
ALF-5755	2.80 mg
Tris HCl pH8	10 mM
NaCl	50 mM
Sucrose	200 mM
Water for injection *qsp*	7 mL

A total of 10 mg (25 mL) was administered every 12 hours over 3 days.

ALF-5755, and placebo were administered as slow intravenous infusions, each a 10-minute infusion of 10 mg (25 mL) using automatic syringes every 12 hours from H0 until H60 (H0, H12, H24, H36, H48 and H60, all ± 10 minutes).

9.4.2 Identity of Investigational Product(s)

9.4.2.1 Presentation of ALF-5755

<u>Nomenclature</u>

ALF-5755 is an *Escherichia coli* (*E. coli*)-borne recombinant protein with the amino acid sequence of the human protein HIP/PAP, (Lasserre et al., 1992; Christa et al., 1994; Lieu et al., 2005; Lieu et al., 2006).

ALF-5755 has not yet been attributed with a non-proprietary name. ALF-5755 is the code name of the made recombinant human HIP/PAP by Alfact Innovation.

<u>Structure</u>

ALF-5755 has the sequence of the human HIP/PAP protein. For expression in *E. coli*, the first 26 amino acids corresponding to the signal sequence were removed, and an additional methionine was added in position 1.

Figure 9-2: Amino Acid Sequence of ALF-5755.

```
Position 1    11   21   31   41
ALF-5755    MEEPQRELPS ARIRCPKGSK AYGSHCYALF LSPKSWTDAD LACQKRPSGN

Position 51   61  71   81   91
ALF-5755 LVSVLSGAEG SFVSSLVKSI GNSYSYVWIG LHDPTQGTEP NGEGWEWSSS

Position 101  111  121  131  141
ALF-5755    DVMNYFAWER NPSTISSPGH CASLSRSTAF LRWKDYNCNV RLPYVCKFTD
```

Cysteines with the same colour (#15 + #26, #43 + #143 and #121 + #146) are shown to form intrachain bonds.

Batch numbers are provided per patient in Appendix 16.1.6.

9.4.2.2 Storage/Stability of ALF-5755

Before reconstitution, the IP, ALF-5755, was stored at -20 ± 5 °C unless otherwise specified by Alfact Innovation. The product could be left for up to 24 hours at room temperature because it was lyophilised. Upon reconstitution and transfer into the provided syringe, the product could be stored for up to 20 hours (following start of reconstitution procedure) at +5± 3°C, if not infused within 2 hours after reconstitution. All IPs were stored in a secure location, and dispensed only by the Investigator or by a member of staff specifically authorised by the Investigator, or by a pharmacist, as appropriate. Any deviations from the recommended storage conditions were to be immediately reported to ORION Clinical Services and the use of drug interrupted until authorisation for its continued use had been given by Alfact Innovation.

9.4.3 Method of Assigning Patients to Treatment Groups

Patients were randomly assigned to either Group A (ALF-5755) or Group B (Placebo) in the randomisation ratio 1:1.

Randomisation was stratified by centre. The site pharmacist (or designee) was unblinded as the pharmacist (or designee) had to reconstitute ALF-5755 vials as well as the syringes for intravenous infusion containing 25 mL of placebo or ALF-5755. No other site personnel were unblinded. The site pharmacist (or designee) was provided with envelopes containing treatment allocation. An excess of envelopes were provided to make provision for extra recruitment following patient discontinuation.

9.4.4 Selection of Doses in the Study

The dose regimen of ALF-5755 was 10 mg administered every 12 hours over 3 days.

The dose of 20 mg daily was chosen as it was above the anticipated therapeutic dose of 4 mg and had a satisfactory tolerance when given in one infusion of 20 mg in healthy volunteers. Given in a split dose of 10 mg twice a day, it enables better coverage of the 24-hour period as ALF-5755 was shown to have a short half-life of 4 hours in the first in man study. Accumulation was not anticipated as there was no accumulation in two species of animal PK models after a daily infusion of ALF-5755 over 14 days, and the no observed adverse effect level (NOAEL) was above maximum tested dose in all species. ALF-5755 PK characteristics have shown to be similar amongst species including humans with low inter individual variability.

The route and mode of administration, a slow intravenous infusion over 10 minutes, were kept as in the first-in-man study.

9.4.5 Selection and Timing of Dose for Each Patient

Given in split dose of 10 mg twice a day, it enables better coverage of the 24-hour period as ALF-5755 was shown to have a short half-life of 4 hours in the first in man study.

The 3-day treatment period was chosen as that used for most experimental or registered therapies as N-acetyl cysteine (NAC) or medical device under clinical evaluation in ALF. It was also the time delay where physicians were assessing the prognosis of spontaneous recovery in patients with ALF, and deciding whether or not to list the patient on the registry for liver transplantation. This time period also covered the disease period where ALF-5755 efficacy was shown, as the efficacy of ALF-5755 had been demonstrated at 6 hours and above after induction of ALF in the Fas mice model.

Patients were dosed at H0 (first injection) to H60 (last injection), with one infusion every 12 hours (e.g. H0, H12, H24, H36, H48, H60).

9.4.6 Blinding

The pharmacist or designee was the only personnel to have access to the randomisation envelopes in order to prepare the drug for administration.

9.4.6.1 Emergency Unblinding

In case of an emergency, when knowledge of the IP assignment was required for the medical management of an individual subject, the treatment for that patient could be unblinded. A set of emergency unblinding envelopes were stored on site and at ORION Clinical Services. The Investigator notified the Sponsor within 24 hours after determining that it was necessary to unblind the treatment assignment.

This documentation included the name of the individual breaking the blind, the date on which the blind was broken, and a description of the event that led to the unblinding. The Investigator also indicated in source documents and in the CRF that the blind was broken and provide the date, time, and reason for breaking the blind. Any adverse event (AE) or serious adverse event (SAE) associated with breaking the blind was recorded and reported (Appendix 16.1.1).

The independent drug monitor routinely checked the integrity of the envelopes that were stored at the site. The envelopes were collected from the site prior to study close-out and sent to the Sponsor to ensure that they were all intact.

9.4.7 Prior and Concomitant Therapy

9.4.7.1 Concomitant Therapy/Medical Management of Adverse Events

Reasonable efforts were made to determine all relevant treatments received by the patient within 30 days before IP administration. All relevant information was recorded on the patient's CRF.

Any medications, with the exceptions noted in Section 9.4.7.2~~9.4.7.2~~, which were considered necessary for the patient's welfare, and which did not interfere with the study medication, could be given at the discretion of the Investigator. Medications/therapies which were administered as part of the study treatment schedule are listed in Section 9.4.7.3~~9.4.7.3~~.

Administration of all concomitant drugs was reported in the appropriate section of the CRF along with dosage information, dates of administration and reasons for use. Additionally any unplanned diagnostic, therapeutic or surgical procedure performed during the study period was recorded in the comments section of the corresponding AE report.

9.4.7.2 Medications That May Not Be Administered

The use of any herbal/natural products (including the use of vitamins, nutritional supplements) or other "folk remedies" was to be discouraged, unless deemed necessary by the Investigator (as part of symptom management or standard of care). The patients were instructed that no additional medication was allowed without the prior consent of the Investigator. Any medication considered necessary for the patient's safety and wellbeing could be given at the discretion of the Investigator. Use of these products and all other concomitant medications were recorded in the CRF.

Haemodialysis, haemofiltration or haemodiafiltration treatment were to be discouraged too, but could be deemed necessary (as part of symptom management or standard of care). In the

latter case, the Investigator was instructed whenever possible to perform dialysis sessions before IP infusion and/or at least 6 hours after last IP taking into account half-life of the IP and to avoid the IP filtration.

9.4.7.3 Mandatory Concomitant Therapy

Patients were to continue and receive (in addition to ALF-5755 or placebo) the best standard treatment on assessment of investigators.

9.4.8 Treatment Compliance

All doses were administered in the clinical unit under direct supervision of the Investigator or an attending physician. All drug administration information was recorded in the CRF and in the drug movement form.

9.5 Efficacy and Safety Variables

9.5.1 Efficacy and Safety Measurements Assessed and Flow Chart

Efficacy assessments are outlined in Table 9-3~~Table 9-3~~ including timing of each assessment. Further details are provided in the study protocol in Appendix 16.1.1. The site personnel responsible for carrying out critical measurements are listed in Appendix 16.1.4.

Table 9-3: Flowchart of Schedule of Evaluations

Study assessment & exams	Baseline	RANDOMIZATION	H0 ± 10 min	H12 ± 10 min	H24 ± 10 min	H36 ± 10 min	H48 ± 10 min	H60 ± 10 min	H72 ± 10 min	H84 ± 10 min	D8 (after H0) +2 days	D21 (after H0) +2 days	Premature Discontinuation
Informed consent signed	X												
Check Incl./excl. Criteria	X												
Medical history	X												
Demographics (age, gender)	X												
Weight, height & BMI	X												
Viral serologies and/or PCR (HAV, HBV, HCV, HDV, HEV, HSV,VZV, HIV)	X												
Acetaminophen, alcohol plasma levels	X												
Cupremia, ceruloplasmin plasma levels, and cupruria	X												
Autoimmune markers (ANA, ASMA, anti-LKM Ab, Ig levels)	X												
Beta hCG blood pregnancy test for women of childbearing potential only	X											X	X
ALF-5755/ Placebo infusions			Within 48 hrs after baseline	X	X	X	X	X					
Clinical examination	X		Before, 10 min after infusion	Before, 10 min after infusion	Before, 10 min after infusion	Before, 10 min after infusion	Before, 10 min after infusion	Before, 10 min after infusion	X	X	X	X	X
Vital signs (BP, HR, RR)	X		Before infusion, 10 min + HR and BP only during 1 hr after infusion	Before infusion, 10 min + HR and BP only during 1 hr after infusion	Before infusion, 10 min + HR and BP only during 1 hr after infusion	Before infusion, 10 min + HR and BP only during 1 hr after infusion	Before infusion, 10 min + HR and BP only during 1 hr after infusion	Before infusion, 10 min + HR and BP only during 1 hr after infusion	X	X	X	X	X
Body temperature	X		Before infusion	Before infusion	Before infusion	Before infusion	Before infusion	Before infusion	X	X	X	X	X
ECG	X		Before, 10 min and 1 hr after infusion		Before, 10 min and 1 hr after infusion		Before, 10 min and 1 hr after infusion		X	X	X	X	X
Hepatic Encephalopathy grade & GCS	X		Before infusion	Before infusion	Before infusion	Before infusion	Before infusion	Before infusion	X	X	X	X	X

Arterial blood gas with lactates if grade I or II hepatic encephalopathy or GCS < 15 or if required from investigator's assessment or in case of abnormality at screening	X				Before infusion		Before infusion		X		X	X	X
PR, FV, INR, ALT, AST	X		Before infusion	Before infusion	Before infusion	Before infusion	Before infusion	Before infusion	X	X	X	X	X
Haematology, Biochemistry	X		Before infusion	Before infusion	Before infusion	Before infusion	Before infusion	Before infusion	X	X	X	X	X
Urinalysis			Before infusion		Before infusion		Before infusion		X		X	X	X
Anti-ALF-5755 antibodies[1]			Before infusion								X	X	
Concomitant medications & AE recording (from signed PICD)	X		X	X	X	X	X	X	X	X	X	X	X
CK18 (at Paul Bousse and Beaujon hospital)[2]			Before infusion	Before infusion	Before infusion	Before infusion	Before infusion	Before infusion	X	X			X

1: For safety reasons, anti-ALF-5755 antibody plasma levels were performed at Day 8 and Day 21 as described (Appendix 16.1.1).

2: According to sites recruitment rate as well as patient withdrawal during treatment period that could occur, additional sites could be asked to also perform to have a minimum of patients for CK18 results interpretation.

9.5.2 Safety Assessments

Safety assessments involved the comparison of:

- Local and systemic AEs (AEs/SAEs) (AEs were to be recorded from the time point that the PICD had been signed).

Safety assessments involved the assessment of:

- Laboratory findings (including haematology, biochemistry and urinalysis);
- Clinical examination, clinically significant vital signs (blood pressure [BP], heart rate [HR], respiratory rate [RR]), body temperature;
- Hepatic encephalopathy (HE) grades and Glasgow Coma Scores (GCSs);
- Electrocardiogram abnormalities;
- Plasma levels of anti-ALF-5755 antibodies;
- Beta hCG blood pregnancy test in women of childbearing potential;
- Arterial blood gas with lactate at H24 before infusion, H48 before infusion, H72 in case of grade I or II HE or GCS < 15 or if required from Investigator's assessment or in case of abnormality at screening.

Full information about the procedures for reporting AEs and SAEs and the assessment of other safety parameters is given in the protocol (Appendix 16.1.1). The definitions of AEs and SAEs are given below.

Adverse Events

An AE is defined as any untoward medical occurrence in a patient or clinical investigation subject administered a pharmaceutical product, occurring at any dose and which does not necessarily have a causal relationship with the treatment. An AE can therefore be any unfavourable and unintended sign (including an abnormal laboratory finding), symptom, or disease temporally associated with the use of a medicinal IP, whether or not related to the medicinal IP.

An AE can refer to an untoward response to the administration of the IP, but can also occur as a result of the protocol-required procedures. Therefore, safety data was collected after written informed consent had been obtained and before administration of the IP.

Adverse events include the following:

- All suspected adverse medication reactions;
- All reactions from medication overdose, abuse, withdrawal, sensitivity, or toxicity;
- Apparently unrelated illnesses, including the worsening of a pre-existing illness;

- Injury or accidents. Note that if a medical condition was known to have caused the injury or accident, the medical condition and the accident were to be reported as two separate AEs;
- Abnormalities in physiological testing or physical examination findings that required clinical intervention or further investigation (beyond ordering a repeat [confirmatory] test);
- Laboratory abnormalities that required clinical intervention or further investigation (beyond ordering a repeat [confirmatory] test) unless they were associated with an already reported clinical event. Any new or aggravated clinically relevant abnormal medical finding at a physical examination as compared with the baseline assessment was reported as an AE. Where possible the Investigator was to use the clinical and not the laboratory term (e.g. hyperkalaemia versus high potassium).

Serious Adverse Events

A SAE is defined as any untoward medical occurrence that at any dose:

- Results in death:
    - Death is an outcome of an AE, and not an AE in itself. In reports of death due to "disease progression", where no other information was provided, the death was assumed to have resulted from progression of the disease being treated with the IP(s);
    - Is life-threatening (i.e. the patient was at risk of death at the time of the event. It did not refer to an event which hypothetically might have caused death if it were more severe).
- Requires inpatient hospitalisation or prolongation of existing hospitalisation;
    - Complications that occurred during hospitalisations were AEs. If a complication prolonged hospitalisation, it was a SAE;
    - "In-patient hospitalisation" meant the patient had been formally admitted to a hospital for medical reasons, for any length of time, overnight or not. It did not include presentation and care within an emergency department.
- Results in persistent or significant disability/incapacity;
- Is a congenital anomaly/birth defect;
- Is an important medical event(s) that might have not been immediately life-threatening or resulted in death or hospitalisation but that might have jeopardised the patient or required intervention to prevent one of the above outcomes.

Medical and scientific judgment was to be trained in deciding whether a case was serious.

"Occurring at any dose" did not imply that the patient was receiving IP at the time of the event. Dosing could have been given as treatment cycles or interrupted temporarily prior to the onset of the SAE, but could have contributed to the event.

9.5.3 Primary Efficacy Variable

The primary efficacy variable was the rate of change of PR during 72 hours following treatment initiation.

9.5.4 Drug Concentration Measurements

9.5.4.1 Pharmacokinetic (PK) assessments

PK assessments are outlined in Table 9-4~~Table 9-4~~ including timing of each assessment. Further details are provided in the study protocol in Section 9.7.1.11~~9.7.1.11~~ and Appendix 16.1.1. The site personnel responsible for carrying out critical measurements are listed in Appendix 16.1.4.

Table 9-4: Schedule of PK

	H0 10 min	H1	H2	H6	H12	H48	H60	H60 10 min	H61	H62	H66	H72	H84	Premature discontinuation
X just before 1st infusion	X end of 1st infusion	X	X	X	X just before 2nd infusion	X just before 5th infusion	X just before 6th infusion	X end of 6th infusion	X	X	X	X	X	X

9.6 Data Quality Assurance

9.6.1 Quality Control

Quality control was defined as the operational techniques and activities undertaken within the quality assurance system to verify that the requirements for quality of the trial-related activities had been fulfilled.

Quality control was to be applied to each stage of data handling to ensure that all data were reliable and had been processed correctly.

9.6.2 Quality Assurance

Quality assurance was defined as the planned and systematic actions that were established to ensure that the trial was performed and the data generated, documented (recorded) and reported in compliance with GCP and the applicable regulatory requirements.

9.6.3 Audit

The Investigator permitted an audit mandated by the sponsor after reasonable notice. The purpose of an audit was to confirm that the study was conducted as per protocol, GCP and applicable regulatory requirements, that the rights and well-being of patients enrolled had been protected and that all data relevant for the evaluation of the IP captured, processed and reported in compliance with the planned arrangements. The Investigator permitted direct access to all study documents, IP accountability records, medical records and source data. The Investigator and their study team were also available for discussion regarding study progress and procedures during the audit (both during the audit and at the end of the audit for an "exit" discussion).

9.7 Statistical Methods Planned in the Protocol and Determination of Sample Size

9.7.1 Statistical and Analytical Plans

The data were analysed by ORION Clinical Services. Statistical analyses were performed using SAS®, Version 9.2 or later, SAS Institute, Cary, Northern Carolina, USA. Detailed information on statistical methods is provided in the statistical analysis plan (SAP) version 3 dated 17 May 2013 (Appendix 16.1.1).

9.7.1.1 Analysis Data Sets

The statistical analysis was based on the following analysis data sets:

- **Safety Population**: all patients who were randomised to treatment and received at least one administration of ALF-5755 or placebo. Patients who received the wrong treatment were to be analysed as treated when using the Safety Population.
- **Intention-to-treat (ITT) Population**: all patients in the Safety Population who provided at least one post-dose efficacy assessment. Note that patients who withdrew early due to worsening disease before the first post-baseline efficacy assessment were to be included as their efficacy assessments were to be imputed. Patients who received the wrong treatment were to be analysed as randomised when using the ITT Population.
- **Per-protocol (PP) Population**: all patients in the ITT Population who completed the double blind treatment phase of the study with no major protocol violations. Protocol violations were to be defined in the SAP, and discussed and agreed in a blind review meeting prior to database lock.

9.7.1.2 Patient Demographics and Other Baseline Characteristics

Data is summarised by treatment group. A total column showing all patients is included for baseline and safety summaries. Where appropriate, data is also summarised by visit.

In summary and analysis tables of continuous variables, standard descriptive statistics (N, mean, standard deviation [SD], median, minimum and maximum), and 95% confidence interval (CI), are presented in the statistical analysis outputs as appropriate.

For PK summaries, arithmetic mean (AM), geometric mean (GM) and coefficient of variation (%CV) are used to summarise the data. The minimum and maximum statistics are presented in summary tables to the same number of significant figures as the original data. The mean/AM, median, GM, CI and SD are presented to one more significant figure than the original data.

9.7.1.3 Patients and Treatments

Clinical profiles of groups treated with ALF-5755 and placebo are summarised to identify any baseline imbalance between the treatment groups. Age, gender, aetiology, and severity of disease at inclusion are presented.

Limited programming checks e.g. prohibited medication (based on agreed codes), lab results, to determine deviations were proposed and reviewed on an ongoing basis. Definition of major/minor deviations and allocation to the study populations were agreed before database

lock and unblinding. It was intended that BMI ≥ 35 kg/m^2 was defined as a minor deviation, and that visits outside the window for all visits except H72 were defined as a minor deviations.

The primary analysis was repeated using the PP Population.

9.7.1.4 Statistical Model, Hypothesis, and Method of Analysis

The statistical methods for this study are described in the SAP (Appendix 16.1.1).

All hypothesis testing was performed at the 5% (two-sided) significance level unless stated otherwise.

P-values are rounded to four decimal places. P-values less than 0.0001 are reported as <0.0001 in tables.

The imputations/modifications to be used for missing data are defined in the SAP (Appendix 16.1.1).

Sites were pooled for all analyses. There was no adjustment for centre effect or treatment by centre interaction.

There were no corrections to nominal p-values for multiple comparisons.

Subgroups were defined based on initial PR, hepatitis aetiology and concomitant medication as follows:

- Initial PR above/below the median value for the ITT population;
- Hepatitis aetiology (viral hepatitis A/viral hepatitis B/viral hepatitis E/autoimmune hepatitis/other aetiology/undetermined aetiology/drug-induced aetiology);
- Hepatic encephalopathy grade (0/I/II/III/IV) (only classifications 0, I and II were to be represented at baseline);
- NAC treatment (yes/no).

Subgroups could be combined for post-hoc exploratory analyses.

9.7.1.5 Efficacy Analyses

Treatment comparisons were ALF-5755 versus placebo. The main analysis set for the efficacy analyses was the ITT Population.

The primary efficacy analysis was the treatment comparison of rate of change of PR from H0 to H72 using the Wilcoxon rank-sum test and the ITT Population. A p-value below 0.05 was considered significant. This analysis was repeated for the PP Population.

9.7.1.6 Evaluation of Primary Efficacy Endpoint

The primary endpoint of the study was PR, measured and recorded on the CRF at baseline, H0 before first infusion, H12 before second infusion, H24 before third infusion, H36 before fourth infusion, H48 before fifth infusion, H60 before sixth infusion, H72, H84, D8 (after H0), D21 (after H0) or premature discontinuation.

The primary variable for statistical comparison between treatment groups was the **rate of change of PR during 72 hours following treatment initiation** calculated as:

$$\frac{[\text{PR at H72} - \text{PR at H0 pre-dose}]}{72}$$

Assessments were included for the primary (ITT) analysis whether or not they were within the visit window.

If PR reached the limit of the hospital laboratory normal range of values (LNR) before H72, the formula was to be modified as follows:

$$\frac{[\text{First value} \geq \text{LNR} - \text{PR at H0 pre-dose}]}{\text{Nominal[1] time to first reach LNR or above}}$$

For PR the value of LNR is 80% which is the lower limit of normal i.e. the modification applies to the first value of PR ≥ 80%.

If the patient had no recorded PR at H72 but did have an earlier post-H0 assessment, the formula was to be modified as follows:

$$\frac{[\text{Last recorded PR} - \text{PR at H0 pre-dose}]}{\text{Nominal[2] time to last recorded PR}}$$

If the patient had no recorded PR at H0 but did have an earlier pre-H0 assessment (baseline), the relevant formula above was be modified to use PR at baseline instead of PR at H0 pre-dose, and to add 12 hours to the time in the denominator to reflect the time between baseline and H0[3].

---

[1] Planned assessment time, i.e. 12 hours, 24 hours,etc

[2] Planned assessment time, i.e. 12 hours, 24 hours,etc

[3] This applied to just one patient (1205), whose baseline assessment was reported by Alfact to have been done 12 hours pre-dose. The decision was made before unblinding. If the patient was on placebo, this gave a reliable estimate of rate of change of PR; if the patient was on ALF5755, it gave a conservative estimate.

If the patient withdrew early (before H72), an extra PR sampling at time of withdrawal was to be done if possible, and used as the last recorded value of PR in this formula. If the patient was transplanted before H72, assessments after the transplant were treated as missing for this formula.

It was expected that all patients would have at least one post-baseline assessment of PR. If any patients had no post-baseline assessment of PR, a decision was to be made on a case by case basis at the blind data review meeting before database lock whether or not to impute values for the analysis.

Rate of change of PR during the 72 hours following treatment initiation in ALF-5755 and placebo arms was compared by a non parametric test (Wilcoxon). In addition, the comparison was adjusted for sub-groups based on initial PR and aetiology using the Cochran-Mantel-Haenszel test.

Analyses were performed with the ITT Population and the PP Population. A p-value below 0.05 was considered as significant.

9.7.1.7 Evaluation of secondary efficacy endpoints

The secondary endpoints of the study were:

Model for end-stage liver disease (MELD) was calculated as follows:

$$\text{MELD} = 3.78 \ln(\text{bilirubin}) + 11.2 \ln(\text{INR}) + 9.57 \ln(\text{creatinine}) + 6.43$$

with the following modifications:

- Any value (bilirubin, INR or creatinine) less than 1 was to be scored as 1;
- If the patient had been dialysed twice within the previous 7 days, then the value of creatinine used was to be 4.0 mg/dL. (Evidence of no dialysis before enrolment was to be based on Exclusion Criterion 9. If dialysis was performed after enrolment, it was to be recorded on the concomitant procedures page of the CRF. This information was to be used to determine the appropriate value for creatinine to use in the formula).

- **Rate of change of Factor V (FV) plasma level** calculated as follows:

$$\frac{[\text{FV at 72 hours (i.e. 12 hours after the last infusion)} - \text{FV at H0 pre-dose}]}{\text{72 hours}}$$

If the FV reached the hospital laboratory normal range of values before 72 hours, the formula was modified as follows:

$$\frac{[\text{Hospital laboratory normal range of values of FV} - \text{FV at H0 pre-dose}]}{\text{Time (hours) to first reach hospital laboratory normal range of values of FV since H0}}$$

If the patient had no recorded FV at 72 hours but did have an earlier post-H0 assessment, the formula was modified as follows:

$$\frac{\text{[Last measured value of FV – FV at H0 pre-dose]}}{\text{Time (hours) elapsed when last FV value was measured since H0}}$$

If the patient withdrew early, an extra FV sampling at time of withdrawal was performed if possible, and used as the last measured value of FV in this formula:

- **Rate of change of international normalized ratio (INR)** calculated as follows:

$$\frac{\text{[INR at 72 hours (i.e. 12 hours after the last infusion) – INR at H0 pre-dose]}}{\text{72 hours}}$$

If the INR reached the hospital laboratory normal range of values before 72 hours, the formula was modified as follows:

$$\frac{\text{[Hospital laboratory normal range of values of INR – INR at H0 pre-dose]}}{\text{Time (hours) to first reach hospital laboratory normal range of value of INR since H0}}$$

If the patient had no recorded INR at 72 hours but did have an earlier post-H0 assessment, the formula was modified as follows:

$$\frac{\text{[Last measured value of INR – INR at H0 pre-dose]}}{\text{Time (hours) elapsed when last INR value was measured since H0}}$$

- **Rate of change of MELD during 72 hours** following treatment initiation; defined as for the primary including the modifications other than LNR.

If the patient withdrew early, an extra INR sampling at time of withdrawal was performed if possible, and used as the last measured value of INR in this formula:

- **Rate of change of alanine transaminases [ALT] plasma level** calculated as follows:

$$\frac{\text{[ALT at 72 hours (i.e. 12 hours after the last infusion) – ALT at H0]}}{\text{72 hours}}$$

If the ALT reached the hospital laboratory normal range of values before 72 hours, the formula was modified as follows:

$$\frac{\text{[Hospital laboratory normal range of values of ALT – ALT at H0 pre-dose]}}{\text{Time (hours) to first reach hospital laboratory normal range of values of ALT since H0}}$$

If the patient had no recorded ALT at 72 hours but did have an earlier post-H0 assessment, the formula was modified as follows:

[Last measured value of ALT – ALT at H0 pre-dose]

Time (hours) elapsed when last ALT value was measured since H0

If the patient withdrew early, an extra ALT sampling at time of withdrawal was performed if possible, and used as the last measured value of ALT in this formula:

- **Rate of change of aspartate transaminases (AST) plasma level** calculated as follows:

$$\frac{\text{[AST at 72 hours (i.e. 12 hours after the last infusion) – AST at H0]}}{\text{72 hours}}$$

If the AST reached the hospital laboratory normal range of values before 72 hours, the formula was modified as follows:

$$\frac{\text{[Hospital laboratory normal range of values of AST – AST at H0 pre-dose]}}{\text{Time (hours) to first reach hospital laboratory normal range of values of AST since H0}}$$

If the patient had no recorded AST at 72 hours but did have an earlier post-H0 assessment, the formula was modified as follows:

$$\frac{\text{[Last measured value of AST – AST at H0 pre-dose]}}{\text{Time (hours) elapsed when last PR value was measured since H0.}}$$

If the patient withdrew early, an extra AST sampling at time of withdrawal was performed if possible, and used as the last measured value of AST in this formula.

- Change of HE grade during 72 hours following treatment initiation;
- Overall survival rate at Day 21;
- Transplant-free survival rate at Day 21;
- Liver transplant rate at Day 21;
- Length of hospitalisation (days).

Change in HE grade between the treatment groups was compared using the Cochran-Mantel-Haenszel test.

Overall survival rate, transplant-free survival rate and transplantation rate were analysed by the Chi-squared test and the log-rank test. Kaplan-Meier estimates were presented. Analyses were also adjusted for aetiology.

Rate of change of FV, INR, ALT and AST were calculated using the same formula as for PR and analysed in the same way. Analyses used the ITT Population. A p-value below 0.05 was considered as significant.

9.7.1.8 Evaluation of Exploratory Endpoint(S)

The following exploratory efficacy variables were analysed:

- Total and cleaved cytokeratin (CK) 18 plasma levels. Rate of change of CK 18 is defined as for the primary endpoint including the modifications other than LNR;
- Other biomarkers.

9.7.1.9 Sub-Group Evaluation of Efficacy and Exploratory Endpoints

Sub-group analyses of efficacy and exploratory endpoints were performed. The subgroups are defined in the SAP (Appendix 16.1.1).

9.7.1.10 Safety Analyses

Safety was evaluated by the following parameters:

- Adverse events recorded from the timepoint that the PICD had been signed;
- Safety laboratory assessments, including haematology (haemoglobin, haematocrit, complete WBC count, red blood cell [RBC] count platelet count); biochemistry (fibrinogen, alkaline phosphatase, gamma-glutamyltransferase [GGT], total and conjugated bilirubin, ammonia, albumin, creatinine, urea, sodium, potassium, chloride, bicarbonates, lactates, calcium, magnesium, phosphorus, glucose, total proteins, amylase and lipase);
- Vital signs (body temperature, systolic and diastolic blood pressure, heart rate and respiratory rate);
- Physical examination;
- ECG;
- Recording of concomitant medication;
- Beta human chorionic gonadotropin (hCG) blood pregnancy test for females of child-bearing potential.

9.7.1.11 Pharmacokinetic (PK) Evaluation

ALF-5755 plasma concentrations will be performed in the first 16 patients included at Paul Brousse Hospital and Beaujon Hospital and other centres if necessary for adequate recruitment. Baseline plasma concentrations measured at H0, reflecting endogenous HIP/PAP levels will be subtracted from each ALF-5755 plasma concentration. PK samples will be collected at the following times:

- First infusion: H0 just before first infusion, 10 minutes (end of infusion), H1, H2, H6, H12 (just before second infusion);
- Fifth infusion: H48 (just before fifth infusion);
- Sixth infusion: H60 just before sixth infusion, H60 10 minutes (end of infusion), H61, H62, H66, H72, H84.

PK parameters (C_{max}, t_{max}, AUC_{0-12}, AUC_{0-t} and $t_{1/2}$) of ALF-5755 were determined from plasma concentrations measured after first infusion (H0) and after the sixth infusion (H60) and provided to ORION CS for statistical analysis. T_{max} was expected to reflect only the time of the sample collection at the end of infusion.

All PK blood samples (of 5 mL each) were always to be taken from the opposite arm to the one where study treatment was infused.

9.7.2 Determination of Sample Size

The sample size was determined from the Beaujon series for patients that fulfilled the inclusion criteria for this study. The mean (0.22) and standard deviation (0.27) of the PR slope (0-72H) from this series were assumed for the placebo group. The SD for the active group was assumed to be 0.32 (Appendix 16.1.1.) Based on these assumptions, a sample of size 60 (30 per group) would have 80% power to detect a treatment effect of doubling the PR slope (mean slope for ALF-5755 = 0.44) with 5% significance using a two-sided two-sample t-test.

Considering the recruiting rate of eligible patients in the 2 major centres (Beaujon and replicated at Paul Brousse, cf paper Nalpas et al.) and the 8 other centres planned to participate to the trial, this sample size was to be reached within 18 months.

9.8 Changes in the Conduct of the Study or Planned Analyses

9.8.1 Protocol Amendments

The study protocol was amended thrice, the original protocol and all amendments being provided in Appendix 16.1.1. Earlier sections of this report describe the study conduct as amended. The key features of each amendment are given below:

Amendment 1 (11 February 2011)

Amendments included:

- The change from 10 to 19 study centres in France, to 10 to 20 in Europe;
- The change of inclusion criterion no. 1 to ‘A signed written informed consent from patient or from patient's next of kin or from an authorized person according to local procedures’, to allow for different consenting requirements according to local requirements
- Samples of total and cleaved CK18 plasma levels were to be taken only at Paul Brousse and Beaujon Hospitals for logistical reasons;

- ECG abnormalities were recorded;
- The first infusion being within 48 hours (instead of 12) after biological baseline assessment;
- The following assessments for safety reasons:
  - Anti-ALF-5755 antibodies plasma levels at Day 8 and Day 21, whatever the date of discontinuation;
- The following safety information being recorded in the CRF in case of premature discontinuation (Appendix 16.1.1):
  - Liver transplantation, hospitalisation and survival statuses at Day 21;
- Any AEs being followed up until resolution or a period of 18 (change from 21) days from the last dose of the IP had elapsed, whichever was the longer;
- The product being stored for/used within 20 hours (change from 24 hours) upon reconstitution and transfer into the provided syringe following start of reconstitution procedure at +5 ± 3 °C, if not infused within 2 hours after reconstitution.

Amendment 2 (26 July 2011)

Amendments included:

- The maximum age of patients raised from 65 to 75 years in the inclusion criteria;
- The removal of absolute contra-indication to liver transplantation from the exclusion criteria.

Amendment 3 (13 February 2012)

Amendments included:

- For the exploratory endpoint:
  - Sampling of total and cleaved CK18 plasma levels to be performed only at Paul Brousse and Beaujon hospital for logistical reasons;
  - Additional sites being asked to also perform CK18 sampling in the case of low recruitment rate and patient withdrawal during treatment periods to have a minimum of patients for CK18 results interpretation.

ORION
Clinical Services Ltd.

- For the PK endpoints and parameters:
  - Plasma concentrations of ALF-5755 were to be measured as described in the PK Schedule (Appendix 16.1.1), in the first 16 patients included at Paul Brousse Hospital and Beaujon Hospital (8 patients per centre). As a minimum of 8 patients were required per treatment group and in order to replace patient that prematurely discontinue treatment, additional sites could be asked to also perform the PK samples.
- The need for no evidence of underlying cirrhosis (instead of chronic liver disease) in the inclusion criteria;
- The change of inclusion criterion no. 7 to 'Patients who received fresh frozen plasma, PPSB (Prothrombine-Proconvertine-Stuart-B), or vitamin K infusion over the last 24 hours (instead of 48 hours)'
- Prohibited medical treatment:
  - Haemodialysis, hemofiltration or haemodiafiltration treatment were to also be discouraged, but could be deemed necessary as part of symptom management or standard of care. In this last case, the Investigator was instructed whenever possible to perform dialysis sessions before IP infusion and/or at least 6 hours after last IP taking into account half-life of the IP and to avoid the IP filtration.
- For the safety assessments:
  - Sampling was now no longer only performed at the Paul Brousse and Beaujon Hospital for logistical reasons;
  - Concomitant medication and monitoring of AEs at all time points were recorded;
  - Arterial blood gas with lactates at H24 before 3rd infusion, H48 before 5th infusion and H72, in case of grade I or II hepatic encephalopathy or GCS < 15 or if required from investigator's assessment or in case of abnormality at screening.

9.8.2 Other Changes in Study Conduct

- Submission of additional sites to EC in France (June 2011 and October 2011) and in Germany (May 2012);
- Submission of new PI (June 2012).

9.8.3 Changes in Planned Analysis

Changes from Previous Versions of the SAP:

- From Version 1:
  - The use of planned assessment times and LNR=80 in the derivations of the primary efficacy variable has been clarified;
  - The modification using baseline when H0 was not recorded for the primary efficacy variable has been defined;
  - Only hepatitis aetiology will be included as a factor in the efficacy analyses;
  - Details of the survival analysis have been clarified;
  - A subset of the outputs was defined to be produced only on request by Alfact.
- From Version 2:
  - Rate of change of model for end-stage liver disease (MELD) was added as a secondary endpoint;
  - LNR values were added for FV, INR, ALT and AST.

Subgroup analysis: Subgroups were defined based on initial PR, hepatitis etiology, hepatic encephalopathy and NAC treatment (from the concomitant medication page of the CRF). However, the definition was changed after lock to define some or all of the hepatitis etiology subgroups to be identical to the factor levels i.e. patients previously included in more than one subgroup were redefined. The definition of NAC treatment was specified after lock to include treatment before or on the first day of study treatment.

Additional subgroup analysis was requested following database lock. ITT Population with HBV or AIH at baseline and who received 6 infusions of treatment.

Additional post-hoc exploratory analyses were to be performed following review of the main results. These could include:

- Repeating the primary and/or secondary analyses for different combinations of the subgroups;

- Repeating the primary and/or secondary analyses for biomarker subgroups e.g. based on CK18, IL6, GC globulin, HIP/PAP at admission.

Complementary analyses: After the unblinding of the study data, Alfact decided to have additional efficacy analyses Variables included:

- Rate of change of PR from 0-12 h;
- Other time-points for efficacy markers (i.e., Day 8 and Day 21);
- Comparison of patient liver function status at inclusion according to population (ALF-5755 versus placebo in HBV AIH subgroup)
- Analysis of time to achieve PR of 50% for HBV AIH;
- Plots of total bilirubin and GCGlobulin at baseline for patients who died or were transplanted compared to patients who were alive without transplantation at the end of the study.

ORION
Clinical Services Ltd.

10 STUDY PATIENTS

10.1 Disposition of Patients

This was a multi-centre study with 60 patients planned to be enrolled. A total of 61 patients were screened, 59 randomised treated, and 45 completed the study. Patient disposition is summarised in Table 10-1~~Table 10-1~~ (Table 14.1.1).

Table 10-1: Patient Disposition (All Available Patients)

	ALF-5755 (N=30)	Placebo (N=29)	Total (N=61)
Screening			61
Randomised	30 (100.0%)	29 (100.0%)	59
Safety Population	28 (93.3%)	29 (100.0%)	57
ITT Population	28 (93.3%)	29 (100.0%)	57
PP Population	28 (93.3%)	28 (96.6%)	56
Completed	21 (70.0%)	24 (82.8%)	45

Source: Table 14.1.1

Of the 45 patients who completed the study, 21 patients were from the ALF-5755 group and 24 from the placebo group (Table 10-2~~Table 10-2~~; Table 14.1.2; Listing 16.2.1). There were nine withdrawals in the ALF-5755 group: three patients required a liver transplant, two patients were lost to follow-up, and one patient each because of a SAE, death, Investigator's decision, and one patient (Patient 0205) was randomised to ALF-5755 in error and had no documented reason for withdrawal and was therefore recorded as missing. There were five withdrawals in the placebo group, two because of SAEs, and one patient each because consent was withdrawn, death, and due to requiring a liver transplant.

Table 10-2: Study Termination and Primary Reason for Withdrawal (All Randomised Patients)

	ALF-5755 (N=30)	Placebo (N=29)	Total (N=59)
Completed	21 (70.0%)	24 (82.8%)	45 (76.3%)
Early withdrawal	9 (30.0%)	5 (17.2%)	14 (23.7%)
Main reason for early withdrawal			
Withdrawn Consent	0	1 (3.4%)	1 (1.7%)
Serious Adverse Event	1 (3.3%)	2 (6.9%)	3 (5.1%)
Death	1 (3.3%)	1 (3.4%)	2 (3.4%)
Investigators Decision	1 (3.3%)	0	1 (1.7%)
Lost to Follow-Up	2 (6.7%)	0	2 (3.4%)
Required Liver Transplant	3 (10.0%)	1 (3.4%)	4 (6.8%)
*Missing**	1 (3.3%)	0	1 (1.7%)

Source: Table 14.1.2

*Patient 02-05 was randomised to ALF-5755 in error and had no documented reason for withdrawal

The final status of all patients is presented in Listing 16.4.1; visit dates are presented in Listing 16.4.2.

10.2 Protocol Deviations

Full details of all protocol deviations as agreed at the blind data review meeting on are summarised in Listing 16.2.2.

Of the 59 patients randomised, one patient (0407) from the ITT population was reported with one major protocol deviation, who received less than 5 complete infusions, and 31 patients from the ITT population were reported with minor protocol deviations. Eligibility confirmation and randomisation status are presented in Listing 16.4.3.3.

Protocol waivers were completed for six patients, but were not considered major protocol deviations:

- Patient M-F arrived at the emergency department with a TP value of 33% of 20Feb2011 at 21:00. Aetiology had not yet been confirmed but was thought to be viral hepatitis A. The patient was transferred to the hepatology department on the morning of the following day. The TP value at that time was 39%. The patient was given 10 mg vitamin K, an exclusion criterion. The patient recovered after one dose.

ORION Clinical Services Ltd.

- Patient L-N was reported with TP value of 17% on 29Mar2011. Alfact Innovation was contacted by the Investigator for the patient's eligibility and were told that the patient was eligible and that treatment had to start within 48 hours. For organisational reasons, perfusion was started the next day. A second complete blood analysis was performed on 29Mar2011 to have a baseline performed within the 12 hours before the first perfusion.

- Patient 0202 was admitted to hospital on 24Oct2010 at around 04:00 (first PR at 04:40 was 19%). He was not included in the study at that time because of suspected cirrhosis. On 26Oct2010 around 15:30, the site obtained the confirmation that the patient was not cirrhotic (biopsy and scanner). The Investigator contacted Alfact Innovation to find out if this patient was eligible, even though the time window between biological baseline assessments (PR at admission) and start of study drug would be more than 12 hours. Alfact Innovation authorised the inclusion of the patient because the biological baseline assessments were considered when the site had the confirmation that the patient was not cirrhotic. It was therefore decided to include the patient despite inclusion criterion 3 not having been respected. Patient 0202 received the first drug administration on 26Oct2010 at 21:30.

- Patient 0203 was admitted to hospital on the afternoon of 17Feb2011. The first injection was planned on the afternoon of 18Feb2011, i.e. more than 24 hours after baseline assessment. The Investigator contacted ORION to receive confirmation that the patient could be included. Alfact Innovation authorised the inclusion of the patient on 18Feb2011.

- Patient 0309 had a creatinine level of 185 µmol/L at baseline, fulfilling Exclusion Criterion No.3. The Investigator contacted Alfact Innovation to discuss the case, and because the creatinine level decreased (actual level at H0 just before infusion was 155 µmol/L), Alfact Innovation authorised the inclusion of the patient in the study.

- Patient 0407 received 10 mg of vitamin K on 11Dec2011. Screening and H0 were performed on 12Dec2011. Exclusion Criterion No.7 was not respected, however Alfact Innovation authorised the inclusion of the patient in the study.

11 EFFICACY EVALUATION

All Tables can be found in-text or in Section 14 and all Listings can be found in Appendix 16.2.

11.1 Data Sets Analysed

Study populations are defined in Section 9.7.1.1.

Patient inclusion in the analysis sets is presented in Listing 16.2.3, and inclusion and exclusion criteria are presented by patient in Listings 16.4.3.1 and 16.4.3.2, respectively.

11.2 Demographic and Other Baseline Characteristics

Patient demographics at baseline are summarized by treatment sequence in Table 11-1~~Table 11-1~~ (Table 14.1.3.1; Listing 16.2.4.1). There was a slight imbalance in baseline characteristics between the treatment groups. The mean age of the patients in the ALF-5755 group was higher compared to the placebo group (43.51 years versus 40.11 years); there were more males in the ALF-5755 group compared to the placebo group (19 versus 14); less females in the ALF-5755 group compared to the placebo group (9 versus 15); more white Caucasians in the ALF-5755 group compared to the placebo group (27 versus 19); patients were slightly taller in the ALF-5755 group compared to the placebo group (173.31 cm versus 169.2 cm); patients were slightly heavier in the ALF-5755 group compared to the placebo group (74.75 kg versus 67.26 kg); and patients had slightly higher BMI in the ALF-5755 group compared to the placebo group (24.5 kg/m² versus 23.3 kg/m²).

ORION
Clinical Services Ltd.

Table 11-1: Demographics and Baseline Characteristics (ITT Population)

		ALF-5755 (N=28)	Placebo (N=29)	Total (N=57)
Age (years)	N	28	29	57
	Mean (SD)	43.51 (4.9)	40.11 (5.3)	41.8 (15.1)
	Range	18 - 65	19- 66	18 - 66
Gender	Male	19 (67.9%)	14 (48.3%)	33 (57.9%)
	Female	9 (32.1%)	15 (51.7%)	24 (42.1%)
Race	White/Caucasian	27 (96.4%)	19 (65.5%)	46 (80.7%)
	Black	0	5 (17.2%)	5 (8.8%)
	Asian	0	1 (3.4%)	1 (1.8%)
	Other	1 (3.6%)	4 (13.8%)	5 (8.8%)
	Hispanic	0	1 (3.4%)	1 (1.8%)
	Maghreb	0	1 (3.4%)	1 (1.8%)
	North Africa	1 (3.6%)	2 (6.9%)	3 (5.3%)
Height (cm)	N	28	29	57
	Mean (SD)	173.71 (9.62)	169.2 (19.12)	171.42 (9.56)
	Range	157.01 - 92.0	155.0 - 194.0	155.0 - 194.0
Weight (kg)	N	28	29	57
	Mean (SD)	74.75 (9.12)	67.26 (14.88)	70.94 (17.36)
	Range	38.0 - 118.0	32.0 - 99.5	32.0 - 118.0
BMI (kg/m²)	N	28	29	57
	Mean (SD)	24.5 (5.0)	23.3 (4.4)	23.9 (4.7)
	Range	14 - 34	12 - 35	12- -35

Source: Table 14.1.3.1

Patients in both treatment groups were generally similar with respect to hepatitis aetiology, hepatic encephalopathy and NAC treatment (Table 11-2~~Table 11-2~~; Table 14.1.3.2; Listings 16.2.4.2 and 16.2.4.3).

Table 11-2: Baseline Factors (ITT Population)

		ALF-5755 (N=28)	Placebo (N=29)	Total (N=57)
Hepatitis Aetiology	Hepatitis A	4 (14.3%)	6 (20.7%)	10 (17.5%)
	Hepatitis B	9 (32.1%)	4 (13.8%)	13 (22.8%)
	Hepatitis E	3 (10.7%)	3 (10.3%)	6 (10.5%)
	AIH	4 (14.3%)	5 (17.2%)	9 (15.8%)
	Other aetiology	1 (3.6%)	2 (6.9%)	3 (5.3%)
	Undetermined aetiology	3 (10.7%)	5 (17.2%)	8 (14.0%)
	Drug-induced hepatitis	4 (14.3%)	4 (13.8%)	8 (14.0%)
Hepatic encephalopathy	Grade 0	25 (89.3%)	26 (89.7%)	51 (89.5%)
	Grade I	2 (7.1%)	3 (10.3%)	5 (8.8%)
	Grade II	1 (3.6%)	0	1 (1.8%)
NAC treatment	Yes	22 (78.6%)	24 (82.8%)	46 (80.7%)
	No	6 (21.4%)	5 (17.2%)	11 (19.3%)

Source: Table 14.1.3.2

Table 11-3~~Table 11-3~~ presents Baseline biological markers for liver function for the ITT population, whilst Table 11-4 presents Baseline biological markers for liver function for patients within the ITT Population with HBV or AIH.

All biological markers for liver function for the ITT Population with HBV or AIH show that patients assigned to the ALF-5755 arm were in a more severe condition at baseline (refer to Table 11-4). Refer also to Figure 11-1 for the plot of baseline total bilirubin. Only patients assigned to the ALF-5755 arm had total bilirubin values > 500, which also indicates a more severe condition at baseline (compared to the placebo arm).

ORION
Clinical Services Ltd.

Table 11-3: Baseline biological markers for liver function: INR, MELD, ALT, AST, Total Bilirubin, PR (ITT Population)

		ALF-5755	Placebo
INR	N	27	28
	Mean (SD)	2.3881 (0.8324)	2.4636 (0.8352)
	Range	1.500 - 4.530	1.200 - 4.410
MELD	N	27	28
	Mean (SD)	25.78 (6.80)	25.03 (6.28)
	Range	13.6 - 41.5	14.4 - 41.8
ALT	N	28	29
	Mean (SD)	2495.7 (1841.8)	3274.6 (2135.8)
	Range	190 - 7215	415- 7951
AST	N	28	29
	Mean (SD)	1887.6 (2085.4)	3465.8 (4984.9)
	Range	98 - 10981	123 – 27033
Conjugated Bilirubin	N	28	29
	Mean (SD)	211.0 (152.0)	162.8 (123.1)
	Range	4 - 433	4 – 412
Total Bilirubin	N	28	29
	Mean (SD)	294.6 (202.4)	229.6 (164.0)
	Range	8- 612	18- 511
PR	N	28	29
	Mean (SD)	34.6 (9.0)	33.6 (11.2)
	Range	16- 48	17- 64

Source: Tables 14.2.2.2, 14.2.2.3, 14.2.2.4, 14.2.2.5, 14.2.2.6 14.2.2.7, 14.2.1.1

ORION
Clinical Services Ltd.

Table 11-4: Baseline biological markers for liver function: INR, MELD, ALT, AST, Total Bilirubin, PR (ITT Population with HBV or AIH)

		ALF-5755 (N=13)	Placebo (N=9)
INR	Mean (SD)	2.6015 (0.9989)	2.4756 (0.9889)
	Range	1.500 - 4.530	1.200- 3.990
MELD	Mean (SD)	29.98 (6.19)	26.62 (5.95)
	Range	22.8- 41.5	16.0- 34.0
ALT	Mean (SD)	2307.8 (1781.6)	1491.2 (912.3)
	Range	369- 5548	537- 2899
AST	Mean (SD)	1535.0 (1111.8)	1149.2 (546.6)
	Range	350- 3490	376- 2167
Conjugated Bilirubin	Mean (SD)	314.8 (84.0)	230.0 (91.8)
	Range	151 - 433	99 - 372
Total Bilirubin	Mean (SD)	440.9 (115.8)	327.6 (127.0)
	Range	236- 612	125- 495
PR	Mean (SD)	32.4 (9.8)	35.6 (14.3)
	Range	16- 46	17- 64

Source: Tables 14.2.2.14, 14.2.2.15, 14.2.2.16, 14.2.2.17, 14.2.2.18, 14.2.2.19, 14.2.1.1.18

Figure 11-1: Plot of Baseline Total Bilirubin (ITT Population with HBV or AIH)

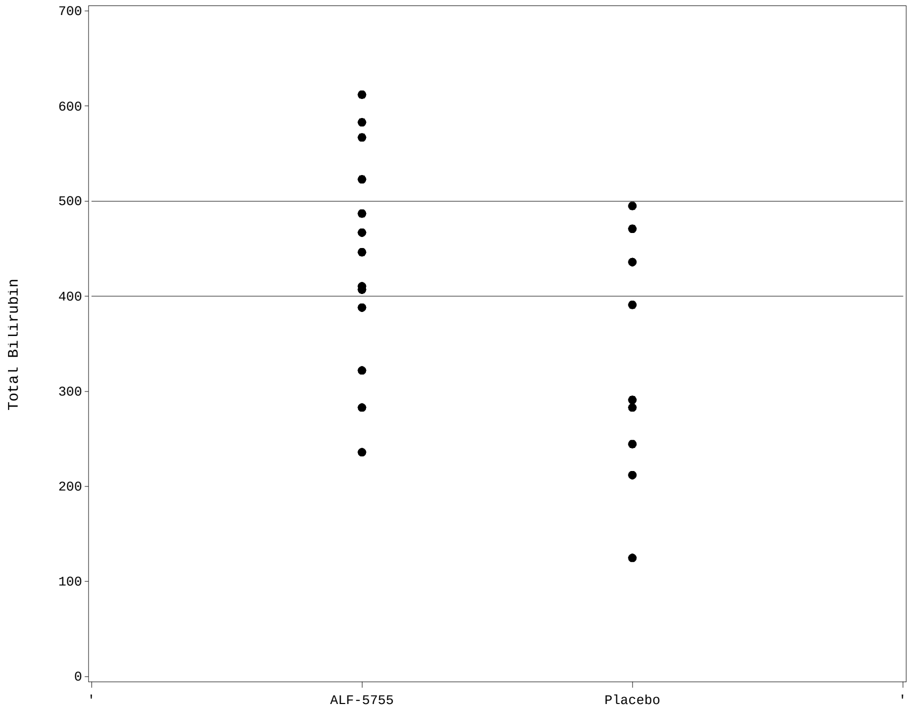

Source: Figure 6.1

Baseline serology data is presented in Listing 16.2.4.4, and medical history is presented in Listing 16.4.4. Concomitant medications, procedures and surgeries are presented in Listings 16.4.5.1, 16.4.5.2 and 16.4.5.3, respectively.

11.3 Measurements of Treatment Compliance

Treatment was by infusion so only exposure was reported.

11.4 Efficacy Results and Tabulations of Individual Patient Data

Efficacy response data is presented in Listing 16.2.6.

11.4.1 Analysis of Efficacy

Analysis of PR (0-72 hours) in patients with viral hepatitis E or other aetiology, undetermined aetiology or drug-induced aetiology is presented in Table 14.2.1.1.2.

11.4.1.1 ITT population

11.4.1.1.1 Primary Efficacy Analysis: Analysis of Prothrombin Ratio (PR) (0-72 hours)

Analysis of PR from 0 to 72 hours for the ITT population is presented in Table **11-5**~~Table **11-5**~~ (Table 14.2.1.1).

The median rate of change of PR in the ALF-5755 group was 0.083, compared to 0.097 in the placebo group. This difference was not significant at the 5% level for both the Wilcoxon and CMH tests (p=0.4577 and 0.8876, respectively).

Table 11-5: Analysis of Prothrombin Ratio (PR) (0-72 hours) (ITT Population)

		**ALF-5755 (N=28)**	**Placebo (N=29)**	**p-value**
Rate of change of PR	N	28	29	
	Mean (SD)	0.188 (0.320)	0.257 (0.326)	
	Median	0.083	0.097	
	Range	-0.25 - 1.31	-0.25 - 1.02	
Wilcoxon test				0.4577
CMH test adjusting for aetiology[1]				0.8876

Source: Table 14.2.1.1
[1] Hepatitis aetiology factor levels: Viral Hepatitis A; Viral Hepatitis B or Autoimmune Hepatitis; Other
SD: Standard deviation

Analysis of PR from 0 to 72 hours of the PP population is presented in Table 14.2.2.1. The results were similar to those from the ITT population.

Median change of PR from baseline (0) to 72 hours is illustrated in Figure 1.1 (refer to Section 14) for the ITT population.

ORION
Clinical Services Ltd.

Mean change of PR from baseline to Day 21 is illustrated in Figure 7.1 (refer to

Figure 11-6Figure 11-6). Within the ITT population, the mean change of PR is comparable between the ALF-5755 treated group and the Placebo group. However, at 12 hours, PR was increased in the ALF-5755 group, indicating a strong efficacy of the first dose of treatment with ALF-5755.

11.4.1.1.2 Secondary Efficacy Analysis

Analysis of PR at different time points, with differing aetiologies, varying types of hepatitis and number of infusions are presented in Tables 14.2.1.1.17 to 14.2.1.1.30.

11.4.1.1.2.1 *Analysis of International Normalized Ratio (INR) (0-72 hours)*

Analysis of INR from 0 to 72 hours is presented in Table 11-6Table 11-6 (Table 14.2.2.2) for the ITT population and the rate of change of INR (0-72 hours) is presented per patient in Listing 16.2.6.

For the ITT population, the median rate of change of INR in the ALF-5755 group was -0.00278, compared to -0.00632 in the placebo group. This difference was not significant at the 5% level for both the Wilcoxon and CMH tests (p=0.2701 and 0.2376, respectively) (refer to Table 11-6Table 11-6).

Table 11-6: Analysis of International Normalized Ratio (INR) (0-72 hours) (ITT Population)

		ALF-5755 (N=28)	Placebo (N=29)	p-value
Rate of change of INR	N	27	28	
	Mean (SD)	-0.00062 (0.01819)	-0.00748 (0.01183)	
	Median	-0.00278	-0.00632	
	Range	-0.0278 - 0.0571	-0.0403 - 0.0142	
Wilcoxon test				0.2701
CMH test adjusting for aetiology[1]				0.2376

Source: Table 14.2.2.2
[1] Hepatitis aetiology factor levels: Viral Hepatitis A; Viral Hepatitis B or Autoimmune Hepatitis; Other
SD: Standard deviation

Median change of INR from baseline (0) to 72 hours in the ITT population is illustrated in Table 14.2.2.2/Figure 1.3 (refer to Section 14).

Mean change of INR from baseline to Day 21 is illustrated in Figure 7.2 (refer to Figure 11-7~~Figure 11-7~~). Mean change of INR over the first 72 hours following infusion was comparable between the ALF-5755 treated group and the Placebo group.

11.4.1.1.2.2 *Analysis Model for End Stage Liver Disease (MELD) (0-72 hours)*

Analysis of MELD from 0 to 72 hours is presented in Table 11-7~~Table 11-7~~ (Table 14.2.2.3) for the ITT population and the rate of change of MELD (0-72 hours) is presented per patient in Listing 16.2.6.

For the ITT population, the median rate of change of MELD in the ALF-5755 group was -0.015, compared to -0.037 in the placebo group. This difference was not significant at the 5% level for both the Wilcoxon and CMH tests (p=0.1835 and 0.1582, respectively) (refer to Table 11-7~~Table 11-7~~).

Table 11-7: Analysis of Model for End-stage Liver Disease (MELD) (0-72 hours) (ITT Population)

		ALF-5755 (N=28)	Placebo (N=29)	p-value
Rate of change of MELD	N	27	28	
	Mean (SD)	-0.004 (0.113)	--0.049 (0.059)	
	Median	-0.015	-0.037	
	Range	-0.15 - 0.44	-0.18 - 0.03	
Wilcoxon test				0.1835
CMH test adjusting for aetiology[1]				0.1582

Source: Table 14.2.2.3
[1] Hepatitis aetiology factor levels: Viral Hepatitis A; Viral Hepatitis B or Autoimmune Hepatitis; Other
SD: Standard deviation

Analysis of MELD from 0 to 72 hours is presented in Table 14.2.2.9 for the Completed Treatment population.

Median change of MELD from baseline (0) to 72 hours in the ITT Population is illustrated in Table 14.2.2.3/Figure 1.4 (refer to Section 14).

A decrease in mean values of MELD can be observed in both the ALF-5755 treated group and the Placebo group that is seen as comparable (refer to Figure 4 in Section 14).

ORION
Clinical Services Ltd.

11.4.1.1.2.3 *Total bilirubin, Conjugated bilirubin, Alanine Transaminase (ALT) and Aspartate Transaminase (AST)*

The analysis of total bilirubin (0-72 hours) is presented Table 14.2.2.7 for the ITT population and in Table 14.2.2.13 for the Completed Treatment population. The rate of change of total bilirubin (0-72 hours) is presented per patient in Listing 16.2.6.

The analysis of conjugated bilirubin (0-72 hours) is presented in Table 14.2.2.6 for the ITT population and in Table 14.2.2.12 for the Completed Treatment population. The rate of change of conjugated bilirubin (0-72 hours) is presented per patient in Listing 16.2.6.

The analysis of ALT from 0 to 72 hours is presented in Table 14.2.2.4 for the ITT population and in Table 14.2.2.10 for the Completed Treatment population. The rate of change of ALT (0-72 hours) is presented per patient in Listing 16.2.6.

The analysis of AST from 0 to 72 hours is presented in Table 14.2.2.5 for the ITT population, in Table 14.2.2.11 for the Completed Treatment population and in Table 14.2.2.17 for ITT patients with HBV or AIH. The rate of change of AST (0-72 hours) is presented per patient in Listing 16.2.6.

Although differences between ALF-5755 treatment and placebo were noted in some instances, these were not considered significant in most instances and, in general, results for total bilirubin, conjugated bilirubin, ALT and AST do not provide any evidence of improvement with ALF-5755 treatment.

11.4.1.1.2.4 *Analysis of Plasma Levels of Total Cytokeratin 18 (CK18)*

Analysis of plasma levels of total CK18 is presented in Table 11-8~~Table 11-8~~ (Table 14.2.6.1).

The median rate of change of total CK18 in the ALF-5755 group was -85.266, compared to -65.059 in the placebo group. This difference was not significant at the 5% level for the Wilcoxon test (p=0.9734).

ORION Clinical Services Ltd.

Table 11-8: Total CK18 (ITT Population)

		ALF-5755 (N=28)	Placebo (N=29)	p-value
Rate of change of total CK18	N	9	13	
	Mean (SD)	-138.237 (197.351)	-147.952 (182.974)	
	Median	-85.266	-65.059	
	Range	-640.40 - 15.19	-586.32 - -18.03	
Wilcoxon test				0.9734

Source: Table 14.2.6.1
SD: Standard deviation

Analysis of Plasma Levels of Cleaved Creatinine Kinase (CK18) Analysis of plasma levels of cleaved CK18 is presented in Table 11-9~~Table 11-9~~ (Table 14.2.6.2).

The median rate of change of cleaved CK18 in the ALF-5755 group was -27.619, compared to -19.221 in the placebo group. This difference was not significant at the 5% level for the Wilcoxon test (p=0.5121).

Table 11-9: Cleaved CK18 (ITT Population)

		ALF-5755 (N=28)	Placebo (N=29)	p-value
Rate of change of Cleaved CK18	N	6	12	
	Mean (SD)	-43.903 (47.692)	-39.792 (55.491)	
	Median	-27.619	-19.221	
	Range	-137.79 - -9.68	1.06 - -201.58	
Wilcoxon test				0.5121

Source: Table 14.2.6.2
SD: Standard deviation

CK18 sampling data is presented in Listing 16.4.8.

11.4.1.1.2.5 *Analysis of Survival, Transplant-free Survival and Time to Transplantation*

A Kaplan-Meier plot of survival is illustrated in (Listing 16.4.6.4/Figure 2.1 [refer to Section 14]). Overall, the decrease in survival over a period of 21 days was the same for both treatment groups (one death in each group).

Figure 11-2: Kaplan-Meier Plot of Survival (ITT Population)

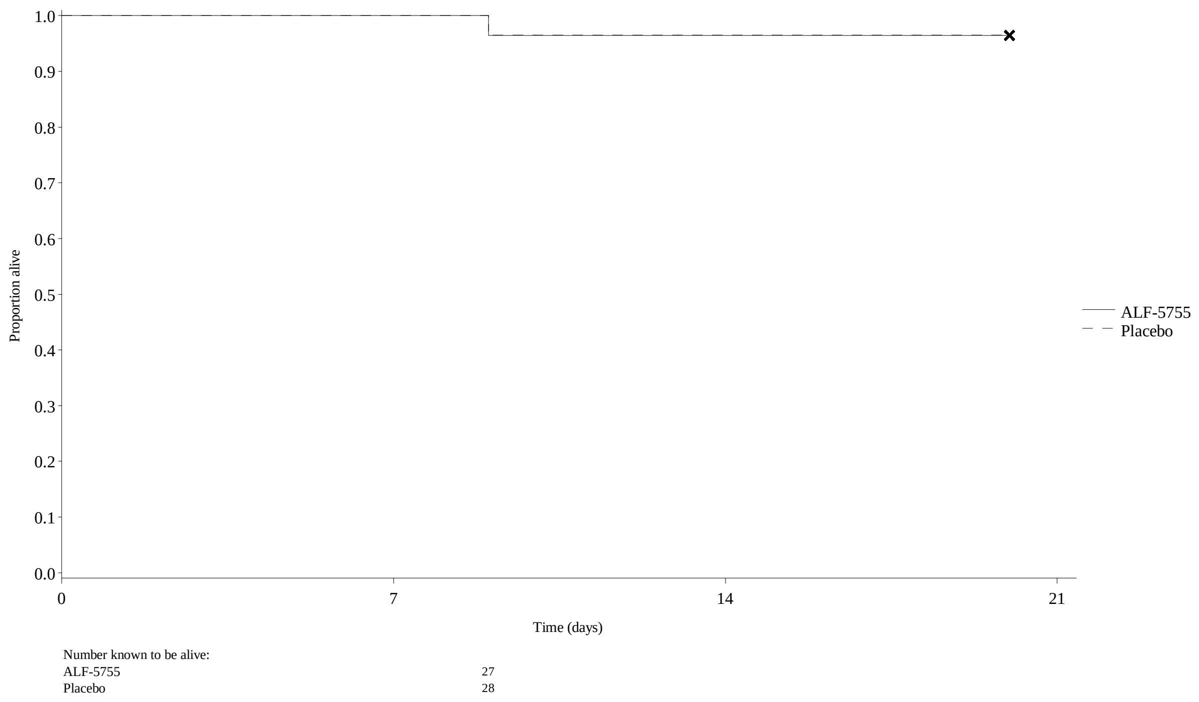

Source: Figure 2.1

Analysis of transplant-free survival until Day 21 is presented in Table 11-10~~Table 11-10~~ (Table 14.2.4.2; Listing 16.4.6.2).

A total of 21 (75%) of the 28 patients in the ALF-5755 group survived transplant-free to Day 21 compared to 25 (86.2%) of 29 patients in the placebo group. This difference was not significant at the 5% level for the Chi-square test (p=0.2838). However, the highest number of patients with total bilirubin >500 (i.e. most severe patients) were in the ALF-5755 group at Baseline.

Stratification of patients according to GCglobulin showed that no liver transplants were required in patients treated with ALF-5755 group if GCglob>155 (0 of the 15 patients in the ALF-5755 group versus 2 of 14 patients in the placebo group).

Figure 11-3: Plot of Baseline GCGlobulin (ITT Population)

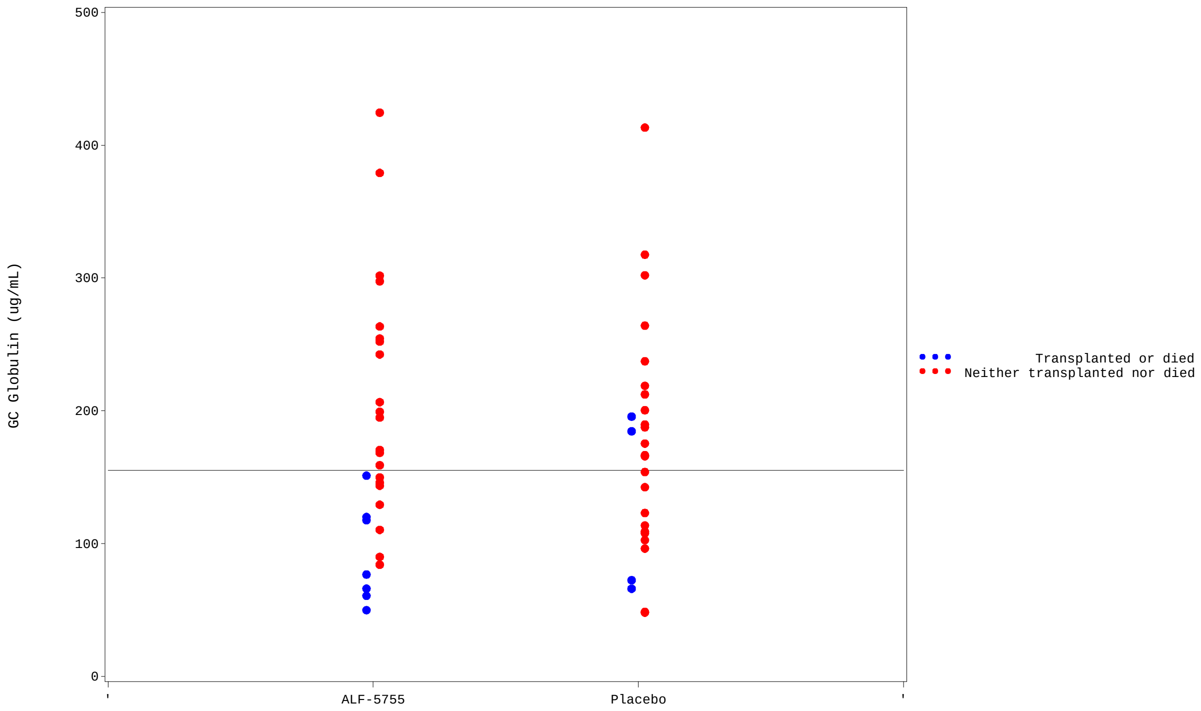

Source: Figure 6.4

Stratification of patients according to total bilirubin showed that liver transplants was decreased by 2 in patients treated with ALF-5755 group if tBili<400 (1 of the 14 patients in the ALF-5755 group versus 3 of 23 patients in the placebo group).

Figure 11-4: Plot of Baseline Total Bilirubin (ITT Population)

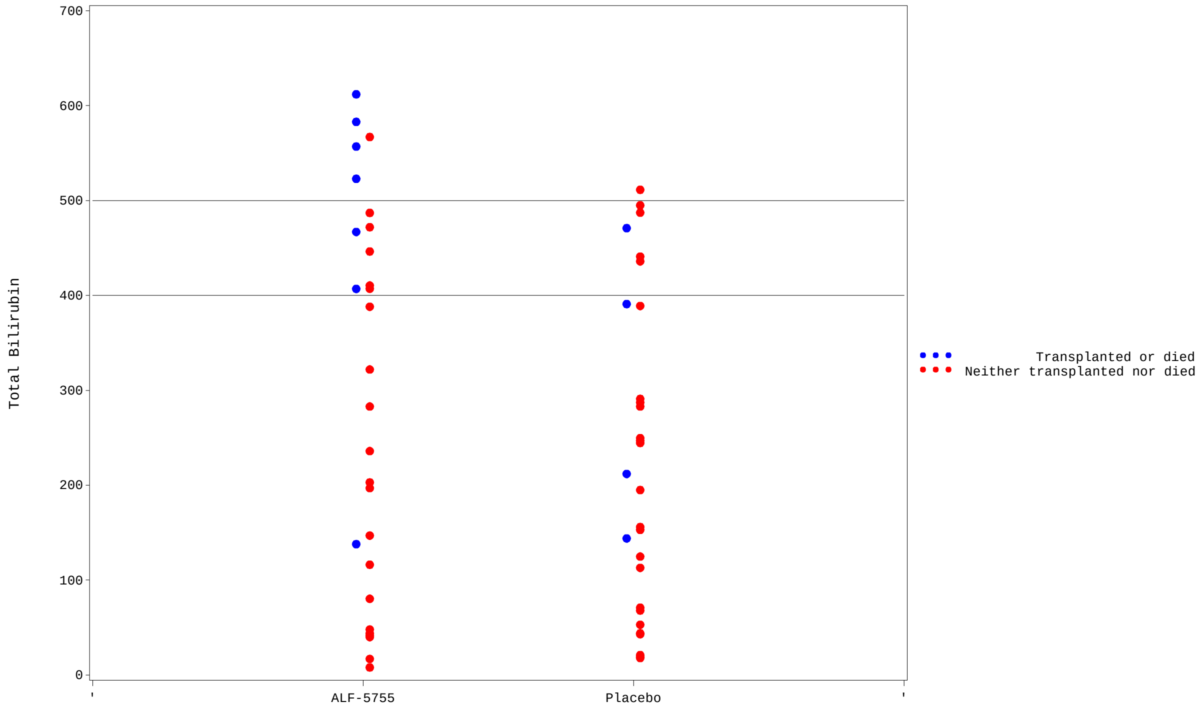

Source: Figure 6.3

The Kaplan-Meier estimates for the lower quartile (95% CI lower bound) were 2.0 and 9.0 days for ALF-5755 and placebo groups, respectively. The difference was not significant at the 5% level for the log-rank test (p=0.2541). Responses were recorded in 7 (25.0%) and 4 (13.8%) patients in the ALF-5755 and placebo groups, respectively. A Cox regression analysis gave a hazard ratio of 1.644 (95% CI: 0.477, 5.661). This difference was not significant at the 5% level (p=0.4308).

Table 11-10: Transplant-Free Survival (ITT Population)

		ALF-5755 (N=28)	Placebo (N=29)	p-value
Transplant-Free and Survived to Day 21	Yes	21 (75.0%)	25 (86.2%)	
	No	7 (25.0%)	4 (13.8%)	
	Chi-squared test			0.2838
Kaplan-Meier estimate (days)	Lower quartile 95% CI lower bound	2.0	9.0	
	Log Rank Test			0.2541
	Number (%) of responses	7 (25.0%)	4 (13.8%)	
	Number (%) censored	21 (75.0%)	25 (86.2%)	
Cox regression analysis adjusting for hepatitis aetiology[1]	Hazard ratio			1.644
	95% CI			0.477, 5.661
	p-value			0.4308

Source: Table 14.2.4.2

[1] Hepatitis aetiology factor levels: Viral Hepatitis A; Viral Hepatitis B or Autoimmune Hepatitis; Other

HR<1 corresponds to longer transplant-free survival in the ALF-5755 treatment group

A Kaplan-Meier plot of transplant-free survival is illustrated in Figure 11-5~~Figure 11-5~~ (Listings 16.4.6.2 and 16.4.6.4/Figure 2.2 [refer to Section 14]).

The transplant-free survival rate over a period of 21 days was lower for the ALF-5755 group (75.0%) compared to placebo group (86.2%).

There was no significant difference between the groups in transplant-free survival (log-rank test: p = 0.2541). The aetiology adjusted hazard ratio for ALF-5755 versus placebo was 1.644 (95% CI 0.477, 5.661).

Figure 11-5: Kaplan-Meier Plot of Transplant-Free Survival (ITT Population)

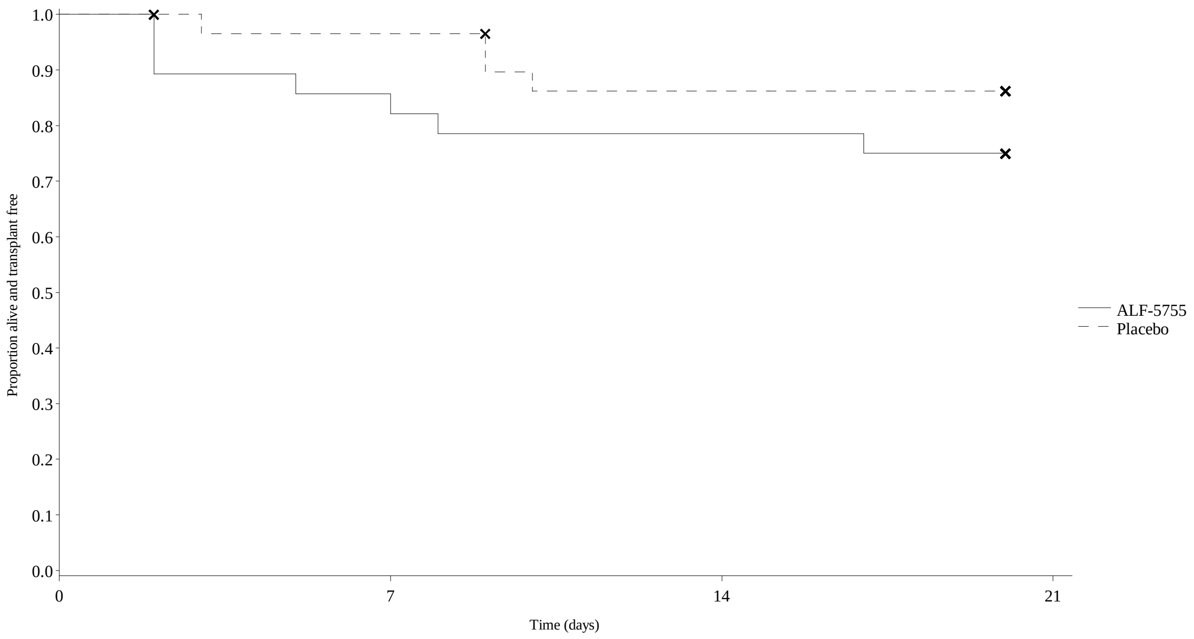

Number known to be alive and transplant free:

ALF-5755	25	25	24	23	22	22	22	21
Placebo	29	28	28	28	28	26	25	25

X indicates censored times

Source: Figure 2.2

Analysis of time to transplantation until Day 21 is presented in Table 11-11~~Table 11-11~~ (Table 14.2.4.3).

A total of 21 (75%) of the 28 patients in the ALF-5755 group survived to Day 21 and to Day 21 with no transplant performed, compared to 25 (86.2%) of 29 patients in the placebo group. These differences were not significant at the 5% level for the Chi-square test (p=0.2838 for both). The Kaplan-Meier estimates for the lower quartile (95% CI lower bound) were 2.0 and 10.0 days for ALF-5755 and placebo, respectively. This difference was not significant at the 5% level for the log-rank test (p=0.1404). Responses were recorded in 7 (25.0%) and 4 (10.3%) patients in the ALF-5755 and placebo groups, respectively. A Cox regression analysis gave a hazard ratio of 2.217 (95% CI: 0.568, 8.646). This difference was not significant at the 5% level (p=0.2517).

Table 11-11: Time to Transplantation (ITT Population)

		ALF-5755 (N=28)	Placebo (N=29)	p-value
Survived to Day 21 with no transplant required or performed	Yes	21 (75.0%)	25 (86.2%)	
	No	7 (25.0%)	4 (13.8%)	
	Chi-squared test			0.2838
Kaplan-Meier estimate (days)	Lower quartile 95% CI lower bound	2.0	10.0	
	Log Rank Test			0.1404
	Number (%) of responses	7 (25.0%)	3 (10.3%)	
	Number (%) censored	21 (75.0%)	26 (89.7%)	
Cox regression analysis adjusting for hepatitis aetiology[1]	Hazard ratio			2.217
	95% CI			0.568, 8.646
	p-value			0.2517

Source: Table 14.2.4.3

[1] Hepatitis aetiology factor levels: Viral Hepatitis A; Viral Hepatitis B or Autoimmune Hepatitis; Other

HR<1 corresponds to longer transplant-free survival in the ALF-5755 treatment group

A Kaplan-Meier plot of time to transplantation is illustrated in Listing 16.4.6.2 and Figure 2.3 (refer to Section 14).

The time to transplantation over a period of 21 days was lower for the ALF 5755 group (75.0%) compared to placebo group (86.2%).

There was no significant difference between the groups in time to transplantation (log-rank test: p = 0.1404). The aetiology adjusted hazard ratio for ALF-5755 versus placebo was 2.217 (95% CI 0.568, 8.646).

11.4.1.1.2.6 *Hospital-free days and length of hospitalisation*

Analysis of hospital-free days is presented in Table 11-12~~Table 11-12~~ (Table 14.2.5.1).

The median number of hospital-free days in the ALF-5755 group was 12.00, compared to 9.00 in the placebo group. This difference was not significant at the 5% level for the Wilcoxon test (p=0.564).

Table 11-12: Hospital-Free Days (ITT Population)

		ALF-5755 (N=28)	Placebo (N=29)	p-value
Hospital-free days up to D21[1]	N	28	29	
	Mean (SD)	8.89 (6.57)	7.76 (6.68)	
	Median	12.00	9.00	
	Range	0.0 - 16.0	0.0 - 17.0	
	Wilcoxon test			0.564

Source: Table 14.2.5.1

[1] Days alive following hospital discharge

For the ITT population (alive with no transplant), the mean number of days in the hospital was lower the ALF-5755 group (9.14 days) compared to the placebo group (12.00 days). The difference in days in hospital was not significant at the 5% level for the Wilcoxon test (p=0.169).

Table 11-13: Days in Hospital (ITT Population, Alive with No Liver Transplant)

		ALF-5755 (N=21)	Placebo (N=25)	p-value
Days in Hospital up to D21[1]	N	21	25	
	Mean (SD)	9.14 (4.63)	12.00 (6.36)	
	Median	8	11	
	Range	5 - 21	4 - 21	
	Wilcoxon test			0.169

Source: Table 14.2.5.2

[1] Up to and including date of hospital discharge, maximum of 21 days.

Analysis of days in hospital is presented in Table 14.2.5.3 for Completed Treatment patients who were alive with no liver transplant. For the Completed Treatment Population, the mean number of days in the hospital was lower the ALF-5755 group (9.14 days) compared to the placebo group (11.96 days). The difference in days in hospital was not significant at the 5% level for the Wilcoxon test (p=0.139).

The days in hospital is presented per patient in Listing 16.2.6.

11.4.1.1.3 Complementary analyses

11.4.1.1.3.1 *Analysis of Prothrombin Ratio (PR) Early Efficacy: 0-12 hours*

Early efficacy:

Median values recorded for analysis of PR 0-12 hours for the ITT Population showed an increase in ALF-5755 treated group (0.292) when compared to Placebo group (0.167), although of no significance due to large standard deviations.

Table 11-14: Analysis of PR (0-12 hours) (ITT Population)

		ALF-5755 (N=28)	Placebo (N=29)	p-value
Rate of change of PR	N	27	29	
	Mean (SD)	0.356 (0.419)	0.187 (0.517)	
	Median	0.292	0.167	
	Range	-0.17- 1.58	-1.00- 1.42	
Wilcoxon test				0.1724
CMH test adjusting for aetiology[1]				0.0585

[1] Hepatitis etiology factor levels: Viral Hepatitis A; Viral Hepatitis B or Autoimmune Hepatitis; Other
Source: Table 14.2.1.1.23
SD: Standard deviation

11.4.1.1.3.2 *Analysis of Prothrombin Ratio (PR) Long Term Efficacy: 0-Day 8*

Long term efficacy:

Median values recorded for analysis of PR 0-Day 8 for the ITT Population showed no real differences between the ALF-5755 treated group (0.151) when compared to the Placebo group (0.203).

Table 11-15: Analysis of PR (0-Day 8) (ITT Population)

		ALF-5755 (N=28)	Placebo (N=29)	p-value
Rate of change of PR	N	28	29	
	Mean (SD)	0.205 (0.305)	0.253 (0.286)	
	Median	0.151	0.203	
	Range	-0.25- 1.31	-0.08- 1.02	
Wilcoxon test				0.5388
CMH test adjusting for aetiology[1]				0.9735

[1] Hepatitis etiology factor levels: Viral Hepatitis A; Viral Hepatitis B or Autoimmune Hepatitis; Other

Source: Table 14.2.1.1.27

SD: Standard deviation

11.4.1.1.3.3 *Prothrombin Ratio (PR) and International Normalized Ratio (INR)*

Refer to

Figure 11-6Figure 11-6 and Figure 11-7Figure 11-7 for the mean plot of PR and INR to Day 21, respectively.

As per

Figure 11-6Figure 11-6, mean PR values generally increased in both the ALF-5755 group and the Placebo group within the ITT population, indicating an comparable improvement in PR in both groups.

Figure 11-6: PR: Mean Plot to Day 21 (ITT population)

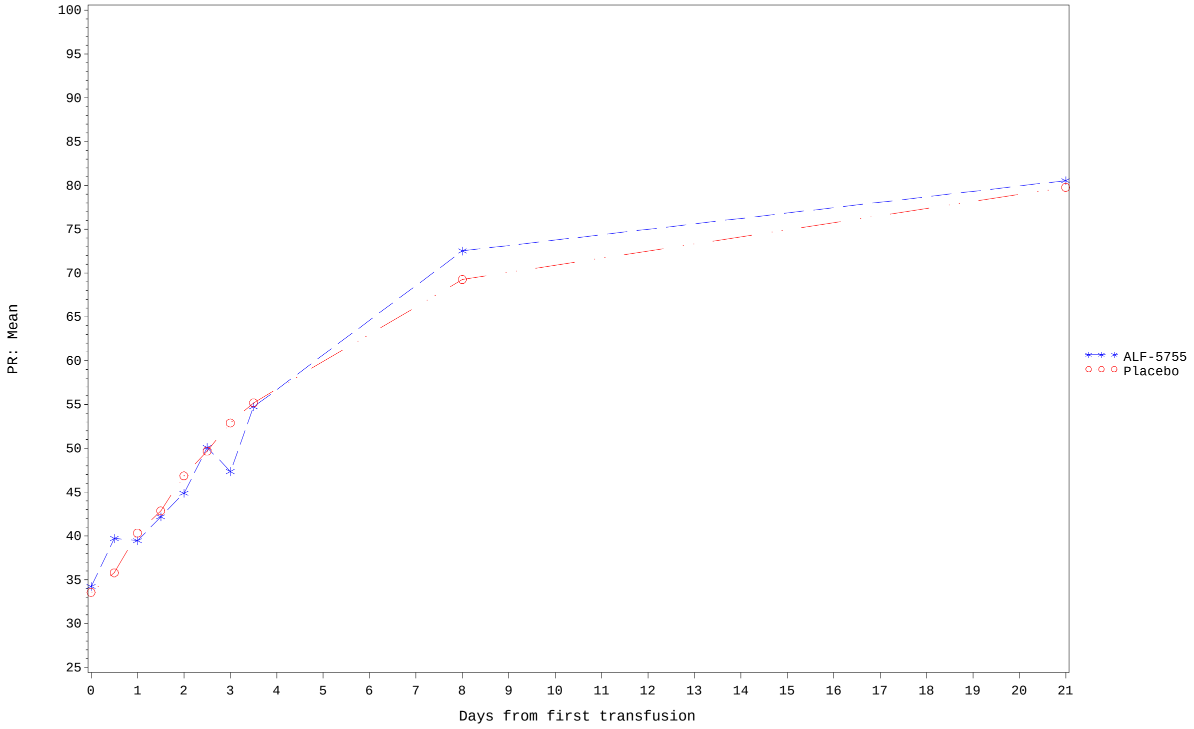

Source: Figure 7.1

As per Figure 11-7Figure 11-7, mean INR values showed decreases in both the ALF-5755 group and the Placebo group within the ITT population. Results were similar for the two groups, with a slightly higher decrease observed for the long-term results in the ALF-5755 group.

Figure 11-7: INR: Mean Plot to Day 21 (ITT population)

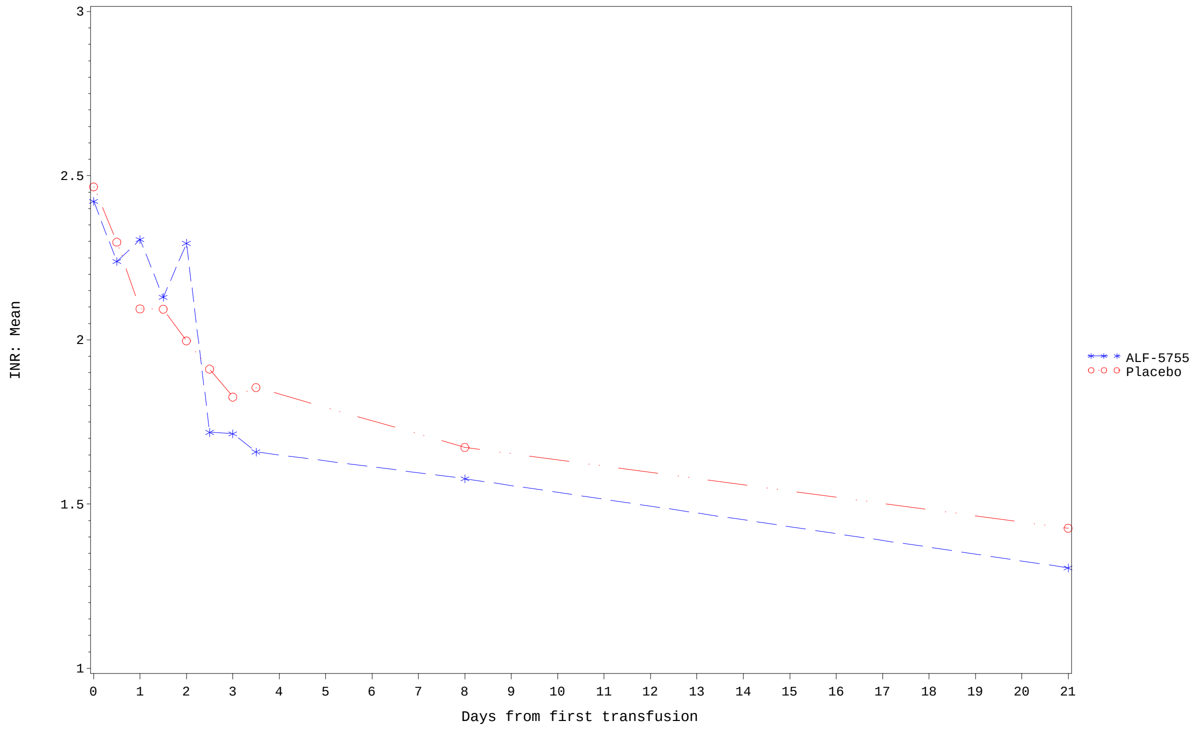

Source: Figure 7.2

11.4.1.2 HBV / AIH population

This subgroup comprised of 22 patients with HBV or AIH, 18 of whom received completed treatment (i.e., six infusions of treatment).

11.4.1.2.1 Primary Efficacy Analysis: Analysis of Prothrombin Ratio (PR) (0-72 hours)

Median values recorded in the analysis of PR (0-72 hours) in the ITT Population with HBV or AIH showed an increase in the primary endpoint for ALF-5755 versus placebo (0.028 versus -0.042). This difference was not significant at the 5% level for both the Wilcoxon and CMH tests (p=0.2696 and 0.4956, respectively).

Table 11-16: Analysis of PR (0-72 hours) (ITT Population with HBV or AIH)

		ALF-5755 (N=13)	Placebo (N=9)	p-value
Rate of change of PR	N	13	9	
	Mean (SD)	0.000 (0.118)	-0.032 (0.096)	
	Median	0.028	-0.042	
	Range	-0.25 - 0.14	-0.25 - 0.08	
Wilcoxon test				0.2696
CMH test				0.4956

Source: Table 14.2.1.1.1

Median values recorded in the analysis of PR (0-72 hours) in the ITT Population with HBV or AIH who received completed treatment showed an increase in the primary endpoint for ALF-5755 versus placebo (0.042 versus -0.042) that can be considered significant (p=0.0449) (refer to Table 11-17~~Table 11-17~~).

Table 11-17: Analysis of PR (0-72 hours) (ITT Population with HBV or AIH and who received 6 infusions of treatment)

		ALF-5755 (N=10)	Placebo (N=8)	p-value
Rate of change of PR	N	10	8	
	Mean (SD)	0.046 (0.067)	-0.040 (0.099)	
	Median	0.042	-0.042	
	Range	-0.08- 0.14	-0.25- 0.08	
Wilcoxon test				0.0449
CMH test adjusting for aetiology[1]				0.0474

Source: Table 14.2.1.1.20

SD: Standard deviation

11.4.1.2.2 Secondary Efficacy Analysis

11.4.1.2.2.1 *International Normalized Ratio (INR)*

Analysis of INR from 0 to 72 hours for the Completed Treatment population is presented in Table 14.2.2.14 for ITT patients with HBV and AIH and in Table 14.2.2.20 for Completed Treatment patients with HBV and AIH. INR Median Plot for the Completed Treatment Population with HBV or AIH is presented in Figure 5.2 (refer to Section 14) and INR

Median Plot for the Completed Treatment Population with HBV or AIH in Figure 5.4 (refer to Section 14).

A higher decrease of INR values is observed in the ALF-5755 group following infusion until Day 21 compared to the placebo group.

11.4.1.2.2.2 *Model for End Stage Liver Disease (MELD)*

Analysis of MELD from 0 to 72 hours is presented in Table 14.2.2.15 for ITT patients with HBV or AIH and in Table 14.2.2.21 for Completed Treatment patients with HBV or AIH. The rate of change of MELD (0-72 hours) is presented per patient in Listing 16.2.6.

A higher decrease of MELD values is observed in the ALF-5755 group following infusion until Day 21 compared to the placebo group.

11.4.1.2.2.3 *Total bilirubin, Conjugated bilirubin, Alanine Transaminase (ALT), Aspartate Transaminase (AST)*

The analysis of total bilirubin (0-72 hours) is presented in Table 14.2.2.19 for ITT patients with HBV or AIH and in Table 14.2.2.25 for Completed Treatment patients with HBV or AIH. The rate of change of total bilirubin (0-72 hours) is presented per patient in Listing 16.2.6.

The analysis of conjugated bilirubin (0-72 hours) is presented in Table 14.2.2.18 for ITT patients with HBV or AIH and in Table 14.2.2.24 for Completed Treatment patients with HBV or AIH. The rate of change of conjugated bilirubin (0-72 hours) is presented per patient in Listing 16.2.6.

The analysis of ALT from 0 to 72 hours is presented for ITT patients with HBV or AIH and in Table 14.2.2.22 for Completed Treatment patients with HBV or AIH. The rate of change of ALT (0-72 hours) is presented per patient in Listing 16.2.6.

The analysis of AST from 0 to 72 hours is presented in Table 14.2.2.17 for ITT patients with HBV or AIH and in Table 14.2.2.23 for Completed Treatment patients with HBV or AIH. The rate of change of AST (0-72 hours) is presented per patient in Listing 16.2.6.

Although differences between ALF-5755 treatment and placebo were noted in some instances, these were not considered significant in most instances and, in general, results for total bilirubin, conjugated bilirubin, ALT and AST do not provide any evidence of modification with ALF-5755 treatment.

11.4.1.2.2.4 *Analysis of Survival, Transplant-free Survival and Time to Transplantation*

A Kaplan-Meier plot of survival is illustrated in Figure 11-8Figure 11-8 (Listing 16.4.6.4/Figure 2.5 [refer to Section 14]). Survival data shows that 1 patient in the Placebo group died.

Figure 11-8: Kaplan-Meier Plot of Survival (ITT Population with HBV and AIH)

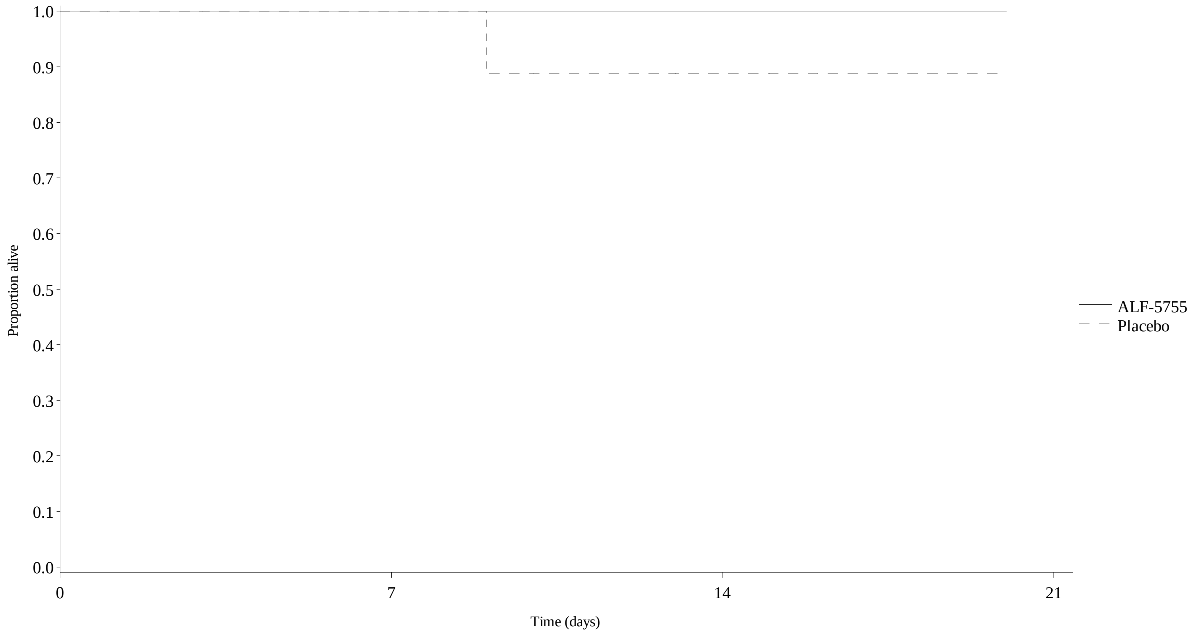

Source: Figure 2.5

For HBV or AIH patients, no liver transplants were required in patients treated with ALF-5755 group if GCglob>155 (0 of the 5 patients in the ALF-5755 group versus 1 of 4 patients in the placebo group).

Figure 11-9: Plot of Baseline GCGlobulin (ITT Population with HBV or AIH)

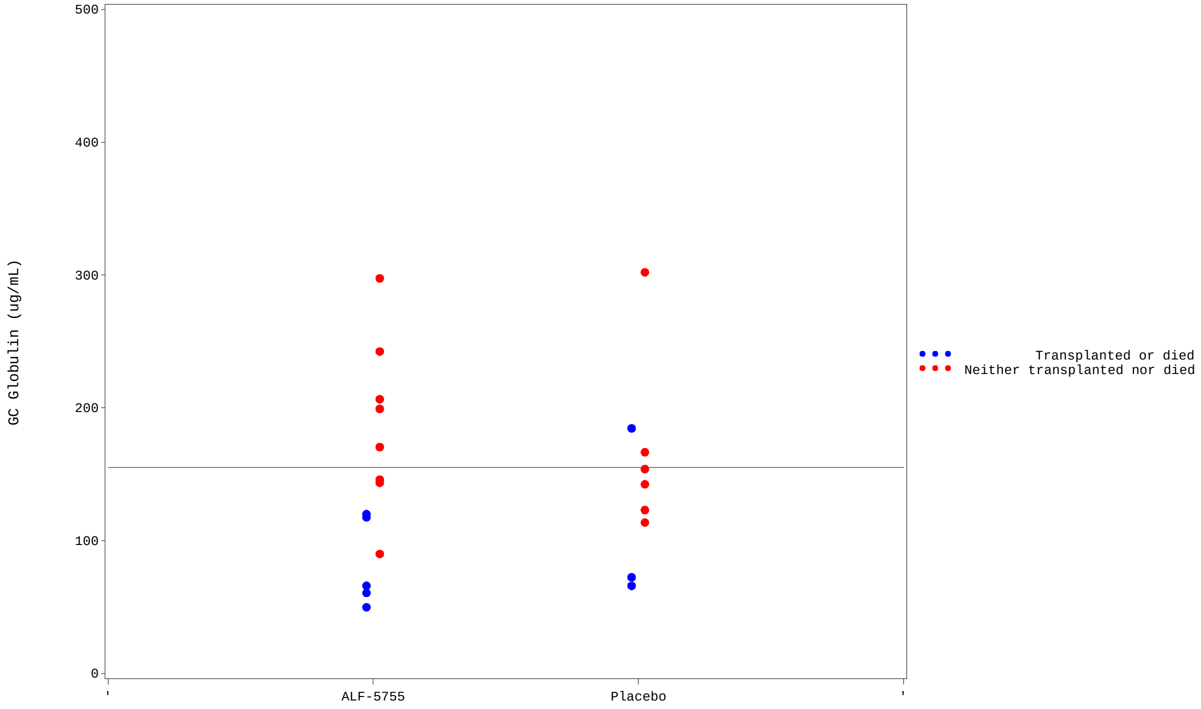

Source: Figure 6.2

In addition, stratification of patients according to total bilirubin showed similar results: that no liver transplants were required in patients treated with ALF-5755 group if Total Bilirubin<400.

Figure 11-10: Plot of Baseline Total Bilirubin (ITT Population with HBV or AIH)

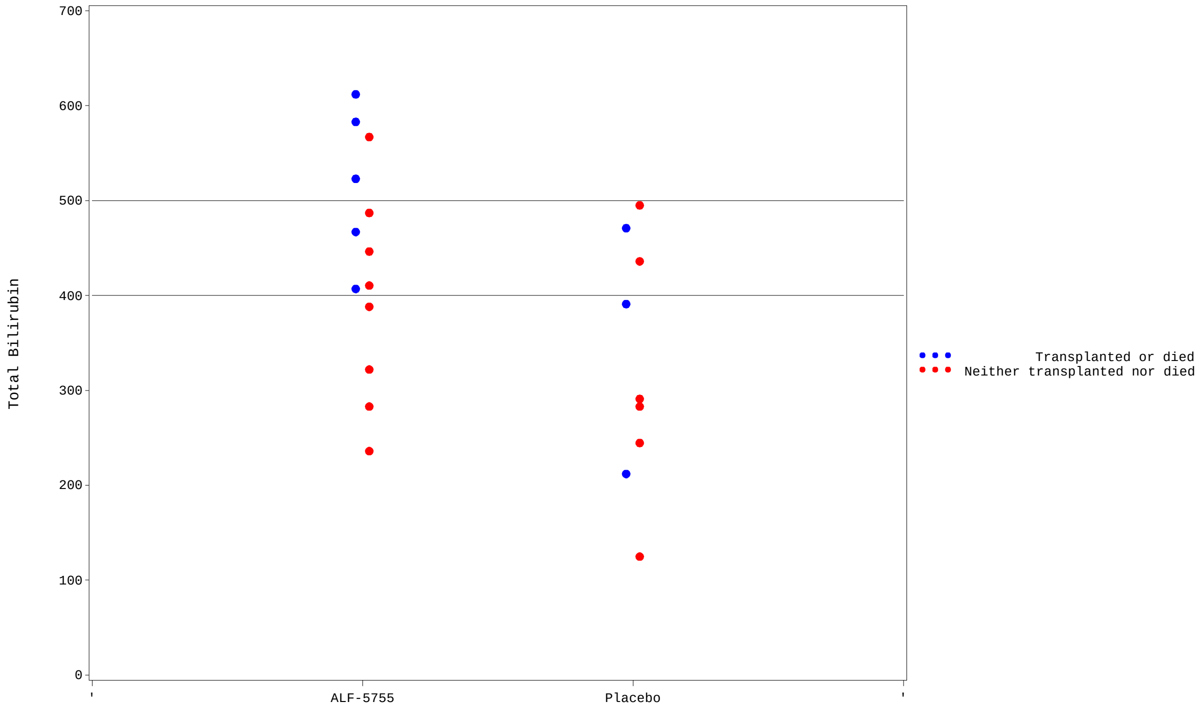

Source: Figure 6.1

Thus, stratification of patients according to Tbilirubin or GCglobulin showed that no liver transplants were required in patients treated with ALF-5755 group if TBili<400 and GCglob>155 when a rate of 33% of liver transplantation was observed in the placebo group.

A Kaplan-Meier plot of transplant-free survival is illustrated in Figure 11-11~~Figure 11-11~~ (Listings 16.4.6.2 and 16.4.6.4/Figure 2.6 [refer to Section 14]). No differences were observed between the ALF-5755 group and the placebo group.

Figure 11-11: Kaplan-Meier Plot of Transplant-Free Survival (ITT Population with HBV or AIH)

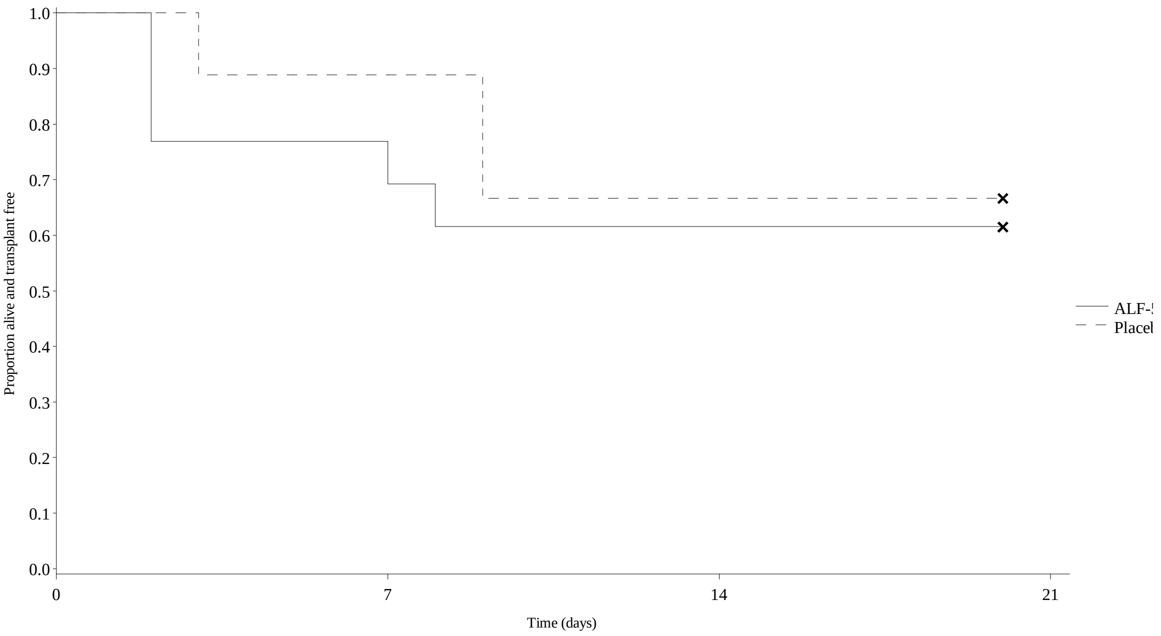

Number known to be alive and transplant free:

ALF-5755:	13	10		9	8
Placebo:	9		8		6

X indicates censored times

Source: Figure 2.6

A Kaplan-Meier plot of time to transplantation is illustrated in Listing 16.4.6.2 and Figure 2.7 (refer to Section 14). No differences were observed between the ALF-5755 group and the placebo group.

11.4.1.2.2.5 *Hospital-free days and length of hospitalization*

Analysis of hospital-free days is presented in (Table 14.2.5.5).

The median number of hospital-free days in the ALF-5755 group was 7, compared to 0 in the placebo group. This difference was not significant at the 5% level for the Wilcoxon test (p=0.116).

Table 11-18: Hospital-Free Days (ITT Population with HBV and AIH)

		ALF-5755 (N=13)	Placebo (N=9)	p-value
Hospital-free days up to D21[1]	N	13	9	
	Mean (SD)	6.77 (6.48)	2.56 (4.59)	
	Median	7.00	0.00	
	Range	0-16.0	0-13.0	
	Wilcoxon test			0.116

Source: Table 14.2.5.5

[1] Days alive following hospital discharge

Analysis of days in hospital is presented in Table 11-19 (Table 14.2.5.4) for ITT patients with HBV and AIH who were alive with no liver transplant.

In the ITT Population with HBV and AIH (alive with no liver transplant), the median number of days in the hospital was lower the ALF-5755 group (8.5 days) compared to the placebo group (19.5 days). The difference in days in hospital was significant at the 5% level for the Wilcoxon test (p=0.023) (refer to Table 11-19).

Table 11-19: Days in Hospital (ITT Population with HBV or AIH, Alive with No Liver Transplant)

		ALF-5755 (N=8)	Placebo (N=6)	p-value
Days in Hospital up to D21[1]	N	8	6	
	Mean (SD)	10 (4.34)	17.17 (5.27)	
	Median	8.5	19.5	
	Range	5 - 18	8 - 21	
	Wilcoxon test			0.023

Source: Table 14.2.5.4

[1] Up to and including date of hospital discharge, maximum of 21 days.

11.4.1.2.3 Complementary analyses

11.4.1.2.3.1 *Analysis of Prothrombin Ratio (PR) Early Efficacy: 0-12 hours*

Refer to Table 14.2.1.1.18 and Table 11-20~~Table 11-20~~ for analysis of PR (0-12 hours) for ITT population with HBV and AIH. Median values recorded showed a significant increase in the primary endpoint (0.167 versus -0.167 for placebo; p=0.0385).

ORION Clinical Services Ltd.

Table 11-20: Analysis of PR (0-12 hours) (ITT Population with HBV or AIH)

		ALF-5755 (N=13)	Placebo (N=9)	p-value
Rate of change of PR	N	12	9	
	Mean (SD)	0.177 (0.246)	-0.167 (0.400)	
	Median	0.167	-0.167	
	Range	-0.17- 0.67	-1.00- 0.25	
Wilcoxon test				0.0385
CMH test adjusting for aetiology[1]				0.0291

Source: Table 14.2.1.1.18

SD: Standard deviation

Median values recorded in the analysis of PR (0-12 hours) in the ITT Population with HBV or AIH and who received 6 infusions of treatment showed a significant increase in the primary endpoint (0.250 versus -0.208 for placebo; p=0.0295).

Table 11-21: Analysis of PR (0-12 hours) (ITT Population with HBV or AIH and who received 6 infusions of treatment)

		ALF-5755 (N=10)	Placebo (N=8)	p-value
Rate of change of PR	N	9	8	
	Mean (SD)	0.153 (0.219)	-0.198 (0.415)	
	Median	0.250	-0.208	
	Range	-0.17- 0.42	-1.00- 0.25	
Wilcoxon test				0.0295
CMH test adjusting for aetiology[1]				0.0469

Source: Table 14.2.1.1.26

SD: Standard deviation

11.4.1.2.3.2 *Analysis of Prothrombin Ratio (PR) Long Term Efficacy: 0-Day 8*

Median values recorded for the analysis of PR 0-Day 8 for patients in the ITT population with HBV or AIH showed an increase in ALF-5755 treated group (0.068) when compared to Placebo group (0.010), although of no significance.

Table 11-22: Analysis of PR (0-Day 8) (ITT Population with HBV or AIH)

		ALF-5755 (N=13)	Placebo (N=9)	p-value
Rate of change of PR	N	13	9	
	Mean (SD)	0.063 (0.154)	0.022 (0.075)	
	Median	0.068	0.010	
	Range	-0.25- 0.26	-0.07- 0.20	
Wilcoxon test				0.1088
CMH test adjusting for aetiology[1]				0.4601

Source: Table 14.2.1.1.28

SD: Standard deviation

In the ITT Population in patients with HBV or AIH and who received the complete treatment, median values recorded for the analysis of PR 0-Day 8 showed an increase in the ALF-5755 treated group (p=0.0067).

Table 11-23: Analysis of PR (0-Day 8) (ITT Population with HBV or AIH and who received 6 infusions of treatment)

		ALF-5755 (N=10)	Placebo (N=8)	p-value
Rate of change of PR	N	10	8	
	Mean (SD)	0.127 (0.086)	0.021 (0.081)	
	Median	0.099	0.010	
	Range	0.02- 0.26	-0.07- 0.20	
Wilcoxon test				0.0067
CMH test adjusting for aetiology[1]				0.0216

Source: Table 14.2.1.1.30

SD: Standard deviation

11.4.1.2.3.3 *Prothrombin Ratio (PR) and International Normalized Ratio (INR)*

Refer to Figure 11-12 and Figure 11-13 for the mean plot of PR and INR to Day 21 for the ITT population with HBV or AIH, respectively.

As per Figure 11-12, increases in mean PR values were consistently higher for the ALF-5755 group than the increases in the mean PR values of the placebo group. PR values were above

50% from Day 5 onwards for the ALF-5755 group, whereas mean PR values never reached 50% for the placebo group.

Figure 11-12: PR: Mean Plot to Day 21 (ITT population with HBV or AIH)

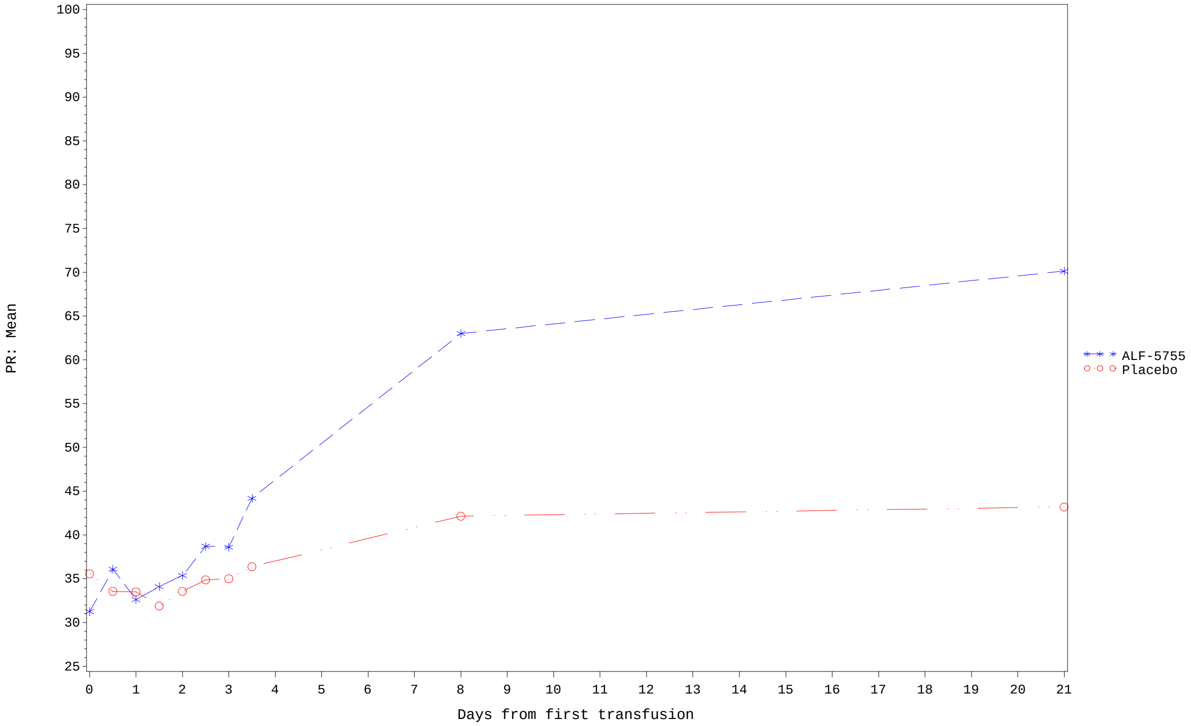

Source: Figure 7.3

As per Figure 11-13, decreases in mean INR values were consistently higher for the ALF-5755 group than the decreases in the mean INR values of the placebo group, with values below 2 from Day 2 onwards for the ALF-5755 group.

Figure 11-13: `INR: Mean Plot to Day 21 (ITT population with HBV or AIH)

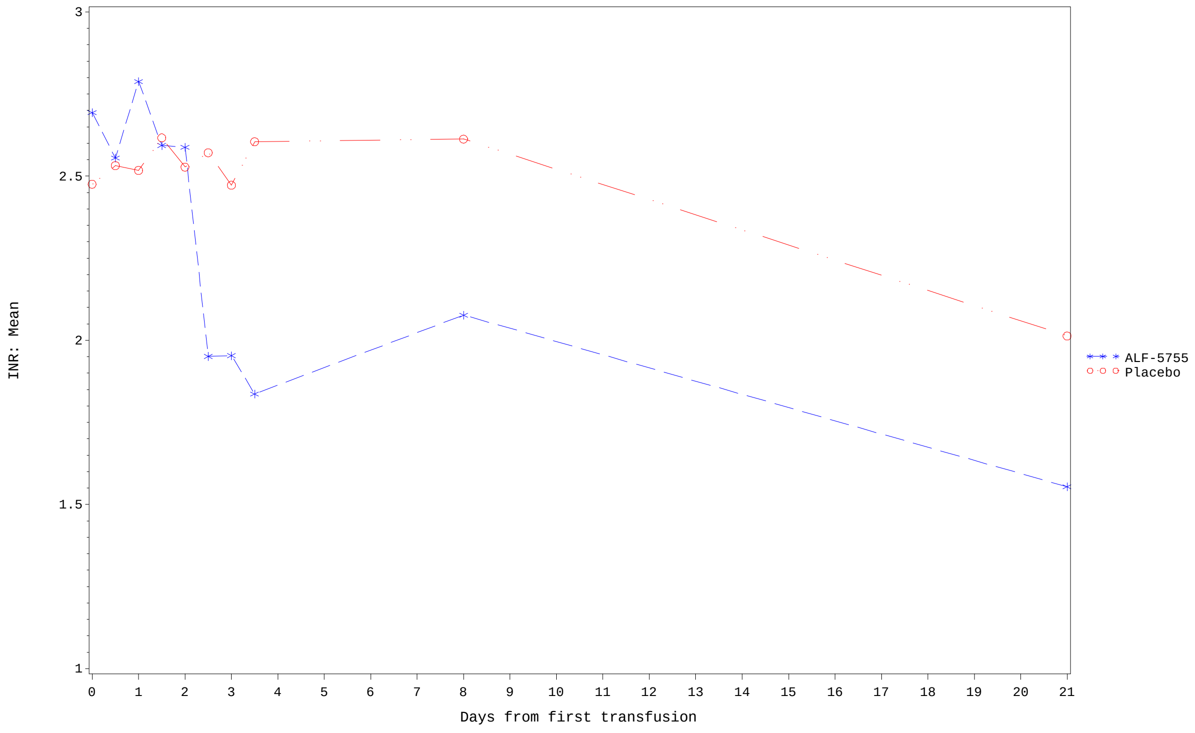

Source: Figure 7.4

11.4.1.2.3.4 ***Crossing time for PR>50% if significant***

Time to PR of 50% is presented in Table 11-24 for the ITT Population with HBV and AIH and listed per patient in Listing 16.4.6.1.

The median time in the ALF-5755 group is 8 days, whilst the median time for the placebo group was greater than the cut off time of 21 days. There was a significantly higher percentage of censored responses in the placebo group (85.7%) compared to the ALF-5755 group (38.5%) (p=0.0301).

The Kaplan-Meier plot of time to PR of 50% is illustrated in Figure 11-14.

ORION Clinical Services Ltd.

Table 11-24: Time to PR of 50% (ITT Population with HBV or AIH)

		ALF-5755 (N=13)	Placebo (N=9)	p-value
Kaplan-Meier estimate (days)	Upper quartile	20.0		
	95% CI	(8.0, 28.0)	(20.0,)	
	Median	8.0		
	95% CI	(7.0, 20.0)	(20.0,)	
	Lower quartile	7.0	20.0	
	95% CI	(0.5, 8.0)	(20.0,)	
	Log Rank Test			0.0301
	Number (%) of responses	8 (61.5%)	1 (14.3%)	
	Number (%) censored	5 (38.5%)	6 (85.7%)	
	Missing	0	2	

Source: Table 14.2.4.4

Footnotes: 2 patients in Placebo group excluded from analysis because baseline PR >50%

Calculations are based on actual times of assessments (not scheduled times).

Baseline value was used instead of H0 for patient 1205 since the H0 result was missing.

Patients who are transplanted censored at the last assessment for PR before the transplant.

SD: Standard deviation

Figure 11-14: Kaplan-Meier Plot of Time to Prothrombin Ratio (PR) of 50% (ITT Population with HBV and AIH)

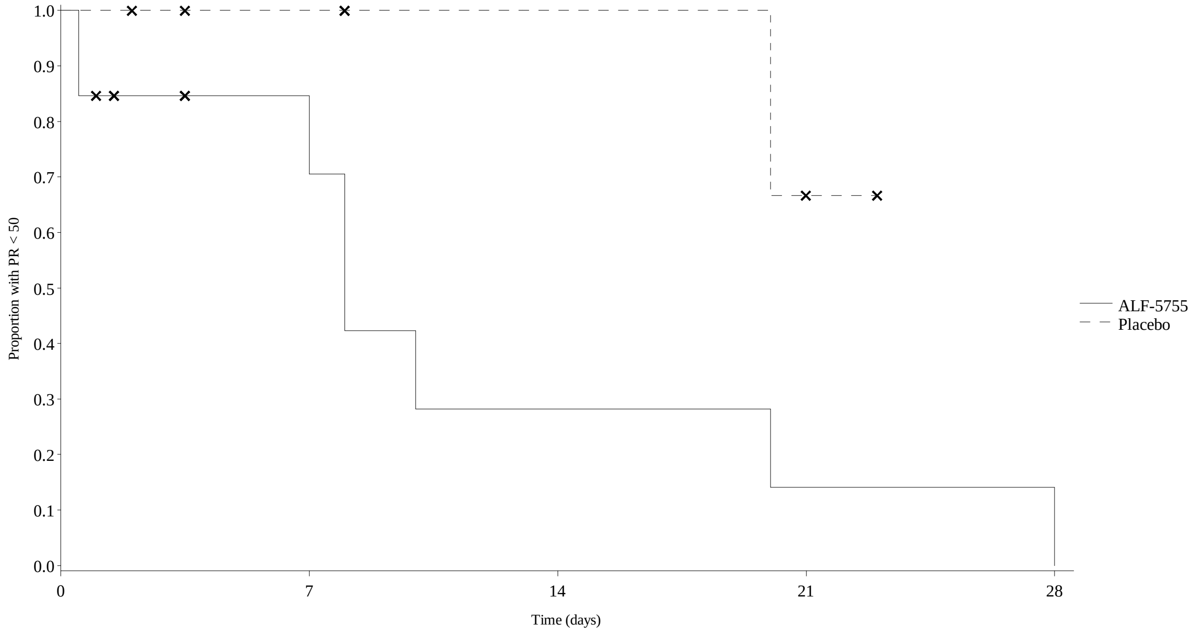

Number over 50%:

ALF-5755:	13	11	5	3	2	1	0
Placebo:	7					2	

X indicates censored times and may represent more than one patient
Programming note: Calculations are based on actual times of assessments (not scheduled times). Scale may need to extend beyond 21 days.
Baseline value was used instead of H0 for patient 1205 since the H0 result was missing.
Patients who are transplanted will be censored at the last assessment for PR before the transplant.

Source: Figure 2.4

11.4.1.3 Hepatic encephalopathy and Glasgow coma evaluation

Hepatic encephalopathy and Glasgow coma evaluation is presented in Listing 16.4.6.3 and survival status is presented in Listing 16.4.6.4.

11.4.2 Statistical/Analytical Issues

There were no statistical/analytical issues at the time of writing the SAP.

Additional analyses were planned and performed after database lock and unblinding and after reviewing the data. These must be interpreted in this light to reflect their exploratory nature to inform the planning of future studies.

No adjustment was made to p-values for multiple testing.

11.4.2.1 Adjustments for Covariates

None

11.4.2.2 Handling of Dropouts or Missing Data

Missing data was not imputed. If a baseline value was missing, no change from baseline was calculated. Baseline was pre-treatment and defined in the SAP (Appendix 16.1.1).

11.4.2.3 Interim Analyses and Data Monitoring

There was no interim analysis. Data listings were reviewed by DSMB. Refer to the DSMB meeting minutes included in Appendix 16.1.12.

11.4.2.4 Multicentre Studies

Sites were pooled for all analyses. No adjustment was made for centre effect or treatment by centre interaction.

11.4.2.5 Multiple Comparison/Multiplicity

No corrections were made to nominal p-values for multiple comparisons.

11.4.2.6 Use of an "Efficacy Subset" of Patients

Efficacy subsets include both ITT and PP populations. Please see Section 9.7.1.1 for the definitions of these analyses sets. Additional subsets were defined following database lock and unblinding for the complementary analyses.

11.4.2.7 Active-Control Studies Intended to Show Equivalence

Not applicable.

11.4.2.8 Examination of Subgroups

Subgroups are defined and described in the SAP (Appendix 16.1.1). Additional subgroups were defined following database lock or unblinding for the complementary analyses.

11.4.3 Tabulation of Individual Response Data

Individual response data are presented in Appendix 16.2.

11.4.4 Drug Dose, Drug Concentration, and Relationships to Response

Dose variation was not permitted. All 57 patients received at least one of the six planned doses (25 mL) of either ALF-5755 or placebo at the planned time points during the study (Listing 16.2.5).

11.4.4.1 Pharmacokinetics (PK)

Plasma concentration of ALF-5755 is presented over different time points, pre- and post-fusion in Table 11-25~~Table 11-25~~ (Table 14.2.7.1; Listing 16.4.7.1).

The concentrations of ALF-5755 following transfusion show the classic pattern of Absorption, Distribution and Elimination: The geometric mean plasma concentration of ALF-5755 rose sharply from 16.73 ng/mL H0 pre-infusion to a maximum of 2054.19 ng/mL at 10 minutes following infusion. It then fell to 568.74 ng/mL at H1 post-infusion, to 304.31 ng/mL at H2 post-infusion, to 80.94 ng/mL at H6 post-infusion and then further to 46.13 ng/mL at H12 post-infusion. By H48, the geometric mean plasma concentration of ALF-5755 was 43.46 ng/mL and rose slightly to 46.69 ng/mL at H60 pre-infusion, indicative of a small residual accumulation seen amongst infusions. However, this was still almost thrice the geometric mean plasma concentration of that at H0 pre-infusion. The geometric mean plasma concentration of ALF-5755 again rose sharply to a maximum of 2031.69 ng/mL at H60 + 10 minutes following infusion. It then fell to 50.50 ng/mL by H72 and then further to 33.82 ng/mL at H84, still more than double that at H0 and higher than at H60 pre-infusion.

Table 11-25: Plasma Concentration of ALF-5755 (PK Population)

		ALF-5755 (N=8)
H0 (pre-infusion)	N	8
	Arithmetic Mean	35.86 ng/mL
	Geometric Mean	16.73 ng/mL
	SD	58.93 ng/mL
	%CV	164.33
	Median	20.85 ng/mL
	Range	3.6 - 180.0 ng/mL
H0+10 mins (end of infusion)	N	8
	Arithmetic Mean	2144.00 ng/mL
	Geometric Mean	2054.19 ng/mL
	SD	695.59 ng/mL
	%CV	32.44
	Median	1992.00 ng/mL
	Range	1280.0 - 3552.0 ng/mL
H1	N	8
	Arithmetic Mean	595.88 ng/mL
	Geometric Mean	568.74 ng/mL
	SD	199.58 ng/mL
	%CV	33.49
	Median	528.00 ng/mL
	Range	405.0 - 920.0 ng/mL
H2	N	8
	Arithmetic Mean	355.75 ng/mL
	Geometric Mean	304.31 ng/mL
	SD	204.19 ng/mL
	%CV	57.40
	Median	309.50 ng/mL
	Range	135.0 – 640.0 ng/mL
H6	N	8
	Arithmetic Mean	111.45 ng/mL
	Geometric Mean	80.94 ng/mL
	SD	105.28 ng/mL
	%CV	94.46
	Median	60.00 ng/mL

ORION
Clinical Services Ltd.

		ALF-5755 (N=8)
	Range	30.0 – 324.0 ng/mL
H12	N	8
	Arithmetic Mean	97.53 ng/mL
	Geometric Mean	46.13 ng/mL
	SD	165.20 ng/mL
	%CV	169.40 ng/mL
	Median	28.70 ng/mL
	Range	15.6 - 500 ng/mL
H48	N	6
	Arithmetic Mean	52.67 ng/mL
	Geometric Mean	43.46 ng/mL
	SD	44.14 ng/mL
	%CV	83.81
	Median	37.60 ng/mL
	Range	25.6 - 142.0 ng/mL
H60 (pre-infusion)	N	6
	Arithmetic Mean	60.67 ng/mL
	Geometric Mean	46.69 ng/mL
	SD	56.87 ng/mL
	%CV	93.73
	Median	42.00 ng/mL
	Range	20.8 - 174.0 ng/mL
H60+10 mins (end of infusion)	N	6
	Arithmetic Mean	2168.00 ng/mL
	Geometric Mean	2031.69 ng/mL
	SD	777.08 ng/mL
	%CV	35.84
	Median	2276.00 ng/mL
	Range	1008.0 - 3000.0 ng/mL
H72	N	6
	Arithmetic Mean	73.80 ng/mL
	Geometric Mean	50.50 ng/mL
	SD	85.78 ng/mL
	%CV	116.24
	Median	37.20 ng/mL
	Range	24.4- 246.0 ng/mL

		ALF-5755 (N=8)
H84	N	6
	Arithmetic Mean	75.37 ng/mL
	Geometric Mean	33.82 ng/mL
	SD	130.72 ng/mL
	%CV	173.45
	Median	24.30 ng/mL
	Range	12.4 -342.0 ng/mL

Source: Table 14.2.7.1

PK parameters for ALF-5755 are presented over different time points, pre- and post-fusion in Table 11-26~~Table 11-26~~ (Table 14.2.7.2; Listing 16.4.7.2).

C_{max} was stable from the first to the sixth infusion (2144.00 ng/mL versus 2189.33 ng/mL). T_{max} increased from 10.00 minutes at the first infusion to 18.33 minutes at the sixth infusion. $T_{1/2}$ increased from 5.32 hours at the first infusion to 17.58 hours at the sixth infusion. AUC_{0-t} increased from 3085.83 ng/mL at the first infusion to 4443.46 ng/mL at the sixth infusion. AUC_{0-12} increased from 3085.83 ng/mL at the first infusion to 3563.59 ng/mL at the sixth infusion. The accumulation ratio was 1.45 ng/mL at the sixth visit.

Plasma parameters of ALF-5755 showed slight accumulation between the first and last infusions with slightly higher values of AUC_{0-12} following infusions at H60 compared to H0 (geometric mean 2709.63 ng/mL versus 3072.19 ng/mL, respectively). Steady state was achieved by H48.

Table 11-26: PK Parameters for ALF-5755 (PK Population)

		ALF-5755 (N=8)
C_{max} (ng/mL)		
1st infusion	N	8
	Arithmetic Mean	2144.00
	Geometric Mean	2054.19
	SD	695.59
	%CV	32.44
	Median	1992.00
	Range	1280.0 - 3552.0
6th infusion	N	6
	Arithmetic Mean	2189.33
	Geometric Mean	2047.15
	SD	797.80
	%CV	36.44
	Median	2276.00
	Range	1008.0 - 3000.0
$T_{1/2}$ (h)		
1st infusion	N	8
	Arithmetic Mean	5.32
	Geometric Mean	3.94
	SD	5.95
	%CV	111.95
	Median	3.21
	Range	2.4 - 19.9
6th infusion	N	6
	Arithmetic Mean	17.58
	Geometric Mean	14.79
	SD	13.07
	%CV	74.33
	Median	12.17
	Range	8.4 - 43.0

ORION Clinical Services Ltd.

		ALF-5755 (N=8)
AUC_{0-12} (ng/mL)		
1st infusion	N	8
	Arithmetic Mean	3085.83
	Geometric Mean	2709.63
	SD	1826.54
	%CV	59.19
	Median	2373.08
	Range	1567.3 - 6744.7
6th infusion	N	6
	Arithmetic Mean	3563.59
	Geometric Mean	3072.19
	SD	2206.30
	%CV	61.91
	Median	3019.23
	Range	1307.3 - 7597.3
Accumulation Ratio		
6th infusion	N	6
	Arithmetic Mean	1.52
	Geometric Mean	1.45
	SD	0.49
	%CV	31.99
	Median	1.51
	Range	0.8 - 2.3

Source: Table 14.2.7.2

Figure 11-15: Individual Patient PK Plots of ALF-5755 (PK Population): Patient 0101

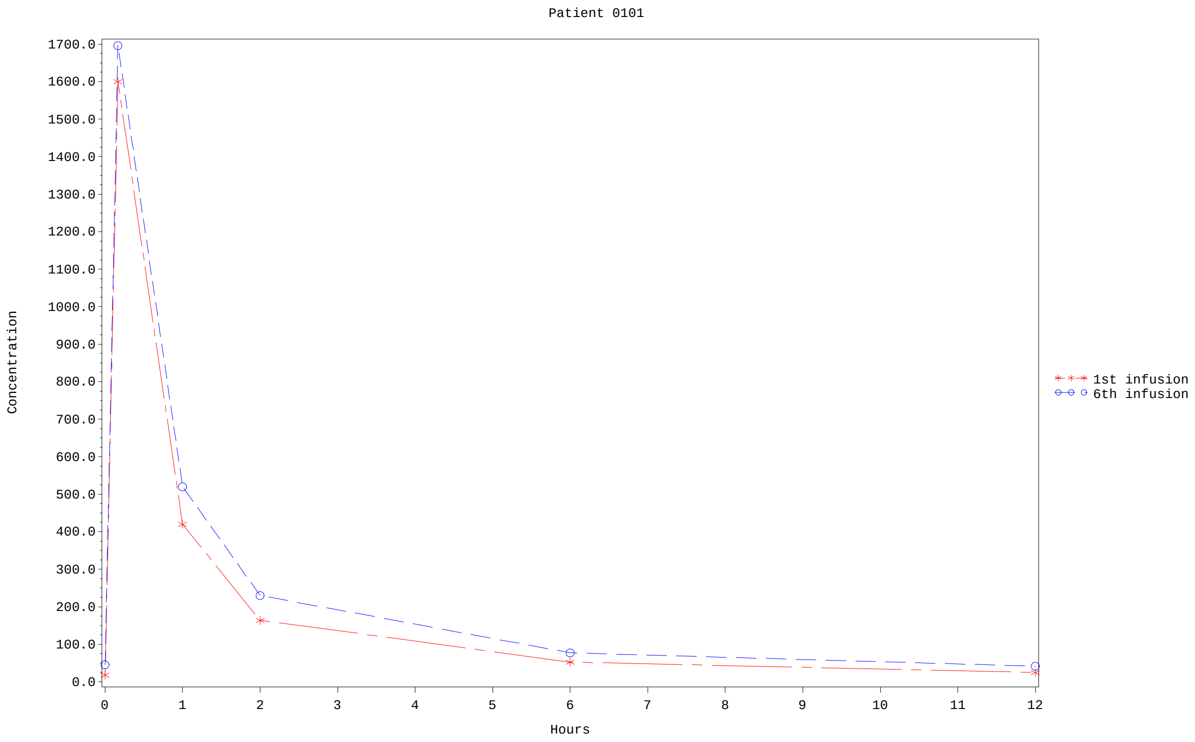

Source: Figure 3

Figure 11-16: Individual Patient PK Plots of ALF-5755 (PK Population): Patient 0102

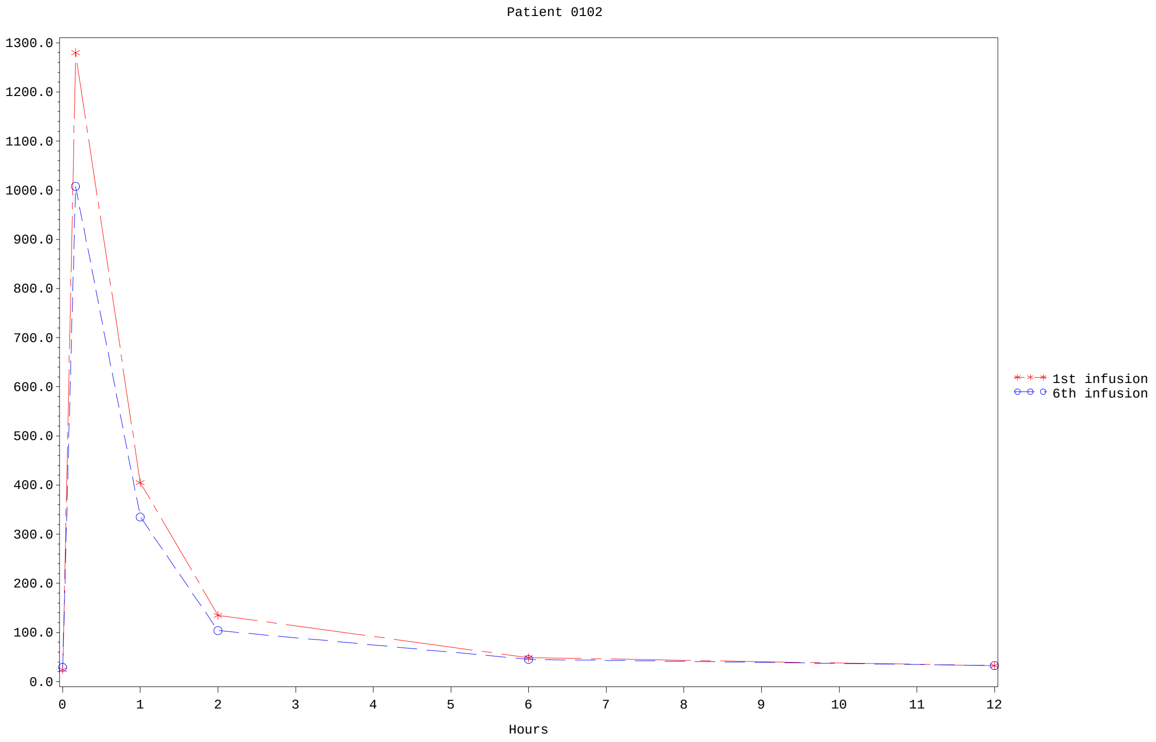

Source: Figure 3

Figure 11-17: Individual Patient PK Plots of ALF-5755 (PK Population): Patient 0108

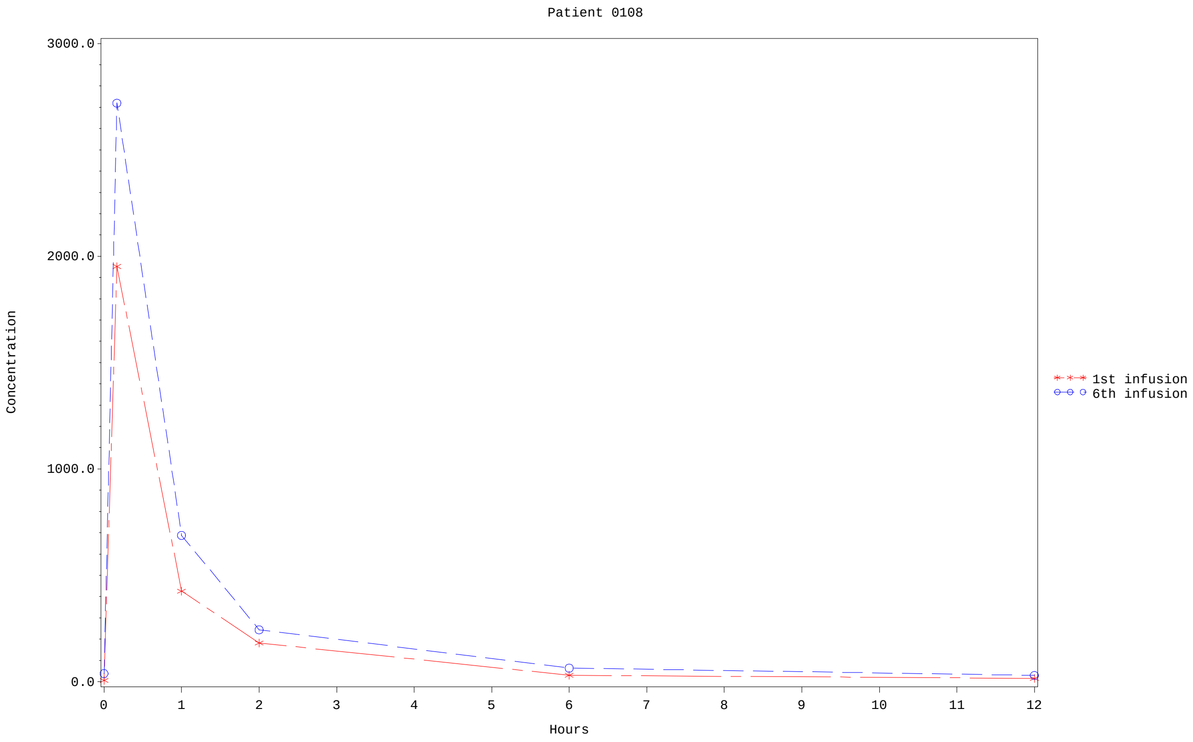

Source: Figure 3

Figure 11-18: Individual Patient PK Plots of ALF-5755 (PK Population): Patient 0112

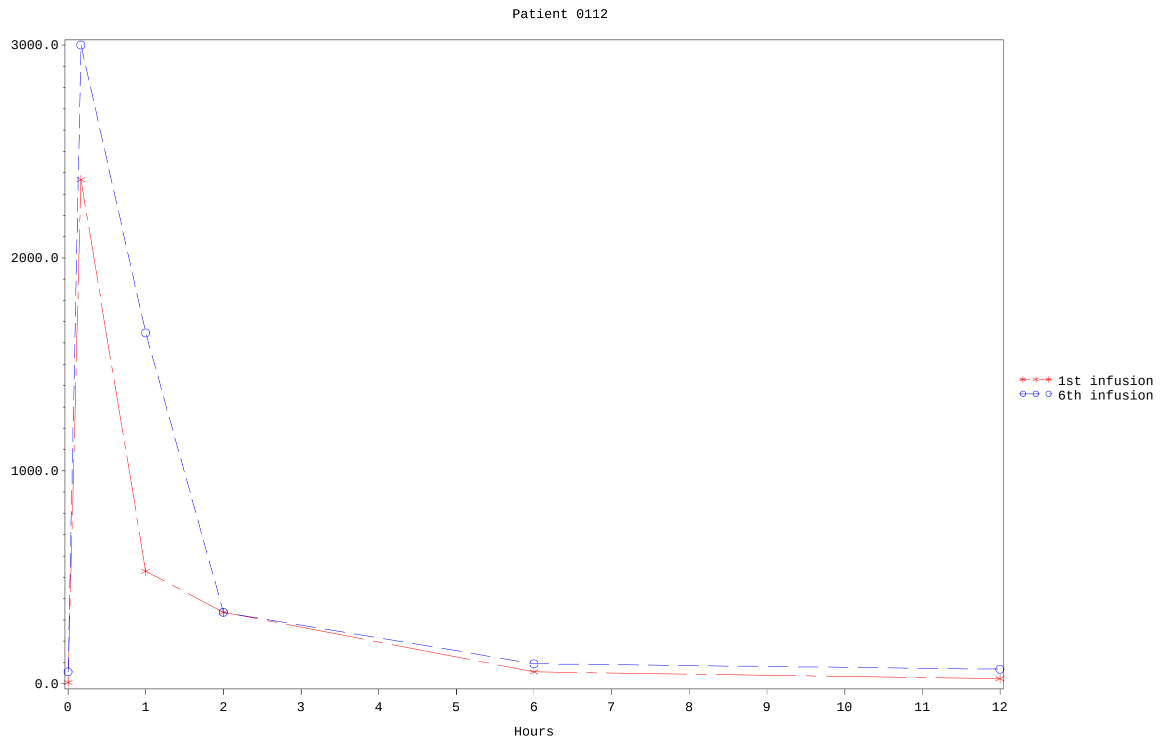

Source: Figure 3

Figure 11-19: Individual Patient PK Plots of ALF-5755 (PK Population): Patient 0113

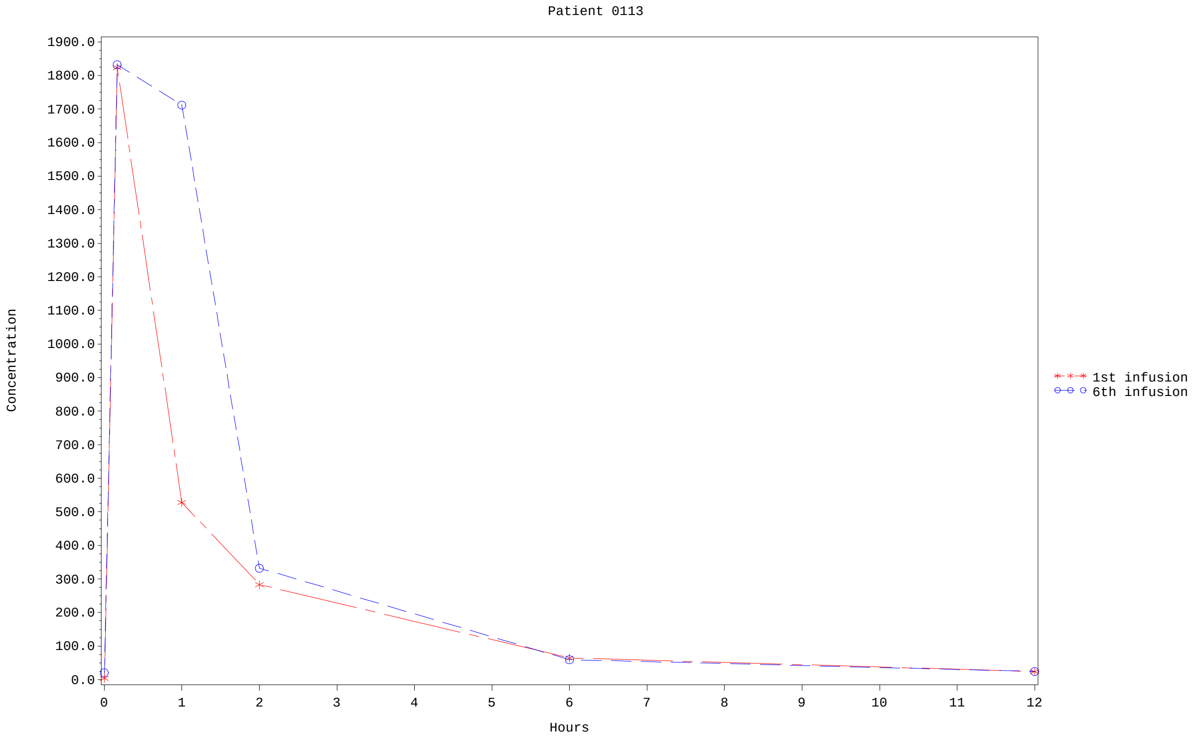

Source: Figure 3

Figure 11-20: Individual Patient PK Plots of ALF-5755 (PK Population): Patient 0114

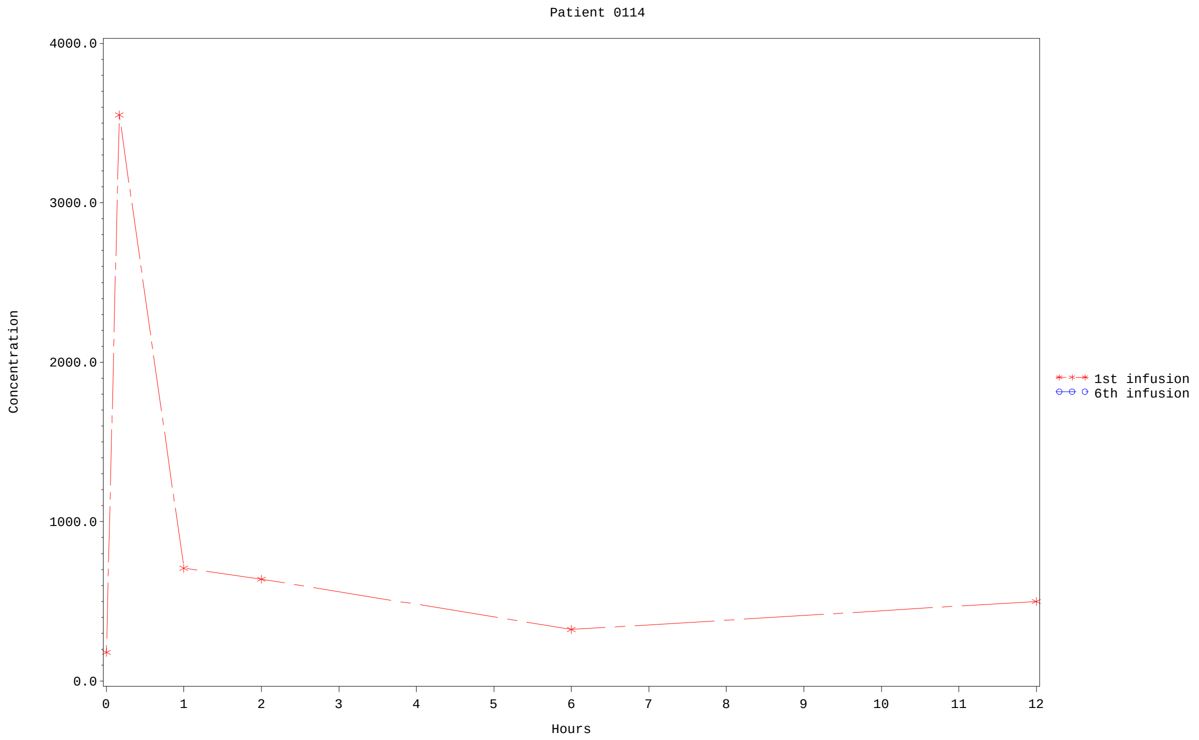

Source: Figure 3

Figure 11-21: Individual Patient PK Plots of ALF-5755 (PK Population): Patient 0201

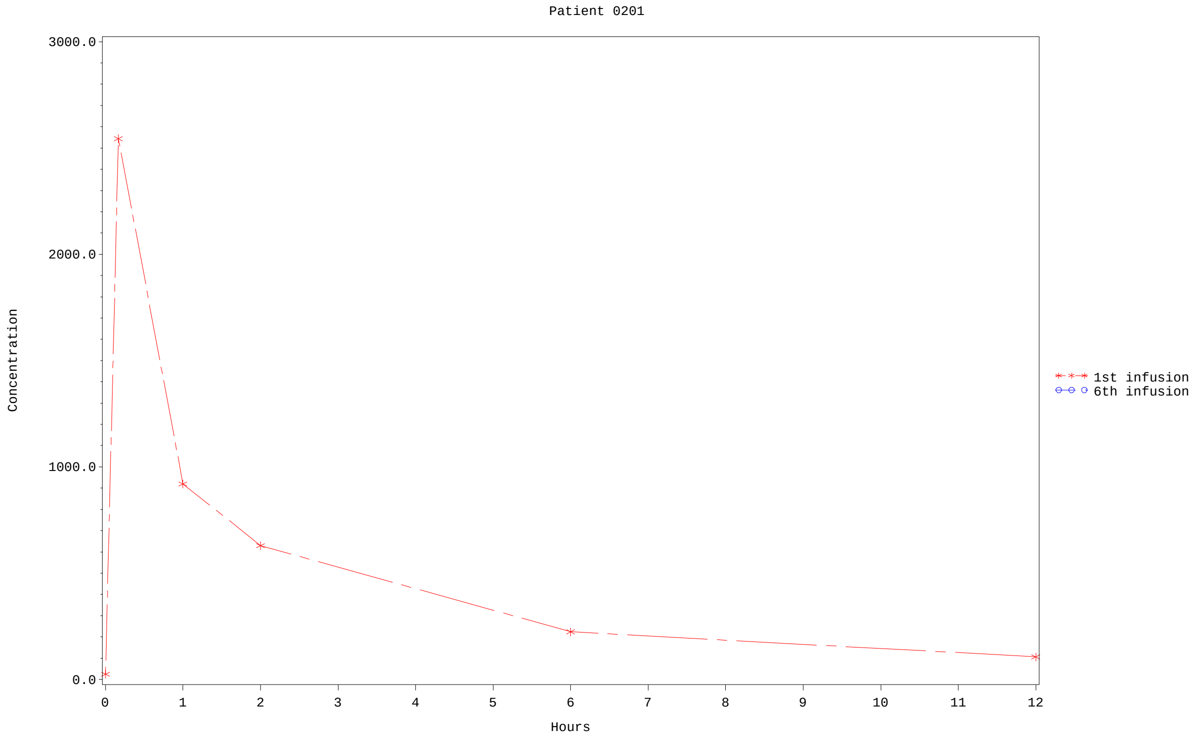

Source: Figure 3

Figure 11-22: Individual Patient PK Plots of ALF-5755 (PK Population): Patient 0204

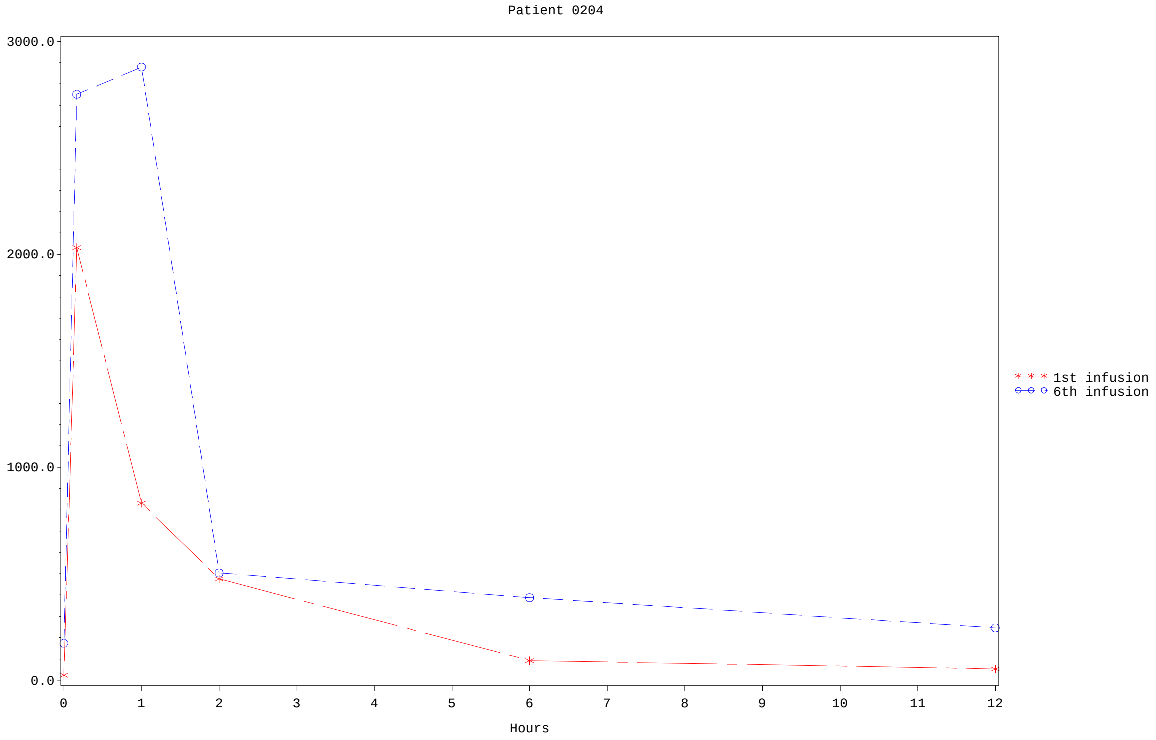

Source: Figure 3

Refer also to Listing 16.4.7.1.

11.4.5 Efficacy Conclusions

Additional subgroup analysis was requested following database lock, including patients who received 6 infusions of treatment.

Complementary analyses: After the unblinding of the study data, Alfact decided to have additional efficacy analyses. Variables included:

- Rate of change of PR from 0-12 h;
- Other time-points for efficacy markers (i.e., Day 8 and Day 21);
- Comparison of patient liver function status at inclusion according to population (ALF-5755 versus placebo in HBV AIH subgroup);
- Analysis of time to achieve PR of 50% for HBV AIH;
- Plots of total bilirubin and GCGlobulin at baseline.

ITT population:

Primary variables:

The analysis of PR from 0 to 72 hours showed no significant changes or obvious effect for the ITT population with complete treatment.

Secondary variables:

No significant changes or obvious effects were seen for patients in the ITT population treated with ALF-5755 versus placebo for MELD, INR, transplant-free survival and total and cleaved levels of CK18.

Median values recorded showed an increase in hospital-free days for ALF-5755 versus placebo for the ITT population, although of no significance.

For the ITT population (alive with no transplant), the mean number of days in the hospital was lower the ALF-5755 group (9.76 days) compared to the placebo group (12.64 days); however, the difference in days in hospital was not significant.

Complementary analyses:

Early efficacy: Median values recorded for analysis of PR 0-12 hours for the ITT Population showed an increase in ALF-5755 treated group (0.292) when compared to Placebo group (0.167), although of no significance due to large standard deviations.

GC globulin and bilirubin values at inclusion identified a group of patients for whom ALF-5755 avoid liver transplantation.

HBV/AIH subgroup:

Although results of the primary efficacy analysis (i.e., the rate of change of PR during 72 hours following treatment initiation) did not show any effects on the ITT population, results within the pre-defined HBV/AIH subgroup showed some benefit:

- The primary endpoint (i.e., the rate of change of PR during 72 hours following treatment initiation) was achieved for the population receiving the complete treatment (p=0.0449);
- Early efficacy (0-12 hours) for the analysis of PR was observed for ITT and complete treatment populations (p=0.0385 and p=0.0295, respectively);
- Long term efficacy (0-Day 8) for the analysis of PR was observed for the population receiving the complete treatment (p=0.0067);
- Amongst the transplant free patients, the mean number of days in the hospital was significantly lower in the ALF-5755 group (compared to the placebo group) (p=0.023).

The median time to PR of 50% for the ITT Population with HBV and AIH in the ALF-5755 group is 8 days, whilst the median time for the placebo group was greater than the cut off time of 21 days. There was a significantly higher percentage of censored responses in the placebo group (85.7%) compared to the ALF-5755 group (38.5%) (p=0.0301).

Liver transplant and transplant-free survival: Stratification of patients according to Tbilirubin or GCglobulin showed that no liver transplants were required in patients treated with ALF-5755 group if TBili<400 and GCglob>155 when a rate of 33% of liver transplantation was observed in the placebo group.

Pharmacokinetics (PK):

The plasma concentrations of ALF-5755 following infusions at H1 and H60 were all higher at H84 compared to baseline. The concentrations of ALF 5755 following transfusion, show the classic pattern of absorption, distribution and elimination.

Plasma concentrations of ALF-5755 following infusions at H1 and H60 were higher at H84 compared to baseline, and all PK parameters (t_{max}, $t_{1/2}$, AUC_{0-t}, and AUC_{0-12}) were higher by the sixth infusion compared to the first; C_{max} remained stable throughout. This increase has no specific meaning or consequences in terms of PK. Steady state was achieved by H48.

12 SAFETY EVALUATION

The Safety Population was defined as all patients who were randomised to treatment and received at least one administration of ALF-5755 or placebo (Section 9.7.1.1). Patients who received the wrong treatment were to be analysed as treated when using the Safety Population. A total of 57 patients were treated and included in the Safety Population, which was used for all safety analyses.

12.1 Extent of Exposure

Dose variation was not permitted. All 57 patients received at least one of the six planned doses (25 mL) of either ALF-5755 or placebo at the planned time points during the study (Listing 16.2.5).

12.2 Adverse Events

All AEs are presented in Listing 16.2.7.

12.2.1 Brief Summary of Adverse Events

A total of 262 TEAEs were reported in 55 of the 57 patients (Table 14.3.1.1). A total of five patients were reported with nine SAEs in the ALF-5755 group, and a total of seven patients in the placebo group were reported with 11 SAEs (Table 14.3.2.2). Two patients died during this study (Table 14.3.2.2): Two patients in the placebo group were reported with four AEs that led to withdrawal of treatment (Table 14.3.2.2).

12.2.2 Display of Adverse Events

Treatment-emergent adverse events by preferred term and system organ class (SOC) are summarised in Table 12-2~~Table 12-2~~ (Table 14.3.1.2).

The majority of patients experienced a TEAE in the system organ class metabolism and nutrition disorders; there were 71 events experienced by a total of 33 patients (57.9%). The most common preferred term in this system organ class was hypophosphataemia, experienced by 12 patients (21.1%). Treatment-emergent adverse events in the system organ class gastrointestinal disorders were also common, with 49 events experienced by 26 patients (45.6%), most commonly abdominal pain (11 events in 10 patients; 17.5%). There were events in the system organ class general disorders and administration site conditions experienced by 15 patients (26.3%), most commonly catheter site haemorrhage and peripheral oedema, each reported by 3 patients (5.3%).

12.2.3 Analysis of Adverse Events

Treatment-emergent adverse events are summarised in Table 12-1~~Table 12-1~~ (Table 14.3.1.1).

A total of 262 TEAEs were reported in 55 (96.5%) of the 57 patients. The number of TEAEs was balanced between the two treatment groups (133 TEAEs in 28 patients in the ALF-5755 group versus 129 TEAEs in 27 patients in the placebo group). Severe TEAEs were higher in the ALF-5755 group, however serious TEAEs were higher in the placebo group. The majority of TEAEs were mild or moderate in severity.

Table 12-1: Treatment-Emergent Adverse Events (Safety Population)

	ALF-5755 (N=28)	Placebo (N=29)	Total (N=57)
	**n N (%)**	**n N (%)**	**n N (%)**
Treatment Emergent Adverse Events	133 28 (100.0%)	129 27 (93.1%)	262 55 (96.5%)
Severe Treatment Emergent Adverse Events	27 10 (35.7%)	17 12 (41.4%)	44 22 (38.6%)
Serious Treatment Emergent Adverse Events	8 5 (17.9%)	11 7 (24.1%)	19 12 (21.1%)
Drug-Related Treatment Emergent Adverse Events	18 7 (25.0%)	9 7 (24.1%)	27 14 (24.6%)
Serious Drug-Related Treatment Emergent Adverse Events	2 2 (7.1%)	6 4 (13.8%)	8 6 (10.5%)
Treatment Emergent Adverse Events leading to withdrawal	0 0	4 2 (6.9%)	4 2 (3.5%)
Severity			
CTCAE grade 1 or Mild	86 25 (89.3%)	67 23 (79.3%)	153 48 (84.2%)
CTCAE grade 2 or Moderate	20 10 (35.7%)	45 18 (62.1%)	65 28 (49.1%)
CTCAE grade 3 or Severe	18 10 (35.7%)	13 11 (37.9%)	31 21 (36.8%)
CTCAE grade 4 or Life Threatening	8 3 (10.7%)	3 3 (10.3%)	11 6 (10.5%)
CTCAE grade 5 or Death	1 1 (3.6%)	1 1 (3.4%)	2 2 (3.5%)

Source: Table 14.3.1.1
n=number of events, N=number of subjects, %=percentage of subjects
Drug related is defined as relationship to study drug-related or missing

Treatment-emergent adverse events by preferred term are summarised in Table 12-2~~Table 12-2~~ (Table 14.3.1.2).

A total of 262 TEAEs were reported in 55 (96.5%) of the 57 patients. The proportion of TEAEs was balanced between both treatment groups. The majority of TEAEs were reported

for metabolism and nutrition disorders (57.9%), gastrointestinal disorders (45.6%), general disorders and administration site conditions (26.3%), nervous system disorders (21.1%), and blood and lymphatic system disorders (17.5%).

Table 12-2: Treatment-Emergent Adverse Events by Preferred Term (Safety Population)

	ALF-5755 (N=28)	Placebo (N=29)	Total (N=57)
	**n N (%)**	**n N (%)**	**n N (%)**
Any Adverse Event	133 28 (100.0%)	129 27 (93.1%)	262 55 (96.5%)
Metabolism and nutrition disorders	34 16 (57.1%)	37 17 (58.6%)	71 33 (57.9%)
Gastrointestinal disorders	28 16 (57.1%)	21 10 (34.5%)	49 26 (45.6%)
Blood and lymphatic system disorders	8 4 (14.3%)	16 6 (20.7%)	24 10 (17.5%)
Nervous system disorders	9 5 (17.9%)	8 7 (24.1%)	17 12 (21.1%)
General disorders and administration site conditions	7 6 (21.4%)	9 9 (31.0%)	16 15 (26.3%)
Investigations	10 4 (14.3%)	5 4 (13.8%)	15 8 (14.0%)
Infections and infestations	11 5 (17.9%)	3 3 (10.3%)	14 8 (14.0%)
Skin and subcutaneous tissue disorders	4 3 (10.7%)	6 5 (17.2%)	10 8 (14.0%)
Psychiatric disorders	3 3 (10.7%)	6 6 (20.7%)	9 9 (15.8%)
Renal and urinary disorders	4 4 (14.3%)	4 3 (10.3%)	8 7 (12.3%)
Cardiac disorders	4 4 (14.3%)	3 3 (10.3%)	7 7 (12.3%)
Vascular disorders	6 5 (17.9%)	1 1 (3.4%)	7 6 (10.5%)
Respiratory thoracic and mediastinal disorders	2 2 (7.1%)	3 3 (10.3%)	5 5 (8.8%)
Musculoskeletal and connective tissue disorders	1 1 (3.6%)	3 3 (10.3%)	4 4 (7.0%)

Source: Table 14.3.1.2
n=number of events, N=number of subjects, %=percentage of subjects

12.2.4 Listing of Adverse Events by Patient

Listing 16.2.7 provides a list of all AEs by patient.

12.3 Deaths, Other Serious Adverse Events, and Other Significant Adverse Events

12.3.1 Listing of Deaths, Other Serious Adverse Events, and Other Significant Adverse Events

12.3.1.1 Deaths

Two patients died during this study (Table 14.3.2.1):

- Patient 0204 in the ALF-5755 group died of multi-organ failure, and was experiencing acute respiratory distress syndrome at the time.
- Patient 0203 in the placebo group died of toxic shock syndrome.

12.3.1.2 Other Serious Adverse Events

Post-text table 14.3.2.2 provides the details of all SAEs reported during the study.

A total of five patients were reported with nine SAEs in the ALF-5755 group, and a total of seven patients in the placebo group were reported with 11 SAEs.

12.3.1.3 Other Significant Adverse Events

Two patients in the placebo group were reported with four AEs that led to withdrawal of treatment (Table 14.3.2.3):

- Patient 0202 was reported with life-threatening respiratory distress on Day 3. This SAE was rated as serious and deemed related to the study drug. Medication was provided and the placebo treatment was discontinued permanently. This patient recovered without sequelae on Day 13.
- Patient 0407 was reported with moderate paraesthesia on Day 1. This SAE was rated as serious and deemed related to the study drug. No treatment was provided and the placebo was discontinued permanently. The patient recovered without sequelae on the same day.

  This patient was also reported with moderate palpitations on Day 1. This SAE was rated as serious and deemed related to the study drug. No treatment was provided and the placebo was discontinued permanently. The patient recovered without sequelae on the same day.

  This patient was also reported with moderate hallucinations on Day 1. This SAE was rated as serious and deemed related to the study drug. No treatment was provided and

the placebo was discontinued permanently. The patient recovered without sequelae on the same day.

12.3.2 Narratives of Deaths, Other Serious Adverse Events, and Certain Other Significant Adverse Events

A total of five patients were reported with eight SAEs in the ALF-5755 group and were subsequently withdrawn from the study (Table 14.3.2.2):

- Patient 0108 was reported with mild bradycardia on Day 3. This SAE was rated as serious and deemed related to the study drug. No treatment was provided. The patient recovered on the same day without sequelae.

  The same patient was also reported with severe seizures and convulsions on Day 4. This SAE was rated as serious and deemed not related to study drug. Treatment was provided. The patient recovered without sequelae on Day 5.

- Patient 0114 was reported with life-threatening complications of a transplanted liver on Day 2. This SAE was reported as serious and deemed not related to the study drug. No treatment was provided. The patient recovered without sequelae on Day 3.

- Patient 0204 had acute respiratory distress, considered life-threatening, on Day 5. This SAE was rated as serious and deemed related to the study drug. Medication was provided and intubation was performed. This SAE was ongoing at the time of death.

  This patient was also reported with grade life-threatening post-LT arterial thrombosis on Day 8. This SAE was rated as serious and deemed not related to the study drug. Surgery was performed. The patient recovered without sequalae on Day 9.

  This patient was also reported with multi-organ failure on Day 6. This SAE was rated as serious and deemed not related to the study drug. No treatment was provided. The patient died on Day 10.

- Patient 0411 was reported with severe acute psychosis on Day 1. This SAE was rated as serious and deemed not related to the study drug. No treatment was provided and this SAE was ongoing.

- Patient 0702 was reported with moderate abdominal pains on Day 25. This SAE was rated as serious and deemed not related to the study drug. Medication was provided. The patient recovered without sequelae on Day 27.

A total of seven patients in the placebo group were reported with 11 SAEs (Table 14.3.2.2):

- Patient 0105 was reported with mild syncope on Day 4. This SAE was rated as serious and deemed not related to the study drug. No treatment was provided. The patient recovered without sequelae on Day 5.

  This patient was also reported with a moderate mediastinum neoplasm on Day 15. This SAE was rated as serious and deemed not related to the study drug. No treatment was provided. This SAE was ongoing.

  This patient was also reported with moderate nodal arrhythmia on Day 4. This SAE was rated as serious and deemed related to the study drug. No treatment was provided. The patient recovered without sequelae on the same day.

- Patient 0202 was reported with life-threatening respiratory distress on Day 3. This SAE was rated as serious and deemed related to the study drug. Medication was provided and the placebo treatment was discontinued permanently. This patient recovered without sequelae on Day 13.

- Patient 0203 was reported with septic shock on Day 8. This SAE was rated as serious and deemed not related to the study drug. Medication was provided. The patient died on Day 10.

- Patient 0306 was reported with life-threatening hepatic cirrhosis on Day 3. This SAE was rated as serious and deemed not related to the study drug. Surgery was performed. This SAE was ongoing.

- Patient 0405 was reported with severe renal failure on Day 3. This SAE was reported as serious and deemed not related to the study drug. Medication was provided and haemodialysis was performed. This SAE was ongoing.

- Patient 0407 was reported with moderate paraesthesia on Day 1. This SAE was rated as serious and related to the study drug. No treatment was provided and the placebo was discontinued permanently. The patient recovered without sequelae on the same day.

  This patient was also reported with moderate palpitations on Day 1. This SAE was rated as serious and deemed related to the study drug. No treatment was provided and the placebo was discontinued permanently. The patient recovered without sequelae on the same day.

This patient was also reported with moderate hallucinations on Day 1. This SAE was rated as serious and deemed related to the study drug. No treatment was provided and the placebo was discontinued permanently. The patient recovered without sequelae on the same day.

- Patient 1503 was reported with life-threatening agranulocytosis on Day 23. This SAE was rated as serious and deemed related to the study drug. Medication was provided. The patient recovered without sequelae on Day 25.

12.3.3 Analysis and Discussion of deaths, Other Serious Adverse Events and Other Significant Adverse Events

Two patients, Patient 0202 (ALF-5755) and Patient 0204 (placebo) (Section 12.3.2) were unblinded by the DSMB on 17Oct2011 due to life-threatening respiratory distress. It was decided that these patients were to continue in the study, with the recommendation that better evaluation of blood gas in the trial be implemented to clearly evidence the absence of respiratory safety concern.

12.4 Clinical Laboratory Evaluation

12.4.1 Listing of Individual Laboratory Measurements by Patient and Each Abnormal Laboratory Value

A listing of all individual laboratory measurements by patient is found in Appendix 16.2.8.

Abnormal laboratory values are provided in post-text tables 14.3.4.1 and 14.3.4.2.

12.4.2 Evaluation of each Laboratory Parameter

The following laboratory parameters were evaluated: haematology (haemoglobin, haematocrit, complete WBC count, RBC count platelet count) (Table 14.3.5.1); biochemistry (fibrinogen, alkaline phosphatase, GGT, total and conjugated bilirubin, ammonia, albumin, creatinine, urea, sodium, potassium, chloride, bicarbonates, lactates, calcium, magnesium, phosphorus, glucose, total proteins, amylase and lipase (Table 14.3.5.2).

12.4.2.1 Laboratory Values over Time

Listings 16.2.8.1.1, 16.2.8.1.2, 16.2.8.2.1, 16.2.8.2.2, 16.2.8.3, 16.2.8.4 and 16.2.8.5 provide summaries for all laboratory parameters over time. Values measured at each visit as well as changes from the end to the start of each treatment period as well as to the start of the first treatment period were summarized by treatment.

No difference between the two treatments was seen in any parameter, neither in average values at a visit nor with respect to average changes between the end and the start of the treatment period.

12.4.2.2 Individual Patient Changes

Listings 16.2.8.1.1 to 16.2.8.5 list all individual laboratory results per parameter for all patients who reported an abnormal value of this parameter at least once during the study, including the screening and the baseline value.

12.4.2.3 Individual Clinically Significant Abnormalities

Listings 16.2.8.1.1 to 16.2.8.5 list all individual laboratory results per parameter for all patients who reported an abnormal value of this parameter at least once during the study, including the screening and the baseline value.

12.5 Vital Signs, Physical Findings and Other Observations Related to Safety

12.5.1 Vital Signs

Listing 16.4.10 presents body temperature, systolic and diastolic blood pressure, heart rate, and respiratory rate. In addition the changes between the start and the end of the period are summarised for both treatments. Listing 16.4.14 presents blood pressure and heart rate monitoring.

12.5.2 Other Safety Evaluations

Listing 16.4.9 presents clinical examinations, Listing 16.4.11 presents the 12-lead ECG findings, Listing 16.4.12 presents arterial blood gas with lactates, and Listing 16.4.13 presents other sampling collection dates.

Listing 16.2.8.5 presents anti-ALF-5755 antibodies per patients. Anti-ALF5755 antibodies were analysed at Days 0, 8 and 21, but no antibodies were detected.

12.5.3 Special Safety Topics

There were no additional safety topics.

12.6 Safety Conclusions

A total of 262 TEAEs were reported in 55 of the 57 patients. The number of TEAEs was balanced between the two treatment groups. Severe TEAEs were higher in the ALF-5755

group, however serious TEAEs were higher in the placebo group. The majority of TEAEs were mild or moderate in severity. The majority of TEAEs were reported for metabolism and nutrition disorders, gastrointestinal disorders, general disorders and administration site conditions, nervous system disorders, and blood and lymphatic system disorders.

Two patients died during the study, one in the ALF-5755 group which was deemed related to the study drug, and one in the placebo group which was deemed not related to the study drug.

A total of five patients in the ALF-5755 group were reported with nine SAEs; two SAEs were deemed related to the study drug. A total of seven patients in the placebo group were reported with 11 SAEs; 5 SAEs were deemed related to the placebo. A total of five patients were reported with eight SAEs in the ALF-5755 group and were subsequently withdrawn from the study.

No difference between the two treatments was seen in any laboratory parameter, neither in average values at a visit nor with respect to average changes between the end and the start of the treatment period.

Anti-ALF5755 antibodies were analysed at Days 0, 8 and 21, but no antibodies were detected.

13 DISCUSSION AND OVERALL CONCLUSIONS

Acute liver failure is a life threatening disease with a multiple etiologies. From a previous retrospective study (Nalpas et al, 2011), the HBV/AIH subgroup was identified as the only homogenous sub-group of patients in term of clinical prognosis.

In this trial the benefit of ALF-5755 treatment was not demonstrated in the ITT population due to a very large heterogeneity of patients; however, the treatment partially improved the condition of all patients: the rate of change of PR during 12 hours following treatment initiation was doubled and hospitalization time was decreased (although not significant); stratification with biomarker also shows that ALF-5755 might avoid liver transplantation in some patients.

The positive results observed within the HBV/AIH subgroup confirm the hypothesis that ALF-5755 improves liver dysfunction during acute and acute on chronic liver diseases. This positive result obtained in the most severe subgroup of patients and with a small number of patients is a compelling result. Six (6) infusions of ALF-5755 allowed a significant recovery of patients as demonstrated by significant improvement of primary end-point, shortened time of hospitalization. Post-hoc analyses showed also a significant early and long term efficacy of ALF-5755. In addition, analysis of patients according to total bilirubin or GCglobulin at inclusion identified a group of patients receiving ALF-5755 who did not require liver transplantation.

ALF-5755 acts through an original MOA: an anchorage to extra-cellular matrix which is triggered by liver injury and inflammation, allowing ALF-5755 to exert its strong direct scavenger activity and reduce liver oxidative damages (Moniaux et al, 2010). As inflammation is a prominent feature of Acute on Chronic diseases, this MOA might account for the increased efficacy in this subgroup.

The positive results for patients suffering from HBV/AIH hepatitis need to be confirmed on a phase III trial or phase II on other acute-on-chronic hepatitis such as alcoholic hepatitis or end-stage liver diseases.

The plasma concentrations of ALF-5755 following infusions at H1 and H60 were all higher at H84 compared to baseline, as expected. The concentrations of ALF-5755 following transfusion show the classic pattern of absorption, distribution and elimination.

Plasma concentrations of ALF-5755 following infusions at H1 and H60 were higher at H84 compared to baseline, and all PK parameters (t_{max}, $t_{1/2}$, AUC_{0-t}, and AUC_{0-12}) were higher by the sixth infusion compared to the first; C_{max} remained stable throughout.

The number of TEAEs was balanced between the two treatment groups. Severe TEAEs were higher in the ALF-5755 group, however serious TEAEs were higher in the placebo group. The majority of TEAEs were mild or moderate in severity. Two patients died during the study, one in the ALF-5755 group which was deemed related to the study drug, and one in the placebo group which was deemed not related to the study drug.

The number of SAEs and the number of patients who experienced SAEs were higher in the placebo group, indicating that ALF-5755 had an equal if not a slightly better safety profile compared to placebo.

14 TABLES, FIGURES, AND GRAPHS REFERRED TO BUT NOT INCLUDED IN THE TEXT

14.1 Demographic Data

Table 14.1.1	Patient Disposition	All Available Subjects
Table 14.1.2	Study Termination and Primary Reason for Withdrawal	All Randomised Patients
Table 14.1.3.1	Demographics and Baseline Characteristics	ITT Population
Table 14.1.3.2	Baseline Factors	ITT Population
Table 14.1.4.1	*Medical History*	*ITT Population*
Table 14.1.4.2	*Current Medical Conditions*	*ITT Population*
Table 14.1.5.1	*Prior Medications*	*ITT Population*
Table 14.1.5.2	*Concomitant Medications*	*ITT Population*
Table 14.1.5.3	*Changes in Concomitant Medications*	*ITT Population*
Table 14.1.5.4	*Concomitant Procedures and Surgery*	*ITT Population*
Table 14.1.6	*Study Drug Exposure*	*Safety Population*

14.2 Efficacy Data

Table 14.2.1.1	Analysis of PR (0-72 hours)	ITT Population
Table 14.2.1.2	Analysis of PR (0-72 hours)	PP Population
Table 14.2.2.1	*Analysis of FV (0-72 hours)*	*ITT Population*
Table 14.2.2.2	Analysis of INR (0-72 hours)	ITT Population
Table 14.2.2.3	Analysis of MELD (0-72 hours)	ITT Population
Table 14.2.2.4	Analysis of ALT (0-72 hours)	ITT Population
Table 14.2.2.5	Analysis of AST (0-72 hours)	ITT Population
Table 14.2.2.6	Analysis of Conjugated Bilirubin (0-72 hours)	ITT Population
Table 14.2.2.7	Analysis of Total Bilirubin (0-72 hours)	ITT Population
Table 14.2.2.8	Analysis of INR (0-72 hours)	Completed Treatment Population
Table 14.2.2.9	Analysis of MELD (0-72 hours)	Completed Treatment Population
Table 14.2.2.10	Analysis of ALT (0-72 hours)	Completed Treatment Population
Table 14.2.2.11	Analysis of AST (0-72 hours)	Completed Treatment Population
Table 14.2.2.12	Analysis of Conjugated Bilirubin (0-72 hours)	Completed Treatment Population
Table 14.2.2.13	Analysis of Total Bilirubin (0-72 hours)	Completed Treatment Population
Table 14.2.3.1	*Change in HE Grade*	*ITT Population*

Table 14.2.3.2	*HE Grade*	*ITT Population*
Table 14.2.4.1	*Overall Survival*	*ITT Population*
Table 14.2.4.2	Transplant-free Survival	ITT Population
Table 14.2.4.3	Time to Transplantation	ITT Population
Table 14.2.5.1	Hospital-free Days	ITT Population
Table 14.2.5.2	Days in Hospital	ITT Population, Alive with No Liver Transplant
Table 14.2.5.3	Days in Hospital	Completed Treatment Population, Alive with No Liver Transplant
Table 14.2.6.1	Total CK18	ITT Population
Table 14.2.6.2	Cleaved CK18	ITT Population
Table 14.2.6.3	*Biomarkers*	*ITT Population*
Figure 1.1	PR: Median Change from Baseline	ITT Population
Figure 1.3	INR: Median Change from Baseline	ITT Population
Figure 1.4	MELD: Median Change from Baseline	ITT Population
Figure 2.1	Kaplan-Meier Plot of Survival	ITT Population
Figure 2.2	Kaplan-Meier Plot of Transplant-Free Survival	ITT Population
Figure 2.3	Kaplan-Meier Plot of Time to Transplantation	ITT Population
Figure 3	Individual Patient PK Plots of ALF-5755 Concentration	PK Population
Figure 4	Mean Plots of Laboratory Parameters	ITT Population
Figure 5.1	PR: Median Plot	Completer Population
Figure 5.3	INR: Median Plot	Completer Population
Figure 6.3	Plot of Baseline Total Bilirubin	ITT Population
Figure 6.4	Plot of Baseline GCGlobulin	ITT Population
Figure 7.1	PR: Mean Plot to Day 21	ITT Population
Figure 7.2	INR: Mean Plot to Day 21	ITT Population

14.2.1 Subgroup Analyses

Table 14.2.1.1.1	Analysis of PR (0-72 hours)	ITT Population with Viral Hepatitis B or Autoimmune Hepatitis
Table 14.2.1.1.2	Analysis of PR (0-72 hours)	ITT Population with Viral Hepatitis E, Other Etiology, Undetermined Etiology or Drug-induced Etiology
Table 14.2.1.1.3	*Analysis of PR (0-72 hours)*	*ITT Population with Viral Hepatitis A*
Table 14.2.1.1.4	*Analysis of PR (0-72 hours)*	*ITT Population with Viral Hepatitis B*
Table 14.2.1.1.5	*Analysis of PR (0-72 hours)*	*ITT Population with Viral Hepatitis E*
Table 14.2.1.1.6	*Analysis of PR (0-72 hours)*	*ITT Population with Autoimmune Hepatitis*

Table 14.2.1.1.7	*Analysis of PR (0-72 hours)*	*ITT Population with Other Etiology (including Viral Hepatitis E)*
Table 14.2.1.1.8	*Analysis of PR (0-72 hours)*	*ITT Population with Other Etiology (excluding Viral Hepatitis E)*
Table 14.2.1.1.9	*Analysis of PR (0-72 hours)*	*ITT Population with Undetermined Etiology*
Table 14.2.1.1.10	*Analysis of PR (0-72 hours)*	*ITT Population with Drug-induced Etiology*
Table 14.2.1.1.11	*Analysis of PR (0-72 hours)*	*ITT Population with Initial PR* $\geq$ *the median*
Table 14.2.1.1.12	*Analysis of PR (0-72 hours)*	*ITT Population with Initial PR < the median*
Table 14.2.1.1.13	*Analysis of PR (0-72 hours)*	*ITT Population with HE Grade 0*
Table 14.2.1.1.14	*Analysis of PR (0-72 hours)*	*ITT Population with HE Grade I or II*
Table 14.2.1.1.15	*Analysis of PR (0-72 hours)*	*ITT Population with NAC*
Table 14.2.1.1.16	*Analysis of PR (0-72 hours)*	*ITT Population without NAC*
Table 14.2.1.1.17	Analysis of PR (0-72 hours)	ITT Population with Viral Hepatitis A, Viral Hepatitis E, Other Etiology, Undetermined Etiology or Drug-induced Etiology
Table 14.2.1.1.18	Analysis of PR (0-12 hours)	ITT Population with Viral Hepatitis B or Autoimmune Hepatitis
Table 14.2.1.1.19	Analysis of PR (0-12 hours)	ITT Population with Viral Hepatitis A, Viral Hepatitis E, Other Etiology, Undetermined Etiology or Drug-induced Etiology
Table 14.2.1.1.20	Analysis of PR (0-72 hours)	ITT Population with Viral Hepatitis B or Autoimmune Hepatitis and who received 6 infusions of treatment
Table 14.2.1.1.21	Analysis of PR (0-72 hours)	ITT Population with Viral Hepatitis A, Viral Hepatitis E, Other Etiology, Undetermined Etiology or Drug-induced Etiology and who received 6 infusions of treatment
Table 14.2.1.1.22	Analysis of PR (0-72 hours)	ITT Population who received 6 infusions of treatment
Table 14.2.1.1.23	Analysis of PR (0-12 hours)	ITT Population
Table 14.2.1.1.24	Summary of Rate of Change of PR (0-72 hours) by Etiology Subgroups	ITT Population
Table 14.2.1.1.25	Analysis of PR (0-12 hours)	ITT Population who received 6 infusions of treatment
Table 14.2.1.1.26	Analysis of PR (0-12 hours)	ITT Population with Viral Hepatitis B or Autoimmune Hepatitis and who received 6 infusions of treatment
Table 14.2.1.1.27	Analysis of PR (0-Day 8)	ITT Population

Table 14.2.1.1.28	Analysis of PR (0-Day 8)	ITT Population with Viral Hepatitis B or Autoimmune Hepatitis
Table 14.2.1.1.29	Analysis of PR (0-Day 8)	ITT Population who received 6 infusions of treatment
Table 14.2.1.1.30	Analysis of PR (0-Day 8)	ITT Population with Viral Hepatitis B or Autoimmune Hepatitis and who received 6 infusions of treatment
Table 14.2.2.14	Analysis of INR (0-72 hours)	ITT Population with Viral Hepatitis B or Autoimmune Hepatitis
Table 14.2.2.15	Analysis of MELD (0-72 hours)	ITT Population with Viral Hepatitis B or Autoimmune Hepatitis
Table 14.2.2.16	Analysis of ALT (0-72 hours)	ITT Population with Viral Hepatitis B or Autoimmune Hepatitis
Table 14.2.2.17	Analysis of AST (0-72 hours)	ITT Population with Viral Hepatitis B or Autoimmune Hepatitis
Table 14.2.2.18	Analysis of Conjugated Bilirubin (0-72 hours)	ITT Population with Viral Hepatitis B or Autoimmune Hepatitis
Table 14.2.2.19	Analysis of Total Bilirubin (0-72 hours)	ITT Population with Viral Hepatitis B or Autoimmune Hepatitis
Table 14.2.2.20	Analysis of INR (0-72 hours)	Completed Treatment Population with Viral Hepatitis B or Autoimmune Hepatitis
Table 14.2.2.21	Analysis of MELD (0-72 hours)	Completed Treatment Population with Viral Hepatitis B or Autoimmune Hepatitis
Table 14.2.2.22	Analysis of ALT (0-72 hours)	Completed Treatment Population with Viral Hepatitis B or Autoimmune Hepatitis
Table 14.2.2.23	Analysis of AST (0-72 hours)	Completed Treatment Population with Viral Hepatitis B or Autoimmune Hepatitis
Table 14.2.2.24	Analysis of Conjugated Bilirubin (0-72 hours)	Completed Treatment Population with Viral Hepatitis B or Autoimmune Hepatitis
Table 14.2.2.25	Analysis of Total Bilirubin (0-72 hours)	Completed Treatment Population with Viral Hepatitis B or Autoimmune Hepatitis
Table 14.2.4.4	Time to PR of 50%	ITT Population with Viral Hepatitis B or Autoimmune Hepatitis
Table 14.2.5.4	Days in Hospital	ITT Population with Viral Hepatitis B or Autoimmune Hepatitis , Alive with No Liver Transplant
Table 14.2.5.5	Hospital-free Days	ITT Population with Viral Hepatitis B or Autoimmune Hepatitis
Figure 2.4	Kaplan-Meier Plot of Time to PR of 50%	ITT Population with Viral Hepatitis B or Autoimmune Hepatitis
Figure 2.5	Kaplan-Meier Plot of Survival	ITT Population with Viral Hepatitis B or Autoimmune Hepatitis
Figure 2.6	Kaplan-Meier Plot of Transplant-Free Survival	ITT Population with Viral Hepatitis B or

ORION Clinical Services Ltd.

		Autoimmune Hepatitis
Figure 2.7	Kaplan-Meier Plot of Time to Transplantation	ITT Population with Viral Hepatitis B or Autoimmune Hepatitis
Figure 5.2	PR: Median Plot	Completer Population with Viral Hepatitis B or Autoimmune Hepatitis
Figure 5.4	INR: Median Plot	Completer Population with Viral Hepatitis B or Autoimmune Hepatitis
Figure 6.1	Plot of Baseline Total Bilirubin	ITT Population with Viral Hepatitis B or Autoimmune Hepatitis
Figure 6.2	Plot of Baseline GCGlobulin	ITT Population with Viral Hepatitis B or Autoimmune Hepatitis
Figure 7.3	PR: Mean Plot to Day 21	ITT Population with Viral Hepatitis B or Autoimmune Hepatitis
Figure 7.4	INR: Mean Plot to Day 21	ITT Population with Viral Hepatitis B or Autoimmune Hepatitis

14.3 Safety Data

14.3.1 Display of Adverse Events

Table 14.2.7.1	Plasma Concentration of ALF-5755	PK Population
Table 14.2.7.2	PK Parameters for ALF-5755	PK Population

14.3.2 Safety Data

Table 14.3.1.1	Treatment-Emergent Adverse Events	Safety Population
Table 14.3.1.2	Treatment-Emergent Adverse Events, by SOC and PT	Safety Population
Table 14.3.1.3	*Severe Treatment-Emergent Adverse Events, by SOC and PT*	*Safety Population*
Table 14.3.1.4	*Serious Treatment-Emergent Adverse Events, by SOC and PT*	*Safety Population*
Table 14.3.1.5	*Drug-Related Treatment-Emergent Adverse Events, by SOC and PT*	*Safety Population*
Table 14.3.1.6	*Serious Drug-Related Treatment-Emergent Adverse Events, by SOC and PT*	*Safety Population*
Table 14.3.1.7	*Treatment-Emergent Adverse Events Leading to Withdrawal from the Study, by SOC and PT*	*Safety Population*

14.3.3 Listings of Deaths, Other Serious and Significant Adverse Events

Table 14.3.2.1	Listing of Deaths
14.3.3	*Narratives of deaths, other serious and certain other significant adverse events (to be presented in the CSR)*

14.3.4 Narratives of Deaths, Other Serious and Certain Other Significant Adverse Events

Table 14.3.2.2	Listing of Serious Adverse Events	
Table 14.3.2.3	Listing of Withdrawals Due to Adverse Events	

14.3.5 Laboratory Values

Table 14.3.5.1	Haematology	Safety Population
Table 14.3.5.2	Biochemistry	Safety Population
Table 14.3.5.3	*Urinalysis*	*Safety Population*
Table 14.3.5.4	*Plasma Concentration of anti-ALF-5755 Antibodies*	*Safety Population*

14.3.6 Abnormal Laboratory Value Listing (each patient)

Table 14.3.4.1	Listing of Abnormal Laboratory Values
Table 14.3.4.2	Listing of Abnormal Laboratory Values

14.3.7 Clinical Examination, Vital Signs and ECG

Table 14.3.6	*Clinical Examination*	*Safety Population*
Table 14.3.7	*Vital Signs*	*Safety Population*
Table 14.3.8	*12-lead Electrocardiogram*	*Safety Population*

14.4 PK Data

Table 14.2.7.1	Plasma Concentration of ALF-5755	PK Population
Table 14.2.7.2	PK Parameters for ALF-5755	PK Population

15 REFERENCE LIST

Christa, L., M. Felin, et al. (1994). "The human HIP gene, overexpressed in primary liver cancer encodes for a C-type carbohydrate binding protein with lactose binding activity." FEBS Lett 337(1): 114-8.

Cox NR & Mohanty SR, Acute Liver Failure, Hospital Physician July/August 2009.

Hoofnagle JH, Carithers RL Jr, Shapiro C, Ascher N. Fulminant hepatic failure: summary of a workshop. Hepatology. Jan 1995;21(1):240-52.

Iovanna, J. L. and J. C. Dagorn (2005). "The multifunctional family of secreted proteins containing a C-type lectin-like domain linked to a short N-terminal peptide." Biochim Biophys Acta 1723(1-3): 8-18.

Kondo, T., T. Suda, et al. (1997). "Essential roles of the Fas ligand in the development of hepatitis." Nat Med 3(4): 409-13.

Lasserre, C., L. Christa, et al. (1992). "A novel gene (HIP) activated in human primary liver cancer." Cancer Res 52(18): 5089-95.

Lee, W. M., R. H. Squires, Jr., et al. (2008). "Acute liver failure: Summary of a workshop." Hepatology 47(4): 1401-15.

Lieu, H. T., F. Batteux, et al. (2005). "HIP/PAP accelerates liver regeneration and protects against acetaminophen injury in mice." Hepatology 42(3): 618-26.

Lieu, H. T., M. T. Simon, et al. (2006). "Reg2 inactivation increases sensitivity to Fas hepatotoxicity and delays liver regeneration post-hepatectomy in mice." Hepatology 44(6): 1452-64.

Michalopoulos, G. K. and M. C. DeFrances (1997). "Liver regeneration." Science 276(5309): 60-6.

Polson, J. and W. M. Lee (2005). "AASLD position paper: the management of acute liver failure." Hepatology 41(5): 1179-97.

16 APPENDICES

16.1 Study Information

16.1.1 Protocol and Protocol Amendments

16.1.2 Sample Case Report Form

16.1.3 List of ECs or IRBs and Representative Written Information for Patient and Sample Consent Forms

16.1.4 List and Description of Investigators and Other Important Participants in the Study, Including *Curricula Vitae* or Equivalent Summaries of Training and Experience Relevant to the Performance of the Clinical Study

16.1.5 Signatures of Principal or Coordinating Investigator(s) or Alfact Responsible Medical Officer

16.1.6 Listing of Patients Receiving Test Drug(s)/Investigational Product(s) from Specific Batches

16.1.7 Randomisation Scheme and Codes

16.1.8 Audit Certificates

16.1.9 Documentation of Statistical Methods

16.1.10 Documentation of Inter-Laboratory Standardisation Methods and Quality Assurance Procedures

16.1.11 Publications Based on the Study

16.1.12 Important Publications Referenced in the Report

16.2 Patient Data Listings

16.3 Case Report Forms

16.3.1 Case Report Forms for Deaths, Other Serious Adverse Events and Withdrawals for Adverse Events

16.3.2 Other Case Report Forms Submitted

16.4 Individual Patient Data Listings
